# Abstract

**DOI:** 10.1080/17590914.2025.2541459

**Published:** 2025-09-05

**Table:** 

OR01.01 – Iron Overload Drives Microglia Ferroptosis in White Matter Degeneration in Aging Brains	37
OR01.02 – Synaptic Input and Ca^2+^ Activity in Zebrafish Oligodendrocyte Precursor Cells (OPCs) Contribute to Myelin Sheath Formation	38
OR01.03 – Acidic Nanoparticles Prevent HIV Pre-exposure Prophylaxis (PrEP) Impairment of Oligodendrocyte Maturation	39
OR01.04 – Characterization of a Subpopulation of Astrocyte Progenitor Cells in the Neonatal Subventricular Zone	40
OR01.05 – Deletion of Astrocytic Vesicular Nucleotide Transporter Increases Anxiety and Depressive-Like Behavior and Attenuates Motivation for Reward	41
OR01.06 – An Endolysosomal Protein Mediates Neuron-to-Glia Lipid Signaling to Regulate Glial Bioenergetics and Lipid Mobilization	42
OR02.01 – Neuronal Ensemble in the Medial Orbitofrontal Cortex as a Brake to Excessive Binge Alcohol Drinking.	43
OR02.02 – Small GTPase Rab11 Is Required for Proper Cerebellar Granule Cell Development, Anterior Lobule Formation and Adaptive Motor Function in Mice	44
OR02.03 – Targeting the Meningeal Lymphatic Vasculature to Alleviate Symptoms of Autism Spectrum Disorder in a Mouse Model of Fragile X Syndrome	45
OR02.04 – Cerebrospinal Fluid Extracellular Vesicle miRNAs and Synaptic Dysfunction in Alzheimer’s Disease	46
OR02.05 – Inhibition of p38α MAPK Rescues Behavior and Synaptic Function in a Mouse Model of Mixed Vascular and Amyloid Pathologies	47
OR02.06 – The Role of Wnk Kinase in Axon Degeneration	48
P01.001 – Profiling Glial Cell Surface Molecules That Enable the Engulfment of Neurons	49
P01.002 – Metabolic Rewiring During Remyelination: Stable Isotope Tracing Reveals Increased De Novo Lipogenesis and Identifies Serological Markers of Brain Metabolism	50
P01.003 – Investigating Lanthionine Ketimine Ethyl Ester’s Role in Remyelination and Neuroprotection in the Cuprizone Model of De-remyelination	51
P01.004 – Metabolomic Profiling of Greenhouse-Grown Cultivars of Centella asiatica, a Herb Touted for Improving Memory and Preventing Cognitive Decline	52
P01.005 – Downregulation of Pantothenate Kinase and Upregulation of Pantothenate in Liver Kinase B1 Deficient Mouse Astrocytes	53
P01.006 – Timecourse of Activity-Dependent Endogenous Opioid Peptide Gene Transcription After Seizures in the Mouse Hippocampus	54
P01.007 – Targeted Overexpression of Cyclooxygenase-2 in the CA3 Region of the Hippocampus Is Sufficient to Suppress Global Hyperexcitation in the Brain	55
P01.008 – Chemogenetic Manipulation of Astrocytic Calcium Signaling and Neuroinflammation in Demyelinating Disease	56
P01.009 – The Guidance Cue Receptor, PlexinA1, Plays Differential Roles in the Development of the Mouse Embryonic Spinal Commissural Neurons	57
P01.010 – Niemann Pick Disease Type C1 Affects the Concentration and Cargo of Extracellular Vesicles in Patient Samples	58
P01.011 – Neuronal Subtype Vulnerability to Microglial-Induced Synapse Loss in Neuroinflammation	59
P01.012 – TTYH1 Regulates Autophagy in Astrocytes via Endolysosomal Processing of Neuron-Derived Signaling Lipids	60
P01.013 – Targeting the Pathogenic Synergy of Ceramide and S1P in Alzheimer’s Disease	61
P01.014 – The Effect of Astrocyte Syncytium on Neuronal Excitability and Neurovascular Coupling in the Mouse Cortex	62
P01.015 – Matrix Metalloproteinase-1 and Ninjurin a Govern Glial Responses to Neurodegeneration	63
P01.016 – Maintenance of Engrafted Human OPC Fate via Lentiviral Noggin Expression in a Rabbit Model of Chronic Demyelination	64
P01.017 – Lack of Functional Hippocampal Mossy Cell Inputs Accelerates Adult-Born Dentate Granule Cell Maturation	65
P01.018 – Punishment-Associated Compulsive Methamphetamine Intake and Abstinence Are Associated With Differential Hydroxymethylation of miRNAs in the Nucleus Accumbens of Rats	66
P01.019 – Modified Excitability and Locomotor Behavior in Mice With Conditional Deletion of the Anion Channel Subunit LRRC8A in Interneurons	67
P01.020 – Pediatric OSA and Stem Cell Function	68
P01.021 – Characterization of Aβ and Phosphorylated Tau Changes Across the Gut-Brain Axis of AD and Control Individuals	69
P01.022 – Exploring the Mechanisms of STX, a Novel Estrogen Receptor Modulator That Protects Against Amyloid-Beta Neurotoxicity in Alzheimer’s Disease Models	70
P01.023 – Calcium Signaling in Schwann Cells Development and Myelination	71
P01.024 – Discoidin Domain Receptor Signaling Regulates Ensheathment and Caliber of Peripheral Axons	72
P01.025 – B Cell Depletion Reduces Chronic but Not Acute Cognitive Deficits After Prefrontal Stroke in Aged Female Mice	73
P01.026 – The Antiretroviral Drug Dolutegravir Inhibits Oligodendrocyte Maturation In Vitro	74
P01.027 – Diminished Tetrahydrobiopterin (BH4) in Multiple Sclerosis and Its Effects on Oligodendrocytes and CNS Myelination	75
P01.028 – Dock1 Functions in Schwann Cells to Regulate the Development, Maintenance, and Repair of the Peripheral Nervous System	76
P01.029 – Differential Responses of Reactive Astrocytes to Antagonists of CSF1 Receptor in a CLN2 Mouse Model	77
P01.030 – The Muscle-Brain Axis of Resilience Explored Through Differential Gene Expression in Muscle and Brain of the Hibernating Arctic Ground Squirrel	78
P01.031 – Senolytic Therapy Maintains BBB Integrity, Alleviates Cerebral Hypometabolism and Stabilizes Microglia Homeostatic Subtype in the PS19 Mouse Model	79
P01.032 – Harnessing Spatial Transcriptomics to Investigate the Intersection of Senescence and Inflammation in Neurodegeneration	80
P01.033 – Investigating PC12 Cell Differentiation to Sympathetic Neuron-Like Phenotype Through Single-Cell Proteomics Analysis	81
P01.034 – Oligodendrocyte-Derived Carnosine Protects the CNS From Lipid Peroxidation	82
P01.035 – Will Chronic Inhibition of Dual Leucine Zipper Kinase (DLK) Be Neuroprotective and Restore Neuronal Function in Aged-Demyelination?	83
P01.036 – Investigating Astrocyte Endfoot Formation Dynamics During Development and Across Species	84
P01.037 – Impact of Sound Repetition Rate on Cortical Development and Behaviors in Young Fmr1 KO Mice	85
P01.038 – Global Cerebral Ischemia Decreases Dendritic Spine Density, Increases Microglial Reactivity and Microglial/Synapse Co-localization in a Mouse Model of Cardiac Arrest	86
P01.039 – Efforts to Mitigate Neurodegeneration in a Model of Traumatic Optic Neuropathy	87
P01.040 – The Effect of Neonatal Intermittent Hypoxia on Brain Lipid Metabolism	88
P01.041 – TRPML1 Modulates the Oligodendrocyte Cytoskeleton via Rac1	89
P01.042 – Modeling Phenotypic Onset in a Human iPSC Model of CLN3 Batten Disease	90
P01.043 – A Conserved Stress Response Associated With Dark Microglia Drives Neurodegeneration in Alzheimer’s Disease	91
P01.044 – ProNGF Elicits Retrograde Axonal Degeneration of Basal Forebrain Neurons via p75NTR and Induction of APP	92
P01.045 – Therapeutic Potential of Targeting Heparan Sulfate Proteoglycan Sulfatases in an EAE Demyelination Model	93
P01.046 – Altered Activation in the Dentate Gyrus of Seizure-Prone CACNA2D2 Knockout Mice	94
P01.047 – Mitochondrial Functional Assays in a Canine Survival Model of Hypothermic Circulatory Arrest	95
P01.048 – Chemogenetic Manipulation of Astrocyte Functions During Postnatal Brain Development	97
P01.049 – Centella asiatica Alters Cognition in Aged Male and Female Mice, and Anxiety and Plasma GABA and Corticosterone Only in Females	98
P01.050 – RNA Sequencing Identifies Sex-Related Differences in Transcriptional Signatures in the Female and Male Control Rats	99
P01.051 – Transgenic Mouse Models to Define the Role of Microglia in Glioblastoma (GBM) Progression	100
P01.052 – Delayed Phospho-BTK Inhibition Reduces the Reactive Phenotype of Microglia and Oligodendrocytes and Improves Myelin Structure After Stroke in Neonatal Mice	101
P01.053 – Characterizing Microglial Dynamics During White Matter Ischemic Injury: An Age and Sex Dependent Response	102
P01.054 – Effects of Neonatal Ethanol Exposure on Prefrontal Cortex Astrocyte Gene Expression In Vivo	103
P01.055 – Receptor-Mediated Activation of G12/13 Signaling in POMC Neurons Regulates Key Metabolic Functions	104
P01.056 – What Regulates the Morphogenesis of Astrocytes?	105
P01.057 – Examining the Role of αVCAM-1 in Attenuating the Neuroinflammatory Response in High-Fat Diet Mice After Stroke	106
P01.058 – Elucidating Downstream Pathways of the Soluble Amyloid Precursor Protein and GABAB Receptor Interaction	107
P01.059 – PAK2 Is Necessary for Myelination in the Peripheral Nerve System	108
P01.060 – The Role of Inflammatory Oligodendrocyte Lineage Cells in CNS Demyelination	109
P01.061 – Exocytosis of ATP in Astrocytes Regulates Amyloid-Beta Pathology	110
P01.062 – Investigating the Utility of iPSC-Derived Choroid Plexus Organoids to Yield Fluid Biomarkers in SCA1 Disease Modeling	111
P01.063 – Parabrachial Extended Amygdala Circuit Activity Is Heightened Following Repeated Stress	112
P01.064 – How Do Glia Regulate Synapse Development?	113
P01.065 – Myelination and Lipid Metabolism in the Adolescent HIV-1 Transgenic Rat Brain	114
P01.066 – Circadian Alignment of Intermittent Fasting Is Crucial for Cerebral Ischemic Tolerance	115
P01.067 – Tweek-Dependent Formation of ER-PM Contact Sites Enables Astrocyte Phagocytic Function and Remodeling of Neurons	116
P01.068 – Defining the Role of B-raf and mTOR Signaling in Spinal Cord Oligodendroglia	117
P01.069 – High-Mobility Group Box-1 HMGB1-Mediates Isenecense And Contributes To Cognitive Dysfunctions In Tauopathies	118
P01.070 – Investigation of Cyp46a1 and Cholesterol Homeostasis as a Novel Therapeutic Target for Rett Syndrome Phenotypes in Mecp2-Mutant Mice	119
P01.071 – Choline Transporter-Like Protein 1 (CTL1) Regulates Intracellular Choline Metabolism and Phosphoinositide Signaling in Schwann Cells	120
P01.072 – TFEB/3 Govern Repair Schwann Cell Generation and Function Following Peripheral Nerve Injury	121
P01.073 – Neuroprotective Properties of the Methyl Donor Betaine in EAE	122
P01.074 – Anti-PLP1 IgG1 Cloned From Patients With Multiple Sclerosis Impedes Oligodendrocyte Differentiation and Induces Myelin Pathology Independently of Demyelination	123
P01.075 – Elucidating Interactions Between Triggering Receptor Expressed on Myeloid Cells 2 and Apolipoprotein E in Microglial Activation With Molecular Dynamics Simulations	124
P01.076 – Characterization of Neurogenic Fate Decisions in TSC2-/- Induced Pluripotent Stem Cell-Derived Model	125
P01.077 – Respiratory Infection With Influenza a Virus Alters Glial Metabolism	126
P01.078 – Respiratory Infection With Influenza a Virus Delays Remyelination Following Cuprizone-Induced Demyelination	127
P01.079 – Mice Carrying a Novel NAMPT Mutation Exhibit Metabolic Impairments	128
P01.080 – Analysis of Senescent Cell Development and Depletion in Mice After Experimental Autoimmune Encephalomyelitis (EAE) Induction	129
P01.081 – Genetic Pathways That Drive Axon Loss	130
P01.082 – The Role of Aurora B Kinase in the Development of Neuron Dysfunction in Amyotrophic Lateral Sclerosis	131
P01.083 – Regional Differences in Oligodendroglial Cholesterol Acquisition and Myelin Lipid Composition	132
P01.084 – A Novel CD27 + CD138+ B Cell Subset Localizes to the Brain in Aged Mice	133
P01.085 – Dissection of Inter-disorder Astrocyte Reactivity Reveals a Novel Astrocyte Subtype That Regulates White Matter Repair	134
P01.086 – Identifying Lanthionine Ketenamine (Ethyl Ester) (Phosphonate) Derivatives for Relative Maturation and Proliferation Effects in OPCs	135
P01.087 – RNA-Seq of Extracellular Vesicle (EV) RNA Separated From a Small Volume of Human Cerebral Spinal Fluid (CSF)	136
P01.088 – Development and Characterization of a Severe, Dietary Fat-Free Cuprizone Model of Remyelination	137
P01.089 – Th17 Cells Reprogram Astrocytes Through a JAK1 Dependent Process That Contributes to Autoimmune Neuroinflammation.	138
P01.090 – Müller Glia Glutamate Metabotropic Receptors Regulation Upon Excitotoxic Conditions: Correlation to the Dark Cycle	139
P01.091 – Trem2 Mediates Sulfatide Deficiency-Induced Microglia-, but Not Astrocytes-Mediated Neuroinflammation or Lipid Homeostasis Disruption	140
P01.092 – Examining TBI-Induced Astrocyte Heterogeneity Across Spatial and Temporal Boundaries	141
P01.093 – Mild Traumatic Brain Injury Induces an Astrocyte Atypical Response Astrocytes Mediated by Protein Degradation	142
P01.094 – ER Stress Underlies Altered Cell Fate During Brain Development in a Human Model of the Cortical Malformation Syndrome Tuberous Sclerosis	143
P01.095 – CRISPR Inactivation Strategies for ALS, Charcot-Marie-Tooth, and Other Dominant Neurogenetic Diseases	144
P01.096 – CK2 Inhibition Preconditions White Matter Against Ischemia by Differentially Regulating CDK5 and AKT/GSK3β Pathways	145
P01.097 – The Role of NOX in Post-ischemic Protection of White Matter Against Ischemia	146
P01.098 – Herpes Simplex Virus Type-1 (HSV-1) Accelerates Alzheimer’s Disease Progression: Targeting Vulnerable Brain Regions and Amplifying Neuroinflammation	147
P01.099 – The mGluR5 Agonist, CHPG, Enhances Differentiation of Developing Human Oligodendrocyte Lineage Cells	148
P01.100 – Microglial Lipoprotein Lipase Regulates Myelin Processing and Is Elevated in Multiple Sclerosis	149
P01.101 – TFEB Treatment in the Dorsal Hippocampus of Obese Female and Male 5xFAD Mice	150
P01.102 – Aligned Nanofibrillar Collagen Scaffolds Promote Repair Schwann Cell Phenotype in Peripheral Nerve Regeneration	151
P01.103 – Region Specific Inflammatory Response in Cerebrovascular Patients	152
P01.104 – Physiologic, Biomarker and Clinical Outcomes Associated With Ketamine Exposure After Traumatic Brain Injury, a Single Center Retrospective Study	153
P01.105 – Voltage-Gated Calcium Channels Regulate Developmental Myelination in Oligodendrocytes	154
P01.106 – Loss of Carnitine Palmitoyl Transferase 2 (CPT2) Enhances Proliferation Post Traumatic Brain Injury in Adult Mice	155
P01.107 – Oxidative Stress Triggers Secretion of Neurotoxic Ceramide-Rich Extracellular Vesicles (CREVs)	156
P01.108 – The Impact of Reduced Glial Plasma Membrane Cholesterol on Subcellular Actin Dynamics	157
P01.109 – Store-Operated Calcium Entry-Dependent Signaling in Adult OPCs Acts to Delay Oligodendrocyte Differentiation Following Demyelination	158
P01.110 – The Function of Sex-Specific Extracellular Vesicles in the Pathology of Alzheimer’s Disease	159
P01.111 – Characterization of the Inflammasome in the CLN2 Mouse Model	160
P01.112 – A Translational Readthrough Variant of AQP4 Has Altered Gliovascular Properties and Modifies Neurological Diseases	161
P01.113 – MiRNAs as Biomarkers for and Mediators of Alzheimer’s Disease	162
P01.114 – Development and Validation of TRPML1 Assays on Automated Patch Clamp Platforms	163
P01.115 – Increasing Adult Oligodendrogenesis Through the Deletion of Glial-Expressed Voltage-Gated Calcium Channel Subunits	164
P01.116 – System-Based Integrated Metabolomics and microRNA Analysis Identifies Potential Molecular Alterations in Human X-linked Cerebral Adrenoleukodystrophy Brain	165
P01.117 – Microglia-Mediated Synaptic Dysfunction Contributes to Chemotherapy-Related Cognitive Impairment	166
P01.118 – Interactions Between TDP-43 and Amyloid Beta Pathology in a Drosophila Model of Alzheimer’s Disease	167
P02.01 – Thyroid Hormone Decreases Cx43-Containing Extracellular Vesicle Production in Cultured Astrocytes	168
P02.02 – Oxygen Availability Regulates PG Production Independently of Cyclooxygenase Induction Upon Stimulation With LPS	169
P02.03 – Adhesion GPCR-Dependent Communication Across Cells	170
P02.04 – An Alternative Fixation Method for Preventing Post-mortem Prostanoid Increase in the Brain	171
P02.05 – The Role of Mechanosensation in Central Nervous System Myelination	172
P02.06 – Facilitating Oligodendrocyte Maturation and Safeguarding Myelin Integrity in Multiple Sclerosis via the TET1-BHMT Axis	173
P02.07 – A Molecular Dissection of Complement in Demyelinating Disease	174
P02.08 – Fever-Like Temperatures Without Immune Activation Improve Cognitive Deficits in the Scn2a Autism Mouse Model via Increases in K + Channel Activity	175
P02.09 – Measuring ATP:ADP in Neurons and Astrocytes Using PercevalHR In Vitro	176
P02.10 – Recovery of Astrocyte Calcium Signaling and Cerebrovascular Dynamic From General Anesthesia	177
P02.11 – Multi-omics Analyses of Synapses From C9ORF72 ALS/FTD Cortical Neurons	178
P02.12 – Neurochemical Perturbations and Synaptic Dysfunction Associated With Inhaled Environmental and Occupational Toxicants	179
P02.13 – TSG-6 Mediated Extracellular Matrix Modifications Regulate Hypoxic-Ischemic Brain Injury	180
P02.14 – Reactive Astrocytes May Underlie Diminished Neurovascular Coupling After Stroke	181
P02.15 – Unravelling the Metabolic Changes Mediated by Ketone Body Utilization in the Nervous System	182
P02.16 – Cell Synchronization and Glial Neurotransmitter Transporters in Retinal Müller Cells: Role in Excitotoxicity	183
P02.17 – Chronic In Vivo Calcium Imaging of Registered Spinal Neurons to Characterize Mechanical Sensitization During a Month of Inflammatory Pain	184
P02.18 – Extracellular Vesicles From Toxoplasma gondii Infected Neurons Decrease GLT-1 Protein Expression	185
P02.19 – Lanthionine Ketimine Ethyl Ester Increases Oligodendrocyte Proliferation Both In-Vitro and In-Vivo	186
P02.20 – Investigating the Roles of Dark/Clec7a + Microglia During Early Postnatal Development in a Mouse Model of Maternal Immune Activation	187
P02.21 – Fluid Flow Shear Stress Induces Acid Sphingomyelinase-Mediated Secretion of Neurotoxic Ceramide-Rich Extracellular Vesicles From Cilia in Glial Cells	188
P02.22 – Biomarker and Edema Attenuation in IntraCerebral Hemorrhage (BEACH): Phase 2a Proof-of-Concept Trial of a Novel Anti-neuroinflammatory Small Molecule Drug Candidate	189
P02.23 – HIV Antiretroviral Drugs Trigger Stress Granule Formation via the PERK Activated Integrated Stress Response in Differentiating Oligodendrocytes	190
P02.24 – Astrocytes Dysregulate GABAergic Inhibition to Impair Auditory Processing in Fragile X Syndrome	191
P02.25 – Investigating the Role of Copper in Oligodendrocyte Myelination	192
P02.26 – PTEN-Induced Kinase 1 Modulates Neuronal Development and Plasticity Through Calcium/Calmodulin-Dependent Protein Kinase II and IV	193
P02.27 – Investigating Ischemic Vascular Dysfunction in Alzheimer’s Disease	194
P02.28 – Delineating the Mechanisms Leading to Aberrant Lipid Storage and Trafficking Defects in Astroglia Models of Cholesterol Synthesis Disorders	195
P02.29 – Role of Autophagy and Fatty Acid Binding Protein 5 During Docosahexaenoic Acid Inhibition of Palmitic Acid-Induced Lipotoxicity	196
P02.30 – The Effect and Mechanism of GDNF Released From Reactive Astrocytes on Neuronal and Brain Protection After Ischemic Stroke	197
P02.31 – The Sphingosine-1-Phosphate Receptor 1 Antagonist Ponesimod Reduces Neuroinflammation via Microglial Aβ Clearance	198
P02.32 – Effects of Centella asiatica Water Extract on Cerebrovascular Function in Mice	199
P02.33 – Developing Tools to Study Cholesterol Metabolism in Microglia During Demyelination	200
P02.34 – Kindling Epileptogenesis Induces Broad Alterations in the Hippocampal Transcriptome of Mice Lacking TIA1 Cytotoxic Granule Associated RNA Binding Protein	201
P02.35 – Metabolic Derangement Resulting From Diet-Induced Obesity Is Dependent on Estrogen in a Mouse Model of Menopause	202
P02.36 – A Novel Mechanism for Brain Prostanoid Regulation Through Oxygen Availability Under Ischemia	203
P02.37 – Fractalkine Overexpression via rAAVs Differentially Regulate Microglial Activation and Vascular Damage in the Diabetic Retina	204
P02.38 – TMEM106B Core Deposition Associates With TDP-43 Pathology and Is Increased in Risk SNP Carriers for Frontotemporal Dementia	205
P02.39 – Inferring Axon Diameters in the Mouse Corpus Callosum Using Diffusion MRI: Comparison of ROI-Based and Voxel-Based Analysis	206
P02.40 – Neuroactive Effects of Ashwagandha: From Ayurvedic Medicine to Cellular Bioactivity and Mass Spectrometry	207
P02.41 – A Novel Nanoparticle-Mediated Delivery of Estrogen to Protect Neurons and Improve Functional Recovery in Spinal Cord Injury	208
P02.42 – PMP22 Null Mutant Mice Exhibit Morphological and Functional Deficits in the Respiratory System	209
P02.43 – The Natural Compounds Oridonin and Shikonin Exert Beneficial Effects on Reactive Microglia, Independent of Their Inflammasome Inhibitory Activity	210
P02.44 – N6-Cyclohexyladenosine Is Better Than Meperidine and Buspirone at Suppressing Metabolism During TTM32 but Does Not Improve Outcome Post Cardiac Arrest	211
P02.45 – The Role of Connexin43 Mediated Coupling in Neuronal Activity	212
P02.46 – Distinct Odor and Reward-Evoked GABAergic Neuronal Activity in the Basal Forebrain Influences Odor Perception and Decision-Making	213
P02.47 – NMNAT2: A Guardian of Axonal Integrity and Cortical Energy Balance in the Brain	214
P02.48 – Differentiation State Impacts the Interaction of mTOR and TFEB in Oligodendrocytes	215
P02.49 – Serpina3n/SERPINA3 in LPS-Induced Neonatal Sepsis: Liver-Brain Axis	216
P02.50 – Diiodothyropropionic Acid Facilitates Oligodendrocyte Differentiation and Myelination to Enhance Neuroprotection and Neurorepair in the Central Nervous System	217
P02.51 – Delayed Administration of an Angiotensin(II) Type(2) Receptor Agonist for the Treatment of Ischemic Stroke: A Preclinical Trial in Hypertensive Rats	218
P02.52 – Sex and Viral Infection-Induced Inflammation as Contributing Factors to Alterations in Cognitive Performance and Microglial Response in Aged C57BL6/J Mice	219
P02.53 – The Beneficial Effects of NLY01 on Astrocyte Coverage and Functional Recovery in a Mouse Model of Stroke	220
P02.54 – Transcriptome Analysis in Lumbosacral Dorsal Root Ganglia Revealed Sex-Specific Mechanisms Underlying Visceral Hypersensitivity	221
P02.55 – The Role of Oligodendrocyte Specific Serpina3n in Physiological and Pathological Condition	222
