# Supplementary material for: Abstract
Source: ASN Neuro. 2025 Sep 5;17(1):2541459. doi: 10.1080/17590914.2025.2541459 (PMC12416199; doi:10.1080/17590914.2025.2541459)
Supplement: Related texts

# Abstract: OR01.01 – Iron Overload Drives Microglia Ferroptosis in White Matter Degeneration in Aging Brains

**Published:** 2025-09-05

**Authors:** Philip Adeniyi, Xi Gong, Ellie MacGregor, K. Degener-O’Brien, E. McClendon, Mariel Garcia, J. Russell, O. Romero, Taasin Srivastava, Jerry Miller, C. Dirk Keene, Stephen Back

**Affiliations:** a Department of Pediatrics, Oregon Science and Health University, Portland, OR, USA; b Department of Laboratory Medicine and Pathology, University of Washington School of Medicine, Seattle, WA, USA; c Allen Institute for Brain Science, Seattle, WA, USA; d Department of Neurology, Oregon Health and Science University, Portland, OR, USA

## Abstract

As the brain ages, recurrent cerebral white matter injury (WMI) causes disruptions in white matter integrity and myelination, contributing to the development of cognitive impairment seen in Alzheimer’s disease (AD) and vascular dementia. We found a high percentage of microglia in aging human WMI that exhibited degenerative changes associated with the phagocytosis of myelin debris. These degenerative microglia (DM) were characterized by enrichment in the iron-binding protein light chain ferritin and the accumulation of PLIN2-labeled lipid droplets. Additionally, they manifest lipid peroxidation injury and an elevated expression of TOM20, a mitochondrial translocase, and oxidative stress sensor. Analysis of the DNA fragmentation marker phospho-histone H2A.X suggests that iron-enriched DM are susceptible to senescence-related degeneration, indicating a mechanism consistent with ferroptosis. In comparison to gray matter neurodegeneration, a distinctive set of ferroptosis-related genes, involving iron-mediated lipid dysmetabolism and oxidative stress, were identified as selectively expressed in WMI. Ferroptosis appears to be a significant mechanism of WMI in AD and vascular dementia. The failure of remyelination is linked to a substantial population of aging microglia sensitive to oxidative stress induced by phagocytosis and abnormal accumulation of myelin debris. This sensitivity promotes microglial lipid peroxidation injury that promotes ferroptosis-related degeneration.

# Abstract: OR01.02 – Synaptic Input and Ca^2+^ Activity in Zebrafish Oligodendrocyte Precursor Cells (OPCs) Contribute to Myelin Sheath Formation

**Published:** 2025-09-05

**Authors:** Jiaxing Li, Tania Miramontes, Tim Czopka, Kelly Monk

**Affiliations:** a OHSU, Portland, OR, USA; b Centre for Clinical Brain Sciences, University of Edinburgh, Edinburgh, UK

## Abstract

In the nervous system, only one type of neuron-glial synapse is known to exist: that between neurons and oligodendrocyte precursor cells (OPCs), yet their composition, assembly, downstream signaling, and in vivo functions remain largely unclear. Here, we address these questions using in vivo microscopy in zebrafish spinal cord and identify postsynaptic molecules PSD-95 and Gephyrin in OPCs. The puncta containing these molecules in OPCs increase during early development and decrease upon OPC differentiation. These puncta are highly dynamic and frequently assemble at “hotspots.” Gephyrin hotspots and synapse-associated Ca^2+^ activity in OPCs predict where a subset of myelin sheaths form in differentiated oligodendrocytes. Further analyses reveal that spontaneous synaptic release is integral to OPC Ca^2+^ activity, while evoked synaptic release contributes only in early development. Finally, disruption of the synaptic genes dlg4a&b, gphnb, and nlgn3b impairs OPC differentiation and myelination. Together, we propose that neuron-OPC synapses are dynamically assembled and can predetermine myelination patterns through Ca^2+^ signaling.

# Abstract: OR01.03 – Acidic Nanoparticles Prevent HIV Pre-Exposure Prophylaxis (PrEP) Impairment of Oligodendrocyte Maturation

**Published:** 2025-09-05

**Authors:** Caela Long, Lindsay Festa, Melanie Cruz, Claire Mitchell, Kelly Jordan-Sciutto, Judith Grinspan

**Affiliations:** a University of Pennsylvania, Philadelphia, PA, USA; b Children’s Hospital of Philadelphia, Philadelphia, PA, USA

## Abstract

Each year, 13- to 24-year-olds disproportionately compose the number of individuals diagnosed with human immunodeficiency virus (HIV) in the United States. Even with effective peripheral viral suppression by antiretroviral therapy (ART), this population is at risk for significant life-long behavioral, cognitive, and motor impairments as approximately 30–50% of HIV + individuals develop HIV-associated neurocognitive disorders (HAND). White matter abnormalities are a hallmark of HAND and transcriptome analysis have found a significant decrease in the expression of genes associated with oligodendrocytes in the white matter of ART treated, HIV + individuals. Our lab has demonstrated that even in the absence of HIV, select antiretroviral drugs of the integrase inhibitor (INSTI) and protease inhibitor (PI) classes inhibit OL differentiation in vitro. PrEP, a combination of emtricitabine (FTC) and tenofovir disoproxil fumarate (TDF) of the NRTI antiretroviral class, is an effective method to prevent the transmission of HIV in adolescents at substantial risk for acquiring HIV. However, as myelination continues through adolescence and into adulthood, it is important to understand the effect of PrEP on developing white matter, which has not been investigated. Here, we report that PrEP treatment significantly reduced oligodendrocyte maturation in adolescent rats. Specifically, adolescent rats treated with PrEP from 3 to 6-weeks of age had significantly fewer mature oligodendrocytes in the rostral corpus callosum and frontal cortex and significantly less proteolipid protein (PLP) expression in the frontal cortex at 6-weeks-old compared to vehicle treated controls. Primary rat oligodendrocyte experiments revealed that PrEP inhibited oligodendrocyte differentiation by deacidifying lysosomes resulting in significantly fewer acidic and proteolytically functional lysosomes. Lysosomes are critical for processing and transporting large quantities of lipids and proteins necessary for the generation of the myelin membrane from the plasma membrane of oligodendrocytes. Specifically, PLP is known to be transported through lysosomes as a synthesized protein before being inserted into the plasma membrane of mature oligodendrocytes. Cell surface protein biotinylation assays revealed that oligodendrocytes treated with PrEP have significantly less cell surface PLP and PLP colocalized more with the lysosomal marker LAMP1 compared to vehicle. Lastly, acidic nanoparticle (PLGA) treatment reacidified lysosomes and restored oligodendrocyte differentiation in the context of PrEP treatment both in vivo and in vitro. Adolescent rats that received intranasal administration of acidic nanoparticles at the same time as PrEP from 3 to 6-weeks of age had significantly more mature oligodendrocytes (ASPA + cells) in the rostral corpus callosum and frontal cortex at 6-weeks-old compared to PrEP only treated controls. Furthermore, compared to PrEP only treated animals, PrEP treated animals that co-received acidic nanoparticles had significantly more proteolipid protein (PLP) expression in the frontal cortex. Thus, acidic nanoparticles prevent PrEP impairment of oligodendrocyte maturation in vitro and in vivo. Our findings address a gap in our understanding of the effects of PrEP on myelination in uninfected adolescents. Identifying the mechanisms underlying myelin changes in uninfected adolescents taking PrEP will allow for the development of adjunctive treatments or new prophylactic therapies that protect this uniquely vulnerable population.

# Abstract: OR01.04 – Characterization of a Subpopulation of Astrocyte Progenitor Cells in the Neonatal Subventricular Zone

**Published:** 2025-09-05

**Authors:** Zila Martinez-Lozada, Alain M. Guillem, Isabella Song, B. Michael Robinson

**Affiliations:** a Children’s Hospital of Philadelphia, Philadelphia, PA, USA

## Abstract

Astrocytes, the most abundant non-neuronal cell type in the mammalian brain, have diverse essential functions. Astrocytes rise from radial glial cells that line the ventricular zone (VZ) (embryonic brain), progenitors in the subventricular zone (SVZ) (early postnatal), and NG2+ cells, and they undergo local proliferation in the cortex followed by morphological and functional maturation. Astrocytes are a heterogeneous population of cells with intra- and inter-regional diversity. However, the mechanisms that generate this diversity are unknown. In addition, the environmental cues that direct the migration of the newly born astrocytes and their progenitor cells from the VZ and SVZ to their final location in the cortex have not been identified. There is accumulating data that suggests that the combination of diverse lineages and environmental signals contributes to the generation of astrocyte diversity. Here, we used a double reporter mouse line BAC-GLT1-GFP/8.3-EAAT2-tdT, which in adult mice allows us to identify a subpopulation of cortical astrocytes. We found that in this mouse line, GFP and tdT label two different populations of cells. At postnatal day 1 (P1), GFP + cells are found throughout the brain in a defined position, while the tdT + cells are found in the SVZ and the striatum; no tdT + cells were found in the cortex at this developmental stage. Using organotypic brain cultures and explants, we found that some of the tdT + cells migrate to the cortex postnatally and, after migration, express GFP and the astrocyte marker NFIA. We also found that antagonists of the ionotropic glutamate receptors block the migration of the tdT + cells. In contrast, an inhibitor of the glutamate transporters increases it. Suggesting that glutamate drives the migration of astrocyte precursor cells. These results indicate that subpopulations of astrocyte precursors with different temporal and regional genesis generate cortical astrocytes and that the local environment is required to drive astrocyte migration and maturation.

# Abstract: OR01.05 – Deletion of Astrocytic Vesicular Nucleotide Transporter Increases Anxiety and Depressive-Like Behavior and Attenuates Motivation for Reward

**Published:** 2025-09-05

**Authors:** Qian Huang, Hiu Ham Lee, Bryan Volpe, Lucy Yu, Yahia R. Abuhasan, Lingyun Li, Jeremy S. Yang, Julie Egholm, Allen Li, Alyssa Lee, Sharon Tang, Raddy L. Ramos, C. Ronald Kahn, Emmanuel N. Pothos, Weikang Cai

**Affiliations:** a NYITCOM, USA; b Tufts Medical Center, Boston, MA, USA; c Joslin Diabetes Center, Harvard Medical School, Boston, MA, USA

## Abstract

Astrocytes are multi-functional glial cells in the central nervous system that play critical roles in modulation of metabolism, extracellular ion and neurotransmitter levels, and synaptic plasticity. Astrocyte-derived signaling molecules mediate many of these modulatory functions of astrocytes, including vesicular release of ATP. In the present study, we used a unique genetic mouse model to investigate the functional significance of astrocytic exocytosis of ATP. Using primary cultured astrocytes, we show that loss of vesicular nucleotide transporter (Vnut), a primary transporter responsible for loading cytosolic ATP into the secretory vesicles, dramatically reduces ATP loading into secretory lysosomes and ATP release, without any change in the molecular machinery of exocytosis or total intracellular ATP content. Deletion of astrocytic Vnut in adult mice leads to increased anxiety, depressive-like behaviors, and decreased motivation for reward, especially in females, without significant impact on food intake, systemic glucose metabolism, cognition, and sociability. These behavioral alterations are associated with significant decreases in the basal extracellular dopamine levels in the nucleus accumbens. Likewise, ex vivo brain slices from these mice show a strong trend toward a reduction in evoked dopamine release in the nucleus accumbens. Mechanistically, the reduced dopamine signaling is likely due to an increased expression of monoamine oxidases. Together, these data demonstrate a key modulatory role of astrocytic exocytosis of ATP in anxiety, depressive-like behavior, and motivation for reward.

# Abstract: OR01.06 – An Endolysosomal Protein Mediates Neuron-to-Glia Lipid Signaling to Regulate Glial Bioenergetics and Lipid Mobilization

**Published:** 2025-09-05

**Authors:** Ching-On Wong

**Affiliations:** a Rutgers University – Newark, Newark, NJ, USA

## Abstract

Brain cells rely on lipid metabolism to meet energy needs, perform signaling functions, and regulate membrane structures. Alterations in lipidomic and bioenergetic profiles have been observed in aging brains and in neurodegenerative diseases, suggesting that reprogrammed metabolic pathways may underlie dyshomeostasis. At the cellular level, glial cells such as astrocytes are responsible for providing metabolic and energetic support to neurons. Excess lipids are stored as neutral lipids in lipid droplets (LDs), which support both glial bioenergetics and biosynthesis of lipid precursors for other brain cells. Thus, elucidating the lipid metabolic pathways in glial cells is fundamental to our understanding of neural homeostasis. During the investigation of an understudied transmembrane protein, we uncovered an endolysosomal process that regulates glial bioenergetics and lipid mobilization. In astrocytes isolated from human and conditional knockout mouse brains and in Drosophila glial cells, we found that Tweety Homolog 1 (TTYH1) is localized to endolysosomes to facilitate autophagy. Loss of TTYH1 orthologs impairs mitochondrial turnover and LD homeostasis. Concomitantly, glial cells adopt a bioenergetic shift towards glycolysis and diminish Apolipoprotein E secretion. At the molecular level, we identified TTYH1 as a critical endolysosomal processor of specific sphingolipids. Importantly, we found that neuron-derived sphingolipids signal astrocytes to dampen autophagy and lipid mobilization via a TTYH1-dependent pathway. Furthermore, in Drosophila models of tauopathy, perturbing glial expression of TTYH1 ortholog alters lipid storage in brain, locomotor activity, and lifespan. Together, our emerging findings reveal a hitherto unrecognized endolysosomal pathway that mediates the neuron-to-glia lipid signaling involved in regulating lipid mobilization and bioenergetics. Deciphering the mechanistic underpinnings of such pathway will aid in identifying actionable targets to alter outcomes of neurodegenerative diseases.

# Abstract: OR02.01 – Neuronal Ensemble in the Medial Orbitofrontal Cortex as a Brake to Excessive Binge Alcohol Drinking

**Published:** 2025-09-05

**Authors:** Pablo Gimenez Gomez, Timmy Le, Susanna Molas, Max Zinter, Timothy Freels, Violeta Duran-Laforet, Travis Thomson, Andrew Tapper, Dorothy Schafer, Gilles Martin

**Affiliations:** a University of Massachusetts Chan Medical School, Worcester, MA, USA

## Abstract

Alcohol abuse is the seventh leading risk factor for both death and the burden of disease. However, the neural basis governing early alcohol consumption remains unclear. It has previously been established that a group of neurons shows activity during alcohol binge drinking consumption, but the role of this neuronal ensemble and its relationship with alcohol consumption has not been established. In this work we have studied the properties of those neurons and their role in binge alcohol consumption. We are using TRAP2 mice (males and females), a mouse line that, in the presence of 4-hydroxitamoxifen, allows permanent genetic access to neurons activated by a specific experience. In TRAP2 mice subjected to the drinking in the dark paradigm (DID) of alcohol consumption, we performed fiber photometry, single-cell RNA-seq, electrophysiology, and iDisco + combined with light-sheet microscopy to understand the characteristics of the neuronal ensemble (cellular type, intrinsic and extrinsic properties, layer location, areas of projection, or transcriptional pattern, among others). We then employed optogenetics, both in the cell body and the axonal projections, to activate and inhibit the ensemble while the mice drink alcohol and determine the amount of alcohol consumed and the blood alcohol content (BEC) to elucidate the role of these neurons. 5% of mOFC neurons are activated by alcohol as it reaches intoxication level (i.e., BEC higher than 80 mg/dL). Reaching intoxication level is key for the activity of the neuronal ensemble, as without this level, those neurons don’t show activity, indicating that the activity of the ensemble is dependent of BEC. Calcium recording using Gcamp and fiber photometry in the neuronal ensemble allowed us to better understand the pattern of activity while the mice drink. Our results show a strong peak of calcium only at the end of the episode of consumption with no response at the beginning further validating that the ensemble only shows activity when mice reach intoxication levels. Single-cell RNAseq shows that the neuronal ensemble is GABAergic. iDisco + combined with Light Sheet microscopy shows that the ensemble projects to Mediodorsal Thalamus (MD) and the Periaqueductal Grey Area (DPAG). Finally, using optogenetics in the cell body of the ensemble in the mOFC, we were able to activate or inhibit the ensemble in real-time when the mouse starts to sip alcohol, showing that the activation of the ensemble using Channelrhodopsin produces a strong decrease in consumption, while their inhibition using Halorhodopsin renders the opposite effect. Finally, when we photostimulate or photoinhibit the axonal projections from the ensemble to MD, we found the same effect, indicating that the axonal projections from the ensemble in the mOFC to the MD acts as a brake, reducing drinking when the mice reach intoxication level. This neuronal ensemble acts as a moderator of alcohol consumption, marking the first time that a neuronal ensemble is proposed as a brake for a complex behavior such as binge alcohol drinking. By acting as a potent brake on drinking behavior, this ensemble emerges as a promising therapeutic target for interventions in alcohol abuse.

# Abstract: OR02.02 – Small GTPase Rab11 Is Required for Proper Cerebellar Granule Cell Development, Anterior Lobule Formation and Adaptive Motor Function in Mice

**Published:** 2025-09-05

**Authors:** Edward Martinez, Jiyeon Baek, Jack DeLucia, Ivor Joseph, Meroline Bazile, Justina Shafik, Michael Shiflett, Nan Gao, Tracy Tran

**Affiliations:** a Rutgers – The State University of New Jersey, Newark, NJ, USA

## Abstract

The mammalian cerebellum contains approximately 80% of the neurons in the brain, the majority of which are cerebellar granule cells. Cerebellar hypoplasia is known to be one of the leading risk factors for autism spectrum disorders (ASD). Rab11, a member of the Rab GTPase family, resides within the recycling endosome and is responsible for trafficking signaling proteins to and from the plasma membrane. Rab11 has been shown to have important roles in controlling crucial developmental and cellular processes such as proliferation, differentiation, and migration in non-neuronal cell types, while also regulating neurite outgrowth in neuronal cells during development. Human patients harboring mutations of the RAB11 gene display intellectual disability and gross anatomical abnormalities in the brain, including the reduction of cerebellar size. However, the mechanisms of how Rab11 controls cerebellar development are not known. Here, we showed for the first time that both of the Rab11 isoforms that are present in the nervous system, Rab11a and Rab11b, are key players in orchestrating multiple cellular processes during cerebellar development. Animals with granule cell precursor-specific deletion of Rab11a (Rab11af/f;Math1-Cre+) or global deletion of Rab11b (Rab11b-/-) have relatively normal cerebellar size and lobule architecture. Double knockouts (dKOs) were generated by crossing the Rab11af/f;Math1-Cre + into the Rab11b-/- background animals. In the dKO mice, severe loss of granule cell precursors numbers resulted in significantly decreased overall cerebellar size, with a dramatic loss of anterior lobules 1–3 persistent to adult ages. Abnormal granule cell proliferation with an increase in cell death was observed during embryonic ages, while differentiation was also impaired in postnatal dKO animals, leading to subsequent phenotypes of decreased external granule layer thickness, perturbed radial glia scaffolds, and ultimately impairment of cell migration and death in the inner granule layer. Rab11a/b dKO animals had increased numbers of dividing granule cell precursors harboring unipolar mitotic spindles in their external granule layer, suggesting a possible mechanism for Rab11 in regulating spindle organization during mitosis in these cells. Finally, Rab11a/b dKO animals exhibited motor behavioral deficits in the accelerating rotarod test and increased anxiety-like behavior in the elevated zero maze test. Overall, our study demonstrated that Rab11a/b isoforms have compensatory roles in regulating the proper production and maintenance of cerebellar granule cells in vivo, which has a profound impact on anterior lobule formation and animal behavior. This work will serve as the basis for future experiments to investigate the role of Rab11 in cerebellar dysfunction that is seen in developmental disorders such as cerebellar hypoplasia and ASD.

# Abstract: OR02.03 – Targeting the Meningeal Lymphatic Vasculature to Alleviate Symptoms of Autism Spectrum Disorder in a Mouse Model of Fragile X Syndrome

**Published:** 2025-09-05

**Authors:** Gabriel Tavares, Liam O’Reilly, Natalie Frederick, Megan Sullivan, Nathan Jiang, David Shapiro, Haley Brown, Maike Frye, Antoine Louveau

**Affiliations:** a Cleveland Clinic, Cleveland, OH, USA; b UKE Hamburg, Hamburg, Germany

## Abstract

Autism Spectrum Disorder (ASD) is a neurodevelopmental disorder that encompasses several symptoms, including sociability deficits. Neuroglial and synaptic dysfunctions are hallmarks of the pathophysiology of ASD. However, current treatments targeting the above mentioned are not effective, which highlight the needs for investigating alternative pathways for therapeutics. Recent studies show that ASD patients display disrupted cerebrospinal fluid (CSF) homeostasis, a pathway regulated by the meningeal lymphatic vasculature (mLV). We hypothesize that mLV dysfunction may contribute to ASD physiopathology and represent a possible therapeutic target for ASD. Using a mouse model of FXS (Fmr1 KO), which display ASD as a comorbidity, we found abnormal morphology of the mLV in Fmr1 KO mice, accompanied with reduction of drainage of CSF into the cervical lymph nodes. Transcriptomic analysis of in vitro and in vivo LEC deficient for fmr1 demonstrate dysregulation of genes and pathways associated with cytoskeleton organization. We validated these findings by 1) demonstrating increased formation of actin stress fibers in human LECs in vitro after silencing of fmr1, and 2) finding overexpression and mispatterning of tight- (Claudin-5) and adherent- (VE-cadherin) junctions in mLV sprouts of fmr1 KO mice. Specific deletion of fmr1 in LEC reproduces the phenotype of global fmr1 KO demonstrating a new role of fmr1 in lymphatic function. To understand if the LEC dysfunctions caused by the lack of fmr1 contribute to ASD behavioral phenotype, we manipulated the function of the mLV in fmr1 KO mice. The function of the mLV can be improved by local administration of the vascular endothelial growth factor C (VEGFc). Accordingly, injection of AAV-VEGFc improved the mLV morphology and function in fmr1 KO. Furthermore, these mice displayed improved sociability, when compared to mice treated with control virus. Strikingly, we found that the VEGFc treatment rescues the pattern of neuronal activity in brain areas associated with the regulation of social behavior in fmr1 KO mice. Altogether, our data demonstrate that fmr1 intrinsically regulates mLV function and that meningeal lymphatic vasculature is a potential target to improve ASD symptoms. Further analysis will delineate in a deeper and mechanistically fashion, how the improvement of the mLV function leads to normalization of neuronal activity and behavior in Fmr1 KO mice.

# Abstract: OR02.04 – Cerebrospinal Fluid Extracellular Vesicle miRNAs and Synaptic Dysfunction in Alzheimer’s Disease

**Published:** 2025-09-05

**Authors:** Ursula Sandau, Trevor McFarland, Sierra Smith, Douglas Galasko, Joseph Quinn, Julie Saugstad

**Affiliations:** a Department of Anesthesiology and Perioperative Medicine, Oregon Health and Science University, Portland, OR, USA; b Department of Neurosciences, University of California San Diego, La Jolla, CA, USA; c Department of Neurology, Oregon Health and Science University, Portland, OR, USA

## Abstract

Alzheimer’s disease (AD) is the most common form of dementia and is the fifth leading cause of death for individuals aged 65 and older. With only six FDA approved therapies, none of which cure the disease, there is an urgent need to identify new therapeutic targets. Central nervous system cells utilize extracellular vesicles (EVs) to package and secrete miRNAs where they mediate cell-to-cell communication. Importantly, cerebrospinal fluid (CSF) contains EVs derived from the brain whose contents – including miRNAs – reflect the originating cell’s molecular composition. To this end we leveraged our human CSF EV miRNA data, to identify novel genes and canonical pathways that may contribute to AD pathogenesis. Human CSF from living donors with AD (*n* = 28) and neurologically normal controls (*n* = 28) was fractionated by size exclusion chromatography to separate EVs. CSF EVs were characterized by transmission electron microscopy as well as immunoblot for the presence of EV markers (e.g., flotillin) and absence of proteins not associated with EVs, but abundant in CSF (e.g., apolipoproteins [APO]). In AD CSF EVs, we identified four miRNAs (miR-16-5p, -331-3p, -409-3p, and -454-3p) that were significantly increased compared to control CSF EVs. Further, expression levels of miR-16-5 in CSF EVs was found to be sex and genotype dependent, with increases specific to AD females that were also carriers of the APOE4 allele. Relevant to synaptic dysfunction in AD, the predicted targets of miR-16-5p include mRNAs integral to synaptic transmission (SNAP-25, MUNC18-1). In line with this, in postmortem human brain we found that miR-16-5p has a trending increase in AD relative (*n* = 4) with controls (*n* = 3). Also, in the hippocampus of AD females (*n* = 16) there was a significant decrease in both SNAP-25 and MUNC18-1 compared with control females (*n* = 10). While in the hippocampus of AD males (*n* = 16) there were no differences in either SNAP-25 or MUNC18-1 compared with control males (*n* = 8). Together, these data demonstrate that miRNAs altered in AD CSF EVs are informative to changes that occur in the brain and have the potential to identify targets that may serve as novel therapeutics to treat or cure AD.

# Abstract: OR02.05 – Inhibition of p38α MAPK Rescues Behavior and Synaptic Function in a Mouse Model of Mixed Vascular and Amyloid Pathologies

**Published:** 2025-09-05

**Authors:** Hilaree Frazier, David Braun, Caleb Bailey, Chris McLouth, David Powell, Stephen Dundon, Verda Davis, Meggie Coleman, Colin Rogers, Linda Van Eldik

**Affiliations:** a University of Kentucky Sanders-Brown Center on Aging, Lexington, KY, USA; b Department of Neuroscience, University of Kentucky, Lexington, KY, USA; c Department of Biostatistics, University of Kentucky, Lexington, KY, USA; d University of Kentucky MRI and Spectroscopy Center (MRISC), Lexington, KY, USA

## Abstract

Alzheimer’s disease (AD) is hallmarked by amyloid plaques and neurofibrillary tangles, but other pathological changes can also contribute to the severity and progression of this disease. Of these, cerebrovascular dysfunction is arguably the most clinically relevant, with one study reporting that 80% of AD patients had concurrent vascular pathology at the time of autopsy. Despite this, the molecular mechanisms underlying vascular contributions to cognitive impairment and dementia are still unclear, as most studies have been performed in transgenic mouse models with “pure” AD (i.e., only amyloid and/or tau pathology). Recently, p38α MAPK, a regulator of neuroinflammation, has been proposed as a potential target for AD therapies, and this is supported by evidence that p38α inhibition can improve cognitive function and decrease proinflammatory cytokines in amyloidogenic mice. However, inhibition of p38α in the context of mixed vascular and AD pathologies has yet to be thoroughly characterized. We therefore tested if MW150, a small molecule inhibitor highly specific for p38α, could reduce neuroinflammation, vascular and/or amyloid pathology, synaptic dysfunction, and cognitive impairment in a mouse model of comorbid AD (5xFAD) and cerebral small vessel disease (hyperhomocysteinemia [HHcy]). To induce HHcy, 5xFAD mice were transferred to a B-vitamin-deficient diet for 8-weeks. During diet exposure, these mixed dementia (MD) animals also received intraperitoneal injections of either saline vehicle (Veh) or MW150 (0.5 or 2.5 mg/kg). WT animals were maintained on a nutritionally-matched control diet and received injections of saline vehicle only. Endpoints included behavioral assessment, neuroimaging, quantification of cytokines, immunohistochemistry, and electrophysiological measures of synaptic function. Results indicated that MD Veh mice had altered cerebrovascular function, increased proinflammatory cytokines and glial cell activation, impaired synaptic transmission, reduced synaptic protein expression, and worsened behavioral performance compared to WT. No effect of MW150 was detected on cytokine levels, degree of pathology, or glial cell activation. Surprisingly, however, the compound did rescue several measures associated with synaptic dysfunction, including population spike thresholds, LTP maintenance, paired pulse facilitation, synaptic protein expression, and hippocampal synapse numbers. Importantly, MW150 also enhanced performance on a hippocampal-dependent behavioral task (Morris water maze), implying that improvements in hippocampal synaptic function may have mediated these cognitive benefits. Overall, our work suggests that p38α may engage pathways associated with the maintenance of synaptic plasticity, and further support investigations of p38α inhibitors in the clinic.

# Abstract: OR02.06 – The Role of Wnk Kinase in Axon Degeneration

**Published:** 2025-09-05

**Authors:** Adel Avetisyan, M.S. Romina Barria, Ya-Chen Cheng, Marc Freeman

**Affiliations:** a Vollum Institute/Oregon Health and Science University, Portland, OR, USA; b School of Medicine/Department of Biochemistry, Stanford University, Stanford, CA, USA

## Abstract

**Objectives:**

Axon degeneration (AxD) is an active genetic process and a hallmark of many neurodegenerative diseases, metabolic abnormalities, chemotherapy-induced neuropathy, and traumatic brain injury. Despite its prevalence, we know very little about the genetic program that drives AxD. Axonal health is tightly associated with levels of NAD + that are maintained in axons by the activity of the NAD+-producing enzyme Nmnat2. Injury and metabolic insults block Nmnat2 supply to axons and cause activation of a conserved pro-degenerative molecule Sarm1. A growing list of studies shows that the absence of Sarm1 protects axons from degeneration in cases of Nmnat2 depletion, injury and disease models. Interestingly, mutations hyper-activating Sarm1 were also found in patients suffering from ALS and other motor neurons disorders. Despite its key role in neurodegeneration, our understanding on how Sarm1 is activated and whether independent pathways parallel to Sarm1 exist remains scarce.

**Methods:**

To address these questions, we performed an unbiased F1 forward genetic screen, in which we activated dSarm and induced AxD by knocking down dNmnat, Drosophila’s sole orthologue of Nmnat2. A pilot screen of 6,000 chromosomes yielded new molecules mediating AxD, including dWnk (With-no-Lysine) kinase.

**Results:**

Wnk kinases are unique in their ability to sense intracellular concentrations of chloride ([Cl-]i) and potassium ([K+]i). They phosphorylate and activate downstream kinases (e.g., Frayed/STK39) to regulate the activity of cation-chloride cotransporters and hence K + and Cl- influx. Our preliminary data suggest that both dWnk and Frayed promote AxD, and are activated in parallel to dSarm upon dNmnat depletion. We are currently investigating the mechanism by which dWnk activates AxD, and where dWnk resides in the AxD genetic pathways.

**Conclusion:**

Our new screening method efficiently unveiled AxD genes, offering insights into the Sarm1 and AxD pathways. Further research could lead to novel therapeutic strategies.

# Abstract: P01.001 – Profiling Glial Cell Surface Molecules That Enable the Engulfment of Neurons

**Published:** 2025-09-05

**Authors:** Leire Abalde-Atristain, Yunsik Kang, Charles Xu, Amanda Jefferson, Katherine Lehmann, Romina Barria, Taylor Jay, Rachel de la Torre, Dongeun Heo, James A. Frank, Namrata D. Udeshi, Steven A. Carr, Marc R. Freeman

**Affiliations:** a Oregon Health and Science University, Portland, OR, USA; b The Broad Institute of MIT and Harvard, Cambridge, USA

## Abstract

Most organisms first develop an overpopulated and over-wired nervous system, which must be later sculpted and optimized for proper function. This remodeling relies heavily on the work of phagocytes, who recognize and engulf their targets with great temporal and spatial precision. A striking example of the large wave of remodeling occurring during nervous system remodeling is Drosophila metamorphosis, when a large portion of neurons are eliminated or remodeled as the adult nervous system is built. Glial phagocytic activity and neuron-glia signaling is crucial for proper developmental remodeling, both in flies and mammals, and it hinges upon conserved molecules such as the engulfment receptor Draper/MEGF10. Recognizing what needs to be remodeled is believed to rely on a cross-talk between target and phagocyte, but how astrocytes or other subtypes of glia recognize their engulfment targets remains poorly defined. To profile cell-surface molecules that enable astrocyte target recognition and phagocytic activity, we have performed proximity biotinylation proteomics in astrocytes using a plasma membrane-targeted horseradish peroxidase. We have identified 82 proteins enriched on the surface of phagocytic astrocytes, many of which are highly conserved and some of which have previously been characterized as engulfment molecules. We have screened for their functional roles using a variety of molecular-genetic tools in Drosophila, and found that glial knockdown of most of these candidate proteins results in a wide range of defects in neuronal remodeling.

# Abstract: P01.002 – Metabolic Rewiring During Remyelination: Stable Isotope Tracing Reveals Increased De Novo Lipogenesis and Identifies Serological Markers of Brain Metabolism

**Published:** 2025-09-05

**Authors:** Kayla Adkins-Travis

**Affiliations:** a Washington University in St. Louis, St. Louis, USA

## Abstract

Metabolic dysregulation is a key feature of demyelinating disorders, and targeting metabolic processes holds promise as a therapy to stimulate myelin repair. We sought to explore alterations in metabolism associated with demyelination and remyelination using stable isotopes. [13C₆] glucose was administered directly to the brains of mice with cuprizone-induced demyelination and after cuprizone withdrawal, when remyelination is stimulated. Liquid-chromatography/mass spectrometry analysis was performed corpus callosum and serum. We find that in corpus callosum remyelination induces broad rewiring of the citric acid cycle to increase citrate levels and support de novo lipid synthesis. Additionally, we observe distinct labeling in a subset of metabolites in serum, suggesting a CNS origin for these circulating metabolites that can be used to track brain metabolism. Together our results show that broad metabolic rewiring occurs during demyelination and remyelination, and that this can be used to identify serum biomarkers of these processes.

# Abstract: P01.003 – Investigating Lanthionine Ketimine Ethyl Ester’s Role in Remyelination and Neuroprotection in the Cuprizone Model of De-Remyelination

**Published:** 2025-09-05

**Authors:** Intakhar Ahmad, Ankit Tandon, Jacqueline Rocha, Zachary McDonald, Douglas L. Feinstein

**Affiliations:** a Dept. of Anesthesiology, University of Illinois at Chicago (UIC), Chicago, IL, USA; b Jesse Brown VA Medical Center, Chicago, IL, USA

## Abstract

Cuprizone-induced de-remyelination (CPZ) serves as a chemically induced model of reversible oligodendrocyte pathology in animal subjects, exhibiting certain pathological parallels with human Multiple Sclerosis (MS). Post-cessation of CPZ intoxication reveals spontaneous remyelination histologically, however, it does not always correlate with functional recovery. This phenomenon may partially explain why various agents that enhance remyelination in animal studies have not met expected clinical outcomes in human trials. Remyelination also serves as an axonal protection strategy and may enable functional recovery, but the mechanisms of myelin repair, especially after chronic insults remain poorly understood. Lanthionine Ketimine Ethyl Ester (LKE), a synthetic derivative of lanthionine ketimine with improved bioavailability, has shown neuroprotective effects in various animal models of neuropathology. In the current study, our goal is to elucidate the specific neuroprotective mechanisms of LKE in the CPZ model that are distinct from its effects on spontaneous remyelination, potentially leading to enhanced neuroprotection and functional recovery. In the CPZ model, the corpus callosum (CC) is the principal site of demyelination and subsequent remyelination. To identify potential neuroprotective actions, we carried out RNAseq analysis of CC RNA isolated from control mice, mice administered 0.2% CPZ in chow for 6 weeks, and CPZ-treated mice with 2 weeks recovery on control chow or chow containing100 ppm LKE. As expected, CPZ significantly decreased close to 500, and increased close to 900 mRNAs. Recovery on control chow led to restoration of about 400 of those mRNAs. However, compared to CPZ, recovery in the presence of LKE led to significant changes in 1,854 identified mRNA, including upregulation of 554 mRNA that were unaffected by CPZ, and 108 mRNAs that were downregulated by CPZ. Pathway analysis was then carried out utilizing KEGG 2021, an expert-curated knowledge base, and SynGO 2022, a synapse-centric, evidence-based database. KEGG analysis of the set of 108 mRNAs identified pathways including phosphatidylinositol signaling, serotonergic synapse, arachidonic acid metabolism, steroid biosynthesis, inositol phosphate metabolism, retrograde endocannabinoid signaling, Ras signaling pathway and enhanced synaptic signaling. SynGO analysis identified several pathways involved in pre- and post-synaptic membrane structure and function, including regulation of voltage-gated ion channel and membrane potential. In contrast, analysis of the 554 mRNAs were not reduced by CPZ but increased by LKE, identified pathways encompassing circadian entrainment, calcium signaling, axon guidance, cGMP-PKG Signaling, oxytocin signaling, glutamatergic synapse, apelin signaling, actin cytoskeleton regulation, and enhanced synaptic signaling pathways, particularly synaptic vesicle priming and postsynaptic actin cytoskeleton organization, along with elevated calcium level regulation. Together, these data suggest that recovery after CPZ in the presence of LKE not only accelerates processes of remyelination but also increases the expression of mRNAs involved in pre- and post-synaptic functionality. Current studies include carrying out comparative analyses with other notable databases, as well as confirmation of changes in mRNAs using qPCR and immunostaining methods. This work was funded in part by grants from the National MS Society and the Department of Veterans Affairs.

# Abstract: P01.004 – Metabolomic Profiling of Greenhouse-Grown Cultivars of Centella Asiatica, a Herb Touted for Improving Memory and Preventing Cognitive Decline

**Published:** 2025-09-05

**Authors:** Md Nure Alam, Luke Marney, Liping Yang, Jaewoo Choi, Natasha Cerruti, Natascha Techen, Samuel Bassett, James Smith, Kevin Brown, Kadine Cabey, Ramya Viswanathan, Sumanaa Rajagopal, Amala Soumyanath, Jan F. Stevens, Claudia S. Maier

**Affiliations:** a Botanical Dietary Supplements Research Center, Oregon Health and Science University, Portland, OR, USA; b Department of Chemistry, Oregon State University, Corvallis, USA; c Department of Pharmaceutical Sciences, Oregon State University, Corvallis, USA; d Linus Pauling Institute, Corvallis, USA; e School of Chemical, Biological, and Environmental Engineering, Oregon State University, Corvallis, USA; f Oregon’s Wild Harvest, Redmond, USA; g Department of Neurology, Oregon Health and Science University, Portland, OR, USA; h School of Food Science and Nutrition, University of Leeds, Portland, OR, USA; i National Center for Natural Products Research, University of Mississippi, Oxford, USA

## Abstract

**Objective:**

Neurodegenerative disorders are diverse disease conditions resulting from progressive loss of regular neuronal function. These conditions worsen with aging and pose several health problems, such as mild cognitive decline and Alzheimer’s disease. To date, the development of conventional drugs or other formulations (e.g., botanical drugs and dietary supplements) to enhance adaptability to age-related neurological disorders remains a global challenge. The Botanical Dietary Supplements Research Center (BDSRC) on Botanicals Enhancing Neurological and Functional Resilience in Aging (BENFRA) focuses on two medicinal plants, *Withania somnifera* (L.) Dunal (Ashwagandha) and *Centella asiatica* (L.) Urban (Gotu Kola) with scientific evidence for neuroprotective activity. The primary research goal of the BENFRA center is to develop and optimize botanical formulations to promote resilience to neurological disorders and add mechanistic knowledge concerning the bioactive principles associated with enhancing resilience to aging-associated ailments. The identification of plant varieties yielding adequate levels of the active compounds is important for sustainable access to reproducible research materials.

**Method:**

In this study, we investigated four different cultivars of C. asiatica, each grown over three different propagation periods inside a greenhouse, during which aerial parts were collected at four different harvest times: week 8, 10, 12, and 14. Comprehensive metabolomics data were obtained using liquid chromatography high-resolution tandem mass spectrometry (LC-HRMS/MS) from the C. asiatica plant materials extracted with 70% aqueous MeOH.

**Results:**

We employed dimensionality reduction techniques such as principal component analysis (PCA), t-distributed stochastic neighbor embedding (t-SNE), and uniform manifold approximation and projection (UMAP) to examine phytochemical variation among different cultivars and the Bayesian Hierarchical Clustering (BHC) technique to identify changes in the metabolite production rate over time. Presentation of data with Global Natural Product Social (GNPS) molecular networking was used to summarize the chemical variations observed in different cultivars. We focused on 12 specialized metabolites, including mono- and di-caffeoylquinic acids and triterpenoids, that showed neuroactivity in our earlier preclinical research. The aim of this project is to inform best practices for cultivation, growth, and harvest conditions of C. asiatica cultivars and the preparation of standardized formulations for research and clinical testing.

**Conclusion:**

This study shows that the cultivation of C. asiatica in climate-controlled greenhouses is a viable strategy to obtain plant materials for standardized formulations

# Abstract: P01.005 – Downregulation of Pantothenate Kinase and Upregulation of Pantothenate in Liver Kinase B1 Deficient Mouse Astrocytes

**Published:** 2025-09-05

**Authors:** Miss. Rebecca Alemani, Sergey Kalinin, Douglas L. Feinstein

**Affiliations:** a Department of Anesthesiology, University of Illinois Chicago, Chicago, IL, USA; b Jesse Brown VA Medical Center, Chicago, IL, USA

## Abstract

Liver Kinase B1 (LKB1) is a master kinase involved in regulating activation of multiple down-stream kinases, and is associated with cell polarity, metabolism, proliferation, and inflammatory activation. Astrocyte activation and metabolic function have important roles in regulating neuroinflammation and neuropathology, and we previously showed that knockdown of LKB1 from astrocytes exacerbated pathology in the EAE model of MS. To better understand how LKB1 influences astrocyte physiology, we carried out studies using primary mouse astrocytes treated with siRNA to knockdown (KD) LKB1. In brief, primary astrocytes were cultured from mouse pups from postnatal day 0–2. The brains were dissected, trypsinized and plated in DMEM +10% FBS growth medium. The astrocytes were seeded into poly-L-lysine coated coverslips or culture plates and treated with scramble or LKB1 siRNA for 24 h. Untargeted metabolomics showed an increase in Pantothenate (Vitamin B5) in conditioned media from KD cells. Pantothenate is phosphorylated by Pantothenate Kinase (PanK) to generate 4-phosphopantothenate, and mutations in PanK have been shown to cause neurodegeneration. Immunocytochemical analysis for GFAP, PanK2, and LKB1 revealed a decrease of LKB1, as well as GFAP expression in siRNA treated astrocytes compared to control cells. Interestingly, staining for PanK2 appeared to be slightly increased in the KD cells. Similarly, qPCR analysis for GFAP, PanK2, and LKB1 showed altered expression of these mRNAs as compared to control. RNAseq analysis showed that LKB1 KD increased 167 and decreased 179 mRNAs (QValue <0.05 and greater than 1.5-fold change) versus controls. KEGG analysis showed that several metabolic pathways were reduced, including Pantothenate and CoA biosynthesis. These findings indicate that Pank2 gene expression and protein levels are altered in LKB1 KD astrocytes, thereby suggesting a crucial role of the Pantothenate pathway in maintaining metabolic homeostasis in mouse brain astrocytes. Further work is being performed to confirm these preliminary findings.

# Abstract: P01.006 – Timecourse of Activity-Dependent Endogenous Opioid Peptide Gene Transcription After Seizures in the Mouse Hippocampus

**Published:** 2025-09-05

**Authors:** Ashley Anderson, Natasha Warikoo, Kunal Gupta, William Hendricks, Eric Schnell

**Affiliations:** a Oregon Health and Science University, Portland, OR, USA; b Vollum Institute, Portland, OR, USA; c Portland VA Medical Center, Portland, OR, USA

## Abstract

Endogenous opioid peptides (EOs) may modulate excitability within the hippocampal circuit, particularly in the context of temporal lobe epilepsy. EOs, such as enkephalin and dynorphin, demonstrate notable changes in localization and staining during epileptogenesis, but little is known about the roles of EOs in hippocampal circuitry in both healthy and epileptic brains. Here, we examine changes in expression of the endogenous opioid genes Prodynorphin (PDyn) and Proenkephalin (PEnk) in hippocampal tissue from wildtype and genetically modified mouse models using immunohistochemistry, quantitative RT-PCR, and in situ hybridization. First, we quantitatively characterized two PEnk-IRES-Cre::Rosa26 reporter mouse lines, and found that the density of PEnk-reported neurons increases with mouse age. We also stained tissue for cFos and PEnk after pentylenetetrazole (PTZ)-induced seizures using immunohistochemistry and found significant increases in cFos staining at early time points after seizure, and significant increases in EO staining at later stages of epileptogenesis. To improve enkephalin detection, we used Real-Time PCR and in situ hybridization to characterize hippocampal mRNA changes at different timepoints after PTZ-induced seizures or during kainic acid-induced epileptogenesis. Preliminary RT-PCR data show a 60-fold increase in cFos mRNA transcription peaking 15–30 min after PTZ-induced seizure and a more delayed increase in Penk transcripts across different mouse seizure models and at early stages after translational epilepsy models. The effects of seizures on Prodynorphin gene expression at early timepoints after seizures was analyzed and suggested a different pattern of regulation. Together, these data suggest activity-dependent endogenous opioid peptide gene expression may be a component of post-seizure functional circuit reorganization, with potential implications for epileptogenesis and the control of brain hyperexcitability.

# Abstract: P01.007 – Targeted Overexpression of Cyclooxygenase-2 in the CA3 Region of the Hippocampus Is Sufficient to Suppress Global Hyperexcitation in the Brain

**Published:** 2025-09-05

**Authors:** Antoaneta Andonova, Sandra Hewett, James Hewett

**Affiliations:** a Syracuse University, Syracuse, NY, USA

## Abstract

Cyclooxygenase-2 (COX-2) is one of two isozymes that produce prostaglandins from arachidonic acid. It is expressed by certain glutamatergic neurons of the normal brain where its activity and expression are coupled to excitatory activity. Compelling evidence suggests that COX-2 plays an important homeostatic role in the brain by suppressing hyperexcitation. Where it performs this function, however, is currently unknown. The highest level of constitutive COX-2 expression in the normal brain is found in pyramidal neurons of the CA3 region of the hippocampus, where it is >3-fold higher than neurons in layer 2/3 of the cerebral cortex, another glutamatergic population that expresses COX-2 constitutively. To determine if COX-2 in CA3 pyramidal neurons could contribute to suppression of brain hyperexcitation, a transgenic mouse line harboring a Cre-inducible COX-2 Over-Expression (COE) cassette was crossed with a Grik4-Cre transgenic mouse line that targets Cre recombination in CA3 pyramidal neurons. In the absence of Cre, the COE construct drives expression from an EGFP transgene. Cre-dependent excision of this transgene activates expression from a downstream COX-2 transgene. It was reasoned that Cre-induced COX-2 overexpression in the CA3 would further suppress hyperexcitation. One advantage of the COE transgene is that it permits the assessment of Cre-dependent recombination in vivo via the loss of EGFP epifluorescence in the target cells. Double transgenic offspring from this cross (COE+) had four-fold less EGFP epifluorescence in the CA3 region relative to single transgenic littermates that lacked Cre (COE-). As anticipated, EGFP expression in the dentate gyrus and CA1 regions was not different from COE- control mice. Thus, this approach exhibited high specificity and efficacy. Importantly, the recombination led to a > 3-fold increase in COX-2 expression in the CA3 of COE + mice relative to COE- controls. COE- and COE + mice were next treated with the convulsant GABAA receptor antagonist, pentylenetetrazole, to induce brain-wide hyperexcitation. Hyperexcitation was measured by the incidence of convulsive responses in cohorts of each genotype. Whereas the COE- cohort had a convulsive incidence of 69%, COE + mice had an incidence of 28%. There was no sex difference in either genotype. These results shown that targeted overexpression of COX-2 in the CA3 is sufficient to suppress global hyperexcitation in the brain. To test whether endogenous hippocampal COX-2 similarly suppresses hyperexcitation, hippocampal neuron cultures were treated with bicuculline to activate excitatory neurotransmission. Hyperexcitation was assessed 60 min after bicuculline by expression of cFos, an immediate early gene that is known to be rapidly induced by glutamatergic activity. The function of COX-2 activity was assessed using the selective inhibitor, NS398. If endogenous COX-2 functions to suppress hyperexcitation, it was posited that NS398 would potentiate bicuculline-induced cFos expression. In the absence of NS398, bicuculline induced >3-fold increase in cFos expression in the cultures. By comparison, treatment with both bicuculline and NS398 increased cFos expression by >7-fold. Together, these results support the conclusion that COX-2 in the hippocampus, likely in the CA3 region, suppresses brain hyperexcitation. Because hyperexcitation is detrimental to brain function, these results suggest that CA3 COX-2 is a brain protective pathway.

# Abstract: P01.008 – Chemogenetic Manipulation of Astrocytic Calcium Signaling and Neuroinflammation in Demyelinating Disease

**Published:** 2025-09-05

**Authors:** Christina Angeliu, Karissa Garbarini, Veronica Cheli, Pablo Paez

**Affiliations:** a University at Buffalo, Buffalo, NY, USA

## Abstract

In the most common demyelinating disease, Multiple Sclerosis (MS), demyelinated lesions and profound astrogliosis develop in the central nervous system (CNS). While this astrogliosis can be beneficial in some contexts, it can be detrimental in others. Changes in intracellular ion concentration via ion channels influences astrocyte reactivity and recent work shows that astrocyte reactivity can be stimulated by the Gq-GPCR hM3Dq and attenuated by the Gi-GPCR hM4Di. Therefore we hypothesize that in demyelinating disease, activation of astrocytic hM3Dq will promote reactivity, thus worsening neuroinflammation and demyelination, while activation of astrocytic hM4Di will have opposing effects. To first characterize how hM4Di activation affects astrocytic calcium signaling, calcium imaging was done in primary cortical astrocytes expressing hM4Di. Compared to vehicle-treated cells, astrocytes pretreated with the activator of hM4Di, clozapine-N-oxide (CNO), showed suppressed intracellular Ca^2+^ increases in response to ATP, potassium, glutamate, and acetylcholine. In the absence of extracellular Ca^2+^, these responses were significantly reduced and differences between vehicle and CNO-treated cells were abolished. Taken together, these data indicate that hM4Di stimulation in astrocytes is inhibitory via suppression of Ca^2+^-permeable ion channel activity. Through immunocytochemistry, we unexpectedly found that CNO treatment increased cell density and percentage of Ki67+ cells but did not affect GFAP expression. These data indicate that in the absence of other stimuli, activation of hM4Di in astrocytes does not affect reactivity, but does stimulate proliferation. To evaluate the consequences of altered calcium signaling within demyelinating disease, we conducted experiments in the cuprizone and experimental autoimmune encephalomyelitis (EAE) models using mice that express hM4Di in astrocytes. In a preliminary study, activation of hM4Di during the acute phase reduced clinical severity and motor impairment. We found that neuroinflammation and immune cell infiltration was reduced in the brain along with reduced astrogliosis and demyelination in the spinal cord. In the cuprizone model, when hM4Di was activated at the peak of astrogliosis and demyelination, we found suppressed demyelination. We also found an increase in GFAP + cells, which is consistent with increased proliferation seen in vitro. We have demonstrated that hM4Di activation alters calcium signaling as anticipated and found previously unreported effects on astrocyte proliferation. We have begun to characterize the impact of these effects in vivo during demyelination. Presumably through suppression of calcium signaling, reduction of reactivity, and stimulation of proliferation, hM4Di activation in astrocytes seems to attenuate the severity of demyelinating disease.

# Abstract: P01.009 – The Guidance Cue Receptor, PlexinA1, Plays Differential Roles in the Development of the Mouse Embryonic Spinal Commissural Neurons

**Published:** 2025-09-05

**Authors:** Adefemi Baderinwa, Victor Danelon, Ishaq Ansari, Tracy Tran

**Affiliations:** a Rutgers University, Newark, NJ, USA; b Caldwell University, Caldwell, NJ, USA

## Abstract

The establishment of neural circuits requires a series of developmental events including neuronal migration, cell body positioning, and axon guidance. In the vertebrate spinal cord, commissural neurons (CNs) are important for conveying and coordinating sensory information between the right and left sides of the central nervous system. Additionally, their stereotypical axonal trajectory of initially projecting dorsoventrally (pre-crossing segment), crossing in the ventral midline at the floor plate, and turning orthogonally to project in the anterior-posterior axis (post-crossing segment), make them an ideal model system for studying axon guidance. Previously, we have shown that the Semaphorin receptor, PlexinA1 (PlxnA1) which is expressed in spinal CNs, is involved in axon guidance events, however, it remains unknown how distinct subsets of spinal CNs respond to PlxnA1-mediated axon guidance. In this study, we discovered a novel role for PlxnA1 in regulating the final cell body position of a subset of spinal CNs that expresses the transcription factor Neurogenin1, which belongs to the dorsal interneuron 2 (dI2) population. We used the Neurogenin1:tau-mCherry reporter mouse line that genetically labels the subset of the spinal commissural dI2 to assess the role(s) of PlxnA1 in a loss-of-function approach by examining the cell body positioning, survival, and ventral midline axonal pathfinding in the PlxnA1-/- knockout embryos at E10.5 and E11.5. We observed significantly reduced dI2 mCherry + neurons in the mediolateral axis of the dorsal spinal cord of the PlxnA1-/- embryos compared to wild type (WT) littermate controls, suggesting impairment in the migration and/or generation of dI2 neurons. Next, we showed a significant increase in number of dI2 mCherry + apoptotic neurons mostly in the dorsomedial region in the PlxnA1-/- embryos compared to WT, suggesting that increased apoptosis is a likely cause for the reduction observed in dI2 mCherry + neurons. Finally, we observed significantly fewer axons reaching the ventral midline in the PlxnA1-/- embryos compared to WT, which suggests that the observed axon guidance defect might be a consequence of reduction in dI2 mCherry + neurons. Taken together, our results provide novel insights to the diverse roles of PlxnA1 in mediating distinct cellular processes in spinal CN development, and our findings may contribute to future studies to develop better therapeutic strategies following spinal cord injury or disease. This work is supported by grants from the National Science Foundation IOS (1556968, 2034864); the New Jersey Commission on Spinal Cord Research (CSCR16IIRG013); the National Science Foundation Equity for Excellence in STEM (1905142); the National Institute of Health Graduate Research Training Initiative for Student Enhancement (5T32GM140951-02).

# Abstract: P01.010 – Niemann Pick Disease Type C1 Affects the Concentration and Cargo of Extracellular Vesicles in Patient Samples

**Published:** 2025-09-05

**Authors:** Sarah Catherine Baker, Ursula Sandau, Trevor McFarland, Julie Saugstad

**Affiliations:** a Oregon Health and Science University, Portland, OR, USA

## Abstract

**Introduction:**

Niemann Pick Disease Type C1 (NPC1), also known as Childhood Alzheimer’s, is a rare neurodegenerative disease caused by mutations in the cholesterol transport protein NPC1. These mutations disrupt endolysosomal processing by causing cholesterol to accumulate in endosomes and lysosomes. Endolysosomal disruptions are also known to affect the biogenesis of extracellular vesicles (EVs), which can be formed through the ESCRT-dependent pathway in late endosomes. EVs have been implicated in numerous neurodegenerative diseases and are under investigation as potential biomarkers. However, little is known about how EVs are affected by NPC1. To address this, we compared the size, concentration, and molecular cargo of EVs from control and NPC1 patient samples, and investigated changes to EV biogenesis pathways in NPC1 cells.

**Methods:**

We analyzed EVs from cerebrospinal fluid (CSF, *n* = 10) and dermal fibroblasts (*n* = 8) of NPC1 patients and age-matched controls. EV separation was performed via size exclusion chromatography and ultrafiltration, and validated by immunoblots and fluorescent nanoparticle tracking analysis (fNTA). EV size, concentration, and cargo was analyzed using transmission electron microscopy (TEM), microfluidic resistive pulse sensing (MRPS), vesicle flow cytometry (VFC), multiplexed EV immunoassays (MEVI), and microRNA qPCR arrays. Changes to EV biogenesis pathways were investigated using quantitative immunoblots.

**Results:**

Immunoblots and fNTA showed successful separation of particles between 100 and 200 nm positive for EV markers from both CSF and fibroblast conditioned media. Quantitative immunoblots showed a significant increase in EV markers Flotillin 1 (*p* = 0.04) and CD81 (*p* = 0.01) in NPC1 CSF compared to controls, while MRPS and TEM suggest no change in CSF EV size. fNTA showed that NPC1 conditioned media contains more particles of 100–150 nm than controls (*p* = 0.004) with no change in particle size, in agreement with preliminary VFC results. MEVI showed altered surface markers on NPC1 EVs compared to controls, including enrichment for proteins associated with immune activation in CSF EVs and cell stress in conditioned media EVs. qPCR arrays identified 8 miRNAs in NPC1 CSF EVs not found in controls, which by pathway analysis are enriched in hereditary and developmental disorders and neurological disease. Finally, quantitative immunoblots suggest that proteins involved in the ESCRT-dependent EV biogenesis pathway are downregulated in NPC1 cells (*p* = 0.03).

**Conclusions:**

Our results indicate that NPC1 patient CSF and fibroblast conditioned media contain higher EV concentrations relative to controls, which may be due to alterations in EV biogenesis pathways, and that NPC1 EVs carry altered surface proteins and miRNA cargo. Current work is investigating whether these changes are due to alterations in EV biogenesis pathways, and the potential for altered EV cargo to serve as NPC1 biomarkers. Ultimately this work demonstrates how EVs are altered in NPC1, and may offer new insights into the underlying mechanisms of this devastating neurodegenerative disease.

# Abstract: P01.011 – Neuronal Subtype Vulnerability to Microglial-Induced Synapse Loss in Neuroinflammation

**Published:** 2025-09-05

**Authors:** Rebecca Beiter, Chun-Wei Chen, Ruixuan Xiao, Dorothy Schafer

**Affiliations:** a University of Massachusetts Chan Medical School, Worcester, MA, USA

## Abstract

Microglia, the professional phagocytes of the brain, are known to play an important role in shaping neuronal circuitry by engulfing synapses during development. Microglia have also been shown to engulf synapses in multiple neurodegenerative conditions, including Alzheimer’s Disease, Huntington’s disease, and multiple sclerosis (MS). However, unlike development, this engulfment of synapses during disease has been shown to result in both cognitive and physical impairments. Recently, work using experimental autoimmune encephalomyelitis (EAE), an animal model of MS, demonstrated that, in the context of neurodegeneration, microglia engulf synapses from retinal ganglion cells (RGCs) that project into the thalamus, resulting in visual impairment. This engulfment is driven by deposition of the complement component C3 on synapses. However, it remains unclear why some synapses are tagged for removal while others are spared. In this work, we have utilized the known molecular heterogeneity of RGCs to determine whether specific subtypes of neurons are more vulnerable to neuroinflammation-related synapse loss than others. We have demonstrated that microglia preferentially engulf synapses from αRGCs, a subtype of RGC characterized by a large cell body, compared to other subtypes of RGCs. We are currently using this data to identify neuron-specific transcriptional programs that may increase vulnerability to synapse loss. This data will allow us to further dissect the mechanisms driving synapse loss in neuroinflammation and will provide potential therapeutic targets for slowing detrimental synapse loss.

# Abstract: P01.012 – TTYH1 Regulates Autophagy in Astrocytes via Endolysosomal Processing of Neuron-Derived Signaling Lipids

**Published:** 2025-09-05

**Authors:** Jagannatham Naidu Bhupana, Angelid Pabon, Ching-On Wong

**Affiliations:** a Department of Biological Sciences, Rutgers University, Newark, NJ, USA

## Abstract

Astrocytes are responsible for coordinating lipid homeostasis in the central nervous system. Whereas internalized lipids are processed in endolysosomes, the mechanistic basis of how endolysosomes mediate the astrocytic response to neuron-derived lipids remains largely undefined. In this study, we uncover an endolysosomal transmembrane protein that is required for processing signaling lipids released by neurons. The understudied transmembrane protein TTYH1 exhibits astrocyte-specific expression. We found that endogenous TTYH1 is localized to endolysosomes and autolysosomes in astrocytes. In primary astrocytes derived from human and conditional knockout mouse, we found that TTYH1 is required for maintaining autophagic flux. Concomitantly, TTYH1-deficient astrocytes exhibit lipid droplet accumulation, indicating impaired lipophagy. Protein structural analyses revealed that TTYH1 might mediate luminal transport of specific sphingolipids at the endolysosomal membrane. Interestingly, autophagic flux was impeded when astrocytes were treated with these sphingolipids. Importantly, we found that TTYH1 is both necessary and sufficient to mitigate such autophagic impairment by the sphingolipids. Furthermore, we identified the amino acid residues critical for TTYH1 function. Expanding our inquiry to TTYH1’s relevance in neural lipid homeostasis, we observed that interleukin-1β or hyperexcitability stimulates neurons to secrete lipids that impede autophagic flux and lipid droplet degradation in astrocytes. The impediment was exacerbated by TTYH1 deficiency and was reversed by inhibiting neuronal production of specific sphingolipid or overexpression of TTYH1 in astrocytes. Thus, our results demonstrate that astrocytes fine-tune autophagic activity in response to signaling lipids released by neurons, and that TTYH1 mediates the endolysosomal processing of such signaling lipids.

# Abstract: P01.013 – Targeting the Pathogenic Synergy of Ceramide and S1P in Alzheimer’s Disease

**Published:** 2025-09-05

**Authors:** Zhihui Zhu, Xiaojia Ren, Zainuddin Quadri, Priyanka Tripathi, Liping Zhang, Ahmed Elsherbini, Simone Crivelli, Stefka Spassieva, Erhard Bieberich

**Affiliations:** a University of Kentucky, Lexington, KY, USA; b VA Medical Center, Lexington, KY, USA

## Abstract

The classical ceramide-sphingosine-1-phosphate (S1P) rheostat, which predicts opposite roles for pro-apoptotic ceramide and pro-survival S1P in cancer cells, takes on a distinct function in neurodegenerative disease. Research in our laboratory shows that ceramide and S1P mutually amplify their pathogenic function in Alzheimer’s disease (AD). We found that Aβ binds to ceramide-rich extracellular vesicles (CREVs) that are secreted by astrocytes. The Aβ-associated CREVs are taken up by neurons and transported to mitochondria, ultimately leading to mitochondrial damage and neuronal cell death. We also found that Aβ induces the interaction of microglial S1P receptor 1 (S1PR1) with toll like receptor 4 (TLR4), which leads to the activation of microglia that secrete neuroinflammatory cytokines and fail to clear Aβ. In turn, S1P induced suppression of Aβ clearance in microglia and astrocytes and ceramide-mediated Aβ mitotoxicity in neurons synergistically drive AD pathogenesis. We tested several pharmacological approaches to disrupt this pathogenic synergy of ceramide and S1P and mitigate AD pathology. Using the 5XFAD familial AD mouse model, we inhibited Aβ-induced CREV secretion by astrocytes with the neutral sphingomyelinase 2 inhibitor GW4869 and restored Aβ clearance by microglia with the S1PR1 antagonist Ponesimod. These interventions significantly improved AD pathology and memory function in 5XFAD mice. Currently, we are testing additional drugs to target the synergy of ceramide and S1P, aiming to develop new approaches for AD therapy. This work is supported by NIH grants R01AG064234, 1RF1AG078338, and R21AG078601, and VA grant I01 BX003643.

# Abstract: P01.014 – The Effect of Astrocyte Syncytium on Neuronal Excitability and Neurovascular Coupling in the Mouse Cortex

**Published:** 2025-09-05

**Authors:** Danica Bojovic, Steve Sullivan, Andre Luiz Andreotti Dagostin, Anusha Mishra, Henrique von Gersdorff

**Affiliations:** a Vollum Institute, Oregon Health and Science University, Portland, OR, USA; b Department of Neurology Jungers Center for Neuroscience Research, Oregon Health and Science University, Portland, OR, USA; c Department of Anesthesiology and Perioperative Medicine, Oregon Health and Science Univesity, Portland, OR, USA; d Jungers Center for Neurosciences Research, Portland, OR, USA

## Abstract

Astrocytes are connected in a functional syncytium via gap junctions composed of connexin 43 (Cx43) and connexin 30 (Cx30). The specific roles of the astrocyte syncytium in regulating neuronal excitability and neurovascular coupling are still largely unknown. Astrocyte syncytium maintains the K + homeostasis by taking up and buffering K + released from active neurons. Closing of gap junctions decreases the syncytial connectivity and is expected to impair K + buffering, leading to K + accumulation in the extracellular space and impairment of neuronal firing. Furthermore, the functional role of astrocyte syncytium in regulating neurovascular coupling, which is also modulated by extracellular K+, has not been extensively studied. To examine the syncytial function of astrocytes, we used a Cx30-/- knockout crossed with a Cx43fl/fl mouse in which in which Cre was expressed under GfapABC1D promoter to knockdown Cx43 (Cx30KO/Cx43KD) and express a TdTomato reporter. Immunofluorescence was used to evaluate the reduction of Cx30 and Cx43 staining in the cortical astrocytes. The virus knockdown infected <50% of all astrocytes. Cx30KO/Cx43KD mice had reduced gap junction staining in astrocytes compared to littermate controls. We then measured evoked field post-synaptic currents in cortical slices from Cx30KO/Cx43KD mice in the presence carbenoxolone (100 µM CBX), a Cx43 blocker, due to incomplete knockdown of Cx43. In the absence of astrocyte gap junctions, neuronal fiber volley and postsynaptic currents remained unchanged in overall amplitude and timing when compared to littermate controls (Cx30+/-; Cx43) and wild types (C57 strain). These results indicate that Cx43 and Cx30 may not contribute significantly to neuronal excitability at a gross level, but further experiments are necessary to confirm this result. Furthermore, we measured arteriole responses evoked by neuronal stimulation in Cx30-/- mice in the presence of CBX to close astrocyte gap junctions. In wildtype brain slices, neuronal stimulation evoked arteriole dilation, while in Cx30-/- slices treated with CBX, it evoked arteriole constriction. Thus, disconnecting the astrocyte syncytium altered arteriole neurovascular responses in the mouse cortical slices. In conclusion, the syncytial decoupling may have a stronger impact on neurovascular coupling than perhaps on the neuronal excitability. Further studies will explore how syncytial connectivity affects membrane properties of astrocytes and neurons, investigating a mechanism that ties together astrocyte syncytial function in regulating neuronal excitability and neurovascular responses.

# Abstract: P01.015 – Matrix Metalloproteinase-1 and Ninjurin a Govern Glial Responses to Neurodegeneration

**Published:** 2025-09-05

**Authors:** Cole Brashaw, Arpita Ray, Maria Purice, Annie Griffin, Sean Speese, Mary Logan

**Affiliations:** a Oregon Health and Science University, Portland, OR, USA

## Abstract

Glial cells are a critical component of a properly functioning nervous system. When infection, acute injury, or neurodegeneration occur, glial cells become reactive and infiltrate sites of damage to clear apoptotic cells and fragmenting projections through phagocytic engulfment. While some factors involved in the glial immune response to axon degeneration have been identified, many remain unknown or poorly understood. Using well-established models of axotomy in the olfactory system and ventral nerve cord (“spinal cord”) of adult Drosophila melanogaster, our lab has recently characterized the role of Matrix Metalloproteinase-1 (Mmp1) in glial phagocytic clearance of severed axons. Matrix metalloproteinases (MMPs) are a large, conserved family of secreted and membrane-tethered extracellular proteases that are broadly implicated in extracellular membrane (ECM) degradation, as well as targeted cleavage of factors that alter cellular signaling events. Drosophila Mmp1 is transcriptionally upregulated in glial cells after axotomy and is required for timely glial invasion of injury sites and phagocytic clearance of degenerating neuronal material. Notably, MMPs target a wide variety of proteins for cleavage, but key proteins targeted by Mmp1 as glia respond to axon degeneration are still largely unknown. The transmembrane protein nerve-injury induced protein NinjurinA (NijA) is a known Mmp1 cleavage target in S2 cells in the context of cell adhesion. In mammals, Ninjurins are upregulated in injury, stress, and inflammation models, but the specific functions of these proteins are still unclear. Our preliminary data suggests that NijA is required in both glia and antennal lobe neurons for proper glial recruitment and phagocytic engulfment after antennal axotomy. I hypothesize that NijA is cleaved by glial secreted Mmp1 after axotomy to facilitate glial recruitment and phagocytic engulfment. Applying genetic and molecular techniques, I aim to define the role of NijA during glial responses to degenerating axons and determine how Mmp1/NijA interactions specifically drive ECM remodeling, glia migration, and glial phagocytic function.

# Abstract: P01.016 – Maintenance of Engrafted Human OPC Fate via Lentiviral Noggin Expression in a Rabbit Model of Chronic Demyelination

**Published:** 2025-09-05

**Authors:** Gregory Buck, James Cooper, Jonothon Frost, Fraser Sim

**Affiliations:** a University at Buffalo, Buffalo, NY, USA

## Abstract

Multiple Sclerosis (MS) is an autoimmune disease that results in the demyelination of neurons in the central nervous system (CNS). Within chronic MS lesions, there is a decreased pool of oligodendrocyte progenitor cells (OPCs) and insufficient differentiation of OPCs into myelinating oligodendrocytes (OLs). The transplantation of human OPCs (hOPCs) has been proposed as a promising therapeutic strategy for human demyelinating disease and has been shown to restore myelination in a genetic mouse model of congenital hypomyelination. However, the highly inflammatory environment in MS lesions may prevent transplanted cells from undergoing oligodendrocyte differentiation. Indeed, our preliminary results in a rabbit model of chronic demyelination demonstrated that the vast majority of transplanted hOPCs differentiated into astrocytes within the lesion. As BMP signaling is highly active in demyelinated lesions, we hypothesized that BMP signaling acts to induce OPC quiescence and to divert OPCs to astrocyte fate following transplantation. In this study, we developed a Noggin-expressing lentivirus to block BMP signaling that co-expressed H2B-mCherry (LV-Nog-P2A-mCherry) to label infected cells. LV-Nog-P2A-mCherry significantly blocked BMP response element-driven luciferase in CG4 cells compared to LV-mCherry (*n* = 5). Importantly, LV-Nog-P2A-mCherry also reduced BMP-induced astrocyte differentiation from primary hOPCs in vitro (*n* = 4 human samples). Six rabbits were transplanted with 5 x 104 hOPCs following induction of demyelination. hOPCs were infected with either LV-Nog-P2A-mCherry or LV-mCherry prior to transplant. Rabbits were immunosuppressed beginning 1 day prior to transplantation. At 12 weeks post-transplant, animals were euthanized, and tissue was prepared for histological analysis. The majority of human cells were localized within the demyelinated lesion. However, a significant fraction of migratory hOPCs were observed in normal brain parenchyma. Noggin-expressing hOPCs exhibited significantly increased proliferation relative to both control mCherry and co-transplanted uninfected cells. Noggin increased the proportion of Ki67-labeled hOPCs located both in the lesion and migratory hOPCs located outside the lesion. These preliminary observations suggest that noggin expression might be sufficient to alter the behavior of transplanted hOPCs and could be a viable approach to overcome the inhibitory consequences BMP signaling in demyelination.

# Abstract: P01.017 – Lack of Functional Hippocampal Mossy Cell Inputs Accelerates Adult-Born Dentate Granule Cell Maturation

**Published:** 2025-09-05

**Authors:** Corwin Butler, Alyssa Danis, Arielle Isakharov, Daniel Kim, Gary Westbrook, Eric Schnell

**Affiliations:** a Oregon Health and Science University, Portland, OR, USA; b Portland VAMC, Portland, OR, USA; c Vollum Institute, Portland, OR, USA

## Abstract

Early survival and circuit integration of adult-born hippocampal dentate granule cells (abDGCs) requires glutamatergic innervation, with hilar mossy cells forming the first functional glutamatergic synapses onto abDGCs. Disruption of hippocampal neurogenesis occurs after brain injury, coincident with loss of the hilar mossy cells. We hypothesized that dysfunction of mossy cells following brain injury contributes to aberrant neurogenesis and integration of abDGCs. We selectively ablated or silenced hilar mossy cells using viral vectors (AAV5-flex-taCasp3, AAV5-flex-TeLC-GFP) in Crlr-Cre (Calcitonin receptor-like receptor) mice. Virus-mediated apoptosis of hilar mossy cells by taCasp3 resulted in extensive (>70%) loss of mossy cell bodies and axonal projections in the inner molecular layer (IML). In contrast, hilar mossy cell bodies and axons were maintained following virus-mediated silencing by TeLC, but axons became beaded in the IML, consistent with block of vesicle release. After mossy cell ablation or silencing, we labeled abDGCs using BrdU or retroviral vectors, and used immunohistochemistry and electrophysiological assays to examine the proliferation, survival, and maturation of abDGCs. Mossy cell loss did not impact proliferation, survival, or later morphology of adult-born DGCs, but dendritic outgrowth of abDGCs was accelerated two weeks post-mitosis. Interestingly, despite the loss of mossy cell axons in the IML, dendritic spine density on proximal abDGC dendrites was unaffected. Immunohistochemical staining and electrophysiological assays revealed structural and functional reorganization of the molecular layer after mossy cell ablation, such that middle molecular layer inputs now innervate more proximal abDGC dendrites, potentially compensating for the lack of mossy cell inputs. In contrast, preliminary studies of TeLC silencing of mossy cells revealed modifications in immature DGC density and migration of these cells away from the subgranular zone, without changing the laminar structure of molecular layer inputs. This suggests that functional synaptic innervation at proximal dendritic sites might critically regulate adult neurogenesis, demonstrating the importance of early functional synaptic innervation in the regulation of abDGCs.

# Abstract: P01.018 – Punishment-Associated Compulsive Methamphetamine Intake and Abstinence Are Associated With Differential Hydroxymethylation of miRNAs in the Nucleus Accumbens of Rats

**Published:** 2025-09-05

**Authors:** Jean Lud Cadet, Ceiveon Munoz, Atul Daiwile

**Affiliations:** a Molecular Neuropsychiatry Research Branch, NIDA, Baltimore, MD, USA

## Abstract

Methamphetamine (METH) use disorder (MUD) is a neuropsychiatric disorder characterized by compulsive and continued use despite adverse life consequences. Fifty percent of METH users develop MUD, and these individuals often experience impairments in learning and memory functions secondary to METH-induced hippocampal dysfunctions. MUD is thought to be due to epigenetic and transcriptional changes that occur in various brain regions after repeated exposure to the drug. A better understanding of these molecular alterations may help to develop therapeutic approaches to psychiatric patients with MUD. The present preclinical study was conducted to identify potential differences between rats that compulsively took METH from those that suppress their intake of the drug in the presence of footshocks used to mimic the ‘adverse consequences’ criteria of the psychiatric Diagnostic Statistical Manual (DSM). We thus used a rat model of METH self-administration (SA) accompanied by contingent foot-shock punishment after the animals had learned to give themselves METH. In the presence of contingent footshocks, some rats suppressed their intake (shock-sensitive) whereas other rats kept on taking the drug (shock-resistant). Rats were euthanized 2 h after the last METH SA plus foot-shock session. The nucleus accumbens was immediately removed, frozen, and used later for genome-wide hydroxymethylated DNA immunoprecipitation (hMeDIP) sequencing. All METH-trained rats significantly escalated their intake of the drug per session during the first 16 days and then maintained their intake for the remainder of the training session. The application of footshocks led to the separation of rats into rats that continued to press the lever for METH (shock-resistant, SR) and those that almost completely stopped taking METH (shock-sensitive, SS). We found significant differentially hydroxymethylated peaks in the SRvCT, SSvCT, and SRvSS comparisons. Notably, DNA sequences near several microRNAs (MiRs) showed differentially hydroxymethylated peaks in these comparisons. These include Mir17-1, Mir551b, and Mir708 that showed increased hydroxymethylation in the SRvCT comparison. In addition, sequences near Mir124-2, Mir153, Mir181b1, Mir 206, and Mir708 showed increased hydroxymethylation whereas Mir6322 showed decreased hydroxymethylation in the SSvCT comparison. In the SRvSS comparison, Mir124-1, Mir 145, Mir146a, Mir 3099, and Mir 3596a showed increases whereas those near Mir29b1 and Mir185 showed decreased DNA hydroxymethylated peaks. The present results implicate altered DNA hydromethylation of MiRs is involved in the behavioral manifestations of continuous compulsive METH taking and abstinence in the presence of adverse consequences. Our results suggest that different non-coding small RNAs known to participate in post-transcriptional regulation and modulation of synaptic plasticity might play important roles in compulsive METH taking and abstinence in this model of methamphetamine use disorder.

# Abstract: P01.019 – Modified Excitability and Locomotor Behavior in Mice With Conditional Deletion of the Anion Channel Subunit LRRC8A in Interneurons

**Published:** 2025-09-05

**Authors:** Madison Chandler, Ariella Sprague, Julia Nalwalk, Emily Groom, Kristen Zuloaga, Annalisa Scimemi, Alex Mongin

**Affiliations:** a Albany Medical College, Albany, NY, USA; b University at Albany, Albany, NY, USA

## Abstract

Volume-regulated anion channels (VRACs) are chloride channels, ubiquitously expressed in all brain cells. VRACs are comprised of proteins from the leucine-reach repeat-containing family 8 (LRRC8) and are known for their critical role in cell volume regulation. Yet, recent studies indicate that VRACs may be involved in many other cellular processes, including regulation of brain excitability. We have recently reported that in mice, the brain-wide Nestin-Cre-driven deletion of the indispensable VRAC subunit LRRC8A (bLRRC8A KO) causes spontaneous seizures and 100% mortality during adolescence and early adulthood. bLRRC8A KO mice exhibit modified GABAergic signaling and lose up to 20% of parvalbumin-positive interneurons. In the present work we tested the cell type-autonomous role of VRACs/LRRC8A in interneurons. We produced GABAergic interneuron-specific LRRC8A deletion (iLRRC8A KO) by breeding Lrrc8afl/fl and Lrrc8afl/+;Gad2-IRES-Cre+/− mice. This strategy generated iLRRC8A KOs and the Lrrc8afl/+ littermate controls. Specificity of Cre targeting was validated by inbreeding the Ai19 tdTomato Cre reporter. Functional outcomes were evaluated in 12-week old mice using a battery of behavioral assays including the open-field, elevated plus maze, Y-maze, novel object recognition, Rotarod, and DigiGait tests. In our hands, the Gad2-promoter-driven Cre recombination labeled major interneuron populations in the olfactory bulbs, basal ganglia, cortex, and cerebellum. Interestingly, outside of the cerebellum, we found limited tdTomato expression in parvalbumin + cells. In striking contrast to the lethal seizure phenotype of the brain-wide LRRC8A deletion, iLRRC8A KO mice were fully viable and did not develop overt seizures. In behavioral tests, iLRRC8A KO did not impair memory or produce anxiety-like behavior. However, iLRRC8A KO mice exhibited mild hyperlocomotion in the open-field test and improved performance in the Rotarod test as compared to their littermate controls. In conclusion, loss of VRAC modulates GABAergic interneuron function to produce behavioral changes in iLRRC8A KO mice. Additional work is needed to characterize the electrophysiological properties of GABAergic LRRC8A KO interneurons. Critically, interneuronal ablation of LRRC8A does not recapitulate the lethal seizure phenotype previously reported in brain-wide LRRC8A KO. The latter finding points to additional critical contributions of other cell types to the pathological hyperexcitability caused by the loss of VRAC in the brain.

# Abstract: P01.020 – Pediatric OSA and Stem Cell Function

**Published:** 2025-09-05

**Authors:** Arvind Chandrakantan

**Affiliations:** a Bcm/tch, Houston, TX, USA

## Abstract

**Introduction:**

Pediatric obstructive sleep apnea (OSA) is a common pediatric disease of childhood affecting up to 7.5% of all children worldwide. There are several end organ sequelae, including neurocognitive changes, which have been characterized in clinical studies. The lack of a basic science model has hampered scientific elucidation of the neurocognitive phenotype.

**Methods:**

We have devised a model system using intermittent hypoxia (IH) as the stimulus for OSA. We commenced exposure at p14 in neonatal mice, and used 3 tractable exposure based time points in order to characterize learning and memory task ability. The model utilized the following in order to create the IH regimen utilized:

1. Human polysomnographic data: We studied human data in children with OSA
2. Murine oximetry data: we create murine oximetry curves
3. Cross Species Curve Fitting: We cross fit those across species
4. Optimization of hypoxic gas throughputs: We then optimized this for the hypoxia chamber we are using

The learning and memory tasks utilized were 2 hippocampal dependent tasks (NOR and OPR), and 2 independent tasks (HP and OFA). after characterization of behavior, we then performed synaptic staining, cell counts of all cell lineages, and EdU staining.

In the OB, we also studied behavior with regards to olfactory sensitivity and discrimination, synaptic assays, and cell counts.

**Results:**

We examined 4 behavioral tasks over a number of time periods to establish the temporality needed for hippocampal task ablation, as the hippocampus is the center of learning and memory in mammals. We utilized 3 time periods initially: exposure from p14 to p23 (9 days acute), to p35 (21 days intermediate), and p70 (56 days, chronic). There was no deterioration in hippocampal tasks from p14 to p35, however at p70 we noted task ablation.

Synaptic assays demonstrated no significant difference in excitatory synapse colocalization (VGLUT/PSD95) or inhibitory (VGAT/gephyrin).

Our cellular assays demonstrated an increase in EdU specifically NeuN/EdU in the hippocampus, as well as astrocytes and oligodendrocytes. Characterization of astrocytic complexity through Scholl analysis also demonstrated diminished complexity of IH exposed astrocytes. EdU studies of the olfactory bulb are pending at the time of submission.

**Conclusion:**

1) We have demonstrated a tractable, temporal model for pediatric OSA over a systematic set of time points to show when hippocampal deficits from the OSA stimulus manifest phenotypically. We have further isolated these deficits to the hippocampus of developing mice, which is the mammalian center of learning, memory, and retrieval. 2) We have also demonstrated olfactory deficits, suggesting that both areas of postnatal mammalian neurogenesis are involved.

We further intended to elucidate stem cell function through a transgenic mouse model to study pathways of stem cell differentiation. This study will provide initial insights into how pediatric OSA effects postnatal stem cell function, which will provide translational value to children affected by the disease.

# Abstract: P01.021 – Characterization of Aβ and Phosphorylated Tau Changes Across the Gut-Brain Axis of AD and Control Individuals

**Published:** 2025-09-05

**Authors:** Sathiya Chandrasekaran, Angela Floden, Colin Combs

**Affiliations:** a University of North Dakota, Grand Forks, ND, USA

## Abstract

Alzheimer’s disease (AD) patient brains are characterized by robust extracellular amyloid-β (Aβ) plaques and intracellular tau-containing neurofibrillary tangles. We and others have shown that the gastrointestinal tract can also produce Aβ and contains hyperphosphorylated tau. Moreover, it has been shown that administration of Aβ into the gastrointestinal tract of mice induces AD-like pathology likely via vagal communication mechanisms. Collectively, this suggests that neuronal connectivity via the vagus may allow Aβ and tangle pathology to communicate between the intestines and brain. The objective of this study was to examine possible translocation of Aβ or phosphorylated-tau (p-tau) pathology from the gut to the brain or vice versa in the same individuals. Temporal cortex, medulla, vagus, and colon tissue sections (5–6 µm) from male cognitively normal control (*n* = 9) and AD subjects (*n* = 7), aged 65–99 years, were obtained from Banner Sun Health Research Institute. Immunohistochemistry was performed using antibodies against Aβ, p-tau (AT8, pSer202/pThr205; PHF-1, pSer396/404) using the visual light chromogen, vector VIP. The scanned microscopic images were visually scored semi-quantitatively on a scale of 0–3 (0-none, 1-sparse, 2-moderate, 3-frequent immunoreactivity) and the observers (*n* = 3) were blinded to the experimental specimens in order to avoid any potential bias. The results showed that the temporal cortex and medulla of AD patients had significantly increased positive staining for Aβ when compared to controls. However, Aβ immunoreactivity was absent in the vagus nerve and positive but not significantly different in the colons of the control and AD groups. With respect to tau pathology, the temporal cortex and medulla of AD patients displayed both AT8 and PHF-1 immunoreactivity. The vagus nerve and colons from control and AD patients did not show AT8 positivity. However, PHF-1 staining was positive in both the vagus nerve and colon although not significantly different between the control and AD individuals. Our results suggest that the Aβ/AT8 p-tau pathologies observed in the temporal cortex and medulla of AD patients do not propagate bidirectionally between the brain and intestine through the vagus nerve. In addition, the presence of Aβ in the colons and PHF-1 immunoreactivity in the vagus nerve and colons of control and AD humans warrants further studies verifying a gut-brain axis communication.

# Abstract: P01.022 – Exploring the Mechanisms of STX, a Novel Estrogen Receptor Modulator That Protects Against Amyloid-Beta Neurotoxicity in Alzheimer’s Disease Models

**Published:** 2025-09-05

**Authors:** Philip Copenhaver, Zoe Bostik, John Doherty, Hun-Joo Lee, Martin Kelly, Joseph Quinn

**Affiliations:** a Oregon Health and Science University, Portland, OR, USA

## Abstract

Despite recent progress with antibody-based therapies to treat Alzheimer’s Disease (AD), more affordable treatment options that can be safely administered as a preventative strategy are still needed. Notably, almost two thirds of AD patients are women, and the loss of ovarian steroids at menopause significantly increases vulnerability to AD pathogenesis. Estrogen replacement can have neuroprotective effects but also carry unacceptable risks for elderly patients. As an alternative, we have investigated the benefits of STX, a novel tamoxifen analog that may provide the neuroprotective benefits of estrogen without its side effects. Unlike 17β-estradiol (E2), STX does not engage classical estrogen receptors (ERs); rather, it activates a G protein-coupled membrane estrogen receptor (GqMER), which is expressed by CNS neurons but not peripheral reproductive organs. Oral STX readily crosses the blood-brain barrier and can be safely administered for sustained periods, while studies in animal models have shown that STX recapitulates the beneficial effects of E2 in animal models of menopause and ischemia without its side effects. Accordingly, STX can be considered a ‘neuro-SERM’ (selective estrogen receptor modulator), capable of inducing neuroprotective responses in the brain without the adverse outcomes linked with conventional hormone replacement therapies. In previous work, we showed that STX treatment attenuated Amyloid-β (Aβ) neurotoxicity in vitro, in part by mitigating mitochondrial dysfunction and synaptic toxicity. Subsequent studies using the 5XFAD mouse model showed that STX attenuated Aβ-associated mitochondrial toxicity and synaptic loss in the brain, similar to its effects on cultured neurons. STX also moderately improved spatial memory, while reducing reactive astrocytosis and microgliosis. Engagement of GqMER by STX can regulate responses via several transduction pathways in different contexts. Using neuroblastoma cell lines and primary mouse hippocampal neurons, we focused on the PI3K-Akt pathway, based the neuroprotective effects of STX in ischemia models. Using MC65 cells (an inducible model of amyloid toxicity), we found that STX protected against the cytotoxic effects of Aβ, while supporting endogenous levels of Akt and GSK3β phosphorylation (downstream targets of PI3K). These protective effects were blocked by the broad spectrum PI3K inhibitor LY294002. In primary cultures of hippocampal neurons from the 5XFAD model, STX protected against the loss of dendritic complexity caused by Aβ.These beneficial effects were blocked by the pan-P110 inhibitors PI-103 and BKM120. Subsequent Sholl-based assays showed that the protective effects of STX were also reduced by specific inhibitors of the P110β and P110δ isoforms of the PI3K catalytic subunit, but not P110α or P110γ. In complementary studies using dense neuronal cultures, STX caused a rapid increase in Akt and GSK3β phosphorylation, a response that was again blocked by inhibiting P110δ but not P110α or P110γ. Interestingly, inhibiting phospholipase C (PLC) with U73122 also moderately reduced the protective effects of STX. These results suggest that activation of GqMER by STX in hippocampal neurons induces neuroprotective responses via engagement of complementary transduction pathways that regulate mitochondrial integrity and calcium homeostasis. They also provide the framework for investigating how STX protects against neuronal dysfunction in animal models of AD.

# Abstract: P01.023 – Calcium Signaling in Schwann Cells Development and Myelination

**Published:** 2025-09-05

**Authors:** Jazmin Corral, Karissa Garbarini, Veronica T. Cheli, Pablo M. Paez

**Affiliations:** a Institute for Myelin and Glia Exploration, Department of Pharmacology and Toxicology, Jacobs School of Medicine and Biomedical Sciences, The State University of New York, University at Buffalo, Buffalo, NY, USA

## Abstract

Schwann cells are the major glial cells in the peripheral nervous system (PNS), and they are essential for the maintenance and myelination of peripheral nerves. In the central nervous system, the importance of G-Protein coupled receptors (GPCRs) modulating Ca^2+^ signaling in oligodendrocytes and neurons has previously been shown. However, there is a lack of information of how the modulation of Ca^2+^ signaling by GPCRs in Schwann cells affect myelination of the PNS. Using Cre-mediated recombination we specifically expressed the excitatory hM3Dq and the inhibitory hM4Di GPCRs in Schwann cells. These receptors were created from different subtypes of human muscarinic receptors and are exclusively activated by clozapine N-oxide (CNO). We performed Ca^2+^ imaging, immunocytochemistry, and electron microscopy experiments to assess Schwann cell development and function after hM3Dq and hM4Di activation in vitro as well as in vivo. Our results show that hM3Dq activation during early development significantly delays the myelination of the sciatic nerve and the maturation of Schwann cells. Furthermore, hM3Dq activity in mature Schwann cells disrupts the myelin sheath and induces a severe demyelination in the adult sciatic nerve. In contrast stimulation of hM4Di increases myelin synthesis and Schwann cell proliferation in adult peripheral nerves. We have conducted Ca^2+^ imaging experiments to determine how hM3Dq and hM4Di affects the activity of Ca^2+^ channels and receptors in Schwann cells and we have also performed behavioral test, such as Rotarod and Catwalk, to study how changes in myelination induced by hM3Dq and hM4Di impact motor coordination in young and adult mice.

# Abstract: P01.024 – Discoidin Domain Receptor Signaling Regulates Ensheathment and Caliber of Peripheral Axons

**Published:** 2025-09-05

**Authors:** Megan Corty, Alexandria Hulegaard, Jo Hill, Sue Aicher, Marc Freeman

**Affiliations:** a University of Arizona, Tucson, AZ, USA; b Vollum Institute, Portland, OR, USA; c Oregon Health and Science University, Portland, OR, USA

## Abstract

How non-myelinating axonal ensheathment develops and its roles in supporting long-term neuronal health and function remain unclear even though the majority of axons in mammalian peripheral nerves are not myelinated. In Drosophila, specialized wrapping glia ensheathe axons in peripheral nerves in a manner closely resembling vertebrate non-myelinating (Remak) Schwann cells. In addition to morphological similarities, recent studies have shown molecular conservation between the control of ensheathment in the fly and vertebrates, and we use fly wrapping glia as a model to study non-myelinating ensheathment. We identified discoidin domain receptor (Ddr) and multiplexin (Mp) in a large-scale RNAi screen for novel regulators of ensheathment. Ddr encodes an evolutionarily conserved receptor tyrosine kinase activated by collagens. We found that loss of Ddr in wrapping glia by RNAi or mutation results in profound defects in axonal ensheathment in larval nerves. Genetic interaction experiments demonstrate that Ddr works in an autocrine fashion with the Type XV/XVIII collagen Multiplexin (Mp) to regulate axon wrapping in larval nerves. Extending our studies to adult nerves to study non-myelinating ensheathment over the lifespan, we found that glial knockdown of Ddr increases age-dependent neurodegeneration and are currently investigating the mechanism by which Ddr signaling impacts neuronal survival. Surprisingly, we also found roles for non-myelinating glia in regulating axon caliber during a period of nerve/axon maturation. Ablation of wrapping glia or glia-specific knockdown of Ddr results in decreased axon calibers in fly nerves. These studies establish important new roles for non-myelinating ensheathment and Ddr in proper neuronal health and maturation, and raise new questions about how this glial signaling pathway impacts axon biology.

# Abstract: P01.025 – B Cell Depletion Reduces Chronic but Not Acute Cognitive Deficits After Prefrontal Stroke in Aged Female Mice

**Published:** 2025-09-05

**Authors:** Miss. Katherine Cotter, Thomas Ujas, Mary Colson, Pavel Yanev, Edric Winford, Jadwiga Turchan-Cholewo, Ann Stowe

**Affiliations:** a University of Kentucky Department of Neuroscience, Lexington, KY, USA; b University of Kentucky Center for Advanced Translational Stroke Science, Lexington, KY, USA; c University of Kentucky Department of Neurology, Lexington, KY, USA

## Abstract

**Background:**

B cells migrate into the hippocampus after stroke, and remain there long-term, but the benefits to recovery are unclear in aged animals. Autoshaping (AUTO) is a Pavlovian response learning task sensitive to lesions in the hippocampus and frontal cortex. It utilizes an unconditioned reward to build an association with a conditioned stimulus (CS+) on a touchscreen platform, which has not previously been used to track cognitive decline in aged mice.

**Aim:**

Based on our prior studies, we hypothesized that B cell depletion would result in more severe post-stroke cognitive deficits in the AUTO task in aged animals. Additionally, we hypothesized that this decline in performance correlates with the loss of neuroprotective B cells in the hippocampus and pre-frontal cortex.

**Methods:**

Female aged (16–24 month-old, *n* = 20) hCD20tamCRE(+)/fBDNF(+/+) mice and hCD20tamCRE(-)/fBDNF(+/+) littermate controls were first trained on the Initial Touch phase of the Paired Associate Learning task to confirm motivation for a peanut oil reward. Mice were then trained on the AUTO operant task. Rituximab was injected IP for 3 days before AUTO baseline, with weekly doses to maintain depletion though this failed in 4 mice that were thus reassigned to groups based on immunophenotype. Following AUTO baseline, mice underwent a bilateral prefrontal photothrombotic stroke. Mice completed the AUTO task for 3 days on weeks 2, 4, and 6 weeks post-stroke to measure cognitive function, with splenic B cell depletion confirmed at 6 weeks post-stroke using flow cytometry. Primary AUTO measures were number of trials/session, number of approaches, approach difference, and approach latency. An additional cohort of female aged (9–21-month-old, *n* = 13) hCD20tamCRE/fBDNF(+/+) mice is ongoing with a diluted strawberry milkshake reward to confirm long-term saliency for task acquisition.

**Results:**

Unlike AUTO tasks using strawberry milkshake in young (3–6 mos.) male and female mice that reached criterion (40 trials/60 min) typically on day 1 (PMID:36564387), aged mice on peanut oil reward averaged 26 trials/session with only 7/20 mice completing trial numbers typical of younger cohorts. Several mice (6/20) did not respond as well to peanut oil but remained within the cohort.

In the first cohort, Rituximab induced long-term depletion of splenic B cells by 76.3% (*p* < 0.0001). There was a significant interaction observed between the B cell depletion and the time post-stroke on the approach difference between CS + and CS- (2-way rmANOVA; *F*(3,54) = 2.957; *p* = 0.0404). The B cell-depleted group exhibited an improvement in cognitive function starting at 4 weeks post-stroke, while the WT group had declined cognitive function starting at 4 weeks post-stroke. Localization of B cells in the hippocampus and prefrontal cortex is currently being quantified to confirm splenic immunophenotyping.

**Conclusions:**

Based on our prior work in young male mice, we expected larger deficits in B cell-depleted aged females. The data from the first cohort support this only in the acute phase. An opposite protective effect was shown in the chronic phase, with B cell-depleted animals improving in their cognitive performance at 4 weeks post-stroke (*p* = 0.0089) confirming previous studies showing a detrimental effect of B cells on cognition in young mice (PMID:25653369).

# Abstract: P01.026 – The Antiretroviral Drug Dolutegravir Inhibits Oligodendrocyte Maturation In Vitro

**Published:** 2025-09-05

**Authors:** Melanie Cruz Berrios, Marisa Jeffries, Raj Patatunda, Judith Grinspan, Kelly Jordan-Sciutto

**Affiliations:** a University of Pennsylvania, Philadelphia, PA, USA; b Children’s Hospital of Philadelphia, Philadelphia, PA, USA

## Abstract

As many as 50% of people living with human immunodeficiency virus (HIV) develop HIV-associated neurocognitive disorders (HAND). These are associated with cognitive decline and strongly linked with white matter and myelin pathologies. Strikingly, the overall prevalence of HAND and associated white matter pathology remains unchanged despite widespread antiretroviral therapy (ART) use, suggesting that either the mechanisms driving HAND are not effectively targeted by ART or that ART contributes to this pathology. Adolescents disproportionately constitute 21% of new HIV cases and may be especially vulnerable to white matter pathologies arising from either HIV infection or ART as myelination continues throughout adolescence. One of the ART drugs approved for use in adolescents is the integrase strand transfer inhibitor dolutegravir (DTG). To study whether DTG affects myelination, we employed purified primary rat cultures of oligodendrocytes (OL), the glial cells of the central nervous system that make myelin in white matter. Doses of DTG, centered on human plasma Cmax (800 nM, 8 µM (Cmax), or 24 µM), were applied to OL cultures at various stages of differentiation. Treating OL progenitors with DTG for 24 h prior to initiating differentiation resulted in impaired OL maturation at the highest dose, 24 µM DTG, assessed by Western blotting and immunocytochemistry (ICC) for myelin proteins GalC, MBP, and PLP after 72 h of differentiation. Likewise, treating OLs with DTG only during differentiation for 72 h yielded similar reductions in maturation at the highest dose, and delaying the treatment for 24 h after differentiation had begun did not change the effect on maturation. Strikingly, extended DTG treatment for 24 h prior to differentiation and 72 h during differentiation resulted in significantly fewer GalC + immature OLs and PLP + mature OLs by ICC and significantly reduced MBP expression by Western blotting at all 3 doses of DTG, suggesting that treatment during both phases exacerbates inhibition of OL maturation of treatment. These data demonstrate that DTG impairs OL maturation regardless of the time and duration, but most effectively with extended treatment during both progenitor and differentiation phases. One potential mechanism of the observed deficits may be the integrated stress response (ISR), as extended DTG treatment strongly activates the ISR in OLs, with a significant increase in p-eIF2α at 8 and 24 µM and an increase in ATF4 nuclear localization across all doses. To determine if it was possible to reverse the effects of DTG, ISR inhibitor (ISRIB) was added to OL cultures during extended treatment. However, ISRIB was unable to rescue the OL maturation deficits, indicating that another mechanism may be responsible. Given that OL progenitors continue to proliferate and differentiate during adolescence, adolescents taking DTG may exhibit impaired myelination. Ongoing studies include in vivo treatment of adolescent rats with DTG to examine white matter development.

# Abstract: P01.027 – Diminished Tetrahydrobiopterin (BH4) in Multiple Sclerosis and Its Effects on Oligodendrocytes and CNS Myelination

**Published:** 2025-09-05

**Authors:** Zaenab Dhari

**Affiliations:** a UConn Health and Trinity Health, Granby, CO, USA

## Abstract

Tetrahydrobiopterin (BH4) is endogenous cofactor involved in multiple antioxidant pathways. Previous work has reported that hypoxia reduces brain BH4 levels and BH4 supplementation can rescue myelination deficits in mice following chronic hypoxia. Here, we evaluated BH4 in Multiple sclerosis (MS) patients and examined whether a clinical formulation of BH4 (Kuvan^™^) impacts oligodendrocyte differentiation and CNS myelination in a non-autoimmune model of progressive demyelinating disease. Mass spectrometry of plasma BH4 among MS patients and healthy controls (HC), (*n* = 40) confirmed lower levels in MS patients [(6 ± 2) versus age and sex matched controls (7.7 ± 2) ng/mL, *p* < 0.01]. Analysis of primary oligodendrocyte progenitor cells (OPCs) determined that application of BH4 (10uM) increased oligodendrocyte maturation (Olig2+/MBP+). Administration of BH4 via oral gavage to C57BL/6 mice undergoing cuprizone (0.2% w/w) for 4 weeks enhanced axonal myelination (G ratio) and dramatically improved motor coordination as assessed by accelerated rotarod assays. Interestingly, BH4 treatment improved motor coordination even 4 weeks after discontinuation of treatment. An analysis of published single cell RNA sequencing data from human MS postmortem brains revealed that de novo synthesis gene pyruvoyltetrahydropterin synthase (PTS) required for BH4 production was downregulated, while the salvage pathway gene dihydrofolate reductase (DHFR) was concomitantly upregulated, suggesting impaired metabolism of BH4 within demyelinated MS lesions. Taken together, these data provide insight to the potential endogenous role of BH4 and its regulation in MS as it relates to CNS demyelination. Defining how BH4 supplementation can enhance OPC differentiation and offset CNS demyelination may identify novel links to the consequences of altered BH4 metabolism in MS patients as they contribute to chronic demyelination.

# Abstract: P01.028 – Dock1 Functions in Schwann Cells to Regulate the Development, Maintenance, and Repair of the Peripheral Nervous System

**Published:** 2025-09-05

**Authors:** Ryan Doan, Kelly Monk

**Affiliations:** a The Vollum Institute – Oregon Health and Science University, Portland, OR, USA

## Abstract

Schwann cells, the myelinating glia of the peripheral nervous system (PNS), are critical for myelin development, maintenance, and repair. Rac1 is a known regulator of radial sorting, a key step in developmental myelination, and we previously showed in zebrafish that loss of Dock1, a Rac1-specific guanine nucleotide exchange factor, results in delayed peripheral myelination in development. We demonstrate that Dock1 is necessary for myelin maintenance and remyelination after injury in adult zebrafish. Furthermore, it performs an evolutionary conserved role in mice, acting cell-autonomously in Schwann cells to regulate peripheral myelin development, maintenance, and repair. Additionally, manipulating Rac1 levels in larval zebrafish reveals that dock1 mutants are sensitized to inhibition of Rac1, suggesting an interaction between the two proteins during PNS development. We propose that the interplay between Dock1 and Rac1 signaling in Schwann cells is required to establish, maintain, and facilitate repair and remyelination within the peripheral nervous system.

# Abstract: P01.029 – Differential Responses of Reactive Astrocytes to Antagonists of CSF1 Receptor in a CLN2 Mouse Model

**Published:** 2025-09-05

**Authors:** Miriam Domowicz, Nicole Pagan Torres, Christopher Ware, Samantha Elizalde, Nancy Schwartz

**Affiliations:** a University of Chicago, Chicago, IL, USA

## Abstract

We are studying the roles of glial cells crosstalk in a pediatric neurodegenerative disease mouse model of Neuronal Ceroid Lipofuscinoses type 2 (CLN2). In this model the knockout of the tripeptidyl peptidase 1 (Tpp1) gene leads to progressive neurological phenotypes that include ataxia, vision loss, decrease motor function and early death, mimicking the development of the human disease. Global RNA-seq analysis indicated that the neuroinflammation process develops between 2 and 4 months in this model and is accompanied by activation of microglia and astrocytes, increase production of nitric oxide and reactive oxide species, and activation of leukocyte extravasation. Furthermore, evidence of the stage 1 and 2 disease-associated microglia (DAM) signature starts to be evident as early as 3 months. In other neurodegenerative diseases, depleting microglia was beneficial in stalling the neurodegenerative process. Thus, we depleted microglia using inhibitors of the CSF1 receptor (PLX5622; PLX3397) in both in vivo and slice culture systems in order to understand the microglial involvement in this model. In both systems, there was a downregulation of microglial genes when treated with the inhibitor accompanied by an upregulation in astrogliosis, reflected by increased expression of Gfap and Aqp4 in control and Tpp1-/- brains. Also, longer term exposure to PLX5622 accelerated lethality in Tpp1-/- mice. This astrogliosis activation could indicate increase astrocytic phagocytosis due to microglia cell death since this antagonist affects microglia survival. To avoid this issue we have tested the CSF1R antagonist GW2580, that has been reported to prevent cell proliferation without affecting microglia survival. In slice culture experiments we demonstrated that GW2580 inhibits microglia activation markers like Ctss and CD68 while reducing levels of astrogliosis markers. These data suggest that GW2580 may be a better therapeutic candidate for controlling the development of chronic inflammation in CLN2. Taken together our data show that microglia play a protective role during the neuroinflammation process in Tpp1-/- mice and that microglia-astrocyte communication is essential for controlling the progression of the neurodegenerative process.

# Abstract: P01.030 – The Muscle-Brain Axis of Resilience Explored Through Differential Gene Expression in Muscle and Brain of the Hibernating Arctic Ground Squirrel

**Published:** 2025-09-05

**Authors:** Kelly L. Drew, Inigo Yoldi Bergua, Sarah A. Rice, Vadim B. Fedorov, Anna V. Goropashnaya

**Affiliations:** a University of Alaska Fairbanks, Fairbanks, USA

## Abstract

Sarcopenia, the age and disuse-related loss of muscle mass and strength due to reduced physical activity, is closely linked to age-related diseases and Alzheimer’s disease. Hibernation is relevant to sarcopenia due to the unique ability of hibernating mammals to preserve muscle mass during extended periods of inactivity and reduced energy expenditure. Like muscle, hibernating mammals also preserve most aspects of cognitive function despite prolonged disuse. Moreover, cooling and rewarming is known to promote synaptic plasticity. In hibernating arctic ground squirrels (AGS), RNA-seq analysis has shown modulation of gene expression in muscle, with a reduction in genes associated with muscle protein degradation and an increase in genes related to muscle protein anabolic pathways, indicating a common regulatory mechanism. The specific regulatory mechanisms and the full extent of gene expression changes in the hibernating brain, especially in relation to the muscle-brain axis and preserved anabolic sensitivity, are areas that require further investigation. Here our objective was to compare gene expression in brain and muscle of hibernating AGS to further explore aspects of the muscle-brain axis that could promote anabolic sensitivity in brain and muscle to identify processes sufficient to preserve brain and muscle function during prolonged periods of disuse. Tissue was harvested from male and female AGS during the summer active season, torpor, and interbout arousal (IBA) or early onset of torpor. Hippocampus was dissected from partially thawed whole brain prior to RNA extraction. Quadriceps skeletal muscle tissue was rapidly dissected and frozen in liquid nitrogen within 9 min of death and stored at −80 °C until RNA extraction. Total RNA samples were used for cDNA library construction and sequencing by BGI Americas Corporation (Cambridge, MA) and analyzed using CLC Genomics Workbench (QIAGEN). Genes were mapped to the 13-lined ground squirrel or AGS genome. A greater number of genes were detected in the brain than in the muscle. The maximal number of differentially expressed genes were found in torpor compared to summer active animals: 3,236 in the brain and 628 in the muscle. Among those, 177 genes were shared between the two tissues with 137 genes significantly changed in the same direction. Gene set enriched analysis showed elevated proportion of over-expressed genes involved in inflammatory response in brain during hibernation. Ribosome biogenesis and translation gene sets were enriched with over-expressed genes in muscle during torpor and IBA, and in brain during torpor, compared to summer. Cell cycle genes were upregulated in brain during both stages of hibernation compared to active AGS. RNA Binding Motif Protein 3 (RBM3) expression was universally upregulated in winter compared to summer in muscle and brain. RBM3 is an evolutionarily highly conserved RNA binding protein implicated in various cellular processes, including translation regulation and protection against cell death. These findings point to RBM3 as a biomarker of the hibernation season, and as a potential regulator of regenerative or protective processes in muscle and brain.

# Abstract: P01.031 – Senolytic Therapy Maintains BBB Integrity, Alleviates Cerebral Hypometabolism and Stabilizes Microglia Homeostatic Subtype in the PS19 Mouse Model

**Published:** 2025-09-05

**Authors:** Minmin Yao, Zhiliang Wei, Aaron Kakazu, Yuxiao Ouyang, Jiadi Xu, Manisha Aggarwal, Hanzhang Lu, Wenzhen Duan

**Affiliations:** a Johns Hopkins University School of Medicine, Baltimore, MD, USA; b Kennedy Krieger Institute, Baltimore, MD, USA

## Abstract

**Objectives:**

Cellular senescence has been observed in both Alzheimer’s disease (AD) patients and animal models. In this study, we investigated the effects of senolytic therapy in the PS19 mouse model by exploring human translatable MRI measures and mechanisms involved in senolytic therapy in the Tau model.

**Methods:**

PS19 mice were treated with dasatinib plus quercetin (DQ) from 3 months of age for 6 months. T2 relaxation under spin tagging (TRUST) and phase contrast (PC) MRI was used to assess cerebral metabolic rate of oxygen (CMRO2), water extraction with phase-contrast arterial spin tagging (WEPCAST) MRI was employed to detect the blood-brain barrier (BBB) permeability longitudinally. Brain volumes were analyzed by diffusion tensor imaging (DTI) at the end of study. Cognitive function was evaluated by the tracing fear conditioning test along MRI measures.

**Results:**

PS19 mice displayed significantly reduced cerebral CMRO2 and increased BBB permeability to water at 9 months of age. Meanwhile hippocampal atrophy and impaired cognitive function were evident in PS19 mice at the same age. DQ senolytic treatment dramatically mitigated cerebral hypometabolism, maintained BBB integrity, attenuated hippocampal atrophy, reduced tauopathy and improved cognitive function. Furthermore, the DQ treatment led to a shift of microglia from a disease-associated subtype to a homeostatic subtype.

**Conclusions:**

These findings provide evidence for the potential therapeutic benefits of senolytic therapy and highlight the involvement of microglia in the underlying mechanisms. The use of human translatable biomarkers in the PS19 mouse model contributes to our understanding of the brain functional changes associated with tauopathy and the effects of senolytic therapy.

# Abstract: P01.032 – Harnessing Spatial Transcriptomics to Investigate the Intersection of Senescence and Inflammation in Neurodegeneration

**Published:** 2025-09-05

**Authors:** Violeta Duran Laforet, Christina Hung, Rebecca Beiter, Pablo Gimenez Gomez, Robert Murphy, Dorothy Schafer

**Affiliations:** a University Of Massachusetts Chan Medical School, Worcester, MA, USA

## Abstract

Senescence is a physiological process, which has historically been associated with aging and an inability to undergo cell division. At the same time, senescent cells enter a chronic inflammatory state, which includes secretion of the senescence-associated secretory phenotype or SASP, a cocktail of secreted inflammatory mediators (cytokines, matrix metalloproteases, etc.), which impacts the function of surrounding cells. In addition to aging, recent evidence now implicates senescent cells in neurodegeneration in diseases such as Alzheimer’s Disease and Multiple Sclerosis. However, it remains to be determined where these senescent cells are localized in the brain during neurodegeneration and whether senescence influences the molecular phenotype of these cells. We are now using MERFISH (multiplexed error robust fluorescence in situ hybridization), an innovative spatial transcriptomic technique to measure the copy number and spatial distribution of 100’s of senescence-related genes directly in human and mouse brain tissue. In the process, we have identified that the distribution of senescence cells is highest in Alzheimer’s disease-relevant neurodegeneration vs. normal aging or acute neuroinflammation. Further, of the cell types that have this senescent signature, microglia are the most pronounced. We are now working towards identifying senescence-related secreted factors from microglia that influence neurodegeneration.

# Abstract: P01.033 – Investigating PC12 Cell Differentiation to Sympathetic Neuron-Like Phenotype Through Single-Cell Proteomics Analysis

**Published:** 2025-09-05

**Authors:** Arpa Ebrahimi

**Affiliations:** a Oregon State University, Corvallis, OR, USA; b University of British Columbia, Vancouver, Canada

## Abstract

This study utilizes the potential of single-cell proteomics to explore the intricacies of cellular differentiation processes. Specifically, we investigate the differentiation of PC12 cells into a sympathetic ganglion neuron-like phenotype induced by nerve growth factor (NGF). Our objective is to closely monitor this differentiation process, identify protein and peptide markers indicative of cellular differentiation, and gain insights into the dynamic changes in the proteome throughout various differentiation stages. We determined the optimal NGF concentration and incubation duration required for PC12 cell differentiation, which was subsequently validated through imaging, revealing neurite growth using calcein AM cell-permanent dye. Our experimental approach involved the use of Tecan’s Uno Single Cell Dispenser^™^ for precise single-cell and low-volume reagent dispensing. Single-cell proteomic profiles were generated employing an Orbitrap Fusion Lumos instrument with data-dependent acquisition (DDA) coupled to a nanoflow UPLC system equipped with trapping and C18 peptide columns. Data processing was conducted using Proteome Discoverer v3.0. To ensure accurate single-cell isolation dispensing conditions for PC12 cells, fluorescence microscopy was employed for optimization. Initial experiments utilized bulk PC12 cells to establish proteomic profiles for both undifferentiated and differentiated cells. These profiles were subsequently employed to identify markers specific to PC12 cell differentiation, which were then compared to previously reported markers, including neurosecretory proteins such as VGF, neuromodulin, chromogranin A, annexin A2, peripherin, MAP1B, AHNAK, and secretogranin 2. Our analysis identified a total of 3,800 proteins from 410,961 MS2 scan events, with 40% of scans resulting in peptide spectrum matches (PSM). Single-cell studies monitoring the differentiation of PC12 cells into ganglion neurons yielded proteome profiles consisting of 500–800 identified proteins per cell. Multivariate analyses allowed us to distinguish these profiles into proteome subgroups, highlighting the clear differentiation between undifferentiated and differentiated PC12 states. In future research, we aim to delve deeper into characterizing sub-populations of cells during intermediate differentiation stages. This investigation not only contributes to our comprehension of the molecular mechanisms underlying cellular differentiation but also underscores the potential of single-cell proteomics as a potent tool in the field of neuroscience.

# Abstract: P01.034 – Oligodendrocyte-Derived Carnosine Protects the CNS From Lipid Peroxidation

**Published:** 2025-09-05

**Authors:** Benayahu Elbaz, Tanya Klein, Gwen Marie Schulz

**Affiliations:** a Northwestern University, Chicago, IL, USA

## Abstract

The lipid peroxidation byproduct acrolein (systematic name: propenal) is a highly toxic aldehyde that exerts strong toxic effects by covalently binding to surrounding macromolecules, such as proteins, RNA, DNA, and lipids. Due to the toxicity, cellular mechanisms evolved to detoxify acrolein by quenching acrolein and turning it into a non-toxic compound. In the CNS, acrolein is quenched by carnosine. Carnosine is generated by the enzyme Carnosine synthase 1 (Carns1). Aging is associated with reduced levels of carnosine in the brain. Restoring carnosine levels through food supplementation with carnosine restores the age-related reduction in brain activity in both mouse models and human patients. Nevertheless, the role of carnosine in the protection of the CNS is unknown, partially due to a lack of appropriate experimental models. We have generated a new mouse model carrying the Carns1 conditional allele. Since we found that the enzyme Carns1 is expressed in oligodendrocytes, we have used the Carns1 conditional allele in combination with the inducible PLP-CreERt line that allows tamoxifen-mediated recombination specifically in mature oligodendrocytes. Although carnosine is a ubiquitous metabolite in the body, we found that oligodendrocyte-specific ablation of Carns1 dramatically reduces the level of carnosine in the CNS, suggesting that oligodendrocytes are the sole source of the metabolite carnosine in the CNS. Using the EAE model of demyelination we found that oligodendrocyte-derived carnosine protects the CNS from inflammation and reduces the spread of damaged areas marked by acrolein adducts. Our data suggest that oligodendrocyte-derived carnosine protects the CNS from the toxic byproducts of lipid peroxidation.

# Abstract: P01.035 – Will Chronic Inhibition of Dual Leucine Zipper Kinase (DLK) Be Neuroprotective and Restore Neuronal Function in Aged-Demyelination?

**Published:** 2025-09-05

**Authors:** Katie Emberley, Greg J. Duncan, Samantha Ingram, Ahmed Abdelhak, Christian Cordano, Ari Green, Ben Emery

**Affiliations:** a Jungers Center for Neurosciences Research, Oregon Health and Science University, Portland, OR, USA; b Weill Institute for Neurosciences, University of California at San Francisco, San Francisco, CA, USA

## Abstract

Age-related remyelination failure and neuroinflammation are thought to be key drivers of neurodegeneration which underlies the accumulation of disability in demyelinating diseases such as multiple sclerosis (MS). The mechanisms underlying neurodegeneration in the context of aged demyelination are not completely understood, hindering the development of neuroprotective therapies. Here, we use a transgenic mouse model to experimentally induce demyelination. Tamoxifen-induced ablation of the myelin regulatory factor (Myrf) gene in mature oligodendrocytes using Myrf fl/fl; Plp1-CreERT mice results in CNS-wide demyelination. We administer tamoxifen to animals at eight weeks or one year of age to determine the effects of aging on remyelination and neurodegeneration. By studying mouse models of age-related remyelination failure and neuroinflammation, we aim to unravel the mechanisms driving neurodegeneration in the adult demyelinated central nervous system (CNS). We find the kinetics of demyelination are similar whether tamoxifen is administered at either eight weeks (young) or one year (aged) of age. However, young mice rapidly and efficiently remyelinate a vast majority of the total axons in the CNS by 20 weeks post-tamoxifen. In contrast, aged mice show incomplete remyelination with far fewer remyelinated axons at 20 weeks post-tamoxifen. Both the aged and young mice have microglial/macrophage activation during peak demyelination (10 weeks post-tamoxifen). However, the young microglia/macrophages become less ramified and reactive upon remyelination, whereas the aged microglia retain a foamy macrophage morphology. Congruent with these histological findings, the aged animals exhibit severe motor behavior deficits with no improvement on the rotarod assay, whereas the young mice exhibit substantial motor recovery during their remyelination phase. Assessing the effects of chronic demyelination and inflammation on the neuronal population, we find that the aged demyelinated mice display persistently elevated levels of serum neurofilament light chain (NfL) and staining for degenerating neurofilaments relative to their young counterparts, indicative of axonal injury. In addition, neuronal populations such as retinal ganglion cells show elevated staining for cleaved caspase-3 in the aged demyelinated mice, which is not seen in their young counterparts. Importantly, we also find that aged mice show heightened phosphorylation of c-Jun, a transcription factor activated in response to DLK activity, which we have recently found to mediate neuronal loss in young-adult mice genetically manipulated to prevent remyelination. Together, these results suggest age-related remyelination failure may drive neurodegeneration that is in part mediated by the DLK/c-Jun pathway. These aged demyelinated animals will serve as a useful model to study the possible long-term neuroprotective effects of pharmacological or genetic inhibition of the DLK/c-Jun pathway.

# Abstract: P01.036 – Investigating Astrocyte Endfoot Formation Dynamics During Development and Across Species

**Published:** 2025-09-05

**Authors:** Raja Estes, Cody Call, Teresa Stackhouse, Anusha Mishra, Kelly Monk

**Affiliations:** a Oregon Health and Sciences University, Portland, OR, USA

## Abstract

Astrocyte endfeet are specialized processes that play vital roles in supporting neuronal health by maintaining the blood-brain barrier and regulating cerebral blood flow. How these processes develop and mature remain unresolved questions, due to limited in vivo techniques that allow visualization of endfeet. Further, endfeet form in parallel with vascular development, but mechanisms influencing endfeet formation along mobile and dynamic angiogenic vessels are poorly understood. I am investigating the formation of endfeet using live imaging of astrocytes and vasculature in the optic tectum of larval zebrafish. I use sparse labeling to visualize single astrocytes within a zebrafish in which all vascular structures are also labeled. I perform confocal live-imaging in the hindbrain from 2.5 days post fertilization (dpf) to 10 dpf, spanning the full timeline of vascular development and astrocyte endfeet infiltration stages. My preliminary findings suggest that zebrafish endfeet begin contacting vasculature at 2 dpf and maintain contact on angiogenic vessels as they refine their architecture over development. Vasculature that are fully developed with a lumen and connecting vessels appear to be fully wrapped by endfeet, suggesting that astrocytes contact developing vasculature, then complete wrapping upon vessel maturation. To define evolutionarily conserved mechanisms of endfeet formation, I am also performing time course analyses of endfeet in both rats and mice during astrocyte development ranging from post-natal day(P) P6-P21 using immunohistochemistry and 3D reconstruction. Future studies will address the molecular determinants of endfeet formation in vivo using zebrafish and rodents. The findings from this project will fill a fundamental gap in the field by defining evolutionarily conserved mechanisms of astrocyte endfeet development and generating novel tools to study glia-vascular interactions in vivo.

# Abstract: P01.037 – Impact of Sound Repetition Rate on Cortical Development and Behaviors in Young Fmr1 KO Mice

**Published:** 2025-09-05

**Authors:** Anna Kulinich, Nadia Farooq, Kendal Tapia, Prof Khaleel Razak, Iryna Ethell

**Affiliations:** a University of California, Riverside, CA, USA

## Abstract

Fragile X syndrome (FXS) is the most common genetic cause of autism and intellectual disability. It is associated with a loss-of-function mutation in the Fragile X messenger ribonucleoprotein 1 (Fmr1) gene. The Fmr1 knock out (KO) mice display the core deficits of FXS, including sensory hypersensitivity and abnormal cortical processing and has been used as a major model to study FXS. Our previous studies have demonstrated beneficial effects of developmental sound exposure, and not attenuation, on molecular, cellular, and functional properties in the auditory cortex (AuC) of KO mice. However, it remained unknown what specific sound properties had beneficial effects. Here, we studied the effects of sound trains with two different repetition rates on mouse auditory cortex development and FXS-associated behaviors in a mouse model of FXS. In this study, Fmr1 KO and WT littermates were sound exposed (SE) to a 14 kHz tone with 1 Hz or 5 Hz repetition rate during postnatal (P)9-P21 developmental period. WT and Fmr1 KO mice raised in a regular vivarium (normal exposure, NE) or sound-attenuated chamber (AE) were used as control groups. Similar to our previous findings, we discovered that PV-positive cell density in layer (L)4 AuC was lower in AE KO mice compared to AE WT but was increased of KO mice following the exposure to both sounds with 1 Hz and 5 Hz repetition rates. However, developmental exposure to sound trains with 5 Hz but not 1 Hz repetition rate increased PV levels and the number of PV high expressing cells, while decreasing the density of cFos + cells in L4 AuC of Fmr1 KO mice. Analysis of the baseline cortical activity using electroencephalography (EEG) recordings revealed that sound trains with 5 Hz but not 1 Hz repetition rate restored resting state gamma power in AuC of Fmr1 KO mice to WT levels. Developmental exposure to sound trains with 5 Hz but not 1 Hz repetition rate also improved the fidelity of temporal processing in AuC of Fmr1 KO mice. Finally, behavior testing showed that exposure to sound trains with 5 Hz but not 1 Hz repetition rate improved anxiety but decreased sociability, whereas both repetition rates enhanced hyperactivity in KO mice. Summarizing, our results show that developmental exposure of mice to sound trains with 5 Hz but not 1 Hz repetition rate had beneficial effects on PV cell development, functional cortical responses and behaviors in KO mice. Our findings might have a significant impact on developing new therapeutic approaches to alleviate FXS phenotypes and open new possibilities for a combination of sound exposure with drug treatment.

# Abstract: P01.038 – Global Cerebral Ischemia Decreases Dendritic Spine Density, Increases Microglial Reactivity and Microglial/Synapse Co-Localization in a Mouse Model of Cardiac Arrest

**Published:** 2025-09-05

**Authors:** Macy Falk, Annabelle Moore, Erika Tiemeier, Nidia Quillinan, Jacob Basak

**Affiliations:** a Univeristy of Colorado Anschutz Medical Campus, Aurora, CO, USA

## Abstract

**Background:**

There are over 350,000 cases of out-of-hospital cardiac arrest in the United States every year. Even with improved survival rates, long-term cognitive dysfunction remains common in survivors. Acute neurologic deficits are likely attributed to the ischemia-induced death of CA1 hippocampal neurons. However, global cerebral ischemia also causes persistent synaptic deficits in surviving CA1 neurons in the period that extends beyond immediate cell death. The mechanism of ongoing synaptic dysfunction following global cerebral ischemia is not well understood, but effects on synaptic structures may play a role. The primary goal of this study is to elucidate changes in hippocampal dendritic spine numbers and structure after a cardiac arrest at both an acute and subacute time point following the injury. Increasing evidence also suggests that reactive microglia play an important role in modulating synaptic structures in the setting of brain injury through phagocytosis of synaptic material. Therefore, we also evaluated the extent of microglia activation after a cardiac arrest injury and assessed for changes in both microglial/dendritic spine interaction and microglial phagocytic capability.

**Methods:**

A cardiac arrest with cardiopulmonary resuscitation (CA/CPR) model was utilized to induce global cerebral ischemia in Thy1-GFP adult mice (age 8–12 weeks). Brain tissue was collected at 72 h and 7 days post-procedure and stained for Iba1 (marker of microglia cells) and CD68 (marker of phagocytosis). Images of the CA1 hippocampal region of the tissue were obtained using confocal microscopy and Imaris software was used for 3D reconstruction of spine, microglia, and CD68 surfaces. Dendritic spine density, microglia reactivity, and microglia-spine co-localization were measured and compared to sham animals.

**Results:**

Global cerebral ischemia in the setting of a CA/CPR injury induces a significant decrease in secondary dendritic spine density 72 h post-injury in the hippocampus (1.7 versus 2.5 spines/µm, *p* = 0.016), with a trend toward maintained reduction up to 7 days in CA/CPR compared to sham animals. Microglia cells show persistent activation in cardiac arrest animals 72 h post-injury compared with sham animals. Additionally, microglia-spine interactions are increased in CA/CPR animals with significantly higher levels of spines showing >70% engulfment (6.6% versus 2.9%, *p* = 0.0013). CD68 expression, a marker of cellular phagocytosis, is also significantly increased in the microglia of CA/CPR animals at 72 h (13.6% versus 2.9% of microglia surface, *p* < 0.0001), with a trend toward a higher percentage of spines within microglia showing colocalization with CD68 compared with sham animals.

**Conclusions:**

Our results demonstrate an effect of global cerebral ischemia on decreasing synaptic spine density, suggesting that a decrease in post-synaptic spine numbers may play a role in the long-term cognitive deficits experienced after ischemic brain injury. Furthermore, the reduction in spine density may be impacted by persistent microglia activation and phagocytosis of dendritic spines for several days following the initial injury. These results allow us to propose novel therapeutics targeting microglia to improve cognitive outcomes following ischemic brain injuries.

# Abstract: P01.039 – Efforts to Mitigate Neurodegeneration in a Model of Traumatic Optic Neuropathy

**Published:** 2025-09-05

**Authors:** Micah Feri, Melika Rezanejad, Seema Tiwari-Woodruff

**Affiliations:** a Division of Biomedical Sciences, School of Medicine at University of California Riverside, Riverside, CA, USA

## Abstract

Axon degeneration is an event that occurs in many neurodegenerative disorders and drives disability and disease progression. This process is active and involves activation of sterile alpha and Toll/interleukin-1 receptor motif containing 1 (SARM1). SARM1’s NADase activity is essential to its pro-degenerative function, making it a strong target to treat neurodegenerative diseases such as amyotrophic lateral sclerosis, Parkinson’s disease, and multiple sclerosis. There has been very little work done to address and mitigate the process of axonal degeneration. Recently, a class of orally bioavailable small molecule inhibitors of SARM1 were developed and demonstrated protection against loss of axon function in a model of chemotherapy induced peripheral neuropathy. Here, we investigated the efficacy of small molecule SARM1 inhibitor, 5-iodoisoquinoline (5IIQ) after optic nerve crush (ONC) injury, a model of traumatic optic neuropathy. An ONC was performed for 5 seconds 1 mm behind the optic disc in a group of age and sex-matched C57BL/6J mice. A subset of mice was used as sham controls. Mice were treated with vehicle or 5IIQ at day 0 post injury (dpi). Visual function was assessed by visual evoked potentials (VEPs) and electroretinograms (ERGs) at 14 dpi followed by perfusion for immunohistochemistry (IHC) analysis. Visual function analysis revealed VEP and ERGs with near normal amplitudes and latencies in sham group. Vehicle-treated ONC groups displayed decreased amplitudes in both VEPs and ERGs. 5IIQ-treated ONC groups displayed significantly VEP and ERG amplitudes similar to sham controls suggesting mitigation of ONC-induced axon damage. IHC analysis showed robust expression of NeuN positive cells in the retina of Sham groups. However, significantly less NeuN positive cells in the retina was observed in vehicle-treated and 5-IIQ-treated ONC 14 dpi compared to sham controls group. Myelinated axons with baseline levels of inflammation, higher NMNAT2 staining intensity, and lower SARM1 intensity was observed in optic nerve sections from sham controls. Interestingly vehicle treated optic nerves showed similar myelination and inflammation, but significantly decreased NMNAT2, and significantly increased SARM1 intensity as compared to sham controls. Similar to vehicle treated ONC groups, 5IIQ treatment groups did not show changes in myelination and inflammation in the optic nerves. Surprisingly, 5-IIQ treatment did not change the NMNAT2 and SARM1 levels as compared to vehicle-treated group, even though, functionally, there was a significant improvement in this group. More studies are required to understand the mechanism by which SARM1 inhibitors potentially mitigate axon damage and improve axon function. Overall, the data illuminates the potential of a SARM1 inhibitor treatment to induce neuroprotection and promote recovery in neurodegenerative diseases.

# Abstract: P01.040 – The Effect of Neonatal Intermittent Hypoxia on Brain Lipid Metabolism

**Published:** 2025-09-05

**Authors:** Regina Fernandez Fernandez, Wedad Fallatah, Yuanyuan Ji, Jace Jones, Rida Ali, Ann Moser, Michael Wolfgang, Susanna Scafidi, Joseph Scafidi

**Affiliations:** a The Michael V. Johnston Center for Developmental Neuroscience, Kennedy Krieger Institute, Baltimore, MD, USA; b Department of Neurology, Johns Hopkins University, Baltimore, MD, USA; c Hugo W. Moser Research Institute, Kennedy Krieger Institute, Baltimore, MD, USA; d Department of Pharmaceutical Sciences, University of Maryland, School of Pharmacy, Baltimore, MD, USA; e Department of Biological Chemistry, Johns Hopkins University School of Medicine, Baltimore, MD, USA; f Department of Pharmacology and Molecular Sciences, Johns Hopkins University School of Medicine, Baltimore, MD, USA; g Department of Anesthesiology and Critical Care Medicine, Johns Hopkins University, Baltimore, MD, USA

## Abstract

**Introduction:**

Neonatal brain injury due to intermittent hypoxemia (IHx) in infants born extremely preterm (EPT) is a major contributor to neurodevelopmental delays and disrupted trajectory of brain development evident on neuroimaging. The growing brain has high energy demands; however, the exact metabolic demands during the recovery period following EPT birth and IHx have not been delineated. The developing brain is characterized by extensive de novo fatty acid (FA) synthesis. These newly synthesized FAs are incorporated into complex lipids. We hypothesize that neonatal brain injury disrupts FA metabolism to meet immediate metabolic demands, therefore contributing to aberrant developmental trajectory of brain growth and maturation.

**Methods:**

We used intermittent hypoxia (IHx) as a model of neonatal brain injury from apnea of prematurity. Pups were exposed to 32-cycles of 3-min hypoxic episodes followed by 5-min of re-oxygenation daily from postnatal day (P)1 to P11. Afterward, pups recovered under normoxic conditions and were weaned from the dams at P21. Hippocampal tissue was dissected and metabolic studies were performed at P11, P17, P22, and P30.

**Results:**

Similar to children born EPT, IHx-injured mice displayed deficits in hippocampal-dependent recognition and spatial memory as evident by a poor performance on the novel object recognition and spontaneous alteration Y-maze tests. Lipid profiling using liquid and gas chromatography with tandem mass spectrometry (LC-MS/MS; GC-MS) at P30 revealed significant differences in lipid classes and total FA composition in hippocampi of mice that underwent IHx compared to age-matched control.

Proteomics analysis demonstrated that lipid metabolism was affected at P11 in mice that underwent IHx. Acyl-carnitines in the hippocampus were increased in IHx-injured mice compared to controls indicating FA mobilization for subsequent oxidation. Using the Seahorse Mito Fuel Flex test, we determined that the hippocampus showed increased dependency on FA as a fuel at P11 and P17. We validated these findings by measuring [14C]-FA oxidation to [14C]-CO_2_ in the hippocampus ex-vivo and determined that IHx significantly increased brain FA oxidation at P11 and P17. Using a conditional knockout mouse of carnitine palmitoyltransferase-2 (Cpt2), an enzyme required for FA oxidation, we showed that the loss of Cpt2 in astrocytes significantly reduced FA oxidation compared to IHx-injured wild-type mice. Moreover, we showed that acetate supplementation, not glucose, spared [14C]-FA from being oxidized in IHx-injured mice compared to controls. In agreement, [14C]-acetate oxidation increased after IHx and was not affected by FA supplementation.

**Conclusion:**

Our data show that after IHx, the brain exhibits an increased dependency on FA for fuel that persists for several days after the injury. These data also demonstrate that there is a critical window for intervention. Substrates such as acetate may serve as an alternative fuel to spare FAs from oxidation. Delineating the perturbations of FA metabolism during recovery following EPT birth will result in the development of new therapeutic interventions to improve neurological outcomes.

# Abstract: P01.041 – TRPML1 Modulates the Oligodendrocyte Cytoskeleton via Rac1

**Published:** 2025-09-05

**Authors:** Lindsay Festa, Serena Chen, Judith Grinspan, Kelly Jordan-Sciutto

**Affiliations:** a University of Pennsylvania, Philadelphia, PA, USA; b Children’s Hospital of Philadelphia, Philadelphia, PA, USA

## Abstract

Oligodendrocyte precursor cells (OPCs) and oligodendrocytes (OLs) must undergo dramatic cytoskeleton rearrangement to differentiate and produce myelin. The actin cytoskeleton mediates numerous changes in OPCs and OLs including, but not limited to, OPC migration, surveillance of local environments, and active myelin wrapping. These behaviors are driven by F-actin assembly/disassembly within a lamellipodial structure located at the tip of OL processes. Alterations in F-actin are highly sensitive to changes in intracellular Ca^2+^; however, while research has focused primarily on Ca^2+^ channels present on the plasma membrane, less is known about the role of intracellular Ca^2+^ stores, such as the lysosome, and their relationship with actin cytoskeleton dynamics. The transient receptor potential mucolipin 1 (TRPML1) is the main Ca^2+^ exporter on the lysosome and interacts with the Rho GTPase, Rac1, a key regulator of the actin cytoskeleton via activation of numerous downstream mediators, including PAK. Intriguingly, inactivating mutations of TRPML1 results in the rare neurodevelopmental disorder mucolipidosis type IV, which is characterized by white matter abnormalities and hypomyelination. Taken together, these data implicate TRPML1 in playing a critical role in oligodendrocyte maturation and myelination, though the precise mechanisms underlying these observations are unknown. Our preliminary data demonstrated that as OLs maturate, lysosomes migrate into developing processes where they are poised to influence localized Ca^2+^ signaling necessary for actin cytoskeleton changes. Therefore, we hypothesized that activation of TRPML1 is required for the actin cytoskeleton changes that underlie process extension. Utilizing our well established in vitro OPC differentiation we first assessed whether stimulation of TRPML1 altered expression of OL lineage markers and OL morphology. While we observed no changes in the number of cells expressing OPC (A2B5) or OL (GalC, PLP) markers following application of the synthetic TRPML1 agonist, MLSA1, there were striking morphological alterations, including increased OL process number, process length, and process complexity. Based on this, we next interrogated whether alterations in the actin cytoskeleton were responsible for the structural changes we observed. Activation of TRPML1 via MLSA1 resulted in a significant increase in F/G actin ratio after 3 h, indicative of a shift towards actin polymerization; in line with this, we saw an increase in phalloidin (F-actin specific peptide dye) staining intensity as early as 24 h after the switch to differentiation medium in MLSA1-treated cultures. Lastly, we demonstrated that stimulation of TRPML1 rapidly activates Rac1 and its downstream effectors PAK1, LIMK1, and cofilin, revealing a potential mechanism by which TRPML1 controls the OL actin cytoskeleton; indeed, our data demonstrates that blockade of Rac1 activation by the inhibitor NSC23766 prevents TRPML1-induced actin alterations and process outgrowth. Taken together, our work highlights a previously unknown function of TRPML1 in modulating OL function and has implications not only for homeostatic regulation but also disease states where lysosomal function is known to be dysregulated.

# Abstract: P01.042 – Modeling Phenotypic Onset in a Human iPSC Model of CLN3 Batten Disease

**Published:** 2025-09-05

**Authors:** Miss. Amelinda Firdauzy, Christina Walker, Lisa Lin, Yanping Zhu, David Vocadlo, Lisa Marie Julian

**Affiliations:** a Department of Biological Sciences, Simon Fraser University, Burnaby, Canada; b Department of Molecular Biology and Biochemistry, Simon Fraser University, Burnaby, Canada; c Department of Chemistry, Simon Fraser University, Burnaby, Canada; d Centre for Cell Biology, Development, and Disease, Simon Fraser University, Burnaby, Canada

## Abstract

**Introduction:**

Batten Disease (BD) is a rare fatal neurodegenerative disorder in children characterized by accumulation of autofluorescent lipopigment, structures containing excess cellular protein and lipid deposits that build up within lysosomes in the brain. While there are over a dozen known subtypes of BD, classified by the specific gene altered in the disease, CLN3 BD is among the most common forms. CLN3 BD presents in children around age five with symptoms including loss of vision, epileptic seizures, psychomotor retardation, and dementia, and these symptoms continue to progress in severity. CLN3-BD is caused primarily by autosomal recessive mutations in the CLN3 gene that lead to reduced or altered production of Battenin/ CLN3, a lysosome-resident protein with largely unknown function. CLN3-deficiency causes stereotypical accumulation of waste materials within lysosomes, though the underlying mechanisms are elusive. We will produce a human induced pluripotent stem cell (hiPSC)-derived neural cell model to elucidate early and progressive molecular alterations caused by CLN3 mutations derived from BD patients, focusing on phenotypes related to lysosome function and organelle processes that lead to altered metabolism.

**Methods:**

I have used six hiPSC lines, three obtained from CLN3 BD patients and three from matched familial controls, and standard protocols to induce their differentiation into cell types affected in BD patients – neural stem cells and subsequently neurons and astrocytes. The aim is to establish a robust human model of CLN3-BD progression by characterizing known phenotypes that associate with clinical presentation (lipopigment, ATP synthase-C accumulation, neuronal cell death) and potential unknown early-stage phenotypes (e.g., metabolic, lysosomal, lipid alterations and effects on neuronal maturation) in CLN3 mutant hiPSC-derived neural cell cultures. Various assays including immunofluorescence, live cell high content imaging, and Seahorse metabolic analysis will be employed.

**Results:**

Multiple early-stage phenotypes including decreased neurite length, elevated lysosome and lipid levels and reduced oxygen consumption have been observed in CLN3 BD patient-derived hiPSC-derived neural cell cultures, compared to familial controls. Moreover, through immunocytochemistry, CLN3 patient-derived neuronal-astrocyte cultures displayed elevated levels of the lysosome membrane protein LAMP1 and accumulation of neutral lipids (detected by LipidGreen cell dye) after one month of neuronal differentiation. CLN3 patient-derived neural cells also displayed impaired mitochondrial function, specifically reduced mitochondrial oxidative phosphorylation, as observed through Seahorse Mito-Stress assay.

**Conclusion:**

These results reveal that recessive CLN3 mutations in neural cells cause progressive changes in the lysosome-lipid-metabolism axis. In addition, these results have captured promising characteristics of disease progression that have not yet been robustly described (e.g., decreased neurite length and early lipid accumulation), which could represent the underlying causes or potentially serve as biomarkers of neurodegeneration in CLN3 BD. Our efforts to characterize cellular drivers of disease in our hiPSC-derived CLN3 BD model will ultimately provide improved biomarkers and therapeutic strategies for patients suffering from Batten Disease.

# Abstract: P01.043 – A Conserved Stress Response Associated With Dark Microglia Drives Neurodegeneration in Alzheimer’s Disease

**Published:** 2025-09-05

**Authors:** Anna Flury, Leen Aljayousi, Siaresh Aziz, Hye-Jin Park, Fernando González Ibáñez, Colby Sandberg, Mohammadparsa Khakpour, Damien Marechal, Thi Ngo, Dvir Avnon-Klein, Olivia Braniff, Jackson McGrath, Pragney Deme, John W. Murray, Norman J. Haughey, Patrizia Casaccia, Susana Mingote, Jia Liu, Sebastian Werneburg, Marie-Ève Tremblay, Pinar Ayata

**Affiliations:** a Neuroscience Initiative, Advanced Science Research Center, CUNY Graduate Center, New York, NY, USA; b Division of Medical Sciences, University of Victoria, Victoria, Canada; c Department of Ophthalmology and Visual Sciences, University of Michigan Kellogg Eye Center, Ann Arbor, MI, USA; d Department of Neurology, Johns Hopkins University School of Medicine, Baltimore, MD, USA; e Columbia Center for Human Development/Center for Stem Cell Therapies, Department of Medicine, Columbia University Vagelos College of Physicians and Surgeons, New York, NY, USA; f Department of Psychiatry, Johns Hopkins University School of Medicine, Baltimore, MD, USA; g Department of Molecular Medicine, Université Laval, Quebec City, Canada; h Fishberg Department of Neuroscience, Friedman Brain Institute, Ronald M. Loeb Center for Alzheimer’s Disease, Icahn School of Medicine at Mount Sinai, New York, NY, USA

## Abstract

Microglia – the brain’s immune cells – are implicated as the leading causal cell type in Alzheimer’s disease (AD). As a heterogeneous population, microglia can assume co-existing phenotypes with diverse functional outcomes. While the disease-associated microglia subset with protective functions is well characterized, the identity of microglia that contribute to neurodegeneration remains unresolved. Ultrastructural studies in humans and mice have attributed neurodegenerative properties to a “dark microglia” subset that frequently interacts with synaptic structures and manifests in great numbers in disease conditions. Dark microglia exhibit signs of cellular stress, including aberrant mitochondria and dilated Golgi and endoplasmic reticulum. In line with this, single-cell sequencing studies of human AD brains have consistently reported a microglial subset characterized by cellular stress. Therefore, investigating the idea that cellular stress may distinguish “neurodegenerative” microglia, we identified a microglial subset characterized by a conserved stress response pathway. Using two opposing microglia-specific transgenic mouse models to turn on or off this response, we show that its chemogenetic activation in microglia is sufficient for the emergence of dark microglia in the absence of other insults. Activating stress response also induces a distinct molecular profile, upregulating pathways involved in proteostasis, senescence, and metabolism. In disease models, microglial stress response exacerbates AD-associated amyloid and tau pathologies and causes dysfunction in other brain cell types. Blocking microglial stress response shifts the canonical dark microglia phenotype toward a more intermediate phenotype and improves several of these pathologies. Mechanistically, we show that upon activation of stress response, microglia secrete factors that induce the dysfunction of other brain cell types. Together, these results identify stressed microglia as a neurodegenerative phenotype and pinpoint a novel molecular mechanism of microglia-mediated neurodegeneration.

# Abstract: P01.044 – ProNGF Elicits Retrograde Axonal Degeneration of Basal Forebrain Neurons via p75NTR and Induction of APP

**Published:** 2025-09-05

**Authors:** Srestha Dasgupta, Mansi Pandya, Wilma Friedman

**Affiliations:** a Rutgers University, Newark, NJ, USA

## Abstract

Basal forebrain cholinergic neurons (BFCNs) extend long projections to multiple targets in the brain to regulate cognitive functions, and are compromised in numerous neurodegenerative disorders. Our previous study showed that moderate injury to the cortical target region of these neurons elicits retrograde degeneration in vivo. The injury promoted a significant increase in proneurotrophins in the damaged cortex, leading to the retrograde loss of BFCNs ipsilateral to the injury via the p75 neurotrophin receptor (p75NTR). We determined that direct stimulation of BFCN axon terminals with proNGF elicited retrograde degeneration of the axons leading to cell death of these neurons in vitro. Investigation of the mechanisms of axonal p75NTR signaling shows that retrograde transport and local axonal protein synthesis are necessary for proNGF induced retrograde degeneration initiated at the axon terminal. Analysis of the nascent axonal proteome revealed numerous newly synthesized proteins after stimulation of axon terminals with proNGF. Pathway analysis showed that amyloid precursor protein (APP) was a key upstream regulator. Our results show a functional role for APP in promoting proNGF induced BFCN axonal degeneration and cell death.

# Abstract: P01.045 – Therapeutic Potential of Targeting Heparan Sulfate Proteoglycan Sulfatases in an EAE Demyelination Model

**Published:** 2025-09-05

**Authors:** Farah Gadelkarim, Roopa Ravichander, Jon Frost, Rupadevi Muthaiah, Ding Xu, Fraser Sim

**Affiliations:** a University at Buffalo, Buffalo, NY, USA

## Abstract

Chronic demyelination contributes to neurological dysfunction and neurodegeneration in multiple sclerosis (MS). The inhibitory extracellular matrix (ECM) environment present in MS contributes to a failure of functional myelin repair known as remyelination. The sulfation pattern of heparan sulfate proteoglycan (HSPG) influences the paracrine signaling environment surrounding oligodendrocyte progenitor cells (OPCs). We have previously found that both the genetic loss of OPC-expressed 6-O sulfatases and systemic pharmacological inhibition of sulfatase improves remyelination following toxin-induced demyelination in adult mice. In this study, we hypothesized that systemic delivery of PI-88, a heparan sulfate mimetic capable of antagonizing sulfatase and heparanase, would promote remyelination and improve clinical outcome following immune-mediated demyelination. We generated tamoxifen-inducible NG2creER;Mapt(tau)-mGFP reporter mice to identify and quantify the generation of new oligodendrocytes and axonal ensheathment. Following injection of tamoxifen at 6-weeks age, chronic EAE-mediated demyelination was induced using MOG35-55 peptide at 9-weeks and clinical assessment performed daily thereafter. A therapeutic treatment regime of PI-88 or saline control was initiated at the peak of clinical disease, 13 days post-immunization (dpi). Results from our preliminary studies of revealed lower clinical disease scores in PI-88 treated animals compared to control animals, and preliminary histological analyses revealed a clear effect of PI-88 on the expression of highly sulfated HSPG within demyelinating lesions in the lumbar spinal cord. Using HS20 antibody to label 6-O and 2-O sulfated HSPG, PI-88 treatment was observed to substantially increase global HS20 immunofluorescence in demyelinating lesions as well as specifically around Olig2-defined oligodendrocyte lineage cells located within the lesion (*n* = 9–10/group). Alongside these changes in HSPG sulfation, we observed a substantial increase in the density of Olig2+ oligodendrocyte lineage cells and CC1+ oligodendrocytes within animals treated with PI-88. Future work will examine the precise effects of PI-88 on glial response to demyelination including the genetic assessment of new oligoneogenesis and remyelination as well as determine the PI-88 dependent effects on the peripheral immune response in EAE. We anticipate that these proof-of-concept studies will establish a strong scientific rationale for the pharmacological targeting of sulfatases in the context of human demyelinating disease

# Abstract: P01.046 – Altered Activation in the Dentate Gyrus of Seizure-Prone CACNA2D2 Knockout Mice

**Published:** 2025-09-05

**Authors:** Ashlynn Gallagher, Alyssa Danis, Ashley Anderson, Eric Schnell

**Affiliations:** a Oregon Health and Sciences University, Portland, OR, USA; b Neuroscience Graduate Program, Oregon Health and Sciences University, Portland, OR, USA; c Portland VA Healthcare System, Portland, OR, USA

## Abstract

The voltage-gated calcium channel subunit gene CACNA2D2 controls calcium-dependent signaling in neurons, and loss of this subunit causes epilepsy and ataxia in both mice and humans. Although this gene is primarily associated with cerebellar Purkinje cell function, it is also expressed in the hippocampus, and homozygous CACNA2D2 mutant mice manifest electroencephalographic spike-wave discharges (SWDs) as well as generalized tonic-clonic seizures. SWDs are typically associated with aberrant thalamocortical activation, but due to the existence of generalized seizure events, we sought to determine whether these mice manifested signs of hippocampal involvement in seizure activity. We analyzed various histopathological correlates of epilepsy in the hippocampal dentate gyrus of juvenile (21–28 do) CACNA2D2 wildtype (WT) and knockout (KO) mice, using immunohistochemical staining and confocal microscopy. Despite intermittent behavioral seizures, juvenile CACNA2D2 KO mice demonstrated no difference in expression of the activity-dependent gene cFos within the dentate granule cell layer (GCL) compared to CACNA2D2 WT mice. Interestingly, even KO mice who displayed handling-induced behavioral tonic-clonic seizures in the hour prior to sacrifice did not demonstrate increased activation of granule cells and putative semi-lunar granule cells when compared with WT littermates. As previously reported, other histopathological markers of epilepsy in these mice, including markers of altered neurogenesis (Ki67 and doublecortin), glial activation (GFAP AND Mac-2), and mossy fiber sprouting (ZnT3), as well as structural changes such as granule cell layer dispersion, were all minimally changed in juvenile mice. Overall, it does not appear that generalized seizure activity involves widespread hippocampal activation on an ongoing basis, suggesting that alternative loci might be involved in the initiation or maintenance of behavioral seizures in these mice.

# Abstract: P01.047 – Mitochondrial Functional Assays in a Canine Survival Model of Hypothermic Circulatory Arrest

**Published:** 2025-09-05

**Authors:** MD Bruno Gallo, Regina F. Fernandez, Holly L. Hammond, Noelle L. Puleo, Yuma Kitase, Tim Heck, Ifeanyi D. Chinedozi, Hannah Rando, Zachary E. Darby, Jin Kook Kang, Jessica Briscoe, Kannan Rangaramanujam, Lauren L. Jantzie, Susanna Scafidi, Jennifer S. Lawton, Joseph Scafidi

**Affiliations:** a The Michael V. Johnston Center for Developmental Neuroscience, Kennedy Krieger Institute, Baltimore, MD, USA; b Department of Neurology, Johns Hopkins University School of Medicine, Baltimore, MD, USA; c Department of Anesthesiology and Critical Care Medicine, Johns Hopkins University School of Medicine, Baltimore, MD, USA; d Department of Pediatrics, Johns Hopkins University School of Medicine, Baltimore, MD, USA; e Department of Cardiac Surgery, Johns Hopkins University School of Medicine, Baltimore, MD, USA; f Center for Nanomedicine, Johns Hopkins University School of Medicine, Baltimore, MD, USA

## Abstract

**Introduction:**

Neurological injury after cardiopulmonary bypass (CPB) surgery occurs in nearly 30% of patients and results in significant morbidity. While hypothermic circulatory arrest (HCA) offers neuroprotection during CPB, the brain remains susceptible to injury due to bioenergetic demands and mitochondrial dysfunction. To date, there is no clinically established pharmacotherapeutic intervention that protects against HCA-induced neurological dysfunction. Using an established canine model of HCA, we aimed to determine whether targeted pharmacological intervention can improve neurologic outcome by supporting bioenergetic demands during HCA.

**Method:**

Here we used an established canine survival model of HCA. Each subject was randomly assigned to one of the five drug intervention groups: (1) ketamine; (2) conjugated ketamine dendrimer; (3) diazoxide; (4) diazoxide +5-hydroxydecanoate; or (5) 0.9% saline control. All investigators were blinded to treatment throughout the entire study. Canines were labeled as belonging to either groups B,C,D,E, or F. On the day of surgery, animals were sedated, intubated, and placed on CBP via right femoral and right external jugular venous drainage and right femoral arterial cannulation with subsequent cooling to 18oC. After 90-min of HCA, the canines were rewarmed and subsequently liberated from CPB. Neurological assessments using the Finnish and Pittsburgh neurobehavioral batteries were performed at baseline (Pre-HCA), 24h, 48h, and 72h after the procedure. On the third post-operative day after HCA, heart, liver, and brain were rapidly harvested. The following brain regions were assessed: (1) sensorimotor cortex; (2) hippocampus; (3) basal ganglia; (4) and cerebellum. We measured the oxidation of the following 14 C-substrates to 14CO_2_: [U-14C]-Glucose; [U-14C]-Lactate; [U-14C]-Glutamine; [3–14C]-βHB; and [1–14C]-Oleic Acid (Fatty acid). Mitochondrial functional assays were performed using a modified Seahorse Mito Stress Test protocol. Biopsy samples were obtained in triplicate from the specific brain regions noted above and plated on a spheroid 96-well assay plate.

**Results:**

The results of the oxidation studies indicate that the brain has a higher oxidation of glucose compared to liver and heart. Specifically, the hippocampus and basal ganglia exhibited higher levels of glucose oxidation compared to the sensorimotor cortex and cerebellum. In comparing the blinded drug intervention groups, in the hippocampus, group C showed higher oxidation of all substrates compared to groups B and D.

Mitochondrial function, as measured by maximal respiration and spare respiratory capacity on the Mito Stress Test, revealed differences between brain regions as well as between groups. The basal ganglia, followed by the hippocampus and cortex had higher values of maximal respiration and spare respiratory capacity compared to the cerebellum. The drug intervention group C, E and B had higher maximal respiration and spare respiratory capacity compared to groups D and F.

**Conclusion:**

Currently, we remain blinded to drug and group assignments. However, this study is unique in that we are performing oxidation studies and mitochondrial functional assays in a large vertebrate model of extended HCA. The outcomes of this blinded study will lead to clinical trials of pharmacotherapeutic interventions that protect against HCA-induced neurological injury during CBP surgery in adults, with the aim of improving neurological outcomes.

# Abstract: P01.048 – Chemogenetic Manipulation of Astrocyte Functions During Postnatal Brain Development

**Published:** 2025-09-05

**Authors:** Karissa Garbarini, Christina Angeliu, Jazmin Corral, Veronica Cheli, Pablo Paez

**Affiliations:** a University at Buffalo, Buffalo, NY, USA

## Abstract

Astrocytes are the most abundant glial cell in the brain, and during brain development these cells regulate the migration and maturation of neurons and the formation of synapses. Additionally, astrocytes release growth factors which are central for normal oligodendrocyte development and myelination. Astrocytes express G-protein-coupled receptors (GPCRs) that modulate intracellular Ca^2+^ signaling, and ion channels activity on their membranes. Previous studies have focused on the effects of astrocytic Ca^2+^ signaling in the mature CNS, but the role of astrocytic Ca^2+^ signaling in the developing brain is still largely understudied. Via Cre-mediated recombination we expressed a GPCR in astrocytes based on the human muscarinic receptors hM3Dq. This excitatory Ca^2+^ receptor is exclusively activated by clozapine N-oxide (CNO). Prior work from our lab has shown that the activation of hM3Dq by CNO increases intracellular Ca^2+^ levels and changes the activity of several Ca^2+^ channels in cortical astrocytes. Thus, we have evaluated how these changes in astrocytes affect the maturation of neurons and oligodendrocytes during the postnatal development of the mouse brain. We have analyzed oligodendrocyte maturation and myelination in the corpus callosum and striatum, and neuronal development and synapse formation in the somatosensory cortex, motor cortex, and hippocampus. We have found that hM3Dq activation in astrocyte early during postnatal development affects the myelination of several brain areas, the density of oligodendrocytes and neurons in the cortex, and the morphology of the hippocampus. These experiments may lead to mechanistic insights of astrocytes during development and potential therapeutics for neurodevelopmental disorders.

# Abstract: P01.049 – Centella Asiatica Alters Cognition in Aged Male and Female Mice, and Anxiety and Plasma GABA and Corticosterone Only in Females

**Published:** 2025-09-05

**Authors:** Nora Gray, Wyatt Hack, Mikah Brandes, Jon Zweig, Seiji Koike, Thuan Nguyen, Jacob Raber, Joseph Quinn, Amala Soumyanath

**Affiliations:** a BENFRA Botanical Dietary Supplements Research Center, Oregon Health and Science University, Portland, OR, USA; b Department of Neurology, Oregon Health and Science University, Portland, OR, USA; c Biostatistics Design Program, Oregon Health and Science University – Portland State University School of Public Health, Portland, OR, USA; d Department of Behavioral Neuroscience, Oregon Health and Science University, Portland, OR, USA; e Department of Neurology and Parkinson’s Disease Research Education and Clinical Care Center (PADRECC) Veterans Affairs Portland Healthcare System, Portland, OR, USA

## Abstract

We have previously reported that the water extract of the Ayurvedic plant Centella asiatica (CAW) can improve cognitive deficits in mouse models of cognitive aging and neurodegenerative diseases when administered in the drinking water. Here we evaluated the effects of CAW on cognition and anxiety in aged wild-type mice and assessed whether changes in circulating levels of GABA and corticosterone were associated with those effects. Eighteen-month-old male and female C57BL6 mice were administered CAW in their drinking water (10 mg/mL) for a total of 5 weeks with behavioral testing of cognition and measures of anxiety in the fourth and fifth weeks. Results from behavioral testing were compared to 3- and 18-month old mice that did not receive any CAW. At the conclusion of testing plasma levels of corticosterone and GABA were quantified by ELISA. We found that CAW-treatment improved age-related deficits in learning, executive function and recognition memory in both sexes and attenuated the increased measures of anxiety that was only observed in aged female mice. We also observed that aged female mice had higher plasma levels of corticosterone and lower plasma levels of GABA than young female mice. These alterations were not evident in CAW-treated aged female mice where plasma levels of both analytes were no different from those in young female mice. No alterations in plasma GABA or corticosterone levels were seen in male mice regardless of age or treatment status. These results suggest that effects of CAW on measures of anxiety but not cognition are associated with changes in circulating GABA and corticosterone.

# Abstract: P01.050 – RNA Sequencing Identifies Sex-Related Differences in Transcriptional Signatures in the Female and Male Control Rats

**Published:** 2025-09-05

**Authors:** Vaibhav Gujar, Atul Daiwile, Jean Lud Cadet

**Affiliations:** a National Institute on Drug Abuse, Baltimore, MD, USA

## Abstract

Substantial evidence supports the existence of significant behavioral differences between the two sexes in animal species. These variances might be related to baseline dissimilarities in molecular and biochemical pathways in the brain. These baseline variations might account for disparate behavioral and molecular responses to rewarding substances like methamphetamine and cocaine. As a first step towards identifying sex-based differences in transcriptional signatures in the rat brain, we have used RNA sequencing to measure basal gene expression in the dorsal striatum of drug-naïve male and female rats. RNA sequencing analysis identified 1005 genes with higher and 932 genes with lower expression in female compared to male rats. The genes with higher basal expression belonged to biological domains such as membrane function, glycoproteins, neuroactive ligand-receptor interactions, and calcium ion binding. In contrast, genes with lower expression were linked to extracellular space, endoplasmic reticulum, transcription regulation, and protein folding. These observations provide further evidence for significant baseline differences in striatal gene expression between the two sexes. More studies are underway to assess to what extent rats show global sexually dimorphic responses to rewarding substances.

# Abstract: P01.051 – Transgenic Mouse Models to Define the Role of Microglia in Glioblastoma (GBM) Progression

**Published:** 2025-09-05

**Authors:** Payton Haak, Rex Gaskins, Andrew Steelman

**Affiliations:** a University of Illinois, Champaign, IL, USA

## Abstract

A hallmark of glioblastoma (GBM), the most aggressive and common brain cancer, is the tumor mass often consisting of ∼30% microglia and macrophages (TAMs), yet their precise contributions to tumor development and progression remain unknown. Recent findings show transmembrane protein 119 (TMEM119) is exclusively expressed in microglia, enabling their distinction from other myeloid populations such as macrophages. In the current study we exploited the differential expression of Tmem119 in microglia but not macrophages to create transgenic mouse lines that allow 1) microglia-specific labeling to visualize localization during disease progression, and 2) access microglia involvement in disease progression following stereotaxic injection of CT-2A glioma cells into immunocompetent syngeneic C57BL/6J mice. Flow cytometry performed on cells from tamoxifen injected Tmem119-CreERT2 x Ai14 mice revealed 85–90% of CD11b + CD45int cells in the brain and 70–80% of microglia in the spinal cord expressed tdTomato. This model allowed for a visualization of microglia (TdTomato + Iba1+) and macrophages (TdTomato-Iba1+) within the brain throughout the course of tumor development. Immunofluorescent staining of brain tissues obtained from tamoxifen treated-Tmem119- CreERT2 x Csf1rfl/fl (MGCKO) revealed an approximate 70% reduction in Iba1+ microglia within the cortex, hippocampus, and striatum when compared to Csf1rfl/fl control mice. Following microglia depletion, tumor-bearing mice experienced prolonged survival (log-rank *p* = 0.005) and decreased weight loss compared to mice harboring normal microglia populations. Collectively, we developed mouse models that provide immunofluorescent labeling and depletion of microglia, enabling greater understanding of this cell type’s role in GBM pathogenesis.

# Abstract: P01.052 – Delayed Phospho-BTK Inhibition Reduces the Reactive Phenotype of Microglia and Oligodendrocytes and Improves Myelin Structure After Stroke in Neonatal Mice

**Published:** 2025-09-05

**Authors:** Elisabeth Harmon, Danae Mitchell, Macy Falk, Andra Dingman

**Affiliations:** a Department of Pediatrics, Division of Child Neurology, University of Colorado Anschutz Medical Campus, Aurora, CO, USA; b Graduate Medical Education, University of Colorado Anschutz Medical Campus, Aurora, CO, USA; c Department of Anesthesiology, University of Colorado Anschutz Medical Campus, Aurora, CO, USA

## Abstract

**Background:**

Arterial ischemic stroke in the perinatal period is common and results in life-long deficits. Most neonates are not diagnosed until days to months after stroke has occurred, and so acute neuroprotection is not a viable treatment. We use 60 min of transient middle cerebral artery occlusion (MCAO) in postnatal day 10 (p10) mice to model neonatal stroke. Our published data demonstrates persistence of disease associated oligodendrocytes (DOLs) with an inflammatory gene expression profile and myelin structural abnormalities in striatal white matter two weeks after p10 MCAO. We hypothesize that microglia influence oligodendrocyte and white matter phenotype at late timepoints after p10 MCAO.

**Objective:**

To investigate whether delayed treatment with the Bruton’s Tyrosine Kinase (BTK) inhibitor, Ibrutinib, reduces microglia reactivity, reduces DOLs, and improves white matter (WM) myelin structure after p10 MCAO in mice.

**Methods:**

IHC for phospho-BTK and glial cell specific markers was used to assess phospho-BTK expression in microglia, astrocytes, and oligodendrocytes. Mice were treated with the Bruton’s tyrosine kinase inhibitor, Ibrutinib (1 mg/kg/day), or vehicle on days 7–12 post-MCAO and were sacrificed on post-MCAO day 14. IHC was used to evaluate microglia phenotype and fluorescent in-situ hybridization (FISH) was used to quantify DOLs. Nile red myelin spectroscopy was used to assess white matter structure.

**Results:**

In ipsilateral striatum, Phospho-BTK colocalization was significantly greater in microglia (0.48 ± 0.15, ratio overlap with Iba1), compared to astrocytes (0.13 ± 0.10, ratio overlap with GFAP, *p* = 0.006) and oligodendrocytes (0.15 ± 0.04, ratio overlap with BCAS1, *p* = 0.001, *n* = 5, one-way ANOVA). Iba + cells in vehicle treated mice had decreased expression of the homeostatic marker TMEM119 in ipsilateral striatum compared to contralateral (ipsi TMEM119 mean intensity in Iba + cells: 758.7 ± 361.96 vs contra: 1296.53 ± 131.13, *p* = 0.03, *n* = 5). In Ibrutinib treated mice TMEM119 expression was similar between ipsilateral and contralateral striatum (ipsi:1028 ± 189.26 vs contra: 1188.32 ± 109.93, *p* = 0.65, *n* = 4, two-way ANOVA). The proportion of Sox10 expressing cells that also expressed the DOL markers B2m and Neat1 was significantly increased in ipsilateral striatum compared to contralateral in vehicle treated animals (ipsi: 0.40 ± 0.13 vs contra: 0.11 ± 0.06 Neat1 + B2m + Sox10+/Sox10+, *p* = 0.003, *n* = 5). In Ibrutinib treated animals the proportion of oligodendrocytes with a DOL phenotype was similar between ipsilateral and contralateral hemispheres (ipsi: 0.21 ± 0.15 vs contra 0.14 ± 0.02 Neat1 + B2m + Sox10+/Sox10+, *p* = 0.50, *n* = 4). The proportion of DOLs was significantly less in ipsilateral striatum in Ibrutinib treated animals compared to vehicle (*p* = 0.03, two-way ANOVA). Finally, spectral analysis of striatal white matter showed that myelin from ipsilateral striatum in vehicle treated animals had significantly higher polarity index (indicative of less mature myelin with lower cholesterol content) compared to contralateral (ipsi polarity index (PI): 0.33 ± 0.13 vs contra: 0.17 ± 03, *p* = 0.03), but PI was similar in Ibrutinib treated mice (ipsi PI: 0.19 ± 0.04 vs contra 0.17 ± 0.05, *p* = 0.94) and PI was significantly lower in ipsilateral myelin in Ibrutinib treated animal compared to vehicle (*p* = 0.03, two-way ANOVA).

**Conclusions:**

Delayed treatment with Ibrutinib results in more homeostatic microglia phenotype, reduces stroke disease associated oligodendrocytes, and improves white matter structure two-weeks after p10 MCAO. These data suggest that targeting sub-acute microglia reactivity may improve white matter development after neonatal stroke.

# Abstract: P01.053 – Characterizing Microglial Dynamics During White Matter Ischemic Injury: An Age and Sex Dependent Response

**Published:** 2025-09-05

**Authors:** Isabela Harmon, Eli Stabler, Selin Dogan, Hung Nguyen, Selva Baltan

**Affiliations:** a OHSU, Portland, OR, USA

## Abstract

Stroke remains the second leading cause of death and the third leading cause of disability worldwide. Major neurological disability from ischemic stroke is due to white matter injury (WMI), which can vary by age and sex. In the gray matter of the brain, microglia play a dual role after ischemic injury based on an assumed morphology: they exacerbate injury or promote neuroprotection. However, the role of microglia in WM is inconclusive, and the question of whether microglia are detrimental or beneficial towards ischemic recovery remains understudied. WM functional recovery is sex and age-specific, as young female mouse axons achieve greater functional recovery than young male mouse axons, yet this difference is reversed in aging mice. Hence, in this study, we aim to define the microglial dynamics in WMI by characterizing the morphological characteristics and ischemic response of microglia as a function of age and sex. We used isolated mouse optic nerves (MONs), a pure WM tract, from 3-month-old and 24-month-old male and female Cx3Cr1-GFP mice to capture the behavior and morphological changes of green fluorescent microglia during an in vitro ischemic model. Both optic nerves from each animal were placed in a Cell MicroControls chamber and fully submerged in oxygenated artificial cerebrospinal fluid (37 °C, 2 mL/minute). The imaging area was chosen approximately 1 mm from the optic chiasm for locational consistency and contained 5–7 microglia with visible processes and movement. Live cell images (11 z-stack) were captured using a silicone 40x objective (NA1.25) for 5 min every 20 min during baseline, 1 h of oxygen-glucose deprivation (OGD), and 1 h of recovery. Images were processed and assigned scores for eight morphological characteristics using ImageJ (developed by NIH) with the fractal analysis (FracLac) plugins. Microglial characteristics were separated by sex and age, run through principal component analysis (PCA), and grouped by both Euclidean dendrogram and cluster mapping data analysis in R coding language. The PCA grouping defined three microglial clusters that when contextualized by imaging and FracLac data depicted activated (increased in cell body and processes volume and size), unresponsive (low change in size and volume), and dying morphologies (shrinkage in size and volume) during OGD. Of the young male (YM) microglia, 10% were activated, 41% were unresponsive, and 49% were dying. For aged male (AM) microglia, 25% were activated, 36% were unresponsive, and 39% were dying. YM-activated and dying microglia had greater structural variance between time points than the AM microglia. Quantification (two-way ANOVA) of the data showed that the prominent dynamic range changes observed in YM microglia were significantly dampened in AM microglia. Our analysis of young and aging female microglial dynamics is in process. We highlighted an unbiased method to define microglia behavioral subtypes during acute OGD in WM. Our findings indicate microglia vary in resiliency, performance, and recruitment time when subjected to acute OGD, implicating a novel route of mechanistic investigation into activation factors and phenotypic polarization as a function of age and sex.

# Abstract: P01.054 – Effects of Neonatal Ethanol Exposure on Prefrontal Cortex Astrocyte Gene Expression In Vivo

**Published:** 2025-09-05

**Authors:** Joel Hashimoto, Caterina Santelli, Nicholas Margolies, Marina Guizzetti

**Affiliations:** a Oregon Health and Science University, Portland, OR, USA; b VA Portland Heath Care System, Portland, OR, USA

## Abstract

The importance of astrocytes in neurodevelopmental disorders, including the effects of developmental exposure to ethanol, has been highlighted in recent studies with increased focus on the diverse functions of astrocytes. Astrocytes express and release numerous factors that contribute to neuronal development; for instance, they are the primary producers of the brain extracellular matrix providing structural support and crucial pathfinding cues to developing neurons which may be altered by environmental insults, including ethanol. Astrocytes, upon exposure to noxious stimuli, can also release neuroimmune molecules. Utilizing a model of third-trimester equivalent exposure to intoxicating levels of ethanol we have previously characterized the effects of developmental ethanol vapor exposure on hippocampal (HIP) astrocyte translation using the translating ribosome affinity purification (TRAP) procedure. Here we expand on these studies to examine the effects of neonatal ethanol exposure on prefrontal cortex (PFC) astrocytes in vivo using the TRAP procedure. Litters of Aldh1l1-EGFP/Rpl10a mice were placed in an ethanol vapor chamber or in a control chamber for 4 h a day from postnatal day 2 (PD2) to PD7. Immediately following the ethanol or control exposure on PD7, pups were euthanized and brains were dissected. Following the TRAP procedure, we analyzed mRNA in the pull-down fractions, containing astrocyte-specific translated RNA, as well as total PFC RNA from the input fractions. We identified 557 ethanol regulated genes in the astrocyte translating RNA. In the input fraction we identified 412 ethanol regulated genes. Of these, 98 genes were ethanol regulated in both fractions and 429 genes were regulated by ethanol selectively in astrocytes. Gene Ontology (GO) enrichment analysis of ethanol-regulated genes identified translation, ribosomal proteins, and neuroimmune-related genes as the main GO categories enriched in the astrocyte translating RNA altered by ethanol exposure. In the input fraction, the main GO categories were PDGF signaling, BDNF signaling, and lipoprotein biosynthesis related genes. Comparing the results in the PFC to our previous analysis of HIP astrocyte translation showed 87 genes regulated by ethanol vapor in both the PFC and HIP. Most of the genes that were dysregulated by ethanol were in the same direction in the PFC and HIP, however 15 genes were regulated in opposite direction suggesting some brain region specific effects of ethanol on astrocyte gene translation. Our results show that developmental ethanol exposure alters gene expression in both cell-type and brain region specific manners. Furthermore, studying astrocyte-specific effects in models of Fetal Alcohol Spectrum Disorders has the potential to unmask novel mechanisms for the dysregulation of astrocyte function altering normal neuronal development. This work was supported by VA Merit Review Award I01BX001819 and NIH/NIAAA R01AA029486, P60AA010760, and U01AA029965.

# Abstract: P01.055 – Receptor-Mediated Activation of G12/13 Signaling in POMC Neurons Regulates Key Metabolic Functions

**Published:** 2025-09-05

**Authors:** Dhanush Haspula, Zhenzhong Cui, Srinivas Pittala, Yinghong Cui, Oksana Gavrilova, Asuka Inoue, Jürgen Wess

**Affiliations:** a Molecular Signaling Section, Laboratory of Bioorganic Chemistry, National Institute of Diabetes and Digestive and Kidney Diseases, Bethesda, MD, USA; b Mouse Metabolism Core, National Institute of Diabetes and Digestive and Kidney Diseases, Bethesda, MD, USA; c Graduate School of Pharmaceutical Sciences, Tohoku University, Sendai, Japan

## Abstract

Pro-opiomelanocortin (POMC) neurons in the arcuate nucleus (ARC) of the hypothalamus and nucleus tractus solitarius (NTS) of the brainstem exert a multi-tier control over energy homeostasis, which involves appetite regulation and autonomic control of metabolic tissues. Stimulation of these neurons leads to the release of alpha-melanocyte-stimulating hormone (α-MSH), which exerts an anorexigenic effect by binding to its target receptors (e.g., melanocortin-4 receptor). POMC neurons express many G-protein coupled receptors which detect changes in hormone and metabolite levels. Several of these receptors activate G proteins of the G12/13 family, besides other classes of heterotrimeric G proteins. The potential metabolic relevance of G12/13 signaling in POMC neurons remains unknown at present. To address this question, we generated mice expressing a G12/13-coupled designer receptor (G12/13 DREADD; G12/13D) specifically in POMC neurons (POMC-G12/13D mice). These mice express the G12/13D receptor in all POMC neurons in the brain. Additionally, we selectively expressed G12/13D in POMC neurons of either the ARC (POMC-ARC-G12/13D) or the NTS (POMC-NTS-G12/13D). Finally, we generated mice lacking the α-subunits of G12/13 in all POMC neurons (POMC-G12/13 KO). Deschloroclozapine (DCZ), a highly selective agonist for G12/13D which is otherwise pharmacologically inert, was administered via the drinking water for up to 8 weeks to G12/13D mice and control littermates. G12/13 activation in POMC-G12/13D mice led to significant metabolic improvements including improved glucose tolerance and elevated plasma adiponectin levels. Furthermore, G12/13 activation in POMC neurons potentiated the effects of leptin on satiety and weight loss. In vitro studies with a POMC-expressing neuronal cell line showed that G12/13D can act alone, or synergistically with the leptin receptor, in activating the JAK2-STAT3 pathway. Like POMC-G12/13D mice, both POMC-ARC-G12/13D and POMC-NTS-G12/13D mice showed improved glucose tolerance following G12/13 activation. However, plasma adiponectin levels were elevated in POMC-NTS-G12/13D mice only, consistent with previous findings that white adipose tissue receives sympathetic input from NTS projections. In contrast to G12/13 activation in POMC neurons, lack of G12/13 signaling in POMC neurons of POMC-G12/13 KO mice resulted in increased weight gain, adiposity and impaired insulin sensitivity. Finally, we showed that lorcaserin-induced activation of endogenous serotonin 5-HT2C receptors led to reduced food intake and blood glucose levels and that these effects were absent in POMC-G12/13 KO mice, indicative of the key role of G12/13 signaling in mediating the anorectic action of lorcaserin. In vitro experiments with POMC neurons further confirmed that lorcaserin-mediated activation of the Rho-ROCK pathway resulted in α-MSH release. Collectively, our data indicate a critical role for G12/13 signaling in POMC neurons in the maintenance of glucose and energy homeostasis. Mechanistic studies further revealed that the signaling pathways stimulated by G12/13 signaling in POMC neurons are similar to those activated by leptin. Furthermore, we identified a novel mechanism by which 5-HT2C receptors regulate appetite and glucose homeostasis, involving the activation of G12/13 signaling in POMC neurons. We conclude that targeting endogenous G12/13-coupled receptors expressed in POMC neurons may prove useful to improve metabolic deficits present in various pathophysiological conditions, including obesity and type 2 diabetes.

# Abstract: P01.056 – What Regulates the Morphogenesis of Astrocytes?

**Published:** 2025-09-05

**Authors:** Dongeun Heo, Alexandra Larson, Matthew LaBella, Kelly Monk, Marc Freeman

**Affiliations:** a Vollum Institute, OHSU, Portland, OR, USA; b Baylor College of Medicine, Houston, TX, USA; c Myriad Genetics, Salt Lake City, UT, USA

## Abstract

Astrocytes are evolutionarily conserved glial cells of the central nervous system (CNS) that exhibit remarkably complex, highly ramified morphology. With such morphological complexity, astrocytes perform a diverse array of functions critical for CNS physiology, including the regulation of synaptic transmission and neurovascular coupling. During development, astrocytes progressively increase their process complexity and territorial volume by growing competitively to occupy non-overlapping domains. This tiling behavior further endows astrocytes to closely associate with all neural structures, including millions of neuronal synapses, all throughout the CNS. Although the complex morphology of astrocytes has been reported more than a century ago, we still only know a handful of genes that regulate astrocyte morphogenesis and tiling. Further, we do not yet clearly know what happens to CNS function when astrocyte morphology and tiling behavior are abnormal. To uncover novel regulators of astrocyte development and cellular behavior, I performed an unbiased, forward-genetic screen in Drosophila. I used the mosaic analysis with a repressible cell marker (MARCM) system, which allows a clonal genetic manipulation of astrocytes, to 1) address developmental competitive growth and tiling and 2) study potentially lethal genes that may be important for the cellular development of astrocytes. From the initial screen, I found a total of 62 putative genetic hits, which include genes that 1) regulate the actin cytoskeleton, 2) are involved in the ubiquitin ligase pathway, and 3) have previously been studied in the context of axon degeneration. Going forward, I will genetically target these pathways in zebrafish to perform longitudinal in vivo imaging of astrocytes from birth to maturation and test whether their roles in astrocyte morphogenesis are evolutionarily conserved in vertebrates. We will also determine how disruption in astrocyte morphology and tiling may affect neuronal circuit development and function.

# Abstract: P01.057 – Examining the Role of αVCAM-1 in Attenuating the Neuroinflammatory Response in High-Fat Diet Mice After Stroke

**Published:** 2025-09-05

**Authors:** John Holsten, Kimberly Prescott, Laura Brock, Ross Brimberry, Faith Bullock, Elan Doyle, Stacie Beuschel, Todd Peterson

**Affiliations:** a University of North Carolina Wilmington, Wilmington, NC, USA

## Abstract

People with obesity are twice as likely to experience an ischemic stroke, which is driven by factors such as hypertension, low-grade chronic inflammation, and cardiovascular disease. Following a stroke, obesity contributes to secondary injury processes, including neuroinflammation. In stroke, the neuroinflammation is characterized by peripheral infiltrates, and activation of glial cells, which can lead to an increased expansion of infarct area. Previously, we demonstrated an increase in pro-inflammatory genes, a four-fold increase in macrophage response, elevated vascular cellular adhesion molecule-1 (VCAM-1), larger expansion of infarct volume, and worsened functional recovery in high-fat diet (HFD) fed animals after stroke. Previous studies have shown that blocking VCAM-1 with an antibody (αVCAM-1) attenuates the activation of glial cells and infiltration of macrophages in Alzheimer’s Disease. Thus, the current study aimed to investigate whether blocking the effects of VCAM-1 could reduce the macrophage infiltration that contribute to the exacerbated neuroinflammatory response in HFD mice after stroke. We used female mice that were given access to either a 10% kCal diet or 60% kCal high-fat diet for six weeks. All mice underwent distal Medial Cerebral Artery Occlusion (dMCAO) stroke induction. Mice were administered with 9 mg/kg of αVCAM-1 or IgG (control) at 4- and 72 hr post-stroke. To assess the alterations in the neuroinflammatory response, we measured cell coverage of astrocyte (GFAP) and microglia/macrophage (CD68) as well as an analysis of infarct expansion (NeuN). Additionally, we assessed the efficacy of αVCAM-1 treatment on motor function recovery by inducing a Photothrombotic stroke (PTS) over the right motor cortex. We assessed the fine and gross motor function both prior to and 1-, 3-, 7-, 14-, and 28 d post-stroke. Results confirmed increased plasma levels of VCAM-1 after stroke in HFD-fed animals compared to control diet (*p* < 0.05), which was reduced following αVCAM-1 administration. HFD mice exhibited significantly increased astrocyte (*p* < 0.01) and macrophage coverage (*p* < 0.001) post-stroke, coinciding with infarct expansion (*p* < 0.01) compared to mice on the control diet. When HFD mice were treated with αVCAM-1, astrocyte (*p* < 0.05) and macrophage coverage (*p* < 0.001), as well as infarct expansion (*p* < 0.001) were significantly attenuated compared to HFD mice given the control treatment. Furthermore, HFD mice treated with αVCAM-1 exhibited improved motor functional recovery (i.e., increased distance traveled on a rotating beam) compared HFD mice treated with the control treatment (*p* < 0.001). These findings suggest that αVCAM-1 is a potential treatment option for reducing neuroinflammation, infarct expansion, and improving functional recovery in the obese population, who are at higher risk for stroke and worsened outcomes. The implications of αVCAM-1 may extend beyond stroke, potentially generalizing to other neurological conditions where neuroinflammation and infiltration of macrophages play a significant role in pathology.

# Abstract: P01.058 – Elucidating Downstream Pathways of the Soluble Amyloid Precursor Protein and GABAB Receptor Interaction

**Published:** 2025-09-05

**Authors:** Samah Houmam, Dylan Barber, Charles Lacy, Kriti Shukla, Heather Rice

**Affiliations:** a Aging and Metabolism Program, Oklahoma Medical Research Foundation, Oklahoma City, OK, USA; b Oklahoma Center for Geroscience and Healthy Brain Aging, University of Oklahoma Health Sciences Center, Oklahoma City, OK, USA; c Department of Biochemistry and Molecular Biology, University of Oklahoma Health Sciences Center, Oklahoma City, OK, USA; d Neuroscience Program, University of Oklahoma Health Sciences Center, Oklahoma City, OK, USA; e Department of Chemistry and Biochemistry, University of Oklahoma, Norman, OK, USA

## Abstract

The amyloid precursor protein (APP) is a type 1 transmembrane protein that can undergo proteolytic processing to shed its ectodomain, soluble APP (sAPP). It is thought that sAPP mediates the physiological functions of APP through protein-protein interactions. To identify potential interactors of sAPP, Rice et al. (Science, 2019) performed a proteomic screen and identified the GABAB receptor (GABABR). They showed that sAPP can bind and interact with GABABR and that this interaction is able to reduce the probability of presynaptic vesicle release in hippocampal neurons. However, the pathways downstream of this interaction are yet to be uncovered. GABABR is a G-protein-coupled receptor that signals through G-proteins Gαi and Gβγ. Gαi can inhibit adenylyl cyclase thereby reducing intracellular cyclic AMP (cAMP) formation. To test whether sAPP utilizes Gαi downstream of GABABR, I used an immunoassay to measure cAMP concentrations in live hippocampal cell suspensions. My preliminary data shows that similar to a GABABR agonist (Baclofen), sAPP decreases cAMP concentrations compared to baseline control levels. This data suggests that sAPP activates canonical pathways downstream of GABABR in mouse hippocampal cells. In future experiments, I will investigate the GABABR-dependency of this effect by measuring cAMP responses to sAPP in the presence of GABABR antagonists and in GABABR knock-out cells. These results are uncovering a molecular mechanism by which the ectodomain of APP signals in neurons through GABABR under physiological conditions. This lays a foundation that the field can utilize in understanding the role of APP and its ectodomain in pathological conditions such as Alzheimer’s Disease.

# Abstract: P01.059 – PAK2 Is Necessary for Myelination in the Peripheral Nerve System

**Published:** 2025-09-05

**Authors:** Bo Hu, Daniel Moiseev, Isabella Schena, Bulat Faezov, Roland Dunbrack, Jonathan Chernoff, Bo Hu

**Affiliations:** a Houston Methodist Hospital, Houston, TX, USA; b School of Medicine, Wayne State University, Detroit, USA; c Fox Chase Cancer Center, Philadelphia, PA, USA

## Abstract

**Objective:**

Myelination enables electrical impulses to propagate on axons at the highest speed, encoding essential life functions. The Rho family GTPases, RAC1 and CDC42, have been shown to critically regulate Schwann cell myelination. P21-activated kinase 2 (PAK2) is an effector of RAC1/CDC42, but its specific role in myelination remains undetermined.

**Methods:**

We produced a Schwann cell-specific knockout mouse of Pak2 (scPak2−/−) to evaluate PAK2’s role in myelination.

**Results:**

Deletion of Pak2 specifically in mouse Schwann cells resulted in severe hypomyelination, slowed nerve conduction velocity, and behavior dysfunctions in the scPak2−/− peripheral nerve. Many Schwann cells in scPak2−/−sciatic nerves were arrested at the stage of axonal sorting. These abnormalities were rescued by reintroducing Pak2, but not the kinase-dead mutation of Pak2, via lentivirus delivery to scPak2−/− Schwann cells in vivo. Moreover, ablation of Pak2 in Schwann cells blocked the promyelinating effect driven by neuregulin-1, prion protein, and inactivated RAC1/CDC42. Conversely, the ablation of Pak2 in neurons exhibited no phenotype. Such PAK2 activity can also be either enhanced or inhibited by different myelin lipids.

**Conclusion:**

We have identified a novel promyelinating factor, PAK2, that acts as a critical convergence point for multiple promyelinating signaling pathways. The promyelination by PAK2 is Schwann cell-autonomous. Myelin lipids, identified as inhibitors or activators of PAK2, may be utilized to develop therapies for repairing abnormal myelin in peripheral neuropathies.

# Abstract: P01.060 – The Role of Inflammatory Oligodendrocyte Lineage Cells in CNS Demyelination

**Published:** 2025-09-05

**Authors:** Jingwen Hu, Matthew Smith, Riley Catenacci, Aayush Pokharel, Krista Solem, Annatje Clark, Peter Calabresi

**Affiliations:** a Johns Hopkins Medicine, Baltimore, MD, USA

## Abstract

Multiple sclerosis (MS) is a chronic inflammatory demyelinating disease in which the central nervous system (CNS) myelin is targeted by the immune system. Oligodendrocyte lineage cells (OLCs) that express major histocompatibility complex (MHC) molecules and can process and present antigen to T cells have been found in animal models of MS. Similar transcriptional feature has also been observed in human postmortem MS tissue. We recently developed TdTomato reporter lines for MHC class I (B2M) and MHC class II (CD74) that allow tracking the MHC-expressing inflammatory oligodendrocyte lineage cells (iOLCs) present in the CNS of mice with inflammation. Using an animal model of MS, in which demyelination is induced by cuprizone followed by adoptive transfer of myelin reactive T cells, we found a proportion of the OLCs start to express MHC molecules following autoimmune-induced demyelination. In order to explore the function of MHC molecules on iOLCs, we conditionally knocked out MHC class I from oligodendrocyte precursor cells (OPCs) before introducing myelin reactive T cells using PDGFR-CreER; H2Kb-flox/flox mice. In initial experiments, we did not see a significant change of the behavioral score of mice in this animal model. The result suggests inhibiting the formation of MHC I + iOLCs did not influence the inflammatory axonal dysfunction. Examining if the number of OLCs or iOLCs is different, as well as the integrity of myelin is needed to further explore the impact of MHC conditional knockout in this system. Our study on immune-related gene expression profile found on OLCs may explain how CD8 T cells are activated in MS and EAE, and could bring a new understanding of the progression of the disease.

# Abstract: P01.061 – Exocytosis of ATP in Astrocytes Regulates Amyloid-Beta Pathology

**Published:** 2025-09-05

**Authors:** Qian Huang, Hiu Ham Lee, Sohyun Moon, Addison Li, Zoya Ramzan, Min Seong Kim, Shan Jin, Matthew Jiang, Jerry Y. Zhao, Weikang Cai

**Affiliations:** a New York Institute of Technology College of osteopathic medicine, Westbury, NY, USA

## Abstract

Alzheimer’s disease (AD) is a devastating neurodegenerative disease with no cure. It is characterized by β-amyloid (Aβ)-containing senile plaques and tau-containing neurofibrillary tangles in the brain. Excessive deposition of toxic Aβ peptides in the brain is believed one of the key mechanisms contributing to AD. A growing body of evidence has demonstrated that dysregulation of astrocyte functions and astrocytic gliotransmitter release is involved in Aβ pathology. Here, we aim to investigate the potential role of astrocyte-derived purinergic signaling in the progression of AD. Aβ42 induced adenosine triphosphate (ATP) release in primary cultured astrocytes. Further, 2-Me-SATP (an ATP analog) triggered a largely overlapping transcriptional response in astrocytes comparable to those treated with Aβ42, exemplified by the induction of inflammation, suppression of pathways involved in extracellular matrix, and regulation on phagocytosis. These data strongly suggest that in response to Aβ42 exposure, astrocytes may release ATP to trigger functional alterations in astrocytes, related to inflammatory response, extracellular protein production and phagocytosis. To further test this hypothesis, we developed a unique transgenic mouse model to specifically target ATP exocytosis in astrocytes. Thus, we crossed astrocyte-specific Aldh1l1-CreERT2 mice with transgenic mice carrying floxed cassette flanking the exon 1 of Slc17a9 (Vnut) gene, which encodes the vesicular nucleotide transporter essential for loading cytosolic ATP into the secretory vesicles. Loss of Vnut in astrocytes effectively reduced vesicular ATP loading and release by ∼50% without any major alterations in total intracellular ATP content, nor major SNARE complex proteins responsible for cellular exocytosis. Notably, loss of Vnut significantly increased the uptake of HiLyte647-conjugated Aβ42 by primary astrocytes likely due to the increased activity of receptor-independent endocytosis/phagocytosis. In agreement with the KO cell model, overexpression of Vnut inhibits Aβ42 uptake by astrocytes. To further examine the role of astrocytic Vnut in AD pathology in vivo, we crossed the VnutAldh1l1KO mice with the 5xFAD mice. Consistent with our data showing increased Aβ42 uptake in VnutKO astrocytes, loss of Vnut in astrocytes of the female 5xFAD mice dramatically reduced Aβ plaques by ∼50% at 6 months of age. These alterations were most prominent in selective brain regions, including the prefrontal and motor cortex, lateral septum, and subiculum of the hippocampal formation. The induction of Gfap expression was greatly blunted in VnutAldh1l1KO/5xFAD mice, accompanied by a normalized expression of key cytokines implicated in neuroinflammatory conditions in the brain. More importantly, loss of astrocytic Vnut greatly improved cognitive deficit in the female mice with 5xFAD background. Together, our results suggest that Vnut-mediated vesicle storage and release of ATP is an important mechanism in astrocytes to regulate astrogliosis, neuroinflammation, and Aβ pathology. Inhibiting astrocytic Vnut and astrocytic-derived purinergic signaling could represent a unique and novel glial-based therapeutic strategy for AD.

# Abstract: P01.062 – Investigating the Utility of iPSC-Derived Choroid Plexus Organoids to Yield Fluid Biomarkers in SCA1 Disease Modeling

**Published:** 2025-09-05

**Authors:** Negin Imani Farahani, Lisa Julian

**Affiliations:** a Department of Biological Sciences, Simon Fraser University, Burnaby, Canada; b Centre for Cell Biology, Development, and Disease, Simon Fraser University, Burnaby, Canada; c Institute for Neuroscience and Neurotechnology, Simon Fraser University, Burnaby, Canada

## Abstract

**Introduction:**

Spinocerebellar ataxias (SCAs) are a group of hereditary, cerebellar atrophy disorders that involve the progressive loss of motor control and coordination, resulting in a 10–20 year life expectancy after the onset of clinical symptoms. Many SCA subtypes exist, with SCA1, SCA2, SCA3 and SCA6 being the most prevalent forms of adult-onset inherited ataxia in Canada and the United States. In this study, we are investigating SCA1, a SCA subtype that presents a more complex degeneration pattern in cortical and subcortical brain regions in addition to the cerebellum. No curative therapeutic interventions to slow the progression of symptoms that SCA patients face exist, in part due to the knowledge gap regarding the cellular mechanisms at work during the pathogenesis and progression of this disease. Amongst the patient community, cerebral spinal fluid (CSF) biomarker analysis is a priority, however current methods for extracting human CSF (hCSF) from patients are invasive and inconvenient.

**Objectives:**

We are developing a platform using our induced human pluripotent stem cell (hPSC) lines derived from patients with SCA1, and wild-type control lines derived from unaffected family members, to generate choroid plexus (ChP) organoids. iPSC-derived ChP organoids display key markers and cell types of the human ChP and produce fluid-filled cysts of highly similar composition to that of hCSF. We aim to evaluate the efficacy of using these patient-derived ChP organoids to yield fluid-based biomarkers that can provide an alternative to sampling hCSF from patients.

**Methods and Results:**

Through immunofluorescent staining of key markers of the ChP such as CLIC6, TTR and Aqp1, we ensure that the organoids are maturing and contain the expected, relevant cell types. After 3 months of ChP development, we harvest the hCSF-like fluid from the ChP organoids and perform mass-spectrometry proteomic analysis, western blots and ELISA to assess for presence of metabolic signatures and proteins that are indicative of the SCA1 disease state, such as neurofilament light chain, tau and inflammatory cytokines. These CSF-resident factors act as key indicators of synaptic dysfunction and of a disease state in neurodegenerative disorders. This work provides a link to translational clinical applications; future work will involve comparing results from organoid-derived hCSF-like fluid to hCSF from SCA patients to determine if these biomarkers can be used to track stage-specific disease progression.

**Conclusion:**

Determining if the hCSF-like fluid collected from ChP organoids can be used as a meaningful surrogate for patient-derived hCSF for biomarker analysis is a crucial first step for disease diagnoses and tracking without the use of invasive procedures like lumbar punctures, addressing this specific priority of the ataxia patient community.

# Abstract: P01.063 – Parabrachial Extended Amygdala Circuit Activity Is Heightened Following Repeated Stress

**Published:** 2025-09-05

**Authors:** Danny Winder, Nicholas Petersen, Anel Jaramillo

**Affiliations:** a University of Kentucky, Lexington, KY, USA; b Vanderbilt University, Nashville, TN, USA

## Abstract

The bed nucleus of the stria terminalis (BNST), a region critical for affective behavior. Lateral parabrachial nucleus (LPBN) projections expressing calcitonin gene related protein (CGRP, Calca gene) drive bed nucleus of the stria terminalis (BNST) in vivo activity in synchrony with anxiety-like behavior. We hypothesize an anxiogenic role for LPBN(CGRP) following stress, associated with heightened BNST activity.

To investigate the role of LPBN(CGRP) activation on stress-induced anxiety and BNST activity, CalcaCRE male mice (*n* = 3/exposure/treatment) received bilateral injections of CRE-dependent hM3D(Gq) DREADDs in the LPBN and the calcium indicator GcAMP7 in the BNST. Following 4 days of repeated forced swim test stress paired with LPBN(CGRP) hM3D(Gq) activation, anxiety-like behavior was measured with NSFT. To identify cell-specific changes in LPBN(CGRP) to BNST neurotransmission BNST-containing brain slices were bath applied CNO (10uM) and changes in GCaMP fluorescence were measured with slice photometry recordings. Post hoc thick slice immunohistochemistry was performed to characterize LPBN(CGRP) projections innervating BNST cells.

Ex vivo slice recordings demonstrate LPBN(CGRP) activation decreased global BNST spike frequency through recruitment of inhibitory and excitatory neuronal populations. This LPBN(CGRP)-induced decrease in BNST activity was potentiated in FSS relative to no stress controls (3-way ANOVA, wash, stress, treatment *p* < 0.02). Post hoc analysis demonstrates colocalization of pituitary adenylate cyclase activating (PACAP)-expressing LPBN(CGRP) projections in the BNST.

These data demonstrate LPBN(CGRP) modulates stress and anxiety-like behavioral responses associated, in part, with heightened BNST activity. Additionally, the data demonstrate a history of repeated LPBN CGRP neuron inhibition is anxiogenic in prolonged abstinence suggesting long lasting LPBN(CGRP)-induced changes in behavior. Given pharmaceutical treatments for migraines targeting CGRP inhibition are bioavailable, these studies inform a potential role for CGRP treatments in anxiety.

# Abstract: P01.064 – How Do Glia Regulate Synapse Development?

**Published:** 2025-09-05

**Authors:** Taylor Jay, Victor Ouellet-Massicotte, Mariel Micael, Yunsik Kang, Marc Freeman

**Affiliations:** a Oregon Health and Science University, Portland, OR, USA

## Abstract

Glia are known to be critical regulators of synapse development, including both synapse formation and subsequent synapse elimination. However, how neurons and glia work together to determine when and specifically which synapses should be formed and eliminated remains poorly understood. To identify candidate molecules required for this process, we performed an in vivo genetic screen. We knocked down over 1,000 genes specifically in glia and then read out levels of synaptic proteins in Drosophila lysates by ELISA to identify candidate glial genes required for synapse development. Of the 91 hits from the screen, six homologs for immune-related scavenger receptors were identified, including Crq (homologous to CD36/SCARB2). Variants in these Crq homologs are associated with epilepsy and neurodegenerative diseases, suggesting that they can play an important role in regulating nervous system function, but what this role might be has not been defined. To define its role in nervous system development, we evaluated how synapses were changed when Crq was knocked down in glia. We found that Crq knockdown resulted in an increase in synapses, and this was driven by a reduction in synapse elimination. Interestingly, Crq seems to be specifically required for developmental synapse elimination, as it was not required for clearance of neuronal debris after injury or during metamorphosis. Ongoing work aims to determine whether its mammalian homologs play conserved roles in regulating synapse elimination by glia, and how the increase in synapses when Crq is knocked down affects circuit function and behavior. Future work will use proximity labeling to identify the neuronal ligands of Crq, which may serve as tags that direct specific synapses for elimination. We have also generated a tool to endogenously label synapses in an inducible manner, which will allow us to address which subtypes of neurons undergo synapse elimination in development, and determine which of those events are Crq-dependent. These tools will also be used to identify the cellular and molecular pathways by which other genes identified in the screen mediate glial regulation of synapses, in pursuit of understanding the rulebook that neurons and glia follow as they collaboratively shape the synaptic architecture of the nervous system.

# Abstract: P01.065 – Myelination and Lipid Metabolism in the Adolescent HIV-1 Transgenic Rat Brain

**Published:** 2025-09-05

**Authors:** Marisa Jeffries, Micah Romer, Melanie Cruz-Berríos, Kelly Jordan-Sciutto, Judith Grinspan

**Affiliations:** a Division of Neurology, Children’s Hospital of Philadelphia, Philadelphia, PA, USA; b Department of Oral Medicine, University of Pennsylvania, Philadelphia, PA, USA

## Abstract

The prevalence of human immunodeficiency virus (HIV)-associated neurocognitive disorders (HAND) remains at 30–50% of people with HIV (PWH) and is associated with persistent white matter pathologies. New HIV infection rates are climbing among adolescents, who may be particularly vulnerable to white matter disruption due to the critical window of adolescent myelination. The HIV-1 transgenic (Tg) rat model is an established noninfectious model of HIV neuropathology which may prove to be a valuable tool for investigating white matter abnormalities in neuro-HIV. However, despite published transcriptome data suggestive of altered myelination in this model, no studies to date have directly examined oligodendroglial myelination and potential mechanisms of white matter disruption in the HIV-1 Tg rat. Myelin is highly enriched in lipids, and the optimal ratio of protein and lipid is critical for oligodendrocytes to produce appropriate myelin structure and function. Interestingly, transcriptome analyses indicate reduced lipid metabolism and myelin proteins in PWH; furthermore disrupted brain lipid metabolism results in myelin abnormalities and is predictive of cognitive decline in HIV. Based on published and preliminary data, we hypothesized that glial lipid metabolism is disrupted by HIV-1 and impairs adolescent myelination. To address our hypothesis, we are analyzing oligodendroglial cell populations and myelination of the brain at 3 and 9 weeks of age. Western blot analyses of myelin protein expression in control and HIV-1 Tg rat microdissected caudate and cortex at 9 weeks of age indicate no significant changes in myelin basic protein (MBP), 2’,3’-cyclic-nucleotide 3’-phosphodiesterase (CNPase), myelin oligodendrocyte glycoprotein (MOG), or myelin associated glycoprotein (MAG), although there was a very strong trend (*p* = 0.054) toward increased MBP expression in the caudate. Similarly, no significant changes in MBP, CNPase, or MOG expression were observed in either prefrontal cortex or corpus callosa of HIV-1 Tg rats compared to controls, despite a trend toward decreased MBP in the callosum. Rather, we identified brain region-specific alterations in rate-limiting lipid biosynthesis enzymes. In the HIV-1 Tg rat caudate and cortex, expression of fatty acid synthase (FASN) was significantly decreased, and HMGCR was decreased in the caudate and similarly trending in the cortex. However, no changes in FASN were observed in the corpus callosa or hippocampi of HIV-1 Tg rats. To determine whether these changes in lipid biosynthesis enzymes are affecting myelin lipid composition, we are currently isolating myelin from control and HIV-1 Tg rat brains at 3 and 9 weeks of age to perform assays for total cholesterol and phospholipid content, as well as lipidomics. If changes in myelin lipid composition are observed, our next step will be to determine potential cellular mechanisms of altered lipid bioenergetics in oligodendroglia or other glial cells that support appropriate myelin formation through lipid synthesis and transport. If observed changes in lipid biosynthesis result in impaired adolescent myelination, lipid metabolism may be a promising therapeutic target to improve white matter integrity and ameliorate associated HAND pathology in PWH.

# Abstract: P01.066 – Circadian Alignment of Intermittent Fasting Is Crucial for Cerebral Ischemic Tolerance

**Published:** 2025-09-05

**Authors:** Soomin Jeong, Anil K. Chokkalla, Charles K. Davis, Hyunmook Jeong, Bharath Celluboina, Vijay Arruri, Bori Kim, V.Arumugam Thiruma, Barbara B. Bendlin, Raghu Vemuganti

**Affiliations:** a University of Wisconsin-Madison, Madison, WI, USA; b Seoul National University, South Korea; c La Trobe University, Melbourne, Australia; d William S. Middleton Memorial Veteran Administration Hospital, Madison, WI, USA

## Abstract

The beneficial effects of prophylactic intermittent fasting (IF) on brain health are extensively highlighted in neurological diseases, particularly after stroke. IF preconditions the brain, promote neurogenesis and plasticity, and improves cognitive and motor functions after a stroke. Although the duration of fasting was shown to elicit different levels of neuroprotection after ischemic stroke, the impact of fasting time with respect to the circadian cycles is not yet explored. We currently tested if circadian alignment plays a role in IF-induced differential ischemic tolerance and to elucidate the differentially altered mechanisms. Circadian cycle-dependent IF paradigms were established in adult male and female C57BL/6J mice by fasting for 16 h either during daytime (inactive phase IF) or nighttime (active phase IF). Following 6 weeks of active or inactive IF, mice were subjected to transient middle cerebral artery occlusion. Post-stroke tolerance was assessed by measuring brain damage (gray and white matter integrity) and neurobehavioral deficits (motor function, cognition, depression, and anxiety) from days 1 to 30 of reperfusion. Furthermore, transcriptomic profiling was performed to elucidate the putative mechanisms responsible for differential ischemic tolerance in the active versus inactive phase. Mice subjected to active phase IF showed better post-ischemic functional recovery compared to mice undergoing inactive phase IF, compared with ad libitum fed cohort. Moreover, circadian cycle-dependent IF is associated with broadly different transcriptomic alterations in the peri-infarct cerebral cortex. Together, our data indicate the regulation of brain preconditioning by the time of feeding and emphasize a crucial link between circadian cycle-dependent IF and ischemic tolerance.

# Abstract: P01.067 – Tweek-Dependent Formation of ER-PM Contact Sites Enables Astrocyte Phagocytic Function and Remodeling of Neurons

**Published:** 2025-09-05

**Authors:** Yunsik Kang

**Affiliations:** a Vollum Institution OHSU, Lake Oswego, OR, USA

## Abstract

Neuronal remodeling generates an enormous amount of neuronal debris, which is cleared from the nervous system by glia. At the larva-to-adult transition, Drosophila astrocytes transform into phagocytes and engulf degraded larval synapses and axonal and dendritic debris. To identify candidate molecules that regulate this process we did a screen and identified tweek. To determine how tweek regulated phagocytic function, we defined how astrocytes engage in phagocytosis of neuronal debris. Tweek is a member of the bridge-like lipid transfer protein family, is upregulated in astrocytes as they ramp up their phagocytic function, and is essential for continued internalization and degradation of neuronal debris. Tweek forms a bridge between the endoplasmic reticulum (ER) and plasma membrane (PM) and loss of Tweek disrupts ER-PM contact formation and lipid distribution. Patient-identified mutations in the human homolog associated with Alkuraya-Kucinskas syndrome resulted in similar phenotypes, indicating that these are loss of function. We propose Tweek helps establish and maintain ER-PM contacts during astrocyte phagocytic function and drives bulk lipid transfer to the plasma membrane for continued efficient internalization of neuronal debris.

# Abstract: P01.068 – Defining the Role of B-Raf and mTOR Signaling in Spinal Cord Oligodendroglia

**Published:** 2025-09-05

**Authors:** Ms. Divyangi Kantak, Teresa Wood, Angelina Evangelou

**Affiliations:** a Rutgers-New Jersey Medical School, Newark, NJ, USA

## Abstract

Multiple Sclerosis (MS), a demyelinating disorder affecting more than 2.5 million people worldwide, is characterized by the loss of myelin that results in attenuated saltatory conduction and axonal dystrophy. Myelin loss can result in deterioration of cognitive and motor function. In addition to demyelinating diseases, developmental disorders can disrupt myelin formation resulting in dysmyelination. Regeneration of myelin is contingent in large part on the differentiation of oligodendrocyte precursor cells (OPCs) into mature myelinating oligodendrocytes. Myelination of the central nervous system (CNS) is a complex multistep process that is governed by multiple extracellular signal transduction pathways. Disruption of the mitogen activated protein kinase pathway (MAPK) through deletion of ERK in mice results in CNS hypomyelination [4]. Interestingly, Raf kinases like B-Raf, direct upstream activators of ERKs are essential for neuronal generation and development of synaptic circuitry in various brain regions. Previous research revealed that neural precursor cell (NPC) specific deletion of the B-Raf kinase in mice results in a striking hypomyelinating and neurodegenerative phenotype [5–7]. These studies also demonstrated that absence of B-Raf in NPCs hinders OPC differentiation [5]. Additionally, phosphatidyl-inositol-3-phosphate kinase (PI3K/Akt/mTOR) pathway is a crucial regulator of oligodendrocyte differentiation and CNS myelination. Mice lacking mTOR in oligodendrocytes display significantly thinner myelin ensheathing spinal cord axons along with impaired oligodendrocyte maturation [1–3]. Previous research presents strong evidence pertaining to the autonomous role of both B-Raf and mTOR in regulating oligodendroglial development and myelination. However, how these two signaling cascades coordinately regulate myelination remains unexplored. To elucidate the function of B-Raf and/or mTOR in oligodendroglia, we developed a rodent model in which Braf Mtor and/or floxed alleles are conditionally deleted in OPCs by Cre recombinase expression under the control of the CNPase (2’,3’-Cyclic nucleotide 3’-phosphodiesterase) promoter. Gene expression analysis revealed significant downregulation in mRNA levels of several myelin proteins crucial for myelin sheath formation and axonal ensheathment in spinal cords of young adult mice lacking Braf or Braf;Mtor. Consistent with these observations we also observed reduction in the number of myelinated axons in the spinal cords of 1 year old mice. Additionally, motor function studies revealed motor deficits in mice lacking both Braf and Mtor in oligodendroglia. These analyses are ongoing to directly assess the impact of individual or combined deletion of Braf and Mtor on developmental myelination and adult myelin. Our future studies will help in determining the mechanism by which B-Raf and mTOR signaling interact to orchestrate CNS myelination.

# Abstract: P01.069 – High-Mobility Group Box-1 HMGB1-Mediates Isenecense And Contributes To Cognitive Dysfunctions In Tauopathies

**Published:** 2025-09-05

**Authors:** Rakez Kayed

**Affiliations:** a University of Texas Medical Branch, Galveston, TX, USA

## Abstract

High mobility group box protein 1 (HMGB1) is a ubiquitous nuclear protein, which is excessively released by glial cells during aging. The extracellular HMGB1 serves as a key initiator of neuroinflammation, which is linked to exacerbated cognitive impairments in tauopathy patients including Alzheimer’s disease (AD) and Frontotemporal dementia (FTD). Importantly, aging – the strongest risk factor for tauopathies is characterized by a chronic, low-grade inflammation known as inflammation. Evidence suggests that senescent cells (SC) accumulate with aging and are characterized by an apoptosis-resistant, arrested cell cycle with a distinct "inflammatory" phenotype known as senescence-associated secretory phenotype (SASP). However, the precise etiology of inflammation in tauopathies remain largely unknown. In this study, we demonstrate that HMGB1 release is linked to advanced age in hTau tauopathy mice and associated with astrocytes senescence-like phenotype. Pathological tau oligomers (TauOs) exposure triggers astrocyte senescence through HMGB1 release, which mediates paracrine senescence in adjacent cells and potentiates inflammatory SASP. Moreover, in primary culture Inhibition of HMGB1 release using ethyl pyruvate (EP) and glycyrrhizic acid (GA) results in decreased astrocyte senescence, in vivo, EP + GA treatment significantly decreased SCs loads in the brain, reduces inflammation, and thus ameliorated cognitive deficits in12-month-old hTau mice. Collectively, our data suggest that HMGB1 release promotes inflammation, and cellular senescence, which could represent an important pathomechanism in the progression of aging-related diseases, including AD and FTD. Future studies are warranted to develop safe and effective therapeutic strategies to control tau aggregation and senescence in tauopathy.

# Abstract: P01.070 – Investigation of Cyp46a1 and Cholesterol Homeostasis as a Novel Therapeutic Target for Rett Syndrome Phenotypes in Mecp2-Mutant Mice

**Published:** 2025-09-05

**Authors:** Miss. Nasim Khatibi, Ashis Sinha, Mayara C. Ribeiro, Jessica L. MacDonald

**Affiliations:** a Department of Biology, Syracuse University, Syracuse, NY, USA

## Abstract

Rett syndrome (RTT) is a severe progressive X-linked neurodevelopmental disorder caused by mutations in methyl-CpG-binding protein 2 (MECP2). RTT is the second leading cause of intellectual disability in girls, yet there is no cure and available treatment options are concentrated on relief of specific symptoms. Thus, our work is focused on finding novel therapeutic targets for RTT. Among the myriad cellular pathways disrupted in Mecp2-mutant mouse brain (null male and heterozygous female) is aberrant activation of the NF-κB pathway. Previous work demonstrated that genetically attenuating the NF-κB pathway rescues reduced neuronal size and complexity phenotypes and increases the reduced lifespan in these RTT model mice. Building on this, we demonstrated that vitamin D (VitD), a clinically applicable NF-κB inhibitor, rescues RTT phenotypes in Mecp2-mutant mice, including increased neuronal size and complexity as well as improved anxiety-like behavior and lifespan. We identified over 200 differentially expressed genes in the female Mecp2 heterozygous (Het) neocortex whose expression is normalized with dietary VitD supplementation, suggesting that they could underpin this rescue of phenotypes. Among these is Cyp46a1, a brain-specific cholesterol homeostasis enzyme that we found is downregulated in Mecp2 Het cortex at highly symptomatic stages but rescued to wildtype levels with VitD dietary supplementation. We have confirmed that the reduced expression of Cyp46a1 is a result of loss-of-function of Mecp2 using an in vitro CRISPR-mediated Mecp2 knock out in mouse cortical neurons, and we have demonstrated that expression of Cyp46a1 is rescued with VitD supplementation within the culture media. We are further investigating whether down-regulation of Cyp46a1 expression contributes to the reduced neuronal size and complexity phenotypes of Mecp2-null neurons. Cholesterol is synthesized in situ by astrocytes as it cannot cross the blood-brain-barrier. Cyp46a1 is expressed by neurons and catabolizes cholesterol to 24S-hydroxycholesterol, allowing it to diffuse across the blood-brain-barrier for turnover. Cholesterol homeostasis in the brain thus involves a complex interplay between neurons and astrocytes that must be very tightly regulated; either insufficient or excess neuronal cholesterol impairs synaptic plasticity and contributes to multiple neurological conditions. We are thus investigating whether dysregulation of Cyp46a1 expression in Mecp2-mutant brains is due solely to cell-autonomous loss of regulation by MeCP2, or whether it is caused by disruptions in cholesterol metabolism in astrocytes. These studies are essential to elucidate mechanisms of cholesterol homeostasis disruptions in RTT brain and to understand the therapeutic potential of Cyp46a1 modulation.

# Abstract: P01.071 – Choline Transporter-Like Protein 1 (CTL1) Regulates Intracellular Choline Metabolism and Phosphoinositide Signaling in Schwann Cells

**Published:** 2025-09-05

**Authors:** Nisha Gautam, Corey Heffernan, Patrice Maurel, Haesun Kim

**Affiliations:** a Rutgers University, Newark, NJ, USA

## Abstract

**Background:**

Choline is a precursor to phospholipids, which are major components of the myelin sheath. Choline also contributes to the biosynthetic pathway generating phosphoinositides, important signaling lipids that regulate myelination. Cells have a limited capacity to synthesize choline, necessitating its transport into the cells. Despite the well-described importance of choline-derived phospholipids and phosphoinositides in Schwann cells, the mechanism by which Schwann cells import choline remains unknown. We have previously shown that Schwann cells express solute carriers 44A2, also known as choline transporter-like protein 1 (CTL1), which is an intermediate-affinity choline transporter. Since Schwann cells do not express the high-affinity choline transporter (ChT), we tested the hypothesis that CTL1 functions as the primary choline transporter in Schwann cells.

**Methods:**

To test the hypothesis, we generated Schwann cell-specific CTL1 knock-out mice (Dhh-Cre:CTL1flox/flox; CTL1 sc-KO). MALDI-TOF mass spectrometry analysis was performed to determine whether CTL1 knockdown impairs choline import in Schwann cells. Lipidomic and bulk RNAseq analyses were performed on the sciatic nerves to compare the choline-derived lipid and Schwann cell gene expression profiles in control and mutant mice. Lastly, the impact of CTL1 loss on myelination was assessed by biochemical, immunohistochemical, and EM analysis.

**Results:**

CTL1 loss resulted in a significant reduction in intracellular choline levels in Schwann cells. We also observed dysregulation in choline-derived lipids, including phosphatidylcholine, phosphatidyl-ethanolamine, and sphingomyelin. RNAseq analysis revealed dysregulation of genes involved in phosphoinositide metabolism, including PI kinases and phosphatases. Consequently, we observed an aberrant increase in PI(3,4,5)P3-mediated Akt and mTORC1 signaling in CTL1 sc-KO sciatic nerves. The sciatic nerves of CTL1 sc-KO mice exhibited hypermyelination and an increased incidence of myelin outfolding and infolding, as well as an overall increase in myelin protein expression.

**Conclusion:**

These results show that CTL1 functions as a choline transporter in Schwann cells and regulates myelin homeostasis through the modulation of lipid metabolism and phosphoinositide signaling, crucial for Schwann cell myelination.

# Abstract: P01.072 – TFEB/3 Govern Repair Schwann Cell Generation and Function Following Peripheral Nerve Injury

**Published:** 2025-09-05

**Authors:** Akash Patel, Hyukmin Kim, Raghu Ramesh, John Svaren, Young-Jin Son, Radek Dobrowolski, Haesun Kim

**Affiliations:** a Rutgers University, Newark, NJ, USA; b Temple University, Philadelphia, PA, USA; c University of Wisconsin-Madison, Madison, WI, USA

## Abstract

TFEB and TFE3 (TFEB/3), key regulators of lysosomal biogenesis and autophagy, play diverse roles depending on cell type. This study highlights a hitherto unrecognized role of TFEB/3 that is crucial for peripheral nerve repair. Specifically, it promotes generation of progenitor-like repair Schwann cells following axonal injury. Deleting TFEB/3 in Schwann cells disrupts the transcriptomic reprogramming essential for the formation of repair Schwann cells. Consequently, mutant mice fail to populate the injured nerve with repair Schwann cells and exhibit defects in axon-regrowth, target reinnervation and functional recovery. TFEB/3 deficiency leads to reduced expression of repair Schwann cell genes including Sox2, Runx2, p75-Ngfr and Gdnf, without impacting the expression of c-Jun, a known regulator of repair Schwann cell function. TFEB/3 binding motifs are enriched in injury-induced enhancers, suggesting their role in repair gene activation. Autophagic activation in Schwann cells is unaffected despite TFEB/3 deficiency. These findings underscore a unique role of TFEB/3 in adult Schwann cells essential for peripheral nerve regeneration.

# Abstract: P01.073 – Neuroprotective Properties of the Methyl Donor Betaine in EAE

**Published:** 2025-09-05

**Authors:** Katherine Knies, Marwan Shalih, Morgan Psenicka, Robert Clements, Jessica Williams, Jennifer McDonough

**Affiliations:** a Biological Sciences, Kent State University, Kent, OH, USA; b Lerner Research Institute, Cleveland Clinic, Cleveland, OH, USA

## Abstract

Multiple Sclerosis (MS) is an inflammatory, demyelinating, and neurodegenerative disorder that affects two million people worldwide. Most of the therapies in MS only target the immune system and not the neurodegeneration that occurs during the course of the disease. Mitochondrial damage contributes to neurodegeneration in MS. It has been shown there is one carbon metabolism dysregulation in MS that is associated with mitochondrial dysfunction in neurons. This one carbon metabolism dysregulation results in a deficiency in the methyl donor betaine in the MS brain. This limits S-adenosyl methionine (SAM) synthesis and results in aberrant gene expression that impacts oligodendrocyte maturation and mitochondrial health. Betaine homocysteine methyltransferase (BHMT) requires betaine as a methyl donor for transmethylation reactions that generate SAM required for histone and DNA methylation. In the present study, we tested the effects of enhancing one carbon metabolism with the methyl donor betaine in a mouse model of MS. We injected betaine intraperitoneally to treat the motor symptoms in the experimental autoimmune encephalomyelitis (EAE) mouse model of MS. To induce EAE, mice were immunized with a myelin oligodendrocyte glycoprotein peptide and adjuvants. Betaine was supplemented intraperitoneally to animals at peak disease, which is around day 15 throughout the chronic stages of the disease (day 25–40 post-immunization). There was a significant improvement in motor ability in the betaine treated group compared to vehicle treated group. Using immunohistochemistry of the ventral horn of the spinal cord, BHMT was found to be located in the nucleus of the NeuN + neurons of wildtype and EAE mice. Cell counts of NeuN + neurons in the ventral horn of the spinal cord using immunohistochemistry showed that betaine supplementation protected neurons. Demyelination was also ameliorated with betaine supplementation. Statistical analyses were done with either a one-way ANOVA or student’s T-test with p ≤ 0.05 to be considered significant. Previous studies have shown that betaine increases H3K4me3 and expression of mitochondrial genes in neurons in cell culture. Effects of the BHMT-betaine pathway on mitochondrial function in EAE are being tested. In conclusion, betaine supplementation decreased disability in the EAE model of MS by supporting neuronal health and myelination.

# Abstract: P01.074 – Anti-PLP1 IgG1 Cloned From Patients With Multiple Sclerosis Impedes Oligodendrocyte Differentiation and Induces Myelin Pathology Independently of Demyelination

**Published:** 2025-09-05

**Authors:** Andrew Lapato, Nicholas Jahahn, Katherine Given, Greg Owens, Jeff Bennett, Wendy Macklin

**Affiliations:** a Department of Cell and Developmental Biology, University of Colorado School of Medicine, Denver, CO, USA; b Department of Neurology and Ophthalmology, University of Colorado School of Medicine, Denver, CO, USA

## Abstract

Patients with multiple sclerosis (MS) accumulate demyelinating lesions as the disease progresses that frequently do not repair. One under-examined mechanism of remyelination failure in MS may be persistence of immunoglobulins (Igs) within the lesion after initial injury. Using an ex vivo murine organotypic cerebellar demyelination/remyelination culture model, we show that recombinant IgGs (rAbs) that were cloned from MS patient intrathecal B cell repertoires and which bind a PLP1-dependent epitope hamper remyelination independently of complement activation. In this system, the myelin formed during remyelination in the presence of MS rAbs displays various abnormalities, including outfolding and lack of association with remaining axons. Glutathione-S-transferase π immunoreactivity was less frequent among CC-1+ post-mitotic oligodendroglia in MS rAb-treated cultures, suggesting that MS rAbs are sufficient to impede oligodendrocyte maturation (*n* = 25–40 folia). To understand how MS rAbs affect oligodendroglia specifically in this system, we investigated their effect on primary rat oligodendrocyte precursor cells (rOPCs). Similar to findings in cerebellar slices, when primary rOPCs were differentiated for five days in the presence of the cloned PLP1 MS rAb, fewer galactosylceramide (GalCer)+ mature oligodendrocytes were observed, suggesting MS rAbs elicited a deficit in differentiation directly, rather than through secondary signaling from other cells. Single-cell analysis of anti-PLP1-treated GalCer-expressing oligodendroglia revealed increased myelin protein immunoreactivity/cell, relative to control rAb or vehicle, suggesting dysregulated myelination in cells that reached maturity. Interestingly, these cells had more robust phosphorylation of the Src family kinase, Fyn. Intriguingly, when rOPCs were differentiated on inert 2 µm nanofibers, cells made fewer wraps and had shorter average wrap lengths in the presence of MS rAb relative to control (*n* = 15–40 cells). Together, these data suggest that anti-PLP1 IgG deposition alone may interfere with myelin repair, representing a heretofore under-appreciated mode of remyelination failure in MS.

# Abstract: P01.075 – Elucidating Interactions Between Triggering Receptor Expressed on Myeloid Cells 2 and Apolipoprotein E in Microglial Activation With Molecular Dynamics Simulations

**Published:** 2025-09-05

**Authors:** Emma Lietzke, Kimberley Bruce, Kayla Sprenger

**Affiliations:** a University of Colorado, Boulder, Boulder, CO, USA; b University of Colorado Anschutz Medical Campus, Aurora, CO, USA

## Abstract

Alzheimer’s disease (AD) is a progressive and ultimately fatal neurodegenerative condition affecting millions of Americans annually. While changes in brain morphology serve as key indicators of the disease, its root cause remains unidentified. A few genetic variants, such as specific isoforms of apolipoprotein E (ApoE) and mutations in the microglial surface protein Triggering receptor expressed on myeloid cells 2 (TREM2), have been associated with an increased risk of AD. Notably, ApoE4 and TREM2 R47H rank as the foremost and second-greatest known genetic risks for late-onset AD, respectively. Their direct interaction is believed to have synergistically detrimental effects on AD pathology. Despite the identification of these variants, the molecular mechanisms underlying the interaction between TREM2 and ApoE, as well as how these interactions are influenced by the presence of AD risk-associated mutations, remain elusive. Herein, we describe the use of molecular docking and molecular dynamics simulations to investigate direct binding interactions between TREM2 and monomeric ApoE. These simulations illustrate the impact that AD-associated mutations in TREM2 (R47H) and isoforms of ApoE (ApoE2/3/4) have on ligand binding. Our simulations recapitulate evidence of key interaction residues on both TREM2 and ApoE surfaces involved in binding. Moreover, our results suggest that structural differences in ApoE4 allow it to have higher levels of interactions with residues within TREM2’s complimentary-determining region 2 (CDR2), a key binding site. In contrast, simulations involving TREM2 R47H exhibited a significant lack of binding interactions at CDR2. Overall, this work provides novel insights into how potential antagonistic protein-protein interactions due to multiple mutations impact ligand binding events at the microglial cell surface. More broadly, the observed changes in binding suggest possible alterations in downstream microglial signaling/activation, which could have important consequences for the onset and/or progression of AD.

# Abstract: P01.076 – Characterization of Neurogenic Fate Decisions in TSC2-/- Induced Pluripotent Stem Cell-Derived Model

**Published:** 2025-09-05

**Authors:** Lisa Lin, Josh Smithman, Siddhi Patel, Shama Nazir, Lisa Julian

**Affiliations:** a Department of Biological Sciences, Simon Fraser University, Burnaby, Canada; b Centre for Cell Biology, Development, and Disease, Simon Fraser University, Burnaby, Canada; c Institute for Neuroscience and Neurotechnology, Simon Fraser University, Burnaby, Canada; d Faculty of Health Sciences, Simon Fraser University, Burnaby, Canada

## Abstract

**Introduction:**

Acquisition of specialized post-mitotic identity from proliferative stem cells is a tightly regulated process involving multiple cellular systems. Dysregulation of cell identity decisions in the brain during fetal growth is implicated in many neurodevelopmental disorders. One disorder, tuberous sclerosis (TS), is caused by inactivating germline mutation of the TSC1/2 complex which inhibits mTORC1, a major nutrient-sensing kinase and critical regulator of metabolism and cell growth. In TS, mTORC1 hyperactivation causes upregulation of anabolic processes, accumulation of lysosomes, and elevated ER stress in neural stem cells (NSCs). Additionally, development of tumor-like cortical malformations called subependymal giant cell astrocytomas and cortical tubers is observed. Composed of hyperactive neurons, hypertrophic glial cells, and stem-like giant cells, these cortical malformations are benign but cause severe functional consequences, including intellectual and behavioural disabilities and seizures. Although the role of mTORC1 in regulating cell survival processes through metabolism and autophagy is well-studied, the mechanism by which dysregulation results in the abnormal neural cell fate decisions that give rise to low-grade tumor development in the brain is unclear.

**Objectives:**

Using genetically engineered human pluripotent stem cells (hPSCs), we have created a model that parallels the morphological and functional characteristics of TS patient neural tumors and recapitulates the disease progression in vitro. Using this model, we aim to characterize the intracellular changes that occur and determine how these molecular alterations impact early neurogenic fate decisions that underly the aberrant tumor development.

**Methods:**

hPSC-derived NSCs that carry inactivating mutations in TSC2 (TSC2-/-) or their isogenic wild-type (WT) counterparts are analyzed using functional metabolic measurements and organelle fluorescent dyes. To further validate effects of metabolism on neurogenesis, metabolic substrate availability will be modified by 1) introducing a hypoxic environment to limit metabolism to glycolysis, 2) adding 2-deoxyglucose, a glucose competitor, to reduce glycolysis, and 3) adding oligomycin to inhibit mitochondrial activity. NSCs then undergo directed differentiation and are assessed for proficiency in producing functional neurons.

**Results:**

While WT NSCs produce neurons more readily, TSC2-/- NSCs show increased oxygen consumption and extracellular acidification rate, indicating an amplified metabolic shift in oxidative phosphorylation and glycolysis, show increased intracellular expression of ER, lysosome, and mitochondria, and produce a largely heterogenous population, comprised mostly of stem-like giant cells. We have also established quantitative parameters to implement a machine-learning guided image-based high-content compound screen to identify 1) potential pharmacological targets to rescue disease phenotypes, and 2) the molecular pathways that drive abnormal neuro- and astrogenesis.

**Conclusion:**

These data demonstrate the reliance of proper neurogenesis on normal metabolic and organelle function, and that dysregulation of these processes underlies altered cell identities that lead to tumor formation in TS. Current pharmaceutical interventions use mTORC1 inhibiting rapalogs to induce partial tumor regression, but continual mTORC1 inhibition has deleterious side effects. Elucidating the biological pathways involved in TS ultimately informs us how tumor development and migration occurs and how it can be effectively targeted therapeutically. This will allow us to identify new strategies for evaluating cancer risk, early detection screening, and avenues for precision medicine with improved prognosis.

# Abstract: P01.077 – Respiratory Infection With Influenza a Virus Alters Glial Metabolism

**Published:** 2025-09-05

**Authors:** Allison Louie, Rodney Johnson, Andrew Steelman

**Affiliations:** a Neuroscience Program, University of Illinois at Urbana-Champaign, Urbana, IL, USA; b Department of Animal Sciences, University of Illinois at Urbana-Champaign, Urbana, IL, USA; c Division of Nutritional Sciences, University of Illinois at Urbana-Champaign, Urbana, IL, USA; d Carl R. Woese Institute for Genomic Biology, University of Illinois at Urbana-Champaign, Urbana, IL, USA

## Abstract

Myelination has been increasingly recognized as a mediator of neural plasticity and myelin dysfunction has been linked to cognitive impairment. Recently, it was shown that oligodendrocytes (OLs) and myelin are vulnerable to environmental insults including stress, diet, and respiratory infections caused by influenza and SARS-CoV-2 viruses. We previously found that respiratory infection with a non-neurotropic strain of influenza A virus induced a pro-inflammatory glial response that coincided with the suppression of transcripts and proteins required for OL maturation and myelin generation. Here, we expand on these findings and demonstrate that both microglia and OL metabolism is altered by peripheral influenza A virus infection. Specifically, we utilized Single Cell ENergetIc metabolism by profilIng Translation inHibition, a recently described flow cytometry-based approach to assess cellular metabolism ex-vivo, and found that infection increased the glycolytic capacity of both microglia and OLs in a manner that was dependent on infectious dose. These changes to glial metabolism were recapitulated in vitro following LPS stimulation of primary mixed glia cultures containing astrocytes, microglia, and OLs. In contrast, we observed increased mitochondrial-dependent energy production in OLs of myelinating P14 mice compared to adult and aged mice. Based on these results, we hypothesize that shifting away from mitochondrial respiration towards glycolysis may be beneficial for OL survival during neuroinflammatory events, but limits their capacity for maturation. Collectively, our data indicate that respiratory infection with a non-neurotropic virus is capable of altering OL metabolism, which may have implications for modulating OL function.

# Abstract: P01.078 – Respiratory Infection With Influenza a Virus Delays Remyelination Following Cuprizone-Induced Demyelination

**Published:** 2025-09-05

**Authors:** Allison Louie, Jenny Drnevich, Andrew Steelman

**Affiliations:** a Neuroscience Program, University of Illinois at Urbana-Champaign, Urbana, IL, USA; b Roy J. Carver Biotechnology Center, University of Illinois at Urbana-Champaign, Urbana, IL, USA; c Department of Animal Sciences, University of Illinois at Urbana-Champaign, Urbana, IL, USA; d Division of Nutritional Sciences, University of Illinois at Urbana-Champaign, Urbana, IL, USA; e Carl R. Woese Institute for Genomic Biology, University of Illinois at Urbana-Champaign, Urbana, IL, USA

## Abstract

Environmental factors are known to influence the natural history of multiple sclerosis (MS). Among these, viral infection has been repeatedly associated with increased risk of relapse in persons with MS. Increased relapse frequency may also reduce remyelination capacity. Studies performed in animal models indicate that remyelination can restore proper signal conduction, prevent axonal injury, and promote recovery of function. However, whether respiratory viral infection can influence endogenous remyelination capacity is currently unknown. Given the ability for peripheral viral infections to disrupt oligodendrocyte (OL) homeostasis, we hypothesized that respiratory infection with influenza A virus could affect remyelination capacity following acute cuprizone (CPZ)-induced demyelination. By spatial transcriptomic analysis in conjunction with immunostaining and three-dimensional reconstruction of myelinated fibers, we demonstrate that infection perpetuated a demyelination- and disease-associated OL transcriptional signature that resulted in delayed OL maturation and remyelination in the prefrontal cortex following CPZ-induced demyelination. Utilizing a recently described flow cytometry-based approach of assessing cellular metabolism ex-vivo, we found that infection suppressed global translation activity of OLs in the brain during remyelination compared to non-CPZ controls. In addition, we found that infection altered OL metabolism in a manner that opposed the metabolic profile induced by remyelination. Specifically, infection increased OL glycolytic capacity while mitochondrial-dependent energy production was increased during remyelination. Collectively, our data indicate that respiratory viral infection antagonizes remyelination capacity, and support the notion that infection-induced changes to metabolic function of OLs may contribute to impaired remyelination.

# Abstract: P01.079 – Mice Carrying a Novel NAMPT Mutation Exhibit Metabolic Impairments

**Published:** 2025-09-05

**Authors:** Samuel Lundt, Nannan Zhang, Shinghua Ding

**Affiliations:** a University of Missouri, Columbia, MO, USA

## Abstract

Nicotinamide adenine dinucleotide (NAD+) is one of the most abundant metabolites in the body and is critical for many cellular processes including glycolysis, oxidative phosphorylation, and DNA repair. In mammals, the majority of NAD + is produced intracellularly via the NAD + salvage pathway, where nicotinamide phosphoribosyltransferase (NAMPT) is the rate limiting enzyme. In adipose tissue, the loss of NAMPT disrupts cold tolerance and systemic metabolic functions. Our previous studies demonstrated that NAMPT is an essential for survival gene and loss of NAMPT is lethal. The present study characterizes the metabolic changes of mice harboring a point mutation which results in a P158A mutation in the NAMPT protein. The P158A mice exhibit a chronic NAD + deficit, however these mice appear phenotypically similar to age-matched wild type (WT) mice. Behavioral tests suggest that P158A mice do not display any cognitive deficits though some reduced motor abilities were observed. Given NAD + is important to many different pathways, we investigated whether aged P158A mice may experience any metabolic dysfunctions due to the low NAD + levels. We found P158A mice at 19–20-months-old have impaired adaptive thermogenesis, manifesting by less capable to maintaining core body temperature both in response to diet restriction and acute cold exposure compared to age-matched WT mice. Moreover, P158A mice demonstrated increased glucose intolerance and insulin resistance. In summary, the current study suggests P158A mice exhibit global metabolic impairments and broaden our understanding of NAD + metabolism in health and diseases.

# Abstract: P01.080 – Analysis of Senescent Cell Development and Depletion in Mice After Experimental Autoimmune Encephalomyelitis (EAE) Induction

**Published:** 2025-09-05

**Authors:** Zeeba Manavi, Phillip Gross, George Melchor, Mia Cozart, Jingwen Hu, Maryna Baydyuk, Jeffrey K. Huang

**Affiliations:** a Department of Biology, Georgetown University, Washington, DC, USA; b Interdisciplinary Program in Neuroscience, Georgetown University, Washington, DC, USA

## Abstract

Multiple sclerosis (MS) is a chronic inflammatory disease of the central nervous system (CNS), affecting more than two million people worldwide. MS pathology is characterized by profound physical disability and cognitive decline with limited therapeutic interventions. Current treatments targeting autoreactive immune cells are highly effective in reducing disease severity in relapse-remitting MS (RRMS). However, their efficacy becomes limited in progressive MS (PMS), indicating that a better understanding of the progressive stage of the disease is required. Cellular senescence is a feature of aging and chronic inflammation and has been observed in chronic MS lesions. Senescent cells secrete proinflammatory factors known as senescence-associated secretory phenotype (SASP) that can further prolong inflammation. However, whether cellular senescence plays a role in MS pathogenesis remains poorly understood. To examine the potential impact of cellular senescence on MS pathology, we examined markers of senescence in CNS inflammatory lesions of mice with experimental autoimmune encephalomyelitis (EAE), a preclinical model of MS, and determined if the pharmacological depletion of senescent cells in mice with EAE affects clinical disability. We observed canonical senescence markers such as senescence-associated β-galactosidase, p16, and p21 in the spinal cord of mice with EAE compared to naïve control. Furthermore, an increase in p16 and p21 was observed in the meninges adjacent to the ventral demyelinating lesions. This increase was quantified by flow cytometry analysis of EAE spinal cord and leptomeninges compared to naïve, and p21 expression was observed in 66.3 ± 6.4% of macrophages (MΦs). To determine the effect of senescent cell depletion on EAE pathology, we tested the therapeutic effect of Dasatinib and Quercetin (DQ), a known senolytic, on EAE clinical outcome. We found that prophylactic treatment of DQ resulted in a decrease in the percentage of p21 + MΦs, CD4+, and CD8+ T cells. However, no obvious changes to the clinical score were observed despite senescent cell depletion. These results suggest that although senescent MΦ (SnMΦs) may contribute to the inflammatory process in EAE, they do not appear to play a role in disease progression.

# Abstract: P01.081 – Genetic Pathways That Drive Axon Loss

**Published:** 2025-09-05

**Authors:** Ernesto Manzo, Alexandra Larson, Marc Freeman

**Affiliations:** a OHSU, Portland, OR, USA

## Abstract

Axon degeneration is a prominent feature of neurodegenerative diseases. The Loss of axons results in the irreversible destruction of neuronal and synaptic connections leading to compromised neuronal circuit function. Understanding the cellular biology of how neuronal fragmentation occurs in molecular terms, and finding ways to block it, will be an essential step if we wish to maintain neuronal health in disease or after neuronal damage. We have developed a novel screening method to precisely target the axon death pathway by depleting Nmnat (nicotinamide mononucleotide adenylyltransferase), the rate-limiting step of NAD + synthesis. Neuronal loss of Nmnat activity in mice and flies initiates spontaneous dSarm/Sarm1-mediated axon degeneration. We are using the adult Drosophila wing in combination with MARCM (mosaic analysis with a repressible cell marker) clonal technology and a genetically encoded neuronal ase-FLP recombinases, we seek to identify and characterize genes required to drive the degeneration of axons after dNmnat depletion in neurons. The MARCM approach allows us to generate homozygous mutant GFP + neuronal clones in the F1 generation that are depleted of dNmnat, and assess axon degeneration by visualizing axons directly through the wing cuticle. We performed an F1 forward genetic screen of 3311 mutant chromosomes, of which 12 lines showed axonal protection even after dNmnat depletion. Interestingly, this screen identified two members of the Roundabout (Robo) family of axon guidance receptors, which are best known for their roles in axonal outgrowth. This exciting observation identifies Robos as new molecules required for axon degeneration, and support the emerging model that many molecules that are initially used to build the nervous system, are also used to drive its destruction during neurodegeneration.

# Abstract: P01.082 – The Role of Aurora B Kinase in the Development of Neuron Dysfunction in Amyotrophic Lateral Sclerosis

**Published:** 2025-09-05

**Authors:** Sarah Martin, Mitch Eisenmenger, Leslei Lopez, Nicholas Romero, Ming Ying Tsai, Scott Brady

**Affiliations:** a University of Illinois at Chicago, Chicago, IL, USA

## Abstract

Amyotrophic lateral sclerosis (ALS) is a fatal neurodegenerative disease that affects the upper and lower motor neurons. While the cause of ALS is unknown, the most common genetic risk factor for ALS is an expansion of a hexanucleotide repeat in the chromosome 9 open reading frame 72 (C9orf72) gene. This repeat expansion leads to the expression and accumulation of 5 different dipeptide repeat proteins; however, it is unknown how these dipeptide repeats contribute to disease pathogenesis. There is evidence that nuclear dysfunction in neurons, such as abnormal size and shape, occurs in ALS. Our data shows that two of these C9orf72 dipeptide repeats, poly-RP and poly-RG, are cytotoxic when expressed in neuronal cells, producing nuclear and cellular pathological phenotypes. To explore a potential mechanism underlying the development of these pathologies, we focused on a signaling pathway that is critical for regulating nuclear processes. Aurora B kinase is a serine/threonine kinase that localizes to chromosomes and has been reported to play an important role in both mitosis and cytokinesis in differentiating cells, as well as neurite outgrowth in neurons. Based on its predominantly nuclear localization and functions, our hypothesis is that an impairment in Aurora B kinase activation contributes to the development of both nuclear pathologies associated with the poly-RP and poly-RG dipeptides. To address this hypothesis, we transfected rat primary cortical neurons with expression plasmids containing the C9orf72 dipeptides. We found that there are alterations in Aurora B kinase levels in cells expressing the poly-RP and poly-RG dipeptides, specifically a decrease in cells expressing the poly-RP dipeptide and an increase in cells expressing the poly-RG dipeptide. Treating primary cortical neurons that were transfected with the dipeptides with an Aurora B kinase inhibitor, Barasertib, resulted in a decrease in neurite length in cells expressing the poly-RP dipeptide and an increase in neurite length in cells expressing the poly-RG dipeptide, suggesting that Aurora B kinase may play a role in axonal processes. Our findings indicate that alterations in Aurora B kinase may be involved in the development of pathological phenotypes in C9orf72-associated ALS, perhaps establishing an overall mechanism of neurodegeneration.

# Abstract: P01.083 – Regional Differences in Oligodendroglial Cholesterol Acquisition and Myelin Lipid Composition

**Published:** 2025-09-05

**Authors:** Marie Mather, Luipa Khandker, Angelina Evangelou, Teresa Wood

**Affiliations:** a Rutgers University – New Jersey Medical School, Newark, NJ, USA

## Abstract

Cholesterol comprises over 40% of myelin lipids and is often dysregulated in neurodegenerative diseases affecting myelin integrity. Despite the prominence of promyelinating drugs targeting sterol synthesis and our increasing knowledge of oligodendrocyte heterogeneity, few studies have explored lipid metabolism in both the brain and spinal cord. Therefore, understanding how cholesterol metabolism is regulated in different oligodendrocyte populations is essential to developing effective promyelinating therapies. Our previous study revealed that spinal cord oligodendrocyte precursor cells (OPCs) have higher rates of cholesterol synthesis compared to brain OPCs. Further analyses show higher expression of lipoprotein receptors in brain oligodendroglia compared to spinal cord oligodendroglia throughout rapid myelin development (P10-P18). Subsequently, treatment of primary OPCs with lipoproteins resulted in increased myelin gene expression in brain OPCs while spinal cord OPCs showed no response. These data suggest that brain OPCs have a greater capacity cholesterol uptake rather than cholesterol synthesis. We also explored whether lower rates of cholesterol synthesis in brain oligodendrocytes could be due to lower lipid requirements to produce myelin. Analysis of myelin composition from spinal cord and several regions of the CNS revealed that brain myelin has a lower lipid concentration compared to spinal cord myelin. Subsequent regional comparisons suggest that myelin lipid content is correlated to average axon diameter within each region. The results of this study further highlight the regional specificity of both myelin and oligodendroglial populations, providing significant functional differences that should be considered when targeting components of lipid metabolic pathways.

# Abstract: P01.084 – A Novel CD27 + CD138+ B Cell Subset Localizes to the Brain in Aged Mice

**Published:** 2025-09-05

**Authors:** Annabel McAtee, Mary Colson, Jadwiga Turchan-Cholewo, Thomas Ujas, Katherine Cotter, Daimen Britsch, Edric Winford, Ann Stowe

**Affiliations:** a Department of Neuroscience, College of Medicine, University of Kentucky, Lexington, KY, USA; b Center for Advanced Translational Stroke Science (CATSS), University of Kentucky, Lexington, KY, USA; c Department of Neurology, College of Medicine, University of Kentucky, Lexington, KY, USA

## Abstract

**Objectives:**

B lymphocytes play a variety of roles in the post-stroke brain, including neurotrophic support of cognitive recovery, as our lab previously showed. Whether a B cell is detrimental or supportive after stroke likely depends on the location and timing of the responding B cell subset. Recent evidence also demonstrates that a B cell niche exists in the skull bone marrow and dura that leads to the maturation of antibody-producing B cells distinct from peripheral populations. However, it is unknown if cells from these regions contribute to post-stroke inflammation in the brain. Therefore, the objective of this study is to identify B cell populations in the brain that are distinct from peripheral populations and that may derive from the skull bone marrow.

**Methods:**

Aged (18–24 months) C57BL/6 female mice (*n* = 7–12 per group) underwent 30-min transient middle cerebral artery occlusion (tMCAo) on the left side and were sacrificed 3 weeks after injury, with uninjured mice serving as controls. Brains were divided into ipsilateral (left) and contralateral (right) cortex and cerebellum. Brains and spleens were processed into single cell suspensions and stained with a 19-antibody panel. Flow cytometry data were analyzed with uniform manifold approximation and projection (UMAP) with X-shift clustering to identify B cell populations. Live cells were gated on CD19 followed by CD27, CD23, CXCR5, CD138, and IgM. Initial population numbers were analyzed in GraphPad Prism with 2-way ANOVA with multiple comparisons (Benjamini; a = 0.05).

**Results:**

CD27 and CD23 are cell markers that are present on mature B cells and CD138 is a marker present on plasma cells. UMAP clustering showed a CD19 + CD27 + CD23 + CXCR5 + CD138 + IgM + cell population that was significantly higher in the left cortex versus the spleen and other brain regions in uninjured animals (all *p* < 0.01). Interestingly, this difference disappeared in injured animals with no significant differences between regions. Post-injury, there was a decline in this population in the left hemisphere only (*p* < 0.01). Confirmation of cells in brain regions is currently being confirmed by histology.

**Conclusions:**

We characterize a distinct population of CD27 + CD138+ activated memory B cells elevated in the left cerebral cortex at baseline that decreases after ipsilateral tMCAo. It is possible that, at baseline, these cells release antibodies and contribute to neuroinflammation known to develop with aging. It is unknown what factors are driving the laterality of this cell type in healthy aged mice. Further, it is unknown if the post-stroke decrease is due to phenotype change or migration out of the brain. Ongoing studies aim to characterize the origins and roles of this unique B cell population.

# Abstract: P01.085 – Dissection of Inter-Disorder Astrocyte Reactivity Reveals a Novel Astrocyte Subtype That Regulates White Matter Repair

**Published:** 2025-09-05

**Authors:** Sarah McCallum, Keshav Suresh, Timothy Islam, Ann Saustad, Oksana Shelest, Aditya Patil, David Lee, Connor Beveridge, Palak Manchandra, Riki Kawaguchi, Gaurav Choppra, Ranjan Dutta, Gordon Meares, Simon Knott, Joshua Burda

**Affiliations:** a Cedars Sinai Medical Center, Los Angeles, CA, USA; b Purdue University, West Lafayette, IN, USA; c University of California Los Angeles, Los Angeles, CA, USA; d Cleveland Clinic, Cleveland, OH, USA; e The Ohio State Wexner Medical Center, Columbus, OH, USA

## Abstract

Chronic white matter degeneration results in protracted innate immune inflammation and is a hallmark of diverse disorders, including traumatic and ischemic central nervous system (CNS) injury, and multiple sclerosis (MS). Debris accumulation within the CNS precipitates maladaptive, microglia-mediated neuroinflammation, which furthers disease progression and prevents repair. Moreover, it is unclear why the clearance of cellular debris from degenerating white matter within the CNS is inefficient compared to that in the peripheral nervous system (PNS). Therefore, it is necessary to uncover central regulatory mechanisms underlying the cellular responses that facilitate CNS debris clearance after white matter insult. We set out to delineate changes in molecular pathways of glial-glial communication that are required for cellular debris clearance from the damaged white matter. To do so, we performed single nuclei RNA-sequencing and spatial sequencing to evaluate gene expression of cells from the mouse spinal cord following an incomplete spinal cord injury (iSCI), which creates neuroanatomically predictable patterns of Wallerian Degeneration (WD) throughout spared neural tissue regions above and below the lesion. This approach enabled the assessment of the transcriptomic cellular landscape through acute, sub-acute, and chronic stages of WD and functional behavioral recovery. Using multiplexed RNA in-situ hybridization, and immunohistochemistry, we determined microglial profiles that correspond to the microglial populations that dominate white matter regions undergoing WD and phagocytose myelin debris. We postulated that astrocytes are well positioned to serve as regulators of myelin phagocytosing microglia since they i) rapidly sense and respond to damage and inflammation; and ii) regulate the immune response in diverse CNS disorders. Using our snRNA-Seq data, we identified a unique reactive astrocyte population, which was located exclusively within white matter tracts undergoing active WD. Our knock-out in-vivo studies suggest that WD-associated reactive astrocytes regulate the lipid metabolism of myelin phagocytosing microglia, disruption in this glia-glia communication pathway, enhance debris clearance and promote regeneration. Finally, investigation of alternative models that exhibit demyelination in both mice and humans suggests this astrocyte-microglial interaction is an evolutionary conserved response to demyelination. This research will enhance the understanding of how astrocyte-microglia interactions shape the CNS innate immune responses that enable white matter repair. This research may also aid the development of therapies aimed at enhancing debris clearance to promote repair and is thereby of broad translational relevance. Future work examining this unique WD-associated astrocyte population may identify key pathways that can be exploited to develop new therapeutics aimed at enhancing debris clearance to encourage neural repair.

# Abstract: P01.086 – Identifying Lanthionine Ketenamine (Ethyl Ester) (Phosphonate) Derivatives for Relative Maturation and Proliferation Effects in OPCs

**Published:** 2025-09-05

**Authors:** Zachary McDonald, Ankit Tandon, Jackie Rocha, Pablo Paez, Douglas Feinstein

**Affiliations:** a University of Illinois at Chicago, Chicago, IL, USA; b Department of Veterans Affairs, Chicago, IL, USA; c University of Buffalo, Buffalo, NY, USA

## Abstract

Identifying drug therapies that successfully induce remyelination is an important step in treating multiple sclerosis – a disease characterized by myelin loss. Stimulating oligodendrocyte (OLG) progenitor cell (OPC) proliferation and maturation are critical components to successful remyelination. Lanthionine ketenamine-5-ethyl ester (LKE), a synthetic derivative of the amino acid metabolite lanthionine ketenamine (LK), has previously been shown to increase markers of OPC maturation. In the present study, we characterize LKE and 7 derivatives (LK(E)-Ps) for their maturation, proliferation, and cytoprotective effects on primary mouse OPCs and the OPC-like Oli-neu mouse cell line. The derivatives in the study are modified from LK containing one or all the following: a 2’ position acyl chain (hexyl, butyl, octyl), 3’ position phosphonate, and/or 5’ ethyl ester. Employing immunocytochemistry (ICC), lactate dehydrogenase (LDH) viability assays, and BrdU proliferation assays, LK(E)-Ps were screened for their relative effects. LDH viability assays show LKE, butyl-LKE-P, octyl-LKE-P, and hexyl-LKE-P have cytoprotective effects in-vitro at 10 uM on primary mouse OPCs and oli-neu cells; no LK(E)-Ps were observed to have cytotoxic effects. OPCs stained for Olig2 (total OLGs), CC1 (mature OLGs), and PLP (mature OLGs) were increased after 48h incubation of LKE and hexyl-LKE-P at concentrations less than 50 uM; while butyl-LKE-P increased PLP expression. OPCs stained for Ki67 (proliferation) showed an increase when treated with LKE, hexyl-LKE-P, or butyl-LKE-P at 25 and 50 uM. Similarly, Oli-neu cells treated with 10 uM butyl-LKE-P showed a significant increase in incorporation of BrdU. These data suggest that over short time courses (24–48 h), LK(E)-Ps effect both the maturation and proliferation of OPCs. Moreover, LK(E)-Ps containing 5’-ethyl ester show greater cytoprotection than those without. While many of the LK(E)-Ps show significant effects on OPCs, the effects were not significantly different than those observed with LKE. Along with further characterization of existing LK(E)-Ps, additional LK derivatives are being tested to identify ones that show significant improvements compared to the maturation and proliferation effects of LKE on OPCs. This work was supported in part by grants from the National MS Society and the Department of Veterans Affairs.

# Abstract: P01.087 – RNA-Seq of Extracellular Vesicle (EV) RNA Separated From a Small Volume of Human Cerebral Spinal Fluid (CSF)

**Published:** 2025-09-05

**Authors:** Trevor McFarland, Ursula Sandau, Tina Chen, Taliah Soleymani, Edgar Gonzalez-Kozlova, Navneet Dogra, Julie Saugstad

**Affiliations:** a Icahn School of Medicine, OHSU, Portland, OR, USA; b New York, NY, USA

## Abstract

**Objective:**

EVs are membranous particles containing bio-reactive cargo which are secreted from cells and act as part of a complex cellular communication system. RNA sequencing of EV RNA isolated from small volumes of CSF is challenging and the number of protocols on this subject are limited. Typically, RNA-seq of CSF is performed using total CSF or from EVs separated from milliliter volumes. Given that human biorepositories typically bank volumes of CSF less than 1 mL, we aim to develop a pipeline for transcriptomic analysis of CSF EV RNA from 0.5 mL of CSF biofluid.

**Methods:**

The Institutional Review Board of the Oregon Health and Science University approved all human participant procedures. CSF from six healthy participants was collected via lumbar puncture under fasting conditions. 300 μL from each donor was pooled, mixed and aliquoted into three 0.5 mL samples. Each replicate sample was concentrated using a 30 kD cut-off ultra-filtration column to a final volume of 150 μL. Each concentrated sample underwent size exclusion chromatography (SEC) using 35 nm resin. A representative subset of the EV separated fractions were characterized by immunoblot, TEM and resistive pulse sensing (RPS). RNA was isolated from each replicate pool using the Urine miRNA purification kit (Norgen) and eluted with 30 μL of elution solution. RNA QC, library prep, RNA-seq and data analysis were performed at the Icahn School of Medicine using their liquid biopsy analysis pipeline.

**Results:**

Immunoblot, TEM and RPS verified the presence of EVs in the fractionated pool. RNA isolation yielded 2–7 ng of RNA and passed sequencing input RNA QC metrics. Following successful cDNA library construction and sequencing, the data analysis revealed a range of coding and non-coding RNAs. The majority of sequences were mapped to known genes (∼75%), while the remaining RNA species consisted of rRNA, lncRNA, and pseudogenes. MiRNA represented a very small percentage of the mapped sequences. Pathway analysis of the top 10% most expressed genes revealed that these EV RNAs are associated with regulation of neuron differentiation, projection development, transmembrane transport and glutamate signaling.

**Conclusion:**

EVs separated from 0.5 mL of CSF is suitable for RNA isolation and downstream RNA-seq. Future work includes repeating the procedure from a larger cohort of Alzheimer’s disease and control donors.

# Abstract: P01.088 – Development and Characterization of a Severe, Dietary Fat-Free Cuprizone Model of Remyelination

**Published:** 2025-09-05

**Authors:** Anna Mcgrath, Sonia Manca, Alexander Verbitsky, Vasily Belov, Yinyin Huang, Jeremy Huang, Giorgio Gaglia, Dinesh Bangari, Caroline Morel, Katie Malley, James Dodge, Stefanie Giera

**Affiliations:** a Rare and Neurological Diseases Therapeutic Area, Sanofi, Cambridge, MA, USA; b Global Bioimaging Group, Sanofi, Cambridge, MA, USA; c Single Cell Biology Cluster – PMCB, Sanofi, Cambridge, MA, USA; d Global Discovery Pathology Group, Sanofi, Cambridge, MA, USA

## Abstract

Demyelination is a hallmark of multiple sclerosis. As the myelin sheath is damaged, neuronal signaling is disrupted, eventually leading to neuronal death and permanent disability for patients. Oligodendrocytes produce myelin and can repair damage to the myelin sheath. However, the naturally occurring remyelination done by oligodendrocytes only partially reverses damage, and the effectiveness wanes as the disease progresses. As a result, there is much interest in developing treatments for patients with multiple sclerosis that would enhance remyelination and reverse symptoms. One popular method for studying remyelination is the cuprizone model. Cuprizone is a copper chelator that induces demyelination. Typically, the toxin is administered daily over 5–6 weeks, causing the death of oligodendrocytes and myelin loss. When treatment with cuprizone is terminated, remyelination occurs. For the current study, we sought to develop and characterize a cuprizone model of more severe demyelination than is observed in this widely used paradigm. Myelin consists mainly of lipids, particularly cholesterol. Dietary fat levels can contribute to myelination, with high fat diets increasing myelin markers in the central nervous system. Therefore, we hypothesized that removing dietary fat during cuprizone treatment would enhance demyelination. Using MRI, IHC and visually evoked potentials (VEP), we tracked demyelination and subsequent remyelination in this fat-free cuprizone model. We found that demyelination of the corpus callosum continues after the cuprizone treatment is terminated, with the most severe myelin loss occurring one week post treatment. VEP data suggests that remyelination of the optic nerve may occur more quickly, as signal transduction starts to improve one week post-treatment. Oligodendrocyte lineage cells were also characterized at various timepoints using snRNAseq. Our severe cuprizone model depletes oligodendrocytes after four weeks. By one week post treatment, both oligodendrocytes and oligo precursor cells have repopulated. Taken together, these data suggest that our dietary fat free cuprizone model effectively depletes oligodendrocytes and causes demyelination, even after the cuprizone treatment is stopped. In the weeks post treatment, oligodendrocytes repopulate and remyelination occurs. Differences in the optic nerve and corpus callosum data suggest possible regional differences in the remyelination timeline.

# Abstract: P01.089 – Th17 Cells Reprogram Astrocytes Through a JAK1 Dependent Process That Contributes to Autoimmune Neuroinflammation

**Published:** 2025-09-05

**Authors:** Gordon Meares, Jacob Feldmann, Sarah Milne

**Affiliations:** a Ohio State University, Columbus, OH, USA; b West Virginia University, Morgantown, VA, USA

## Abstract

Multiple sclerosis (MS) is an autoimmune demyelinating disease affecting the central nervous system (CNS). MS and the widely used animal model of experimental autoimmune encephalomyelitis (EAE) involve CNS infiltration of immune cells, including Th17 cells. These infiltrating lymphocytes produce effector molecules that drive neuroinflammation in part through engagement of resident glial cells. Among these glial cells, astrocytes have a significant role in coordinating inflammatory processes by responding to cytokines and chemokines released by Th17 cells. In this study, we sought to examine the impact and mechanism by which pathogenic myelin-reactive Th17 cells modulate astrocytes. Based on the cytokines that these T cells produce; we hypothesized that the receptor-linked Janus Kinase 1 (JAK1) mediates the astrocytic response to Th17 cells. To evaluate this, we used myelin oligodendrocyte glycoprotein (MOG35-55) primed Th17 cells in co-culture with primary astrocytes and adoptive transfer EAE. We identified that Th17 cells reprogram astrocytes by driving transcriptomic changes partly through a JAK1-dependent mechanism, which included increased cytokines, chemokines, and related receptors. Additionally, JAK1 was responsible for imposing an interferon responsive gene expression signature in the astrocytes following exposure to Th17 cells. Astrocyte selective deletion of JAK1 significantly attenuated Th17 induced EAE and diminished inflammatory gene expression. These data provide further evidence that astrocyte/T cell interactions are vital to driving autoimmune neuroinflammation, and that this process involves astrocytic JAK1 signaling.

# Abstract: P01.090 – Müller Glia Glutamate Metabotropic Receptors Regulation Upon Excitotoxic Conditions: Correlation to the Dark Cycle

**Published:** 2025-09-05

**Authors:** Yurixy Merari Mendoza Silva, Luisa Hernandez-Kelly, Arturo Ortega

**Affiliations:** a CINVESTAV, CDMX, Mexico

## Abstract

Glutamate (Glu) is the main neurotransmitter of the nervous central system (SNC), taking part in regulating a wide type of functions like cognitive, motor, sensitive, and visual to name a few. Glu synthesis mainly depends on the Glutamate/Glutamine cycle between neurons and astrocytes surrounding glutamatergic synapsis. In the retina, Müller glia cells surround the glutamatergic synapses of the three retinal layers of the visual pathways. Taking into consideration, that retinal glutamatergic synapses are active during the whole light/dark cycle Müller cells’ role is fundamental for glutamate turnover. In fact, a tight regulation in glial glutamate transporters EAAT1 and EAAT2 is expected to avoid an excitotoxic insult (dark phase) or a reduced glutamate stimulation (light phase). The role of glial glutamate receptors in this process is not known. Müller glia express ionotropic glutamate receptors of the AMPA, KA, and NMDA subtypes and the group I metabotropic receptors. With this in mind, in the present contribution we evaluated the involvement of group I metabotropic glutamate receptors in Glutamine uptake and release in purified chicken Müller glia cultures and in the human Müller glia cell line MIO-M1 cells. A time and dose-dependent decrease in Glutamine uptake is present after the exposure of the cultured cells to the broad-spectrum metabotropic agonist trans-ACPD agonist is added, suggesting that the involvement of mGluRs in Müller cells’ function. A detailed characterization of this response is underway and will be discussed.

# Abstract: P01.091 – Trem2 Mediates Sulfatide Deficiency-Induced Microglia-, but Not Astrocytes-Mediated Neuroinflammation or Lipid Homeostasis Disruption

**Published:** 2025-09-05

**Authors:** Namrata Mittra, Sijia He, Hanmei Bao, Anindita Bhattacharya, Jeffrey L. Dupree, Xianlin Han

**Affiliations:** a Barshop Institute for Longevity and Aging Studies, University of Texas Health San Antonio, San Antonio, TX, USA; b Division of Diabetes, Department of Medicine, University of Texas Health San Antonio, San Antonio, TX, USA; c Department of Anatomy and Neurobiology, Virginia Common Wealth University, Richmond, VA, USA; d Research Division, McGuire Veterans Affairs Medical Center, Richmond, VA, USA

**Keywords:** Trem2, sulfatide, astrocytes, microglia, inflammation, Alzheimer’s disease

## Abstract

Disrupted lipid homeostasis and neuroinflammation often co-exist in neurodegenerative disorders, including Alzheimer’s disease (AD). However, the intrinsic connection and causal relationship between these deficits remain elusive. Our previous studies show that loss of sulfatides (ST), a class of myelin-enriched lipids, causes AD-like neuroinflammatory responses, cognitive impairments, bladder enlargement, as well as lipid dyshomeostasis. To better understand the relationship between neuroinflammation and lipid disruption induced with ST deficiency, we established a ST-deficient mouse model with constitutional Trem2 knockout and investigated the impact of Trem2 in regulating ST deficiency-induced microglia and astrocyte-mediated neuroinflammation and lipid disruption. Our study demonstrated that Trem2 regulates ST deficiency-induced microglia and astrocyte-mediated neuroinflammatory pathways at the transcriptomic level, but not astrocyte-mediated neuroinflammation at the protein level, suggesting that Trem2 is indispensable for ST deficiency-induced microglia, but not astrocyte -mediated neuroinflammation. On the contrary, ST loss-induced lipidome disruption and bladder enlargement was consistently observed in the absence of Trem2. Collectively, these results emphasized the essential role of Trem2 in mediating lipid loss-associated microglia-, but not astrocyte-mediated neuroinflammation. Moreover, we demonstrated that attenuating neuroinflammation has limited impact on brain ST loss-induced lipidome alteration or AD-like peripheral disorders. Our findings suggest that maintaining lipid homeostasis may be a novel therapeutic approach against AD.

# Abstract: P01.092 – Examining TBI-Induced Astrocyte Heterogeneity Across Spatial and Temporal Boundaries

**Published:** 2025-09-05

**Authors:** Benjamin Wendell, Danielle Goulding, Amy Gorman, Josh Morganti

**Affiliations:** a University of Kentucky, Lexington, KY, USA

## Abstract

Understanding of the molecular linkages of astrocytes’ response to traumatic brain injury (TBI) are continuing to be unraveled. Our group has recently demonstrated that there are distinct aging responses to trauma transcriptionally, compared to young. However, we still lack an understanding of how different anatomical regions (e.g., hippocampus and neocortex) are linked with TBI, especially at the chronic period. In this study we utilized several contemporary techniques to elucidate the spatio-temporal heterogeneity in transcriptional repertoire of astrocytes. Two tamoxifen-inducible mouse models were utilized: Aldh1l1CreERT2 x RiboTag (‘AstroTag’), along with Aldh1l1CreERT2 x Ai9 (‘AstroReporter’), as well as standard C57BL6/J mice, only males were utilized for this study. Mice were subjected to our standard mild-moderate severity controlled cortical impact (CCI) model of TBI or sham surgeries. Animals were euthanized for tissue collection at either 3 or 28d post surgery. AstroTag mice tissue were snap frozen in LN2, while AstroReporter mice were processed for FACS enrichment and live astrocytes were collected based on their TdTomato + expression. Tissues from AstroTag mice were processed via standardized methods for RiboTag mRNA enrichment and utilized for bulk sequencing, while TdTomato + astrocytes were processed for single cell RNA sequencing. Overall, our results give an in depth examination of the susceptibility of astrocyte’s to TBI, highlighting distinct phenotypes conserved between cortical and hippocampus regions, while also demonstrating significant shifts associated temporal dynamics.

# Abstract: P01.093 – Mild Traumatic Brain Injury Induces an Astrocyte Atypical Response Astrocytes Mediated by Protein Degradation

**Published:** 2025-09-05

**Authors:** Carmen Munoz-Ballester, Kijana George, Benjamin Heithoff, Dzenis Mahmutovic, Samantha Golf, Alex Shandra, Stefanie Robel

**Affiliations:** a University of Alabama at Birmingham, Birmingham, AL, USA; b FBRI (Virginia Tech), Roanoke, VA, USA; c Florida International University, Miami, FL, USA

## Abstract

Mild traumatic brain injury affects millions of people every year in the world, increasing their likelihood of developing long-term consequences such as seizures or cognitive decline. Yet, the astrocyte response to this type of injury is still poorly understood. In a mouse model of mild diffuse traumatic brain injury, our lab identified a new astrocyte response characterized by the loss of expression of astrocyte proteins, such as Glt-1, Kir4.1, or Cx43, compromising their normal function. We called it atypical astrocyte response. Yet, how this response gets activated and how it affects the surrounding neurons remained unknown. We first observed that atypical astrocytes appeared near areas of blood-brain barrier (BBB) disruption, and we hypothesized that this BBB disruption was causing the atypical astrocyte phenotype. Using a mouse model of endothelial ablation and serum-free primary astrocyte cultures we determined that mouse serum is sufficient to induce a reduction in Glt-1 and Kir4.1 protein levels. We treated serum-free primary astrocyte cultures with inhibitors of different protein degradation pathways and we showed that astrocyte protein loss occurs through proteasome and calpain-dependent protein degradation. We finally studied neuronal function in areas with atypical astrocytes. We found that neurons lost NeuN, CamKII, PV or Homer1 expression in less than 5 min, while cells were still positive for Nissl staining. This indicates that also neurons acquire an atypical phenotype as early as 5 min after the injury. Interestingly, both the astrocyte and neuronal phenotype were maintained for at least six months after the injury, with no significant cell loss associated to them. Our findings indicate that blood-borne factors entry into the brain induce a fast protein decrease both in neurons and astrocytes, through calpain activation. While the functional impact of this phenotype still needs investigation, this mechanism might also be present in other diseases in which blood-brain barrier is compromised.

# Abstract: P01.094 – ER Stress Underlies Altered Cell Fate During Brain Development in a Human Model of the Cortical Malformation Syndrome Tuberous Sclerosis

**Published:** 2025-09-05

**Authors:** Shama Nazir, Lisa Lin, Kaitlyn Locke, Lisa Julian

**Affiliations:** a Simon Fraser University, Burnaby, Canada; b Centre for Cell Biology, Development, and Disease, Burnaby, Canada; c Institute for Neuroscience and Neurotechnology, Burnaby, Canada

## Abstract

**Introduction:**

Endoplasmic Reticulum (ER) stress has been implicated in numerous neurological disorders, including the cortical malformation syndrome Tuberous Sclerosis (TS). TS is a rare genetic disorder where patients present with foci of abnormally developed brain tissue called cortical tubers, which lead to epilepsy and other neurological pathologies like intellectual disabilities and autism spectrum disorder. TS is caused by inactivating mutations in TSC1 or TSC2, whose protein products normally inhibit the protein translation activator mTORC1. One potential inducer of ER stress is aberrant activation of mTORC1, which leads to increased protein synthesis and accumulation of unfolded protein. Excessive levels of unfolded proteins trigger the Unfolded Protein Response (UPR), a conserved pathway that functions to restore cell proteostasis. Altered expression of proteins that regulate the UPR has been observed in various neurological disorders. Recent findings indicate that UPR proteins also impact neural cell fate decisions, metabolism, and neuroinflammation, suggesting that ER stress and the UPR response play crucial roles in brain development.

**Objectives:**

My research aims to elucidate the intricate relationship between ER stress – UPR signaling and neural stem cell (NSC) fate decisions, particularly during altered brain development in TS, which typifies cortical malformation syndromes. We hypothesize that elevated UPR signaling leads to aberrant NSC identity determination by disrupting the delicate balance between NSC expansion and differentiation. I aim to identify specific UPR mediators that underlie aberrant brain development in TS.

**Methods:**

Human pluripotent stem cell (hPSC) lines carrying inactivating TSC2 mutations (TSC2-/-) and isogenic wild-type lines are induced into the neural lineage and subsequently into NSCs, neurons and astrocytes using standard protocols. To understand how ER stress during early brain development affects the identity and differentiation potential of neuronal cell types, chemical compounds are used to acutely activate and/or inhibit certain proteins of the UPR. Cell cultures are later characterized to determine impacts on cell differentiation and stress-related processes by neuronal morphology analyses, live cell dyes to organelles and immunostaining for expression of neural cell identity proteins (SOX2, PAX6, MAP2 GFAP and β-tubulin). Alterations in cell identity and organelle function are quantified using high-content imaging aided by Cell Profiler and our Nikon GA3 analysis software.

**Results:**

When induced towards the neural lineage, TSC2-deficient hPSCs show elevated ER stress. Subsequent UPR activation preferentially promotes survival of TSC2-/- NSCs, and these cells demonstrate increased accumulation of lysosomes, mitochondria and large vesicles mirroring the phenotype observed in individuals with TS. Upon further differentiation, TSC2-/- cells produce abnormal hypertrophic neurons and astrocytes and NSC-like giant cells, that populate cortical tubers in TS patients. Treatment with ER stress-inducing compounds during early neural induction exacerbates these cell fate alterations, with increasing levels of ER stress-UPR signaling during early brain development leading to increased spontaneous neurogenesis. We are also investigating the impact of ER stress on other processes like neuroinflammation by establishing hPSC-derived neural cells and microglia coculture models.

**Conclusion:**

The neural lineage is highly susceptible to proteostasis-ER stress during neural induction, which leads to aberrant, dose-dependent, pro-neurogenic fate decisions that reflect altered cortical development in TS.

# Abstract: P01.095 – CRISPR Inactivation Strategies for ALS, Charcot-Marie-Tooth, and Other Dominant Neurogenetic Diseases

**Published:** 2025-09-05

**Authors:** Zachary Nevin, Helen Sun, Jessica Stewart, Madeline Matia, Bria Macklin, Hannah Watry, Gokul Ramadoss, Luke Judge, John Svaren, Maurizio D’Antonio, Bruce Conklin

**Affiliations:** a Gladstone Institutes, San Francisco, CA, USA; b University of California San Francisco, San Francisco, CA, USA; c University of Wisconsin-Madison, Madison, WI, USA; d San Raffaele Scientific Institute, Milan, Italy; e Institute for Genomic Innovation, Berkeley, CA, USA

## Abstract

Targeted inactivation of a mutant allele could be therapeutic for many dominant genetic diseases. CRISPR/Cas systems have successfully been used to knockout disease alleles by targeting the causative mutation directly. However, for many diseases, this mutation-dependent approach will not be scalable due to a diversity of rare mutations, absence of PAMs, or poor gRNA discrimination between mutant and wildtype alleles. Instead, we have developed a CRISPR/Cas platform that takes advantage of single nucleotide polymorphisms (SNPs) to inactivate any mutant allele that occurs in cis with common SNPs. By this strategy, a single coding SNP can be targeted to create indels that knockout expression of the mutant allele, while pairs of non-coding SNPs can be targeted to excise the mutant gene entirely. Over 60 different dominant mutations in the gene fused in sarcoma (FUS) have been linked to amyotrophic lateral sclerosis (ALS) and frontotemporal dementia (FTD). However, we have identified two common coding SNPs whose four alleles can each be targeted by CRISPR/Cas9 gRNAs. Based on the prevalence of these SNPs across human populations, optimization of just four gRNAs could be therapeutic in up to 64% of patients. To quantify editing in a disease-relevant system, we engineered isogenic FUS mutations into a human iPSC line that is heterozygous at both SNPs, then tagged the endogenous FUS alleles with either GFP or HaloTag in order to track the wildtype and mutant proteins independently. CRISPR/Cas9 gRNAs targeting each of the four SNP alleles demonstrate efficient and specific generation of indels, with functional knockdown of mutant protein and prevention of disease phenotypes in iPSCs. We are currently optimizing lipid nanoparticle delivery of Cas9 and gRNA directly to iPSC-neurons and rodent models in order to asses editing and rescue of disease phenotypes in a neuron-specific context. Mutations in PMP22 and MPZ are leading causes of a set of genetic peripheral neuropathies collectively called Charcot-Marie-Tooth disease. Although there are no coding SNPs in these genes, we have identified common intronic and intergenic SNPs in both humans and mice and have begun to generate excisions between pairs of gRNAs to inactivate or excise entire mutant alleles. In initial mouse studies, we are testing CRISPR/Cas9 excisions both in dorsal root ganglion explants and by intrathecal lipid nanoparticle delivery in vivo. Although an individual mouse strain is inherently homozygous, crossing two strains results in F1 pups that are heterozygous at every polymorphic locus between the strains, introducing multiple polymorphisms around PMP22 and MPZ. In upcoming human studies, we will isolate cadaveric Sciatic nerve Schwann cells in order to model the diversity of human SNPs and test pairwise combinations of SNP-gRNA, while also generating iPSC-Schwann cells from lines with known mutations to test phenotypic rescue by lead gRNA excision pairs. Together with new bioinformatics algorithms capable of mapping all SNP-gRNA around a given gene, these studies demonstrate new strategies for CRISPR gene inactivation with broad applications to dominant disease.

# Abstract: P01.096 – CK2 Inhibition Preconditions White Matter Against Ischemia by Differentially Regulating CDK5 and AKT/GSK3β Pathways

**Published:** 2025-09-05

**Authors:** Sarah Zerimech, Hung Nguyen, Hamzah Sawab, Isabela Harmon, Selva Baltan

**Affiliations:** a Oregon Health and Science University, Portland, OR, USA

## Abstract

Approaches to induce tolerance in the brain against ischemia have gained new interest in clinical and experimental studies. However, the mechanisms of ischemic preconditioning (IPC) have been primarily studied in gray matter (GM), despite white matter (WM) injury and axon dysfunction being critical components of the clinical deficits observed in stroke patients. Most in vivo rodent models of IPC consist of short episodes of hypoxia-ischemia and therefore are not applicable as therapeutic options. Pharmacologically induced IPC would be easier to translate into clinical therapeutic settings. We reported that Casein Kinase 2 (CK2) is upregulated during ischemia mediating injury acting via Cdk5 and AKT/GSK3β signaling in young and aging WM. Selective and specific CK2 inhibition using CX-4945 (Silmitasertib), a Food and Drug Administration (FDA) approved drug for cancer patients, exerts prominent functional recovery in WM when administered during and after ischemia. This protection shows a spatiotemporal specificity such that Cdk5 inhibition protects WM during ischemia while AKT/GSK3β inhibition confers post-ischemic protection. Therefore, in this study using specific inhibitors we investigated whether CK2 inhibition can precondition WM and whether this benefit is mediated via a specific signaling pathway as a function of sex and age. Isolated mouse optic nerves (MONs), pure myelinated WM tracts, were obtained from male and female Thy1 CFP + mice at 2–4-month-old (young), and 12–14-month-old (aging). MONs were placed at the interface in a Haas chamber, perfused with artificial cerebrospinal fluid (ACSF) at 37 °C, and saturated with 95%O_2_/5%CO_2_. Extracellular compound action potentials (CAPs) were evoked every 30 seconds. Axonal responses were recorded under baseline conditions for at least 1 h. Ischemia was induced by switching to glucose-free ASCF saturated with 95%N2/5%CO_2_ for 1 h, followed by 5 h of recovery. CX-4945 (5 μM) was applied for 30 min before ischemia. We found that CK2 inhibition using CX-4945 effectively preconditioned axon function in young and aging male and female MONs. Selective inhibition of Cdk5 with Roscovitine (5 μM) equally preconditioned axon function in all age groups and sexes. Roscovitine preconditioning for aging axons in males was the most effective compared to other groups. Moreover, preconditioning with Roscovitine in young and aging females was less compared to males. Interestingly, inhibition of AKT/GSK3β with ARQ-092 (500 nM) selectively preconditioned axon function in young males but not in young females. Even more surprising was that AKT/GSK3β inhibition exerted protection to aging female axons. The effects of AKT/GSK3β inhibition in aging axons in males is still under investigation. We conclude that CK2 inhibition preconditions WM by differentially regulating the Cdk5 and AKT/ GSK3β signaling pathways as a function of age and sex. The downstream cellular targets of this protection are currently being studied.

# Abstract: P01.097 – The Role of NOX in Post-Ischemic Protection of White Matter Against Ischemia

**Published:** 2025-09-05

**Authors:** Hung Nguyen, Isabela Harmon, Martin Pike, Selva Baltan

**Affiliations:** a Oregon Health and Science University, Portland, OR, USA

## Abstract

Improved stroke care has increased the number of stroke survivors with long-term neurological deficits and disabilities. White matter injury (WMI) contributes to the majority of clinical deficits observed in stroke patients. However, a scientific gap remains in targeting WM functional recovery due to limited in vivo WMI models in rodents. Therefore, in this study, we have developed an in vivo selective focal WM injury (sfWMI) model by injecting L-NIO specifically into the subcortical WM. We showed that ischemia upregulates Casein Kinase 2 (CK2) causing WMI through mitochondrial dysfunction via activating Cdk5 and AKT/GSK3β pathways. Consequently, CX-4945, an FDA-approved, selective, and specific CK2 inhibitor that crosses the blood-brain barrier, confers post-ischemic protection by conserving mitochondria. Because prior studies reported that ischemia activates NADPH oxidase (NOX) in neurons to increase oxidative stress leading to mitochondrial dysfunction, we hypothesized that CK2 mediates WMI injury by activating NOX leading to mitochondrial dysfunction. Since stroke is a dimorphic disease, using the sfWMI model, we investigated the role of NOX as a function of sex while correlating histological, behavioral, and spatial-temporal WM changes using MRI. Using 8-week-old C57BL/6 male and female mice, three injections each of 200 nL of L-NIO (130 µM) to create focal ischemic strokes or saline for sham were deposited at earlier identified coordinates. Six hours after WMI, the treatment group was administered CX-4945 (75 mg/kg) and the control group was administered saline for 5 days, twice daily. Behavioral deficits were assessed using a cylinder test, and a pasta-eating test at baseline, days 1, 7, 21, and 28 post-injuries. T2-weighted imaging and diffusion tensor imaging (DTI) of the mice were taken on days 2 and 10 post-injury to assess WM changes in the corpus callosum (CC). The loss of WM integrity was characterized by oligodendrocyte death, myelin damage, astrocyte and microglia activation and upregulation of NOX activity correlated with impaired bilateral paw use in cylinder tests and dexterity in pasta-eating tests in male and female mice. These deficits corresponded with edema formation and changes in fractional anisotropy in MRI imaging modalities. Application of CX-4945 preserved WM integrity histologically, alleviated behavioral deficits, attenuated NOX activity, and improved WM structure in MRI. Our findings indicate sfWMI in male and female rodents causes significant damage to axon integrity which correlates with behavioral deficits, upregulation of NOX activity, and altered WM organization in MRI. Moreover, CX-4945 exerts post-ischemic protection of axons, oligodendrocytes, and myelin, effectively alleviating behavioral deficits, and decreasing NOX activity with improved WM integrity observed with MRI images. Our long-term goal is to establish that CK2 inhibition after stroke confers post-ischemic WM protection by regulating NOX activity to preserve mitochondria.

# Abstract: P01.098 – Herpes Simplex Virus Type-1 (HSV-1) Accelerates Alzheimer’s Disease Progression: Targeting Vulnerable Brain Regions and Amplifying Neuroinflammation

**Published:** 2025-09-05

**Authors:** Christy Niemeyer, Laetitia Merle, Andrew N. Bubak, B. Dnate Baxter, James E. Hassell, Maria Nagel, Diego Restrepo

**Affiliations:** a Department of Neurology, School of Medicine, University of Colorado, Aurora, CO, USA; b Department of Cell and Developmental Biology, School of Medicine, University of Colorado, Aurora, CO, USA; c Department of Ophthalmology, School of Medicine, University of Colorado, Aurora, CO, USA; d Department of Psychology, Texas A&M University, College Station, TX, USA; e Centre des Sciences du Goût et de l’Alimentation, CNRS, INRAE, Institut Agro, Université de Bourgogne, Dijon, France

## Abstract

Multiple studies provide evidence that herpes simplex virus type-1 (HSV-1) may contribute to Alzheimer’s disease (AD) progression. HSV-1 increases the risk of dementia, and infection shares similar pathological characteristics as those seen in AD, including amyloid accumulation, neuroinflammation, neurodegeneration, and cognitive impairment. Early AD is characterized by deficits in multiple brain regions before the onset of memory deficits and hippocampal pathology. These brain regions include degeneration in brainstem centers such as the locus coeruleus (LC) and regions within the hypothalamus (HYP). Our data found infection of HSV-1 to target these regions in both control (C57BL/6) and AD transgenic (5xFAD) mice. This raises the possibility that HSV-1 disruption of these vulnerable regions may accelerate AD pathogenesis early on. Thus, we hypothesize that HSV-1 infection in these regions accelerates pathological processes characteristic of early AD. To test this hypothesis, we determined if HSV-1 can infect the brains of control and AD mice. Mice were intranasally inoculated with HSV-1 (10e6 PFU/animal, McKrae strain) or PBS. Five days post-infection (DPI), immunohistochemical analysis revealed HSV-1 antigen spread to distinct brain regions, including the LC, HYP, and other brain stem regions in C57BL/6. Analysis of microglial activation was assessed in C57BL/6 and regional differences in microglial activation. These results suggest that HSV-1 infection triggers a region-specific immune response within the brain, which might contribute to the virus’ differential impact on various CNS disorders. Additionally, these findings shed light on HSV-1’s unique mode of spreading within the CNS, unlike other neurotropic viruses, suggesting a targeted rather than indiscriminate dissemination pattern. Under the same infection paradigm in the 5xFAD mice, we found had similar patterns of infection as wild types. Furthermore, we found an increase in amyloid in regions positive for HSV-1 antigen. These findings suggest that HSV-1 can accelerate neuroinflammation and amyloid production in vulnerable regions of the brain, potentially accelerating AD pathology.

# Abstract: P01.099 – The mGluR5 Agonist, CHPG, Enhances Differentiation of Developing Human Oligodendrocyte Lineage Cells

**Published:** 2025-09-05

**Authors:** Hiroko Nobuta, Yangyang Huang, Anjalika Bandaru, Cheryl Dreyfus

**Affiliations:** a Rutgers University, Piscataway, NJ, USA

## Abstract

Previous studies on adult mice indicate that the mGluR5 agonist, CHPG, reverses cuprizone-elicited losses in myelin. It does so in part by binding to mGluR5 on reactive astrocytes and eliciting release of astrocyte-derived BDNF. BDNF binds to TrkB receptors on oligodendrocytes (OLs). This results in an increase in myelin proteins and myelin, and a reversal of behavioral deficits. However, mGluR5 is also found on young OLs, suggesting that direct effects on these cells may play a role. Using human induced pluripotent stem cell (hiPSC)-derived OLs and primary human fetal brain cells in the present study we examined effects of CHPG and identified the target of CHPG action. Treatment of hiPSCs (30uM, 5 days) or fetal cells (30uM, 3 days) with CHPG increases the numbers of processes extending from the OL cell body and increases the number of O4+/MBP + cells relative to total O4 cells. O4 is a marker of immature-mature OL lineage cells while MBP is a marker of mature OLs. These data suggest that CHPG increases differentiation. The effect of CHPG is blocked by MPEP, a mGluR5 antagonist. However, in contrast to observations seen in the adult cuprizone mouse model, mGluR5 is expressed on young OLs and not astrocytes in human cultures. Our studies suggest that CHPG affects developing human cells to enhance differentiation, but the cellular mechanism may differ from that noted in adult cuprizone-treated mice. This work was supported by the Department of Defense through the Multiple Sclerosis Research Program under Award No. W81XWH-22-1-0788. Opinions, interpretations, conclusions, and recommendations are those of the authors and are not necessarily endorsed by the Department of Defense.

# Abstract: P01.100 – Microglial Lipoprotein Lipase Regulates Myelin Processing and Is Elevated in Multiple Sclerosis

**Published:** 2025-09-05

**Authors:** Dean Oldham, Mikaela Neal, Garrett Potter, Maoping Tank, Sachi Gorkhali, Katherine Given, Wendy Macklin, Robert Eckel, Kimberley Bruce

**Affiliations:** a University of Colorado Anschutz Medical Campus, Aurora, CO, USA

## Abstract

Multiple sclerosis (MS) is the most common neurodegenerative disorder among young adults and is characterized by myelin damage and poor repair. Despite significant efforts to understand MS pathogenesis, reparative therapeutics are lacking. Since microglia clear damaged myelin, facilitating repair, they present a promising target for developing new MS treatments. However, microglial factors that contribute to myelin uptake remain elusive. Lipoprotein lipase (LPL) – the rate-limiting enzyme in lipid processing – is predominantly expressed in microglia and is profoundly upregulated during de- and remyelination (Hammond et al., 2019). Previously, we have shown that LPL is a feature of reparative microglia and is directly involved in microglial lipid and lipoprotein processing (Bruce et al., 2018; Loving et al., 2021). Therefore, we hypothesize that microglial-LPL is also involved in processing myelin-derived lipids. To investigate this, we adopted a multifaceted approach. Microglial-specific LPL knockdown (MiLPLKD) mice were subjected to the Experimental Autoimmune Encephalomyelitis (EAE) challenge, which models relapsing-remitting MS. We found increased MS-like symptom scores, poorer recovery, and metabolic changes in MiLPLKD mice compared to WT mice, suggesting that microglial LPL is involved in myelin clearance and remyelination. We developed an in vitro phagocytosis assay using fluorescently labeled crude myelin (carboxyfluorescein succinimidyl ester; CFSE) to measure LPL deficient microglia and phagocytosis. CRISPR-Cas9 generated LPL KO BV-2 murine microglia demonstrated reduced myelin phagocytosis compared to WT cells, suggesting that LPL was directly involved in myelin uptake. These observations were recapitulated with cells in the presence of the endogenous LPL inhibitor angiopoietin protein-like 4 (Angptl4), or the endogenous LPL activator apolipoprotein C-II (ApoCII), which decreased, and increased the uptake of myelin debris, respectively. In further support, LPL + microglia isolated from whole murine brains abundantly express (200-fold) other myelin processing factors such as triggering receptors expressed on myeloid cells-2 (TREM2). Lastly, we found that LPL activity was elevated in human cerebral spinal fluid from MS patients (MS-CSF) vs. a headache control (HA-CSF). Overall, our data indicate that LPL is a feature of phagocytic, myelin-processing microglia and that microglial-LPL is a rational target for developing novel reparative therapeutics for MS.

# Abstract: P01.101 – TFEB Treatment in the Dorsal Hippocampus of Obese Female and Male 5xFAD Mice

**Published:** 2025-09-05

**Authors:** Danielle Osborne, Adrianah Dorn

**Affiliations:** a Legacy Research Institute, Portland, OR, USA

## Abstract

In both clinical patients and animal models of Alzheimer’s disease (AD), lysosomal dysfunction is among the earliest detectible cellular changes. Major risk factors for AD include obesity and female sex; however, how those variables affect lysosomal function in the hippocampus remains poorly understood. TFEB, a major transcription factor that coordinates autophagic and lysosomal functions in the cell, has been used previously to counter AD pathology and cognitive decline in male mouse models of AD. We investigated whether TFEB gene therapy would improve behavioral and cellular outcomes in 5xFAD mice, with induced obesity and when accounting for sex differences. Female and male 5xFAD mice were placed on an obesogenic diet at six weeks of age, at five months of age we infused an AVV with mutated TFEB serine142, designed to prevent TFEB phosphorylation and enhance translocation to the nucleus. One month later, mice were behaviorally tested in Morris watermaze and tissues were collected. Female and male mice diverged greatly in their responses to TFEB therapy. TFEB therapy improved learning in non-obese 5xFAD female mice, an effect not observed in male mice; indeed, male wildtype mice treated with TFEB did more poorly than 5xFAD males. In contrast to non-obese males, obese wildtype males moderately benefitted from TFEB treatment, while the addition of obesity with 5x mutations was more than TFEB could overcome. Molecular analyses are still ongoing, but so far western blots indicate that TFEB treatment affects pAkt and mTOR signaling, with comprehensive qPCR analysis pending. The results demonstrate that females and males respond very differently to therapeutic approaches, that obesity worsens outcomes in AD and limits therapeutic interventions, and confirms that upregulating lysosomal activity early in AD progression presents a promising approach to preventing behavioral manifestations of the severe phenotype observed in 5xFAD mice.

# Abstract: P01.102 – Aligned Nanofibrillar Collagen Scaffolds Promote Repair Schwann Cell Phenotype in Peripheral Nerve Regeneration

**Published:** 2025-09-05

**Authors:** Ethan Oseas, Cynthia Alcazar, Theo Seah, Owen McCarty, Karina Nakayama

**Affiliations:** a Oregon Health and Science University, Portland, OR, USA

## Abstract

Despite advances in tissue engineering and treatment strategies that mediate complication risk, nerve autograft remains the clinical standard for the treatment of nerve injuries exceeding 1 cm. While the peripheral nervous system (PNS) has some capacity to regenerate after injury, largely due to the plasticity of resident Schwann cells (SCs), endogenous repair programs are often insufficient to fully heal injured nerves. Biomaterial scaffolds have been widely applied as nerve guidance conduits in these cases and have demonstrated the ability to promote regeneration. Our group has demonstrated robust cytoskeletal guidance of a diverse range of cell types including myogenic, osteogenic, and endothelial cells using collagen scaffolds with aligned nanofibrillar topography. In the current study, we extend these concepts of cell-material guidance to hypothesize that topographically aligned nanofibrillar collagen scaffolds promote a repair phenotype in SCs. To investigate this, the SC migratory ability and morphology on scaffolds was evaluated. Briefly, aligned nanofibrillar collagen scaffolds were fabricated by shear extrusion controlling for pH and temperature during fibrilogenisis, while control (random topography) scaffolds were extruded onto slides and submerged in neutral buffer solution to undergo delayed fibrilogenesis. Whole sciatic nerves of C57BL/6 mice were explanted directly onto scaffolds and SC outgrowth was monitored for 12 days in culture. SCs adopt a regenerative phenotype after injury characterized by an elongated morphology with longer, more numerous protrusions and higher motility. SCs on topographically aligned scaffolds migrate 24% further compared to those on randomly patterned scaffolds. In addition, morphological characteristics indicative of a repair phenotype were observed in SCs on aligned scaffolds. SCs had more protrusions on aligned scaffolds (*p* < 0.05), with an average of 2.4 protrusions, compared to 1.8 protrusions on randomly patterned scaffolds. Interestingly, actin fluorescence intensity was lower on aligned scaffolds as compared to random scaffolds, with a mean cell total corrected fluorescence of 5.8E5 and 3.46E6, respectfully (*p* < 0.05). Initial data also suggests that circularity was lower on aligned scaffolds, suggesting a higher degree of cell elongation characteristic of repair SCs. Together, these findings indicate a higher instance of a repair phenotype in SCs on the aligned scaffolds, suggesting that scaffolds with nanofibrillar aligned patterning have the potential to support peripheral nerve regeneration. Supporting and enhancing SC’s regenerative capability is paramount to engineering tissues for PNS repair applications. Initial in vitro experiments show this capability in nano-aligned scaffolds. Future in vivo experiments will examine the potential regenerative effects of implanted nanofibrillar collagen scaffolds in a rat model of sciatic nerve gap injury. This study furthers our understanding of the contribution which topography, specifically, nano aligned fibrillar collagen scaffolds, have in triggering a repair phenotype in SCs. By supporting the resident SCs or seeding with patient derived induced SCs, this material has the potential to improve existing peripheral nerve repair strategies and reduce the need for allografts and autografts in PNS injury.

# Abstract: P01.103 – Region Specific Inflammatory Response in Cerebrovascular Patients

**Published:** 2025-09-05

**Authors:** Ms Jacqueline Frank, Benton Maglinger, David Dornbos, Hunter Hazelwood, Jordan Harp, Shivani Pahwa, David Dornbos, Amanda Trout, Will Crawford, Ann Stowe, Justin Fraser, Keith Pennypacker

**Affiliations:** a University of Kentucky, Lexington, KY, USA; b Beth Israel Deaconess Medical Center, Boston, MA, USA

## Abstract

The objective of this study was to identify proteomic biomarkers predictive of stroke outcomes specific to subjects based on locality. Given the demographic diversity our Comprehensive Stroke Center serves, encompassing both Appalachian and non-Appalachian cohorts. Appalachia is marked by health disparities, especially in ischemic stroke, and represents an underserved population. Our research endeavors aim to discern proteomic biomarkers that could prognosticate stroke outcomes with specificity to individuals residing in Appalachia. Eighty-one stroke and forty-six cerebrovascular control participants met the inclusion criteria for this investigation. These individuals either underwent mechanical thrombectomy (MT) for emergent large vessel occlusion (ELVO), or a diagnostic or procedural angiogram for other cerebrovascular disease. During the procedure, blood samples from the carotid artery were obtained and subsequently subjected to proteomic analysis for 184 cardiometabolic and inflammatory proteins using Olink Proteomics. The processing of samples adhered to the protocols outlined by the Blood And Clot Thrombectomy Registry And Collaboration (BACTRAC; clinicaltrials.gov; NCT 03153683). Statistical analyses were employed to assess whether the associations between protein expression and outcomes varied based on Appalachian status, specifically for functional outcomes (NIH Stroke Scale; NIHSS and Modified Rankin Score; mRS), cognitive outcomes (Montreal Cognitive Assessment; MoCA), and mortality in the stroke group. No significant differences were found in demographic data nor co-morbidities when comparing Appalachia to non-Appalachia subjects. Time from stroke onset to treatment (last known normal) was significantly longer in patients from Appalachia. In the cerebrovascular control group, only interleukin 1 alpha (IL-1alpha) was elevated in the Appalachian cohort while 33 proteins including IL-1alpha were significantly expressed between Appalachian and non-Appalachian patients during stroke. Comparison of Appalachia to non-Appalachian stroke subjects revealed significant differences in functional/cognitive outcomes including NIHSS, MoCA, mRS, as well as neuroradiographic outcomes including infarct volume and edema volume Seven proteins that differed on NIHSS, fourteen associated MoCA, six related to mRS, and seven proteins related to mortality that were significantly different based on the residency of the patient. All these proteins were differentially correlated with these functions dependent on whether the patient was from Appalachia or non-Appalachian county using last known normal as co-variate. Within the Appalachian subgroup, the protein multiple epidermal growth factor-like domains protein 9 (MEGF9) exhibited a positive correlation with discharge NIHSS. Elevated levels of coagulation factor XI (F11) and mannose-binding protein (MBL2) were linked to an increased likelihood of worse mRS. No proteins were identified in the Appalachian cohort that were predictive of worse MoCA scores or increased mortality. Our study, leveraging an ELVO tissue bank and registry, explores proteomic expression during MT. Patients from Appalachian regions exhibit a distinct proteomic response to stroke compared to non-Appalachian patients, with these proteins correlating with stroke outcomes. These differentially expressed proteins may serve as prognostic biomarkers or targets for novel therapies. The disparate proteomic response in Appalachian patients to stroke suggests a link to environmental exposures. Many of these proteins, such as IL-1alpha, have been linked to lung pathologies emphasizing the need for further community-based studies to uncover the underlying causes of this distinct response.

# Abstract: P01.104 – Physiologic, Biomarker and Clinical Outcomes Associated With Ketamine Exposure After Traumatic Brain Injury, a Single Center Retrospective Study

**Published:** 2025-09-05

**Authors:** Austin Peters, Saad Khan, Rami Kim

**Affiliations:** a Oregon Health and Science University, Portland, OR, USA

## Abstract

**Introduction:**

Ketamine has historically been contraindicated for use in traumatic brain injury (TBI) out of concern for its effects on intracranial pressure and metabolism (Shaprio et al., 1972). Because of ketamine’s unique properties as an anesthetic induction agent, interest in its use in TBI has persisted and it is increasingly being utilized after head injuries (Peters et al., 2023). We sought to characterize the demographics of TBI subjects receiving ketamine at our institution and compare them to subjects who did not receive ketamine to assess if there were baseline differences in these groups, and then compare available physiologic measurements, biomarker profiles, and clinical outcomes.

**Methods:**

A retrospective analysis was performed on all subjects enrolled in a separate study examining the effect of TBI on proteomic biomarker responses. In the primary study, 128 TBI subjects with a documented intracranial hemorrhage were enrolled between August 2020 and December 2022, with detailed demographic (including Glasgow Coma Scale (GCS), vital signs (including intracranial pressure (ICP), systolic blood pressure (SBP) and mean arterial pressure (MAP)), admission proteomic biomarker, and subject outcomes (via Glasgow Outcomes Scores (GOS)) available. We performed a chart review of each subject to assess whether ketamine was administered, and then ketamine-exposed and ketamine-unexposed subjects were compared via logistic regression analysis across all variables.

**Results:**

Of 128 subjects, 16 (12.5%) were exposed to ketamine. Baseline demographics including age, sex, weight and initial GCS were similar between groups. Both groups had similar temperature, oxygen saturation and ICP measurements. The ketamine-exposed group had significantly lower SBP and MAP compared to the unexposed group. There were no differences in GOS between groups. Biomarker analysis is currently pending.

**Conclusion:**

At our institution, 12.5% of TBI subjects were exposed to ketamine, with no underlying demographic differences between groups. Ketamine exposure was associated with a significantly lower initial blood pressure measurements and no differences in ICP or outcomes between groups. A prospective study is needed to further clarify differences in vital signs changes when ketamine is administered following TBI.

## References

[CIT0001] Shaprio, H. M., Wyte, S. R., & Harris, A. B. (1972). Ketamine anaesthesia in patients with intracranial pathology. *British Journal of Anaesthesia*, *44*(11), 1200–1204.4647115 10.1093/bja/44.11.1200

[CIT0002] Peters, A. J., Khan, S. A., Koike, S., Rowell, S., & Schreiber, M. (2023). Outcomes and physiologic responses associated with ketamine administration after traumatic brain injury in the United States and Canada: a retrospective analysis. *Journal of Trauma and Injury*, *36*(4), 354–361. 10.20408/jti.2023.003439381569 PMC11309261

# Abstract: P01.105 – Voltage-Gated Calcium Channels Regulate Developmental Myelination in Oligodendrocytes

**Published:** 2025-09-05

**Authors:** Melanie Piller, Stephen Smith, Kelly Monk

**Affiliations:** a Oregon Health and Science University, Portland, OR, USA; b Vollum Institute, Portland, OR, USA; c VA Portland Healthcare System, Portland, OR, USA

## Abstract

Oligodendrocyte lineage cells (OLCs) are glial cells of the central nervous system (CNS) that arise as oligodendrocyte progenitor cells (OPCs) and may persist as progenitors or differentiate into myelin-producing oligodendrocytes. OLCs are sensitive to neuronal activity, and OPCs receive synaptic input from neurons from various receptors, which can affect calcium signaling. Calcium influx in OLCs has been shown to regulate survival, differentiation, and myelination. OLCs possess P/Q-type voltage-gated calcium channels that can contribute to OLC calcium influx, but the functional significance of these channels is not well understood. Understanding P/Q-type channels is critical because mutations in these channels have been implicated in several neurological disorders, including episodic ataxia, epilepsy, and familial hemiplegic migraine. In this study, we employ zebrafish to investigate the role of OLC P/Q-type channels in vivo during development. We use global and cell-type specific CRISPR/Cas9-mediated genome editing approaches in conjunction with live imaging, as well as whole-cell patch clamp electrophysiology of OPCs in the zebrafish spinal cord. We found that these P/Q-type channels are required for normal myelination in the developing CNS; mutants present with reduced myelin in the dorsal spinal cord and produce atypical myelin structures associated with sheath retraction during developmental myelination. These findings provide new insight into the signaling landscape in the developing CNS that regulates oligodendrocyte myelin formation.

# Abstract: P01.106 – Loss of Carnitine Palmitoyl Transferase 2 (CPT2) Enhances Proliferation Post Traumatic Brain Injury in Adult Mice

**Published:** 2025-09-05

**Authors:** Joseph Scafidi, Regina Fernandez, Noelle Puleo, Susanna Scafidi

**Affiliations:** a Kennedy Krieger Institute, Baltimore, MD, USA; b Johns Hopkins University School of Medicine, Baltimore, MD, USA

## Abstract

Neurogenesis in the dentate gyrus of the hippocampus is conserved and highly regulated throughout the lifespan. It relies on neural stem cells – radial glia-like cells (RGL) – to activate resulting in proliferation or remain in quiescent state. Accumulated data suggests that Fatty acid oxidation (FAO) is important in the regulation of these RGL activity.

Severe TBI is characterized by increased proliferation in the hippocampus, however, the extent of proliferation in the hippocampus following mild TBI (mTBI) is not fully delineated. Furthermore, what is the role of FAO in progenitor cells following the injury remains unknown. We hypothesized that: 1) loss of FAO via genetic deletion of mitochondrial carnitine palmitoyl transferase 2 (Cpt2) will activate hippocampal neurogenesis and 2) will be further affected post-traumatic proliferation by activating RGL.

**Methods:**

9–10 weeks old male and female Nestin-Cre/Cpt2 f/f and Cpt2 f/f were injected with BrdU (50 mg/kg ip x 3injections) and subsequently perfused for histological evaluation. Separate subsets of mice were subjected to a concussion model of TBI – a traumatic impact on the intact skull over the midline suture at 1.5 mm depth using a pneumatic CCI device. BrdU (50 mg/kg ip x 3injections) was administered at different times pre- (24 hr, 28 days) and post injury (1 day, 3 days, 7 days and 28 days). Immunohistochemical analysis was performed and sections were analyzed using Imaris.

**Results:**

Naïve Nestin-Cre/Cpt2 f/f and Cpt2 f/f mice did not show significant differences in BrdU(+)/Ki67 (+) cells in DG. Following mTBI there was an increase in proliferation, which peaked on day 3 post-injury. Nestin-Cre/Cpt2 f/f that underwent mTBI had significantly higher number of BrdU(+)/Ki67(+) cells in DG compared Cpt2 f/f (control) mice. Interestingly, TBI-injured males in both Nestin-Cre/Cpt2 f/f and Cpt2 f/f groups had significantly lower number of quiescent cells quantified by Sox2(+)/GFAP(+) in the subgranular zone compared to TBI-injured females.

**Conclusion:**

Our results show that loss of Cpt2 causes a further increase in proliferation in the hippocampus following mTBI in both sexes and peaks on day 3 post-injury. Current studies aimed to delineate whether loss of CPT2 improves long term survival and neurogenesis are in progress.

# Abstract: P01.107 – Oxidative Stress Triggers Secretion of Neurotoxic Ceramide-Rich Extracellular Vesicles (CREVs)

**Published:** 2025-09-05

**Authors:** Zainuddin Quadri, Ahmed Elsherbini, Simone M. Crivelli, Salim S. El-Amouri, Priyanka Tripathi, Zhihui Zhu, Xiaojia Ren, Liping Zhang, Stefka D. Spassieva, Mariana Nikolova-Karakashian, Erhard Bieberich

**Affiliations:** a University of Kentucky, Lexington, KY, USA

## Abstract

The plasma membrane serves as one of the pathways for the release of extracellular vesicles (EVs) through shedding, but the mechanisms governing their regulation and functions are not well understood. Using incubation of Hela cells with H_2_O_2_ as a well-established model for oxidative stress, we discovered that H_2_O_2_ led to a ∼3-fold increase in filopodia formation and a ∼5-fold increase in EV secretion. These EVs exhibited an enrichment of the sphingolipid ceramide, aligning with increased punctate labeling of ceramide observed in the plasma membrane of filopodia. This supports the notion that filopodia may serve as the origin for oxidative stress-induced EVs.Ceramide labeling of filopodia colocalized with labeling for acid sphingomyelinase (ASM) and neutral sphingomyelinase 2 (nSMase2), two enzymes activated by oxidative stress and generating ceramide at the plasma membrane. This colabeling was also observed with filopodia of fibroblasts and reactive astrocytes, suggesting a general mechanism of oxidative stress-induced EV secretion in different cell types. The inhibitors GW4869 and Arc39, which specifically target nSMase2 and ASM, respectively, led to a 1.4-fold and 2.63-fold decrease in the number of EVs. This underscores the crucial involvement of both SMases in the generation of ceramide under oxidative stress and the subsequent release of ceramide-rich extracellular vesicles (CREVs). At filopodia, nSMase2 was S-palmitoylated and interacted with ASM to generate ceramide for the shedding of CREVs. CREVs contained nSMase2 and ASM, resulting in decreased levels of these enzymes in Hela cells. When taken up by neuronal cells, CREVs transported ceramide to mitochondria, ultimately triggering cell death. In summary, oxidative stress induces the interaction of nSMase2 and ASM at the plasma membrane, leading to the release of CREVs that target mitochondria and induce cell death. Since a similar mechanism of ceramide generation is active in astrocytes, we are now investigating if oxidative stress induces secretion of neurotoxic CREVs in the brain.

# Abstract: P01.108 – The Impact of Reduced Glial Plasma Membrane Cholesterol on Subcellular Actin Dynamics

**Published:** 2025-09-05

**Authors:** Athira R. K, Victoria Defilippi, Yeon Hwa Woo, Lucia Notterpek

**Affiliations:** a University of Nevada, Reno, Reno, NV, USA

## Abstract

Hereditary peripheral demyelinating neuropathies are characterized by deficits in myelin formation and maintenance, leading to impaired nerve function. Genetic perturbations in Schwann cell proteins are responsible for these disorders. Understanding the interactions between disease-linked glial proteins and cholesterol, an essential and rate-limiting lipid for myelin biogenesis, is a topic of significance. Our previous studies demonstrated that the absence of peripheral myelin protein 22 (PMP22), a neuropathy-causing Schwann cell protein, disrupts cholesterol trafficking and alters the lipid moiety of the Schwann cell plasma membrane (PM). PMP22 is critical for cholesterol metabolism by partnering with the ATP-binding cassette transporter A1 (ABCA1) and a functional cholesterol recognition motif. Building upon this knowledge, we examined the relationship between the deficiency in glial PM cholesterol and demyelinating neuropathy in the present study, focusing on actin dynamics, a key mechanism in myelin formation. Sciatic nerves and cultured Schwann cells were used from genotyped wild type (Wt) and PMP22-deficient mice and normal rats. To mimic the reduced cholesterol content of the PMP22-deficient glial PM in normal rat Schwann cells, we employed a pharmacological approach and inhibited de novo cholesterol synthesis by simvastatin treatment (5 µM and 25 µM). In independent studies, PM cholesterol was acutely depleted by exposure of the cells to methyl-β cyclodextrin (MBCD; 3 mM). By biochemical protein analyses and high-resolution confocal imaging, we detected a significant (*p* < 0.001) increase in the abundance of filamentous actin (F-actin) compared to monomeric actin (G-actin) in cells with depleted PM cholesterol. In addition, statin exposure induced pronounced morphological changes in Schwann cells, leading to branching of the lamellipodia. Western blots of protein lysates from control and drug-treated cultures revealed an increase in the expression of essential proteins involved in cholesterol uptake and recycling, including low-density lipoprotein receptor (LDLR), ABCA1, and Rab11a. The bioactivity of simvastatin was confirmed by a decrease in the expression of 3-hydroxy-3-methyl-glutaryl coenzyme A reductase (HMGCR) and filipin-cholesterol labeling. Studies in cultured mouse Schwann cells uncovered a significant (*p* < 0.01) alteration in F- and G-actin ratios in PMP22-deficient samples, compared with Wt, along with collapsed lamellipodia and dense F-actin bundles. Nerve lysates from 3-week-old PMP22-deficient mice contained elevated levels of F-actin, compared with Wt. By confocal imaging, we detected a pronounced accumulation of F-actin and G-actin in nerve tomacula. The findings from the neuropathic samples were substantiated by unbiased proteomic studies, which showed significant alterations in the expression of proteins involved in cholesterol uptake (*p* < 0.05) and perturbations within pathways associated with actin polymerization and depolymerization. Together, these findings provide novel insights for unraveling the connection between the reduced PM cholesterol of neuropathic Schwann cells and subcellular alterations in actin dynamics. Furthermore, these results offer fundamental knowledge for understanding the subcellular mechanisms underlying the myelin defects in PMP22-linked neuropathies.

# Abstract: P01.109 – Store-Operated Calcium Entry-Dependent Signaling in Adult OPCs Acts to Delay Oligodendrocyte Differentiation Following Demyelination

**Published:** 2025-09-05

**Authors:** Roopa Ravichandar, Rupadevi Muthaiah, Darpan Saraswat, Farah Gadelkarim, Richard Seidman, Ranjan Dutta, Fraser Sim

**Affiliations:** a University at Buffalo, Buffalo, NY, USA; b Cleveland Clinic Lerner College of Medicine, Cleveland, OH, USA

## Abstract

One of the hallmarks of multiple sclerosis (MS) is inefficient/failed remyelination that contributes to neurodegeneration. Inhibition of M1/3R in oligodendrocyte progenitor cells (OPCs) promotes differentiation and remyelination. However, the operant downstream mechanisms are poorly understood. Gα/q receptor activation triggers endoplasmic reticulum Ca^2+^ release and replenishment via store operated calcium entry (SOCE) modulated by Ca^2+^ sensors, namely STIM1 and STIM2. Our previous gain-of-function studies in human OPCs showed that specific optogenetic activation of SOCE acts to reduce differentiation in vitro. As such, we hypothesize that OPC SOCE acts to delay or prevent OPC differentiation and may thereby impair remyelination. In this study, we utilized a tamoxifen inducible cre-lox strategy to conditionally knockout (cKO) SOCE in adult OPCs prior to lysolecithin-induced spinal cord demyelination. NG2creER;Stim1/2 floxed mice and cre-negative littermates were injected at 8-weeks with tamoxifen. Following demyelination, we analyzed OPC density and proliferation at 5 days post-lesion (dpl). Stim1 or Stim2 cKO had no significant effect on the density of Olig2+ cells at 5 dpl and did not alter the proportion of EdU + proliferating OPCs. Likewise, the density of Pdgfra + OPCs was not influenced at 7 dpl. However, at 7 dpl, both Stim1 and Stim1/2 cKO mice exhibited increased densities of CC1+ and Plp1+ oligodendrocytes (OLs) as well as increased proportion of CC1+ OLs among the Olig2-defined OL lineage pool (2-way ANOVA, *p* < 0.05). As the individual effects of Stim2 and the interaction between Stim1/2 were not significant, these results indicate that Stim1 alone plays a critical role. Next, to determine whether pharmacological inhibition of SOCE may improve OL differentiation following demyelination, we intraspinally injected a small molecule Orai1 inhibitor CM4620 (Zegocractin) at the time of demyelination in wild-type mice. At 7 dpl, both 10 μM and 50 μM CM4620 doses induced a significant increase in the proportion of mature CC1+ OLs (Tukey *p* = 0.0484, *p* = 0.0305). Thus, SOCE inhibitor treatment may act in a similar manner to Stim1 cKO to promote differentiation. Future studies will determine the effects of Stim1 cKO on remyelination, investigate the mechanisms of action of CM4620, and determine whether systemic treatment can influence remyelination in other models of demyelination. These results indicate that OPC-expressed Ca^2+^ sensing proteins can modulate OL differentiation following demyelination and may offer a novel therapeutic means to enhance myelin repair.

# Abstract: P01.110 – The Function of Sex-Specific Extracellular Vesicles in the Pathology of Alzheimer’s Disease

**Published:** 2025-09-05

**Authors:** Xiaojia Ren, Ahmed Elsherbini, Zhihui Zhu, Zainuddin Quadri, Simone Crivelli, Hemendra Vekaria, Priyanka Tripathi, Liping Zhang, Erhard Bieberich

**Affiliations:** a Department of Physiology, University of Kentucky College of Medicine, Lexington, KY, USA; b Spinal Cord and Brain Injury Research Center, University of Kentucky, Lexington, KY, USA; c Veterans Affairs Medical Center, Lexington, KY, USA

## Abstract

After decades of studies on Alzheimer’s disease (AD), the persisting lack of effective treatments remains a major concern. Sex is increasingly recognized as an important variable in AD pathology. Emerging evidence also suggests that extracellular vesicles (EVs) secreted by reactive astrocytes play crucial roles in AD pathology and the sex-specific function of EVs may contribute to the pathological sex differences of AD patients. The goal of the current study was to determine the sex specific differences in the composition and function of EVs in AD. We found that female wild type and 5XFAD mouse brain contained a higher proportion of EVs that are labeled for CD9. CD9 is a tetraspanin typically found in microvesicles, a type of EVs shed from the plasma membrane by activation of acid sphingomyelinase (ASM), an enzyme that generates the sphingolipid ceramide. Nanoview analysis of individual EVs showed that CD9-positive EVs from 5XFAD brain were labeled for ASM, ceramide, GFAP, and Aβ, suggesting that these EVs are derived from astrocytes and enriched with ceramide due to Aβ-induced activation of ASM. Seahorse assays showed that mitochondrial impairment was greater when neuronal cells were treated with EVs from female mouse brain, suggesting that a greater proportion of CD9-labeled EVs leads to increased severity of AD pathology in female brain. Further, we hypothesize that enrichment of these EVs with ceramide critically contributes to EV-mediated AD pathology by impairing mitochondrial function in neurons. To test our hypotheses and determine the function of CD9-positive ceramide-rich EVs (CREVs), we developed a novel EV reporter mouse model. The astrocyte specific Aldh1l1-Cre;CD9-turboGFP mouse secretes CD9-turboGFP labeled EVs that can be tracked in vitro and in vivo. Our data suggests that the sex-specificity of EVs plays an important role in AD pathology and the neurotoxicity of astrocyte-derived CREVs ultimately leads to innovative therapeutic approaches targeting the ceramide pathway for individuals of all genders.

# Abstract: P01.111 – Characterization of the Inflammasome in the CLN2 Mouse Model

**Published:** 2025-09-05

**Authors:** Kathryn Sanchez, Miriam Domowicz, Nancy Schwartz

**Affiliations:** a University Of Chicago Department of Pediatrics, Chicago, IL, USA

## Abstract

Neuronal Ceroid Lipofuscinoses, (NCLs or Batten disease) are a group of pediatric, inherited, early onset, fatal neurodegenerative diseases associated with mutations in 13 genes. All forms of the disease are characterized by lysosomal accumulation of fluorescent storage material, as well as profound neurodegeneration. The classical late-infantile NCL (cLINCL) in humans, caused by mutations in the CLN2/TPP1 gene, is usually diagnosed between 2 and 4 years of age with onset of seizures followed by the severe neurological deterioration typical of NCLs, and most patients die between 7 and 15 years old. An underappreciated component of pathology includes the inflammatory response observed in the central nervous system. When the tripeptidyl peptidase 1 (Tpp1) deficient murine model (Tpp1−/−) was used to replicate the human disorder, inflammatory genes associated with microglia and astrocytes were upregulated. Though it was determined that microglia contribute to the onset of inflammation observed in the Tpp1−/− mouse model through the upregulation of Ctss, Itgb2, Itgax, Lyz2, the mechanisms of their function remain to be elucidated. For example, in response to danger-associated molecular patterns, microglia often assemble a multiprotein complex called the inflammasome. The inflammasome is composed of the NACHT, LRR, and PYD domain-containing protein 3 (NLRP3); apoptosis-associated speck-like protein containing a CARD (ASC); and inflammatory caspase-1. After inflammasome assembly, caspase-1 promotes the maturation of the inflammatory cytokine IL-1β. This study aims to determine how genes related to inflammasome assembly impact the onset of inflammation in the Tpp1−/− mouse since it has not been described in the context of CLN2. Here, we report the mRNA expression of genes such as Caspase-1, ASC, NLRP3, and IL1β are altered in the cerebellum of 4-month-old Tpp1−/− mice by quantitative PCR suggesting that inflammasome assembly is relevant to this mouse model. Currently we are targeting the inflammasome with the small molecule inhibitor MCC950 in Tpp1−/− acute cortical slices to observe its impact on inflammation. These data will provide the basis for future investigations that therapeutically target the inflammasome.

# Abstract: P01.112 – A Translational Readthrough Variant of AQP4 Has Altered Gliovascular Properties and Modifies Neurological Diseases

**Published:** 2025-09-05

**Authors:** Hamza Lalami, Prarthana Suresh, Zhan Shi, Victor Nguyen, Sadan Dahal, Darshan Sapkota

**Affiliations:** a University of Texas at Dallas, Richardson, TX, USA

## Abstract

Aquaporin 4 (AQP4) is a water channel expressed in astrocytes, with roles in the maintenance of water balance, clearance of waste products, and pathogenesis of various brain disorders. Recently, we identified a new variant of AQP4, termed AQP4X, which contains extra amino acids at the C-terminus due to ribosomes continuing past the stop codon of the Aqp4 transcript. This variant localizes exclusively around the blood-brain barrier and mediates amyloid beta removal. Here we report additional gliovascular and neurological roles for AQP4X using mice that lack (Aqp4No_X, two extra stops added to prevent translational readthrough) or overexpress (Aqp4All_X, stop mutated to sense) this variant. Biochemical assays show that the Aqp4All_X mouse has reduced levels of supramolecular complexes that AQP4 is known to form in the astrocyte membrane. In line with this, the Aqp4All_X mouse displays attenuated edema pathology in comparison to wildtype mice. Remarkably, both Aqp4No_X and Aqp4All_X mice exhibit reduced reactivity to sera from patients with neuromyelitis optica, a demyelinating autoimmune disorder in which AQP4 is thought to be the self-antigen. Thus, AQP4X modifies the gliovascular properties of AQP4 and emerges as a potential therapeutic target for managing diseases like brain edema and neuromyelitis optica.

# Abstract: P01.113 – MiRNAs as Biomarkers for and Mediators of Alzheimer’s Disease

**Published:** 2025-09-05

**Authors:** Julie Saugstad, Ursula Sandau, Trevor McFarland, Jack Wiedrick, Zoe Fanning, Joseph Quinn

**Affiliations:** a Oregon Health and Science University, Portland, OR, USA

## Abstract

**Background:**

Alzheimer’s disease (AD) is the most common cause of dementia, and brain changes in AD begin 10–20 years before symptoms appear. We discovered and validated miRNAs in human cerebrospinal fluid (CSF) that differentiate AD from Controls, then identified a subset of CSF AD miRNAs that trend to decrease in expression from Control to Mild Cognitive Impairment (MCI) to AD. As blood is a preferred biofluid for noninvasive biomarker profiling, we i) evaluated the intraindividual longitudinal stability of human miRNAs in plasma, then ii) evaluated the CSF AD miRNAs in plasma. We also iii) examined if CSF AD miRNAs are in extracellular vesicles (EVs) and if two biological risk factors for AD, APOE4 genotype and sex, effect CSF EV miRNA expression.

**Methodology:**

All procedures were approved by the OHSU IRB00009707. i) Longitudinal Stability Study: blood was collected by venipuncture biweekly over 3 months from 22 donors who had fasted overnight. Total RNA was isolated from 200 μL of platelet-free plasma using the Qiagen miRNeasy Serum/Plasma Advanced Kit, and miRNAs analyzed on TaqMan Advanced miRNA Human A Cards. ii) CSF:Plasma Study: 320 donor- and date- matched CSF and plasma samples from AD and Controls were obtained from ADNI. Total RNA was isolated from 250 μL of CSF and plasma using a Urine miRNA Purification kit, and miRNAs analyzed on custom TaqMan Advanced Human miRNA Cards. iii) CSF EV Study: 5.0 mL of CSF was concentrated by ultrafiltration, fractionated by size exclusion chromatography, and miRNAs analyzed on TaqMan Advanced miRNA Human A Cards.

**Results:**

i) Longitudinal Stability Study: Of 134 miRNAs amplified in plasma, 74 were stable in an individual over 3 months, and 13 of 17 AD miRNAs were stable in plasma over 3 months. ii) CSF:Plasma Study: 24 of 25 validated CSF miRNAs differentiate AD from Controls in plasma. iii) CSF EV Study: most AD miRNAs are in EVs, and two biological risk factors for AD (APOE4 genotype and sex), alter the expression of CSF AD miRNAs in EVs.

**Conclusions:**

Our studies i) identified miRNAs with intraindividual longitudinal stability in human plasma over a 3-month time, including candidate AD biomarkers; ii) validated that AD CSF miRNAs differentiate AD from Controls in plasma, and iii) 4 of 5 CSF miRNAs that decrease in expression from Control to MCI to AD are in EVs. Thus, miRNAs can be biomarkers for AD, and miRNAs in EVs can serve as mediators of AD pathology.

# Abstract: P01.114 – Development and Validation of TRPML1 Assays on Automated Patch Clamp Platforms

**Published:** 2025-09-05

**Authors:** Daniel Sauter, Albert Steiner, Archana Jha, Rasmus Jacobsen

**Affiliations:** a Casma Therapeutics, Sophion Bioscience, Bedford, MA, USA; b Cambridge, MA, USA

## Abstract

TRPML1 is a cation-selective ion channel that is ubiquitously expressed in lysosomes and late endosomes. TRPML1 is intimately involved in the regulation of lysosomal Ca^2+^ homeostasis and is consequently a critical component of autophagy and lysosomal biogenesis. Malfunction of these processes are linked to various neurodegenerative disorders including Alzheimer`s and Parkinson`s disease. The relevance of TRPML1 in neurodegenerative disorders is further validated by a loss-of-function mutation that was shown to cause the neurodegenerative lysosomal storage disease mucolipidosis type IV (MLIV). The patch clamp technique remains the gold standard for studying ion channels as recordings provide a direct measure of the protein’s activity. Whilst powerful, the conventional patch clamp method requires highly trained scientists and allows only a low throughput. Automated patch clamp systems have evolved to overcome these limitations and these platforms are therefore well-suited to test large numbers of compounds in a short time to support ion channel drug development programs. Here, an automated patch clamp assay for TRPML1 recombinantly expressed in HEK293 cells is presented. To achieve plasma membrane expression, the two dileucine motifs (15LL and 577LL) of mouse TRPML1 responsible for lysosomal targeting were replaced with alanines to allow trafficking to the plasma membrane. Biophysical characterization of the channel in the whole-cell configuration was in good agreement with the literature. Application of the tool compound ML-SA5 at pH4.6 resulted in a marked increase in current amplitude with EC50 = 3.4 µM. In conclusion, the developed assay allows to record a large number of compounds in a short timespan to identify novel modulators of the TRPML1 channel.

# Abstract: P01.115 – Increasing Adult Oligodendrogenesis Through the Deletion of Glial-Expressed Voltage-Gated Calcium Channel Subunits

**Published:** 2025-09-05

**Authors:** Alaina Scavuzzo, Wenjing Sun, Morgan Saul, Hayes Johnson, Marissa Hooks

**Affiliations:** a Department of Neuroscience, Wexner Medical Center, The Ohio State Univeristy, Columbus, OH, USA; b Department of Biomedical Engineering, College of Engineering, The Ohio State University, Columbus, OH, USA

## Abstract

Myelination, the process by which glial cells produce layers of myelin sheaths that wrap around neuronal axons and act as a layer of insulation for the transmission of action potentials, is essential for central nervous system (CNS) function. In the CNS, myelin sheaths are exclusively formed by oligodendrocytes, which are differentiated from oligodendrocyte precursor cells (OPCs). OPCs express abundant voltage-gated calcium channels (VGCCs), and these VGCCs are known to play a role in regulating myelin development. Alpha2delta1 (A2d1) subunits, the auxiliary subunits of VGCCs, increase the membrane expression of VGCCs and subsequent Ca^2+^ current density. The goal of this study is to investigate how A2d1 subunits expressed in OPCs regulate the proliferation and differentiation of OPCs and the subsequent myelination. To knock out Cacna2d1, the gene encoding A2d1, in OPCs specifically, we crossed a tamoxifen-inducible PDGFRa-CreERT2 mouse line with Cacna2d1fl/fl mice to generate a double transgenic PDGFRα: Cacna2d1fl/fl line. In addition, we crossed the PDGFRα-CreERT2 with the TdTomato reporter mice (Ai9) to generate the control double transgenic PDGFRα:TdTomato line. Tamoxifen was injected into 8-week-old mice for 4 consecutive days. We validated in the PDGFRα:TdTomato line that the majority of OPCs turned on Cre activity and became TdTomato+, 2 weeks after tamoxifen injection. Interestingly, we did not observe any alteration in the OPC proliferation rate using the BrdU incorporation technique. However, we saw an increase in mature oligodendrocyte density when knocking out Cacna2d1 specifically in OPCs. Finally, when analyzing the myelin thickness, we found that 4 weeks after tamoxifen injection, Cacna2d1 KO mice also exhibited thicker myelin sheaths. These results suggest that deleting A2d1 subunits in adult OPCs enhances oligodendrogenesis.

# Abstract: P01.116 – System-Based Integrated Metabolomics and microRNA Analysis Identifies Potential Molecular Alterations in Human X-Linked Cerebral Adrenoleukodystrophy Brain

**Published:** 2025-09-05

**Authors:** Navtej Kaur, Jaspreet Singh

**Affiliations:** a Henry Ford Health System, Detroit, MI, USA

## Abstract

**Introduction:**

X-linked adrenoleukodystrophy (X-ALD) is a severe demyelinating neurodegenerative disease mainly affecting males. X-ALD is the most common peroxisomal disorder and caused by mutations in ABCD1 gene that encodes peroxisomal integral membrane protein ABCD1 which is involved in the transmembrane import of very long chain fatty acids (VLCFA; C ≥ 22:0). The etiology of X-ALD is unclear and same mutation can result is a spectrum of phenotypes. Sixty percent of X-ALD males spontaneously progress to the severe cerebral adrenoleukodystrophy (cALD) phenotype which is fatal in 2–5 years of symptom onset. The underlying mechanism of onset and progression of neuropathology remains poorly understood.

**Objectives:**

In this study our objective was to integrate metabolomic and microRNA (miRNA) datasets to identify molecular and metabolic pathways associated with cALD phenotype severity. Postmortem brain tissue samples from five healthy controls (CTL) and five cALD patients were utilized in this study.

**Methods:**

White matter from cALD patients’ postmortem brain was obtained from normal-appearing areas away from lesions (NLA) and from the periphery of lesions- plaque shadow (PLS). White matter from age and sex-matched postmortem brain of patients who deceased due to non-neurological reasons served as control (CTL). Metabolomics was performed by gas chromatography coupled with time-of-flight mass spectrometry and miRNA expression analysis was performed by next generation sequencing (RNAseq). Ingenuity pathway analysis (IPA) was used for integrated miRNA-metabolomic network analysis.

**Results:**

Principal component analysis revealed that among the three sample groups (CTL, NLA and PLS) there were nineteen differentially expressed miRNA, including several novel miRNA, of which seventeen were increased with disease severity and two were decreased. Untargeted metabolomics revealed thirteen metabolites with disease severity-related patterns with seven increased and six decreased with disease severity. Ingenuity pathway analysis of differentially altered metabolites and miRNA comparing CTL with NLA and NLA with PLS, identified several hubs of metabolite and signaling molecules and their upstream regulation by miRNA. Glucose metabolism, phospholipid metabolism and NAA metabolism along with miR-155-5p, miR-148a-3p, miR-23a-3p and miR-21-5p were modified across all the comparisons indicating thay were modified in concert with increasing pathology of disease in cALD. Glutamine metabolism was altered between NLA and PLS regions of the cALD brain indicating metabolic reprogramming of greater scale in cALD. Novel miR-2114-3p along wih miR-148-3p and miR-378-3p were predicted to target NDP gene that encodes for norrin protein. IPA analysis predicted a linkage of NDP to L-aspartic acid metabolism. This provides a novel line of investigation for miRNA regulation of metabolic derangements in cALD pathology.

**Conclusion:**

In summary, this study combines metabolomic and miRNA expression profiling/bioinformatic analysis to provide broader information on miRNA and metabolites likely to play a role in cALD pathology beyond the genetic defect (ABCD1 mutation) and biochemical defect (VLCFA accumulation) common to all the X-ALD patient phenotypes. The transomic approach to map the crosstalk between miRNA and metabolomics suggests involvement of specific molecular and metabolic pathways in cALD and offers opportunity to understand the complex underlying mechanism of disease severity in cALD

# Abstract: P01.117 – Microglia-Mediated Synaptic Dysfunction Contributes to Chemotherapy-Related Cognitive Impairment

**Published:** 2025-09-05

**Authors:** Jingxiong Wang, Hua Zhang, Marc Augenreich, Peiru Zheng, Luis Martinez-Lemus, Heike Wulff, De-Pei Li

**Affiliations:** a University of Missouri-Columbia, Columbia, MO, USA

## Abstract

Chemotherapy-related cognitive impairment (CRCI) is a severe unmet clinical disorder that significantly impacts cancer survivors. However, the underlying cellular and molecular mechanisms remain unknown. Microglia play crucial roles in healthy and diseased brain. We hypothesize that microglia-mediated synaptic plasticity drives CRCI. We treated adult male C57BL/6J mice with a clinically relevant doses of chemotherapeutic drugs 5-Fluorouracil and leucovorin (5-Fu/LV) for 3 weeks. One month after completion of 5-Fu/LV treatment, the cognitive function was assessed by novel object recognition (NOR) test. Compared with the vehicle-treated mice, 5-Fu/LV-treatment reduced the preference of exploring novel object (68.4 ± 6.8 vs. 48.6 ± 7.6%, *n* = 12, *p* < 0.05) in NOR test. 5-Fu/LV treatment increases the numbers of microglial (from 11500 ± 2400 to 21200 ± 5000 cells/mm3, *p* = 0.0002) and CD68+ microglia (from 8447 ± 1276 to 13220 ± 1529 cells/mm3 (*p* < 0.0001) with shortened process length (150 ± 30 vs. 118 ± 34 μm/cell, *p* = 0.038) and diminished endpoints (160 ± 46 vs. 57 ± 38 per cell, *p* < 0.001), while decreases the number of phagocytotic (≤ 1 FITC-beads) Iba1+ microglia (46 ± 5.1 vs. 22 ± 6.0%). Furthermore, 5-Fu/LV treatment reduces the long-term potentiation (LTP) recorded from hippocampal CA1 region in response to a theta burst stimulation of CA3-CA1 pathway, while decreases the evoked N-Methyl-D-aspartic acid receptor (NMDAR)-excitatory postsynaptic currents (NMDAR-EPSCs) recorded in CA1 neurons. Co-administration of a microglia inhibitor minocycline reverses cognitive decline and microglia changes, restores the reduction of LTP and NMDAR-EPSCs in mice treated by 5-Fu/LV. Take together, our data suggest that microglial malfunction and related synaptic dysfunction contribute to 5-Fu/LV-induced cognitive impairment in mice. Targeting microglia during chemotherapy may mitigate cognitive impairment clinically.

# Abstract: P01.118 – Interactions Between TDP-43 and Amyloid Beta Pathology in a Drosophila Model of Alzheimer’s Disease

**Published:** 2025-09-05

**Authors:** Thaddeus Weigel, Dina Alter, Jaeda Coutinho-Budd

**Affiliations:** a Department of Neuroscience, University of Virginia, Charlottesville, VA, USA

## Abstract

While neurodegenerative diseases can arise from disparate etiologies, in recent years many neurodegenerative diseases have been found to share TDP-43 proteinopathy. TDP-43 is a protein that can form toxic intracellular aggregates in multiple cell types of the brain, including both neurons and glial cells. TDP-43 is most commonly associated with amyotrophic lateral sclerosis (ALS), with mutations to the gene encoding TDP-43 found in some ALS cases; however, TDP-43 pathology is also observed in other neurodegenerative disorders such as frontotemporal dementia, Parkinson’s disease, and Alzheimer’s disease (AD). AD is characterized by the aggregation of the proteins amyloid beta and tau, but TDP-43 pathology has been reported in up to 75% of AD cases. TDP-43 pathology is associated with more severe neurodegeneration and dementia than AD with amyloid or tau pathology alone, highlighting TDP-43 as an important topic of further research in AD. Rodent models of AD have shown that amyloid beta can drive pathological changes to TDP-43 expression, modification, and localization; however, many aspects of the biology underlying these synergistic effects is not well understood. We propose to utilize Drosophila, a powerful genetic model system, to better understand TDP-43 in the context of AD pathology. The plentiful tools available in Drosophila to manipulate genes with exquisite spatiotemporal precision easily enables the expression of human amyloid beta, wild type and mutant TDP-43, and the simultaneous selective up- and downregulation of endogenous genes related to neurodegeneration in both neurons and glial cells. With these tools, our work aims to investigate questions such as the cell type-specific effects of TDP-43 pathology in neurons versus glia in an AD context, the effects of amyloid beta on the spreading of TDP-43 pathology, and the mechanisms driving the development of TDP-43 pathology in AD.

# Abstract: P02.01 – Thyroid Hormone Decreases Cx43-Containing Extracellular Vesicle Production in Cultured Astrocytes

**Published:** 2025-09-05

**Authors:** Amanda Charest, Lars Udo-Bellner, Kaie Ojamaa, Randy Stout

**Affiliations:** a New York Institute of Technology College of Osteopathic Medicine, Old Westbury, NY, USA

## Abstract

Thyroid hormone (triiodothyronine, T3) has been shown by other groups to enhance gap junction connectivity and increase formation of gap junction macromolecular arrays composed of Cx43 in cell types outside the brain. Although the molecular mechanisms underlying T3 enhancement of gap junction connectivity are not known, we speculate that T3 acts to decrease Cx43 endocytosis and subsequent transfer into autophagy and related cellular pathways- thereby decreasing degradation of Cx43 in gap junction plaques. Based on our published and unpublished research, we propose that extracellular vesicles released by astrocytes that contain Cx43 are produced by a process called gap junction transendocytosis. Based upon the above ideas, we aimed to test whether T3 application to astrocytes would reduce the number of extracellular vesicles those astrocytes produce and release. We used primary mouse astrocytes cultured in media supplemented with charcoal stripped serum to remove all T3. We used multichannel Stochastic Optical Reconstruction Microscopy (STORM) to image extracellular vesicles in astrocyte conditioned media and analyzed image data with cluster analysis (Nanometrix EV analysis software) to characterize the number, size, and protein content of extracellular vesicles released by astrocytes. We compared extracellular vesicle characteristics from astrocytes treated with physiologically relevant concentrations of T3 and extracellular vesicles produced by astrocytes in media devoid of T3. In preliminary results, we found a 30% reduction in Cx43 containing vesicles in the supernatant media of astrocytes treated with 100 nM T3 for 48 h in comparison to astrocytes cultured in media without T3. There was also a reduction in the number of extracellular vesicles that expressed both GLAST and Cx43 in vesicles produced by astrocytes treated with T3, compared to controls. We are also examining cultured astrocytes with super-resolution imaging of the intact cells to better understand the effects of T3 on the Cx43 gap junction protein “life cycle” using immunofluorescent co-staining of Cx43, the late-endosome and extracellular vesicle marker CD63, and other endo/lysosomal proteins. This work lends better understanding of T3 influence on gap junctions that connect glia. Decreased gap junction connectivity in astrocytes has been implicated in major depression and other mood disorders. Despite conflicting evidence, hypothyroidism has been associated with cognitive dysfunction and risk of mood disorders. Therefore, better understanding of how astrocyte gap junctions and extracellular vesicle production change in hypothyroidism may aid in development of new biomarkers and therapeutics for brain disorders associated with altered astrocyte Cx43 expression and localization.

# Abstract: P02.02 – Oxygen Availability Regulates PG Production Independently of Cyclooxygenase Induction Upon Stimulation With LPS

**Published:** 2025-09-05

**Authors:** Brennon Schofield, Drew Seeger, Derek Besch, Peddanna Kotha, Svetlana Golovko, Meredith Parmer, Mikhail Golovko

**Affiliations:** a Department of Biomedical Sciences, School of Medicine, University of North Dakota, Grand Forks, ND, USA

## Abstract

Prostanoids (PG) are key regulators of many processes in the brain including inflammation, angiogenesis, and neuroplasticity and are implicated in a variety of pathologies including neurodegeneration, central nervous system injury, and cancer. It is well established that LPS dramatically stimulates PG synthesis as part of the inflammatory response. Traditionally, this PG synthesis is thought to be attributed to the activation of the arachidonic acid cascade which is initiated by toll-like receptor 4 (TLR4) stimulation causing the activation of various calcium and kinase-dependent lipases to release arachidonic acid. As a result, cyclooxygenase 1 and 2 (COX), the rate-limiting enzymes for PG synthesis, along with downstream synthases, convert the released arachidonic acid into PG, leading to a significant increase in PG levels during ischemia. Additionally, increase in COX expression from TLR4 signaling contributes to LPS-stimulated PG synthesis. However, we have recently demonstrated that PG induction may also be regulated by tissue O_2_ levels. In this study, we discovered that PG formation in the ischemic brain does not occur without exposure to exogenous O_2_, but the regulatory role of this mechanism under other stimulatory conditions, including inflammatory response, is unknown. In the present study, we propose that O_2_ availability is an additional regulatory mechanism for COX-dependent PG production in biological systems. This hypothesis is based on the observation that Km for COX by O_2_ is between 10 and 100 μM. Because this is close to the tissue free O_2_ concentration of ∼50μM, we hypothesized that O_2_ concentration may regulate PG production upon stimulation including inflammatory conditions. To address this hypothesis, immortalized microglial cells treated with vehicle control or LPS were cultured at different O_2_ concentrations modeling hypoxic conditions, normoxic tissue, and the physiologically hyperoxic conditions of normal cell culture. PG were measured using a targeted UPLC-MS/MS approach. COX induction was determined by Western blot. O_2_ concentrations in media were measured by O_2_-sensitive electrode. The Km for PG production by O_2_ was calculated based on PG levels at different free O_2_ concentrations in the cell culture media. COX showed dramatic induction upon LPS treatment, that was independent from O_2_ concentrations. Despite the COX induction upon LPS treatment, no increased PG production was detected with LPS treatment at 1% O_2_ for several PG, including PGE2, which showed a 2-fold increase under atmospheric 20% O_2_. Less than 2-fold increase was detected for PGD2 and PGF2α under 1% O_2_ while they were increased 8-fold and 4-fold, respectively, under atmospheric 20% O_2_. At 5% and 10% O_2_, PG production was higher compared to 1% O_2_ but still lower compared to 20% O_2_. The calculated Km for PG synthesis by dissolved O_2_ is 40 µM which corresponds to 5% in the atmosphere. In summary, the results demonstrate that PG in LPS-treated cells increase with O_2_ concentration and are independent of COX induction at low O_2_ levels. These findings provide, for the first time, evidence that O_2_ availability can be a regulatory factor for PG production under inflammatory stimulus independent of COX induction.

# Abstract: P02.03 – Adhesion GPCR-Dependent Communication Across Cells

**Published:** 2025-09-05

**Authors:** Nicole Scholz, Anne-Kristin Dahse, Marguerite Kemkemer, Anne Bormann, Max Buhlan, Amelie Meyer-Mölck, Yin Kwan Chung, Franziska Klose, Mohamed Ali Jarboui, Tobias Langenhan

**Affiliations:** a Rudolf Schönheimer Institute of Biochemistry, Division of General Biochemistry, Medical Faculty, Leipzig University, Leipzig, Germany; b Core Facility for Medical Bioanalytics, Institute for Ophthalmic Research, Eberhard Karls University of Tübingen, Germany

## Abstract

Adhesion GPCR (aGPCR) are present in every organ system of the human body, constituting a large and rather peculiar group of cell surface receptors sensitive to mechanical stimuli. They exhibit a unique combination of characteristics otherwise known from integrins, protease-activated receptors and polycystines. Similar to polycystin-1 proteins, aGPCR undergo auto-proteolysis at the evolutionarily conserved GPCR autoproteolysis-inducing (GAIN) domain. The resulting N- and C-terminal fragments (NTF, CTF) remain non-covalently linked to form heterodimeric receptor molecules. One of the prevalent concepts of aGPCR activation involves the separation of NTF-CTF heterodimers, subsequently exposing the tethered agonist (Stachel). However, whether aGPCR dissociation is relevant under physiological conditions has been a subject of controversy. Here, I present a novel transgenic sensor system designed to monitor and quantify aGPCR dissociation in vivo. Finally, I will share data on the function of latrophilin-type aGPCR Cirl/ADGRL in neural processes during the development of the Drosophila brain.

# Abstract: P02.04 – An Alternative Fixation Method for Preventing Post-Mortem Prostanoid Increase in the Brain

**Published:** 2025-09-05

**Authors:** Drew Seeger, Derek Besch, Brennon Schofield, Svetlana Golovko, Peddanna Kotha, Meredith Parmer, Mikhail Golovko

**Affiliations:** a University of North Dakota, Grand Forks, ND, USA

## Abstract

Previously, we and others have reported a rapid and dramatic increase in brain prostanoids (PG) under ischemia that is traditionally explained through the activation of esterified arachidonic acid (20:4n-6) release by phospholipases as a substrate for cyclooxygenases (COX). Thus, it was assumed that rapid enzyme deactivation is essential for PG analysis. Several methods have been utilized to achieve this rapid tissue fixation, including freeze-blowing that rapidly cools brain attenuating metabolic activity but doesn’t preserve tissue structure and metabolic activity can proceed if thawed. Another technique, that is considered the gold standard, is tissue fixation in situ by head-focused microwave irradiation (MW). However, this technique can suffer from poor inter-sample reproducibility due to tissue loss, metabolite degradation if overheated, and MW devices are not readily available in most labs. Thus, an alternative method that is reproducible, accessible, and retains brain anatomy is needed. Recently, we reported, for the first time, that exogenous oxygen availability is essential for postmortem PG induction. Interestingly, these data suggest that the dramatic postmortem induction of brain PG does not occur before craniotomy. Thus, we proposed that rapid enzyme de-activation is not required for true endogenous PG level quantification if enzymes are de-activated prior to exposure to oxygen. Based on this assumption, we proposed a simple and reproducible alternative method by heat deactivating enzymes in situ by boiling the intact cranium to prevent postmortem PG induction. To assess this approach, intact mouse craniums were dropped into boiling saline after decapitation. Brain temperature reached 100 °C after ∼100 sec during boiling, with samples being collected at 90 sec (95.9 °C) and 180 sec (99.4 °C). Interestingly, 180 sec of boiling was required to completely prevent post-mortem PG synthesis activity tested by tissue incubation for 20 min at 37 °C before PG extraction. Importantly, boiling fixation method attenuates 20:4n-6 release by 77%, but does not arrest release as compared to MW (3.9-fold increase). To validate tissue boiling fixation method, brain basal and LPS-induced PG levels were determined in non-fixed, MW-fixed, and boiling-fixed brains. PG levels in non-fixed brains were significantly increased compared to basal fixed tissue levels, with increased PGE₂ (60-fold), prostaglandin D₂ (235-fold), 6-keto prostaglandin F₁α (60-fold), thromboxane (13-fold), and prostaglandin F₂α (420-fold). The basal and LPS-induced PG levels were not different between fixation method. Importantly, both fixation methods allowed the detection of a higher PG induction upon LPS treatment compared to basal fixed tissue. These data indicate, for the first time, that boiling effectively prevents induced post-mortem PG alterations, allowing for a reproducible, low-cost, and conventionally accessible tissue fixation method for PG analysis. Further studies are required to investigate the effectiveness of this method in preventing post-mortem alterations of other metabolites.

# Abstract: P02.05 – The Role of Mechanosensation in Central Nervous System Myelination

**Published:** 2025-09-05

**Authors:** Amanda Senatore, Julia Halford, Adam Coombs, Ben Emery, Kelly Monk, Swetha Murthy

**Affiliations:** a Oregon Health and Science University, Portland, OR, USA

## Abstract

Oligodendrocytes, the myelinating cells of the central nervous system, sense, integrate and respond to a variety of cues to precisely select neuronal targets for myelination. Among signals such as the presence of axonal membrane proteins and neuronal activity, mechanical cues have emerged as an important factor in directing myelination. For example, oligodendrocytes preferentially myelinate axons of a certain caliber and will even wrap inert nanofibers in culture. Despite the importance of this mechanical input, how oligodendrocytes sense and respond to mechanical cues in their environment is poorly understood. Recently, several case studies have uncovered human mutations in the mechanically activated ion channel, TMEM63A, which lead to transient hypomyelinating leukodystrophy. Interestingly, this channel is highly expressed in oligodendrocytes and preliminary evidence indicates that disruption of Tmem63a in both mouse and zebrafish models recapitulates the transient hypomyelination documented in human patients. By using a combination of mouse in vitro assays and in vivo imaging in zebrafish, this study will elucidate how mechanical cues sensed by TMEM63A contribute to myelination in the central nervous system.

# Abstract: P02.06 – Facilitating Oligodendrocyte Maturation and Safeguarding Myelin Integrity in Multiple Sclerosis via the TET1-BHMT Axis

**Published:** 2025-09-05

**Authors:** Marwan Shalih Maraicar, Katherine Knies, Morgan W. Psenicka, Robert Clements, Jessica Williams, Jennifer McDonough

**Affiliations:** a Department of Biological Sciences, Kent State University, Kent, OH, USA; b Lerner Research Institute, Cleveland Clinic, Cleveland, OH, USA

## Abstract

Multiple sclerosis (MS), a debilitating demyelinating disease characterized by inflammation, neurodegeneration, and impaired oligodendrocyte progenitor cell (OPC) differentiation, presents an ongoing therapeutic challenge. Dysregulation of methionine metabolism has been implicated in MS, leading to decreased levels of key methyl donors such as S-adenosylmethionine (SAM). Our study sought to elucidate a groundbreaking therapeutic strategy targeting methionine metabolism with betaine, focusing on the betaine-homocysteine S-methyltransferase (BHMT) pathway, a key player in restoring SAM levels and facilitating DNA and histone methylation crucial for OPC survival and differentiation. Employing the experimental autoimmune encephalomyelitis (EAE) mouse model as a representative paradigm for MS, we administered betaine intraperitoneally at peak disability and employed coherent anti-Stokes Raman scattering (CARS) microscopy – a state-of-the-art technique – to assess myelin sheath composition around lumbar spinal cord axons without staining. Our results demonstrated a significant improvement in motor ability during the chronic stages of EAE, validated by substantial lipid content enhancement in the betaine-treated mice via CARS microscopy and clinical scoring. Chromatin immunoprecipitation sequencing (ChIP-seq) analysis unveiled significant BHMT enrichment at over 2,000 genes, including critical regulators of differentiation and maturation (Sox10 and Olig2) and genes pivotal for metabolism (e.g., Pdk1). Although BHMT mediated DNMTs activation and DNA methylation are associated with transcriptional repression, Sox10 and Pdk1 genes are activated under betaine administration. Proximity ligation assay (PLA) confirmed BHMT interactions with Ten-Eleven Translocation 1 (TET1), a methyl cytosine dioxygenase in the nucleus of OPCs and oligodendrocytes in both murine and human sclerotic lesions, hinting at a broader influence on transcriptional activation in OPCs through hydroxy-methylation by TET1 and BHMT. Moreover, it appears from our recent PLA assays that betaine administration also significantly increases BHMT and TET1 mediated hydroxy-methylation marks specifically in adult oligodendrocytes surrounding the sclerotic lesions in murine spinal cords, thereby highlighting BHMT’s potential as an epigenetic intervention target. Single cell ChIP-seq analysis of genes targeted by DNA hydroxy-methylation (5-hmc) is underway which will elucidate deeper insights into oligodendrocyte maturation and mediated remyelination after betaine administration. Our study underscores the therapeutic potential of betaine-mediated epigenetic modification via the BHMT pathway, showcasing its impact on motor disability, myelin composition, and the intricate regulation of transcription in OPCs. The nuanced interplay of BHMT with DNMT and TET1 emphasizes the versatility of this pathway in influencing both DNA methylation and hydroxy-methylation, elucidating a comprehensive approach to myelin repair in MS. This innovative strategy opens new avenues for targeted interventions in MS and potentially other neurodegenerative diseases, warranting further exploration of the specific mechanisms underlying hydroxy-methylation and its orchestrated gene activation for precise therapeutic development.

# Abstract: P02.07 – A Molecular Dissection of Complement in Demyelinating Disease

**Published:** 2025-09-05

**Authors:** Patrick Sheehan, Violeta Durán-Laforet, Robert Murphy, Dorothy Schafer

**Affiliations:** a University of Massachusetts Medical School, Worcester, MA, USA

## Abstract

While there are many FDA-approved therapies to treat relapsing-remitting multiple Sclerosis (MS), there are far less options for treating neurodegeneration in the progressive phase of the disease. A hallmark of progressive disease in MS, as well as most other neurodegenerative diseases, is the loss of synapses and gray matter atrophy. Our lab recently showed that preventing synapse loss by blocking complement C3 deposition on synapses in the visual thalamus of an MS-relevant mouse model (experimental autoimmune encephalomyelitis, EAE), rescued vision loss. This was particularly intriguing as visual impairment in MS patients has historically been attributed to demyelination of the optic nerve. While complement proteins C1q and C3 are both known to act as synaptic “eat me” signals, only C3 binds to synapses in the visual thalamus to induce synaptic phagocytosis. To add to this complexity, recent evidence suggests that complement C1q may influence the activation state of microglia and that microglia expressing high amounts of C1q are found around chronic active lesions in MS patients. Therefore, it is still unclear if C1q is acting upstream of C3 to cause synapse phagocytosis, or how C1q is modulating microglia activity. We hypothesize that microglia-derived C1q is necessary for microglia to transition to the full disease-associated microglia (DAM) signature and that this transition is critical for astrocytic C3 production upstream of microglia-mediated synapse elimination. To address these questions, we first mapped the spatial and transcriptional changes in complement during demyelinating disease. To do this, we are using MERFISH (multiplexed error robust fluorescence in situ hybridization), which utilizes custom-designed panels of probes targeting RNAs of interest in order to map expression of each RNA at single molecule and single cell resolution while retaining spatial information. With this technique, we not only map the expression of C1q and C3 in EAE and control tissue, but we can also identify surrounding cells with C1q and C3 receptors and mediators. We are also currently performing experiments to knockout C1q specifically in microglia during EAE. With these experiments, we can analyze the amount of C3 on synapses to understand if loss of C1q impacts C3 deposition on synapses. Ultimately, I aim to gain a more comprehensive understanding of how complement proteins are regulated during demyelinating disease.

# Abstract: P02.08 – Fever-Like Temperatures Without Immune Activation Improve Cognitive Deficits in the Scn2a Autism Mouse Model via Increases in K + Channel Activity

**Published:** 2025-09-05

**Authors:** Yiming Shen, Jordan Meza, Joey Abramovitz, Michelle Antoine

**Affiliations:** a NIH/NIAAA, Rockville, MD, USA

## Abstract

Autism spectrum disorder (ASD) prevalence is increasing – 1 in 54 children is diagnosed, and treatment options for its associated cognitive deficits are limited. Interestingly, 17–83% of ASD patients experience improved cognition during fever, a phenomenon called the ‘Fever Effect’. Fever is considered an increase in body temperature (BT) beyond 38 degree and is usually induced by infection. It remains unclear whether cognitive improvements in ASD patients are facilitated by the thermal or immune component of fever. To test this, we newly developed a ‘thermal fever’ (TF) protocol to elevate mouse BT into fever range via exposure to infrared light. The TF protocol is combined with either the presence or absence of an immune challenge via a single intraperitoneal dose of the bacterial lipopolysaccharide (LPS) antigen. LPS alone did not elicit fever. In this study, we utilized mice haploinsufficient for SCN2A haploinsufficiency (Scn2a+/-), a well-validated model for ASD and intellectual disability. To assess cognition, Scn2a+/-mice and littermate controls performed a reversal learning test during exposure to the TF protocol, with and without immune challenge. Scn2a+/- mice made fewer errors during the task under TF, thus the thermal component of fever is sufficient to replicate the fever effect. To uncover the mechanism mediating improved behavior, we recorded evoked activity in cortical L2/3 pyramidal (PYR) neurons at increasing temperatures (30, 36, and 39 degree). Results revealed an enhanced spike probability at 30 and 36 degree, yet this normalized at 39 degree. We found that this spike probability normalization was associated with temperature-induced increases in potassium channel function in Scn2a+/- excitatory pyramidal neurons that elevated spike threshold. We next applied pimaric acid (a potassium channel opener), which decreased PYR neuron spiking in Scn2a+/- mice to wildtype levels at 36 degree. To confirm that this reduction in spiking improves cognitive function, we used an inhibitory DREADD to decrease cortical PYR neuron spiking during reversal learning. This intervention successfully resulted in normal reversal learning in Scn2a+/- mice. In sum, our results show the thermal component of fever is sufficient to improve cognitive impairments in Scn2a+/- mice, specifically by reducing abnormal spiking activity in cortical networks. These findings may lead to therapeutic strategies which reproduce the beneficial effects of fever in ASD.

# Abstract: P02.09 – Measuring ATP:ADP in Neurons and Astrocytes Using PercevalHR In Vitro

**Published:** 2025-09-05

**Authors:** Sophiya Sims, Sami Case, Ruei-Lung Lin, Nicholas Wright, Leopoldine Galopin, Olivier Thibault

**Affiliations:** a Department of Pharmacology and Nutritional Sciences, University of Kentucky College of Medicine, Lexington, KY, USA; b Department of Biomedical Sciences, College of Veterinary Medicine and Biomedical Sciences, Colorado State University, Fort Collins, CO, USA

## Abstract

Brain homeostatic equilibrium is a well-maintained and orchestrated metabolic process which, when lost, is associated with brain aging or neurodegenerative diseases, and is often detected as hypometabolism in aging and AD. It is, therefore, integral to maintain this homeostasis. In addition to the well-established role of glucose metabolism in the CNS, more recently, insulin has been recognized to be essential in regulating cognitive function, particularly in the hippocampus, where it can ameliorate spatial memory recall. Using mixed primary hippocampal cultures (neurons and astrocytes), we tested the hypothesis that PercevalHR, an ATP:ADP biosensor, could reliably quantify bioenergetics with single cell resolution. Embryonic rat hippocampi (E18) were extracted and maintained for 12–16 days in vitro (DIV). Cultures were treated with lentivirus (Human Ubiquitin C promoter) containing the PercevalHR nanosensor. To control for PercevalHR’s pH sensitivity, some experiments were conducted concomitantly with the intracellular pH sensor pHrodo. We attempted to normalize glucose transporter function following ∼12 days in high glucose concentration (30 mM), by returning the cells to a serum-free 5.5 mM glucose media ∼24 h prior to imaging. PercevalHR emission was filtered at 525 nm and pHrodo emission at 580 nm. After an initial baseline, cells were treated with one of several compounds (0.5 mM, 5.5 mM, and 10 mM glucose; 50 mM KCl; 20 µM glutamate; 10 nM insulin). Glutamate and KCl resulted in rapid decreases in ATP:ADP. Conversely, insulin does not demonstrate any changes in ATP:ADP. To validate insulin response and health of the cultures, ECAR and OCR was measured, and do corroborate results found during imaging. PercevalHR seems to reliably report on cell energetics in mammalian cultures and surprisingly, appears to indicate that neurons display higher baseline ATP:ADP compared to astrocytes. These data evaluate bioenergetic status in two closely associated cell types that are known to share metabolic intermediates. Ongoing studies are investigating PercevalHR imaging in astrocytes using in vivo 2 P microscopy in a mouse model of amyloidosis during ambulation (i.e., awake).

# Abstract: P02.10 – Recovery of Astrocyte Calcium Signaling and Cerebrovascular Dynamic From General Anesthesia

**Published:** 2025-09-05

**Authors:** Moltira Promkan, Rungreudee Kimseng, Kristin Drummond, Blaine Weiss, Christopher Gant, Christopher Norris, Pradoldej Sompol

**Affiliations:** a University of Kentucky, Lexington, KY, USA

## Abstract

General anesthetic agents are widely used to induce sleep-like unconscious condition during clinical practice with painful procedures. Anesthetic drugs directly reduce neuronal activity and synaptic function and how the brain recovers from anesthesia effect is not completely known. Moreover, the mechanism of astrocyte and its related cerebrovascular function during anesthesia recovery are under studied. Here we investigated the effect of isoflurane on astrocyte calcium signaling in time-dependent series of different consciousness stages. Adeno associated virus was injected into the barrel cortex of 3–4 months old male and female mice to express calcium biosensor, GCaMP, under astrocyte specific promoter, Gfa. Then, cranial window surgery was prepared for further intravital imaging. The animals were anesthetized with isoflurane and mounted under multiphoton microscopy. We performed simultaneous air-puff stimulation of contralateral whiskers and recording of astrocyte calcium signaling at pre- and during stimulation at each consciousness stage starting from fully unconscious condition where isoflurane levels were maintained at 1.5%. Then, the same measurements were done when levels of isoflurane were at 0.3% for partial unconscious and 0% for fully awake condition. Our pilot data showed that astrocyte calcium signaling was diminished during whisker stimulation under unconscious condition and significantly increased under partial and fully conscious conditions. However, analysis of astrocyte calcium transient dynamic is ongoing. For cerebrovascular analysis, we found increased basal penetrating arteriole diameter, 1.55 (+ 0.2) and 1.47 (+ 0.17) folds at both at 1.5% and 0.3% condition respectively, compared to fully awake condition. During whisker stimulation, the magnitude of vascular diameter was not changed in the unconscious condition. Interestingly, whisker stimulation induced vasoconstriction in partial unconscious stage where vascular diameter was reduced 11.2 (+ 2.9) percent compared to its baseline. On the other hand, the magnitude of vasodilation was increased 23.4 (+ 8.9) percent in fully awake condition during whisker stimulation-induced neurovascular response. Laser speckle contrast imaging technique was used to investigate superficial cortical blood flow in this corresponding barrel cortex. Similar to astrocyte calcium signaling, superficial blood flow change was diminished during whisker stimulation under unconscious condition and significantly increased under partial and fully conscious conditions. Our results indicate returning of responsiveness of astrocyte calcium signaling, vascular dynamic and physiology of hyperemic response that may involve in brain function and cognitive reconstitution during anesthesia recovery.

# Abstract: P02.11 – Multi-Omics Analyses of Synapses From C9ORF72 ALS/FTD Cortical Neurons

**Published:** 2025-09-05

**Authors:** Ashton Spillman, Lynette Bustos, Nathaniel Hansen, Krystine Garcia-Mansfield, Lauren Gittings, Eric Alsop, Kendall Van Keuren-Jensen, Patrick Pirrotte, Rita Sattler

**Affiliations:** a Barrow Neurological Institute, Phoenix, AZ, USA; b Arizona State University, Tempe, AZ, USA; c Translational Genomics Research Institute, Phoenix, AZ, USA

## Abstract

An important aspect of neuronal function and communication in the central nervous system is the maintenance and refinement of synaptic networks through the selective pruning of synapses, which occurs predominantly, but not exclusively, during development. Interestingly, these processes are also triggered in neurodegenerative diseases and are thought to be responsible for the observed loss of synapses in these disorders, which include Alzheimer’s disease, Frontotemporal Dementia, and other related dementias. Astrocytes and microglia are known to contribute to synaptic pruning during development and thereby play an important role in activity-dependent synapse remodeling. Numerous pathways have been implicated in this process, including the activation of the complement cascade, which is proposed to prime the synapses for removal by exposing so-called ‘eat me’ signals onto neuronal synapses. Here, we sought to uncover “eat me”, but also ‘spare me’ signals deposited onto neuronal synapses in C9ORF72 Amyotrophic Lateral Sclerosis (ALS)/ Frontotemporal Dementia (FTD) using patient-derived induced pluripotent stem cells (iPSC) differentiated into cortical neurons in addition to postmortem autopsy tissue from C9ORF72-ALS/FTD patients. To enrich for synaptic proteins, a series of centrifuge-based fractionations were performed to obtain a synaptosome preparation including fractions containing both pre- and post-synapses, as well as an enriched post-synaptic fraction. Synaptosome preparations were generated from iPSC-cortical neurons and postmortem frontal cortex tissue samples. We validated synaptosome preparations from iPSC neurons and confirmed enrichment of presynaptic marker protein synaptophysin and postsynaptic protein Homer1 in the appropriate synaptosome fractions. At the same time, we generated synaptosomes from 4 healthy, non-neurological control and 6 C9ORF72 ALS/FTD frontal cortex autopsy brain tissues. The purified synaptosomes underwent deep expression proteomics profiling, which revealed on average 550 proteins/sample and 60,000 peptides/sample. Of those, 83 proteins were differentially abundant in C9ORF72-ALS/FTD synaptosome, with 23 being more abundant and 60 being less abundant. Among the 23 proteins to be more abundant were potential biomarkers for ALS/FTD, including TMEM41B, PTP4A3, WRN, MTFMT, HOXA2, KCNT1, SPRED2 and VWF. Gene set enrichment analysis revealed positive enrichment for (1) synapse assembly/organization, (2) neurogenesis and (3) neurotransmitter receptors and postsynaptic signal transmission, while negative enrichment was found in immune response pathways. Interestingly, ingenuity pathway analysis revealed a significant inhibition of the EIF2 signaling pathway, while there is increased activity in glutamatergic receptor signaling pathway, with the most upregulated proteins GABRA1, GABRA3 and GABAa receptor gene variants. Comparison of these proteomics data to recently published snRNA seq data from our laboratory revealed some overlapping aberrantly expressed gene expression, but also highlighted the discrepancies frequently found between RNA and protein analyses within the same tissue samples. Ongoing studies are aimed at validating the ex vivo patient tissue -omics data using the iPSC-cortical neuron synaptosome model, following by mechanistic assessments.

# Abstract: P02.12 – Neurochemical Perturbations and Synaptic Dysfunction Associated With Inhaled Environmental and Occupational Toxicants

**Published:** 2025-09-05

**Authors:** Krishnan Sriram, Gary Lin, Walter McKinney, Jeffrey Fedan, Ann Hubbs

**Affiliations:** a Centers for Disease Control and Prevention (CDC), National Institute for Occupational Safety and Health (NIOSH), Morgantown, VA, USA

## Abstract

Environmental and occupational toxicants deposited in the nose can reach the brain via olfactory or trigeminal nerve pathways and disrupt neurotransmitter signaling, cytoskeletal scaffolding, and synaptic structure. Impaired synaptic connectivity, plasticity, or loss of synapses are often associated with functional decline of the nervous system. Thus, evaluating such changes may provide insight into the early mechanisms underlying neurodegeneration seen in Alzheimer’s and Parkinson’s diseases. Here, we evaluated the neural effects of two toxicants that have the potential for large-scale human exposure. Consequent to the 2010 Deepwater Horizon (DWH) oil spill, there is growing concern regarding the long-term neurological risks of inhaled crude oil vapor (COV). To investigate this, rats were exposed to COV (300 ppm; 6 h/d × 4 d/w × 4 w). After 1, 28, or 90 d, changes in monoamine neurotransmitters and synaptic proteins were evaluated. COV reduced (20–40%) norepinephrine (NE) and dopamine (DA) in the dopaminergic brain areas, striatum (STR), and midbrain (MB) while increasing (232%) serotonin (5-HT) in the STR at distinct time points. COV augmented the expression of synaptophysin (SYP; 35–60%), synaptotagmin (SYT; 30–50%), and tyrosine 3-monooxygenase/tryptophan 5-monooxygenase activation protein epsilon (57%) in the STR or MB. Our findings suggest that an imbalance in monoaminergic signaling due to synaptic modulation may underlie neurotoxicity of COV. Hydraulic fracturing (“fracking”) is a process used to recover oil and gas from shale rock formation. Pressurized liquids containing water and sand (proppant) fracture the oil- and natural gas-laden rock. The transportation and handling of proppant at oil-well sites generate dust aerosols, raising concerns about human inhalation of fracking sand dusts (FSD). Thus, evaluating the neurotoxicity of FSD is essential from a health safety perspective. Therefore, rats were exposed to FSD (30 mg/m³; 6 h/d × 4 d). After 1, 7, 27, or 90 d, changes in monoamine neurotransmitters and synaptic proteins were examined in the olfactory bulb (OB), hippocampus (HIP), and cerebellum (CER). FSD increased NE (13–44%), DA (18–44%), and 5-HT (15–27%) in HIP and CER, while reducing 5-HT (33%) in OB at one or more time points. FSD upregulated (36–131%) SYP in the OB while transiently reducing (31%) its level in the STR. SYT protein transiently decreased (18–30%) in the OB and HIP. An intriguing observation was the persistent reduction (25–42%) of SYP and SYT proteins in the CER 90 d after cessation of exposure, suggesting synaptic disruption and neural injury. Our studies suggest that various chemically distinct environmental and occupational toxicants elicit olfactory and central neurotoxicity. Future studies may determine if the biochemical, molecular, and synaptic aberrations will culminate in neuropathology and neurodegeneration, leading to long-term behavioral and functional deficits.

# Abstract: P02.13 – TSG-6 Mediated Extracellular Matrix Modifications Regulate Hypoxic-Ischemic Brain Injury

**Published:** 2025-09-05

**Authors:** Taasin Srivastava, Parham Diba, Hung Nguyen, Steven Sowa, Gage Haden, Norah LaNguyen, William Reed-Dustin, Wenbin Zhu, Xi Gong, Edward Harris, Selva Baltan, Stephen Back

**Affiliations:** a Department of Pediatrics, Oregon Health and Science University, Portland, OR, USA; b Division of Anesthesiology and Perioperative Medicine, Oregon Health and Science University, Portland, OR, USA; c Department of Biochemistry, University of Nebraska-Lincoln, Lincoln, NE, USA

## Abstract

Proteoglycan dependent extracellular matrix (ECM) modifications regulate cellular homeostasis and can also sensitize tissues/organs to injury and stress. Hypoxic-Ischemic (H-I) injury disrupts cellular homeostasis by activating inflammation and attenuating regeneration and repair pathways. In the brain, the main component of the ECM is the glycosaminoglycan (GAG), Hyaluronic Acid (HA), but whether it can be modified to regulate cellular homeostasis and response to H-I injury is not known. In this report, employing both male and female mice, we demonstrate that proteoglycan, TNFα-stimulated gene-6 (TSG-6), is constitutively expressed in the brain from birth onwards and it differentially modifies ECM HA during discrete neurodevelopmental windows. ECM HA modification by TSG-6 enables it to serve as a developmental switch to regulate the activity of the Hippo pathway effector protein, Yes Associated Protein 1 (YAP1) in the maturing brain and response to H-I injury. Mice that lack TSG-6 expression display dysregulated expression of YAP1 targets, excitatory amino acid transporter 1 (EAAT1; GLAST) and 2 (EAAT2; GLT-1). Dysregulation of YAP1 activation in TSG-6-/- mice coincides with age- and sex-dependent sensitization of the brain to H-I injury such that neonates display an anti-inflammatory response in contrast to an enhanced pro-inflammatory injury reaction in adult males but not females. Our findings thus support that a key regulator of age- and sex-dependent H-I injury response in the mouse brain is modulation of Hippo-YAP1 pathway by TSG-6 dependent ECM modifications.

# Abstract: P02.14 – Reactive Astrocytes May Underlie Diminished Neurovascular Coupling After Stroke

**Published:** 2025-09-05

**Authors:** Teresa Stackhouse, Laura Knittel, Bradley Marxmiller, Steve Sullivan, Zhenzhou Li, Heather McConnell, Rachel De La Torre, Wenri Zhang, Randy Woltjer, Anusha Mishra

**Affiliations:** a Oregon Health and Science University, Portland, OR, USA; b General Hospital of Ningxia Medical University, Ningxia, China

## Abstract

A loss or attenuation of neurovascular coupling (NVC) is reported after stroke in patients. This attenuation is observed in regions far from the infarct and may have long-term consequences on energy supply and neuronal viability. Previous work from our lab showed that a potent vasoconstrictor, 20-HETE, inhibits capillary NVC after stroke. 20-HETE is elevated in patients after stroke, but the source of increased 20-HETE is not well-understood. I hypothesize that the increased production of 20-HETE after stroke is due to metabotropic glutamate receptor 5 (mGluR5) expression in reactive astrocytes. Activation of mGluR5 results in prolonged IP3-dependent release of Ca^2+^ from internal stores and large Ca^2+^ increases, which are required for 20-HETE production. The role of mGluR5 in NVC has been contested because of its expression pattern: mGluR5 is high during development, but largely absent in healthy adult astrocytes. However, mGluR5 is re-expressed after ischemic injury in reactive astrocytes. To test if mGluR5 is contributing to NVC impairment after stroke, I inhibited mGluR5 with the specific antagonist MTEP (1 µM) and uncovered large dilations in the ipsilesional, but not contralateral, hemisphere 24 hrs after stroke. In complementary experiments, applying an mGluR5 agonist, DHPG (20 µM) drove capillary constrictions in both hemispheres, with consistently stronger responses in the ipsilesional cortex. Together, these results indicate that mGluR5 drives capillaries to constrict and plays a role in reducing NVC in the first 24 h after stroke. While mGluR5 may play a role in neurovascular coupling at this acute time-point, we have yet to understand the spatiotemporal characteristics of reactive astrogliosis and its effects on NVC after stroke. Here, we also show that astrogliosis becomes more pronounced over time during the first week after stroke, especially in regions further from the infarct. Depleting microglia increases infarct size and limits, but does not abolish, astrogliosis. These results indicate a complex interaction between microglia and astrocytes following stroke in infarct formation. This work investigates the downstream effects of stroke-induced astrogliosis on NVC and the mechanisms driving this astrogliosis. Our finding that NVC is altered by a change in astrocytic mGluR5 expression, likely by increasing 20-HETE, suggest new strategies for rescuing NVC after stroke. Our data also indicate that astrogliosis may not entirely depend on microglial signals and that interactions between these glial cells can be context dependent and differ between different CNS injuries.

# Abstract: P02.15 – Unravelling the Metabolic Changes Mediated by Ketone Body Utilization in the Nervous System

**Published:** 2025-09-05

**Authors:** Marion Stunault, Arsalan Hashemiaghdam, Zirui Chen, Marissa Laramie, Kate Weathersby, Ghazaleh Ashrafi

**Affiliations:** a Washington University in Saint Louis, St. Louis, USA

## Abstract

The brain is a highly energetic organ and utilizes glucose as primary fuel resource. However, during intense physical activity, fasting, or sleep, glucose availability becomes limited and the brain can utilize alternative fuels such as lactate, pyruvate, and ketone bodies to sustain its activity. Three ketone bodies are naturally synthesized in the liver by breaking down fatty acids: Acetone, Acetoacetate (AcAc), and β-hydroxybutyrate (BHB). AcAc and BHB reach extra-hepatic tissues, including the brain, via the circulation. High fat, low carbohydrate ketogenic diets (KD) have gained great interest over the years due to their therapeutic effects in a wide range of brain diseases. Specifically, KD have been shown to be neuroprotective against neuronal loss and to significantly improve memory in Parkinson’s and Alzheimer’s diseases, mood stabilization in anxiety and depression disorders, and provide seizure protection in children with drug-resistant epilepsies. However, the mechanisms through which ketone bodies affect neuronal physiology remain poorly understood. We hypothesize that ketone bodies directly impact neuronal excitability by modulating synaptic transmission. Using primary isolated hippocampal neurons, we show that neuronal survival in BHB is dose- and age-dependent in vitro. In addition, neuron and astrocyte survival is optimized when both cell types are cultured together, implicating neuro-astrocytic metabolic interaction during ketone body utilization. To assess the impact of the ketone bodies on neuronal excitability, we performed quantitative optical analysis of synaptic function in hippocampal terminals. We found that synaptic vesicle recycling and evoked glutamate release were impaired in neurons supplied with ketones as compared to those with glucose or lactate. In contrast, presynaptic ATP levels remained unaffected by fuel type. We conclude that ketone bodies modulate neurotransmitter release and synaptic transmission, thereby suggesting a mechanism for the anti-epileptic effects of ketogenic diets.

# Abstract: P02.16 – Cell Synchronization and Glial Neurotransmitter Transporters in Retinal Müller Cells: Role in Excitotoxicity

**Published:** 2025-09-05

**Authors:** Temitayo Subair, Luisa C. Hernández-Kelly, Prof Arturo Ortega, Cristina Sandu, Marie-Paule Felder-Schmittbuhl

**Affiliations:** a CINVESTAV, Cuidad de Mexico, Mexico

## Abstract

Retinal degeneration is known to be the pathological hallmark of retinal diseases. Environmental toxicants are one major cause of the degeneration associated with the retina and these compounds range from exposure to methylmercury to blue light among others. The retina is a glutamatergic machine that contains several layers of neurons and interneurons involved in retinal physiology. Glutamate (Glu) is the main excitatory neurotransmitter of the central nervous system (CNS) and exerts its actions through the activation of receptors and transporters located within the layers of the retina. Glu is recycled in an uptake-release fashion by a series of excitatory amino acid transporters (EAATs) and Glu-cysteine exchangers (xCT). The uptake-release system of Glu is facilitated by a special glial system of the retina known as the Müller glial cells. However, an imbalance in the Glu homeostasis causes an increase in the extracellular concentration of Glu leading to neuronal death a process known as Glu excitotoxicity. In this study, the radioligand uptake assay method was used to evaluate the interplay between the two main neurotransmitters in the retina using the retina glial human cell line MIO-M1. The results show a dose and time-dependent decrease in the [3H]-D-Aspartate with GLAST accounting for about 70% of the glutamate uptake while Glt-1 accounted for about 24% uptake in the retina. Synchronized cells were also employed in the study, and this involved serum shock exposure to cells to evaluate the effect of clock genes on neurotransmitter uptake. MIO-M1 cells were exposed to 50% SFB DMEM to evaluate the effect of clock genes on [3H]-D-Aspartate uptake in the retina. The result revealed a considerable increase in the uptake of the synchronized cells when compared to the unsynchronized cells. A pharmacological blocker of glutamate transporters such as dihydro-kainic acid also triggers similar effects in both the synchronized and unsynchronized cells. The main inhibitory neurotransmitter in the CNS, GABA also showed a similar trend in its uptake in synchronized cells indicating the plausible role of clock genes in the uptake of these neurotransmitters. Preliminary results showed that the upregulation of clock genes could play a major role in the uptake and recycle paradigm of neurotransmitters in the retina in relation to the light-dark cycle where different concentrations of neurotransmitters are released by the retinal neurons in maintaining normal retina function.

# Abstract: P02.17 – Chronic In Vivo Calcium Imaging of Registered Spinal Neurons to Characterize Mechanical Sensitization During a Month of Inflammatory Pain

**Published:** 2025-09-05

**Authors:** Steve Sullivan, Andrei Sdrulla

**Affiliations:** a Oregon Health and Science University, Portland, OR, USA

## Abstract

Injury and inflammation can induce long-term changes of the nervous system that give rise to chronic pain. It is thought that plasticity within the superficial dorsal horn (SDH) of spinal cord, where somatosensory information from primary afferents first converges with the central nervous system, acts to amplify nociceptive signaling to pain centers of the brain. Recent advances in in vivo optical recording methods have revealed how SDH neurons are recruited by natural innocuous and noxious stimuli. However, to date, no published studies have characterized how the activity patterns of individual SDH neurons evolve over extended periods of pain. Here we present a method for chronically tracking the calcium activity of registered neurons in mice for one month. In a Complete Freund’s Adjuvant (CFA) inflammatory pain model, excitatory SDH neurons showed early signs of sensitization to innocuous touch stimuli that persisted throughout the testing period. By one week, a brief noxious pinch stimulus (1 sec) induced fits of activity that lasted for nearly 1 min. On closer examination of individual neurons, it was found that these population-level changes were non-uniformly distributed, with some neurons showing more sensitization to a given stimulus than others. Combining this history of functional remapping with behavior studies may provide insights into the origins of individual variation in pain processing.

# Abstract: P02.18 – Extracellular Vesicles From Toxoplasma gondii Infected Neurons Decrease GLT-1 Protein Expression

**Published:** 2025-09-05

**Authors:** Emily Tabaie, Ziting Gao, Wenwan Zhong, Emma Wilson

**Affiliations:** a Division of Biomedical Sciences, UCR, Riverside, CA, USA; b Department of Chemistry, UCR, Riverside, CA, USA

## Abstract

Toxoplasma gondii is an obligate intracellular parasite that can invade any nucleated cell, however, cyst formation in the central nervous system (CNS) only occurs in neurons. Previously our lab discovered that murine primary cortical neurons infected with T. gondii resulted in a downregulation of astrocytic GLT-1. This transporter is responsible for ∼90% of extracellular glutamate uptake, the primary excitatory neurotransmitter in the brain. In vivo astrocytes are one CNS cell type that is able to evade cyst formation, but their presence is fundamental to neuronal health by providing nutrients, structural support, and regulation of neurotransmitters. Although previous work has demonstrated defects in neuronal physiology, it is not yet known how infection disrupts the crucial communication between astrocytes and neurons. Extracellular vesicles (EVs) function in intracellular communication and contain proteins, lipids, DNA, miRNA, and other RNAs subtypes. Normal expression of GLT-1 is driven and regulated by post-translational modifications and neuronal release of EVs. Toxoplasma infection of neurons leads to many changes in neuronal function. It is therefore possible that changes in EV production by neurons is responsible for the downregulation of astrocytic GLT-1 observed during infection. To evaluate if EVs from T. gondii infected neurons will alter astrocyte and neuron communication, primary neuronal cultures were infected and EVs were harvested and analyzed for EV morphology, size, and concentration. TEM images confirm EV morphology and no distinct difference in EV size between the two groups. However, the neuronal production of EVs was significantly reduced following infection, as measured by NTA and CD63 ELISA. To determine if EV-dependent neuronal-astrocyte communication was altered by infection, EV uptake and bulk RNA Sequencing was conducted on astrocytes incubated with EVs from uninfected or infected neurons. Results support EV uptake by astrocytes along with EV regulation of astrocytic GLT-1, in addition to pinpointing other genes regulated by this process. Furthermore, protein analysis of astrocytes incubated with neuronal-derived EVs demonstrate a significant decrease in GLT-1 protein concentration after the addition of EVs from infected neurons. These results support the concept that Toxoplasma infection causes changes in the production and content of neuronal derived EVs and helps better understand how a parasitic infection in the brain alters neuronal-astrocyte communication.

# Abstract: P02.19 – Lanthionine Ketimine Ethyl Ester Increases Oligodendrocyte Proliferation Both In-Vitro and In-Vivo

**Published:** 2025-09-05

**Authors:** Ankit Tandon, Intakhar Ahmad, Jacqueline Rocha, Zachary McDonald, Douglas Feinstein

**Affiliations:** a Department of Anesthesiology, University of Illinois Chicago (UIC), Chicago, IL, USA; b Jesse Brown VA Medical Center, Chicago, IL, USA

## Abstract

Lanthionine ketimine ethyl ester (LKE) is a synthetic bioavailable cell penetrating derivative of the naturally occurring metabolite, lanthionine ketimine. Several studies have shown neuroprotective effects of LKE in attenuating pathology in animal models of Multiple Sclerosis (MS), Alzheimer’s Disease, spinal cord injury; as well as providing neuroprotection and induction of oligodendrocyte (OLG) maturation in-vitro. The basic objective of this work is to study the effects of LKE on OLGs using mouse primary OLGs in-vitro, and in the cuprizone (CPZ)-induced demyelination mouse model of MS. Briefly, primary OLG progenitor cells (OPCs) were cultured in-vitro from post-natal day 0–2 mouse pups. The brains were dissected, trypsinized, then plated in OPC specific medium containing platelet-derived growth factor (PDGF-AA) and basic fibroblast growth factor (bFGF). The OPCs were seeded in poly-D-lysine coated chamber slides and treated with vehicle or LKE (10 µM) for 72h, fixed, then used for immunocytochemical analysis for PDGFRα to label immature OLGs, and proliferating cell nuclear antigen (PCNA) to measure cell proliferation. Similarly, primary neural stem cells (NSCs) were cultured and treated with LKE for 72h via the same protocol, plated in NSC medium containing bFGF and epidermal growth factor (EGF), then analyzed for Sox2 and Ki67 immunoreactivity. Immunocytochemical analysis revealed an increase in the number of PDGFRα+/PCNA + OPCs indicating increased OPC proliferation in the LKE treated group as compared to control group. Similar analysis was done for Sox2+/Ki67+ NSCs and comparable differences were observed between the two groups. To examine effects of LKE on OLG proliferation in-vivo, female C57BL/6 mice were placed on 0.2% CPZ chow for either 3 or 6 weeks, and then remyelination allowed to occur for 2 weeks on control chow or chow containing 100 ppm LKE. Brains were collected, then dissected into specific regions corpus callosum (CC), hippocampus (HC), and cerebellum (CB) to be used for immunostaining and RNA analysis. Initial RNA-seq analysis of CC RNA indicated that LKE treatment increased the expression of proliferation promoting (e.g., BDNF) and differentiation inhibiting (e.g., Lingo1, NOGO receptor) mRNAs. These preliminary results demonstrate the proliferative action of LKE on OPCs as well as NSCs, which may contribute to its known neuro-protective and OPC maturation roles. On going qPCR and immunostaining studies are being carried out to confirm RNAseq results and to determine the cellular localization of mRNAs that are upregulated by LKE during remyelination.

# Abstract: P02.20 – Investigating the Roles of Dark/Clec7a + Microglia During Early Postnatal Development in a Mouse Model of Maternal Immune Activation

**Published:** 2025-09-05

**Authors:** Marianela Traetta, Sophia Loewen, Colin Murray, Mohammadparsa Khakpour, Colby Sandberg, Marie-Ève Tremblay

**Affiliations:** a University of Victoria, Victoria, Canada

## Abstract

Epidemiological studies have associated prenatal inflammation with a higher risk of neurodevelopmental disorders. In mice, we have previously shown that a model of maternal immune activation (MIA) induced with the viral mimetic polyinosinic:polycytidylic acid (Poly I:C) at embryonic day (E) 9.5 presents altered behaviors reminiscent of schizophrenia. Microglia, the immune cells of the brain which are critical for a proper neurodevelopment have emerged as key players linking inflammation to neurodevelopmental disorders. Various microglial states were identified, such as dark microglia, which are abundant during development, rare in healthy adults, and increase in numbers with pathology, including MIA. However, the roles of dark microglia remain elusive. Our hypothesis is that dark microglia play key roles during neurodevelopment that become deleterious with inflammation and participate in the pathological mechanisms of MIA. First, we will characterize dark microglia’s abundance and interactions within the brain parenchyma during neurodevelopment in health and MIA. Using the mouse model of MIA induced with Poly I:C at E9.5, we study male and female offspring, starting with males in which MIA induces more pronounced behavioral outcomes and where dark microglia are abundant in adulthood. We examine different time points during early postnatal development. We use scanning electron microscopy (SEM) to assess the density and distribution of dark microglia in the hippocampus (CA1 and dentate gyrus). We also analyze dark microglia’s interactions with other parenchymal elements including synapses and blood vessels to shed light onto their potential functions. Our preliminary findings at postnatal day (P)10 indicate that dark microglia downregulate classical microglial markers yet they selectively express Clec7a during neurodevelopment. Thus, we will explore the expression of the pattern-recognition receptor Clec7a by performing immunocytochemical SEM. We will quantify dark microglia (vs other microglia) expressing Clec7a in the hippocampus at P10, comparing health and MIA. In addition, we will perform a double immunohistochemistry for Iba1 and Clec7a to be analyzed by brightfield microscopy in order to determine the distribution of Clec7a + cells across the hippocampus, starting with P10, in health and MIA, which will pave the way to determine the role of microglial Clec7a during neurodevelopment and following MIA. Overall, we will study the outcomes of dark microglia/ Clec7a + microglia in health and MIA, providing insight into their roles during MIA and be able to suggest in the long-term a potential therapeutic target to treat or prevent neurodevelopmental disorders.

# Abstract: P02.21 – Fluid Flow Shear Stress Induces Acid Sphingomyelinase-Mediated Secretion of Neurotoxic Ceramide-Rich Extracellular Vesicles From Cilia in Glial Cells

**Published:** 2025-09-05

**Authors:** Priyanka Tripathi, Zainuddin Quadri, Xiaojia Ren, Zhihui Zhu, Liping Zhang, Simone M. Crivelli, Ahmed Elsherbini, Stefka D. Spassieva, Erhard Bieberich

**Affiliations:** a University of Kentucky, Lexington, KY, USA; b Veterans Affairs Medical Center, Lexington, KY, USA

## Abstract

Cells in tissues experience various levels of shear stress due to extracellular fluid flow. While numerous studies show activation of SMases and ceramide elevation by oxidative stress, the effect of shear stress on the lipid composition of the plasma membrane is vastly understudied. We tested the response of primary cultured mouse astrocytes and ependymal cells to fluid flow shear stress using the orbital shaker model. Shear stress led to increased immunolabeling of the sphingolipid ceramide and GFAP in astrocytes, concurrent with decreased glutathione (GSH), an endogenous inhibitor for acid sphingomyelinase (ASM), an enzyme generating ceramide. Restoring GSH with N-acetyl cysteine or inducing proteolytic degradation of ASM with fluoxetine prevented ceramide generation and astrocyte activation. Shear stress reduced the number of primary and motile cilia in astrocytes and ependymal cells, respectively. Loss of cilia by shear stress was prevented by fluoxetine and Arc39, a specific inhibitor of ASM. Cilia co-labeled for ceramide and ASM, indicating that shear stress induced localization of ASM to the ciliary membrane, which was not observed in non-stressed cells. ASM localization to cilia was prevented by thioperamide, a histone 3 receptor agonist which increasing a lysolipid that retained ASM in lysosomes. Shear-stressed cilia showed increased co-labeling for Arl13b, a ciliary transport protein, and glutamylated tubulin, a tubulin modification found in secretory cilia. Shear stress induced the secretion of ceramide-rich extracellular vesicles (CREVs), which was prevented by a pan-TRPV calcium channel blocker (gadolinium chloride), Arc39, fluoxetine, and thioperamide. This data indicated that ASM localization to cilia and ceramide generation led to vesicular shedding of the ciliary membrane, which was consistent with the presence of ASM and ceramide in EVs. EVs secreted by shear stressed glia induced cell death in neuronal (N2a) cells, unless CREVs were removed with anti-ceramide antibody. In conclusion, these data indicate that shear stress leads to the localization of ASM to the ciliary membrane and secretion of CREVs that are neurotoxic. This work was supported by the NIH grants RF1AG078338 and R21AG078601, and VA I01 BX003643.

# Abstract: P02.22 – Biomarker and Edema Attenuation in IntraCerebral Hemorrhage (BEACH): Phase 2a Proof-of-Concept Trial of a Novel Anti-Neuroinflammatory Small Molecule Drug Candidate

**Published:** 2025-09-05

**Authors:** Linda Van Eldik, Wendy Ziai, Lauren Sansing, Daniel Hanley

**Affiliations:** a University of Kentucky, Lexington, KY, USA; b Johns Hopkins University School of Medicine, Baltimore, MD, USA; c Yale University School of Medicine, New Haven, CT, USA

## Abstract

**Background:**

Non-traumatic spontaneous intracerebral hemorrhage (ICH) is a major medical problem with few effective therapies. ICH causes considerable mortality and morbidity, especially in older adults, possibly due to increased prevalence of vascular co-morbidities, cerebral amyloid angiopathy, and hypertension. In addition to the primary injury caused by the hemorrhage and hematoma expansion, secondary neuroinflammatory events after ICH can further damage the brain and lead to increased risk of neurologic complications including Alzheimer’s disease and related dementias. Previous work [PMCID: PMC6093844; PMCID: PMC8252865] suggests that the robust proinflammatory cytokine increase in the brain that occurs in the first few days after injury is a key contributor to cerebral edema, long-term neurological damage, and cognitive deficits. The mechanistic linkage of the acute cytokine surge to progression of ICH-induced injury, plus the attractive therapeutic time window of hours to days post-injury, provide a rational therapeutic target for intervention in the acute care setting. To address the clear and urgent need for interventions that improve neurologic recovery and outcomes, we developed [PMCID: PMC1868432] the investigational drug candidate, MW01-6-189WH (MW189). MW189 is a novel, CNS-penetrant, small molecule developed as a selective suppressor of injury- and disease-induced proinflammatory cytokine overproduction associated with destructive neuroinflammation/synaptic dysfunction cycles. In animal models of acute brain injury, MW189 at low doses attenuated neuroinflammation, reduced cerebral edema, and improved functional and cognitive performance [PMID: 22487803]. MW189 was also safe and well tolerated in phase 1a and phase 1b clinical trials in healthy adults [PMCID: PMC7541708], supporting the further development of MW189 for patients with acute brain injury. Therefore, we designed the first-in-patient exploratory trial with the aim to determine safety and tolerability of MW189 in patients with acute spontaneous ICH.

**Methods:**

The Biomarker and Edema Attenuation in IntraCerebral Hemorrhage (BEACH) trial is a first-in-patient phase 2a, proof-of-concept study of MW189 in patients with ICH (NCT05020535). This multicenter, prospective, randomized, double-blind controlled trial will enroll 120 non-traumatic ICH participants, with an anticipated average age in their mid-60s and substantial numbers of individuals with cerebral small vessel disease and cerebral amyloid angiopathy. Patients will be randomly assigned 1:1 to receive intravenous MW189 (0.25 mg/kg) or placebo (saline) within 24 h of symptom onset and every 12 h for up to 5 days or until hospital discharge.

**Results:**

The BEACH trial is actively enrolling participants. The primary outcome is all cause-mortality within 7 days post-randomization between treatment arms. Secondary endpoints include all-cause mortality at 30 days, perihematomal edema volume after symptom onset, adverse events, vital signs, pharmacokinetics of MW189, and proinflammatory cytokine concentrations in plasma (and cerebrospinal fluid if available). Other exploratory endpoints are functional outcomes collected on days 30, 90, and 180.

**Conclusions:**

Success with MW189 in ICH patients will further de-risk the compound for subsequent larger trials of acute CNS injury and/or to develop the drug for Alzheimer’s and other age-related dementias. The study will also generate important information about the utility of targeting the acute proinflammatory cytokine aspects of neuroinflammation in older adults with vascular disease.

# Abstract: P02.23 – HIV Antiretroviral Drugs Trigger Stress Granule Formation via the PERK Activated Integrated Stress Response in Differentiating Oligodendrocytes

**Published:** 2025-09-05

**Authors:** Eliana von Krusenstiern, Lindsay Festa, Lindsay Roth, Sophie Levin, Kelly Jordan-Sciutto, Judith Grinspan

**Affiliations:** a University of Pennsylvania, Philadelphia, PA, USA; b Children’s Hospital of Philadelphia, Philadelphia, PA, USA

## Abstract

Approximately half of people with HIV (PWH) experience HIV-Associated Neurocognitive Disorder (HAND), a spectrum of neurocognitive impairments. While more than half of PWH in the United States are virally suppressed or undetectable, the overall proportion of PWH with HAND symptoms has remained unchanged. One of the persistent pathologic features of HAND in the era of antiretroviral therapy (ART) is white matter abnormalities. The duration of ART treatment in patients has been shown to correlate with the observed thinning of the corpus callosum, suggesting that ART drugs may be contributing to this pathology. Previous work in our lab has demonstrated that select ART drugs prevent the maturation of oligodendrocytes (OLs) and remyelination. We have demonstrated that treating maturing OLs with ART drugs, such as Elvitegravir (EVG), activates the Integrated Stress Response (ISR) and that co-treatment with the ISR inhibitor ISRIB rescues differentiation. Consistent with ISR activation, here we show that OL treated with ART drugs during differentiation leads to the formation of cytoplasmic stress granules (SGs), as shown via immunohistochemical staining of G3BP1, an RNA-binding protein that is ubiquitous to SGs. During ISR, which inhibits global protein translation, SGs sequester proteins, mRNAs and translation machinery critical for cell survival following resolution of the stress. While SGs have yet to be thoroughly studied in OLs, their chronic presence in neurons is hypothesized to cause acceleration of neurodegeneration in a variety of neurodegenerative diseases. Ex vivo analysis of both mice treated with the ART drug, bictegravir, and small cohort of PWH, revealed the presence of SGs within oligodendrocytes of the corpus callosum and cortical white matter, respectively; In post-mortem tissue of PWH with and without HAND diagnoses, we observed an increased presence of SGs in PWH with HAND compared with neurocognitively normal individuals. In vitro, we demonstrate that when differentiating OLs are co-treated with ART drugs and ISRIB, SGs do not form, indicating that these SGs are formed canonically via the ISR. We further show that co-treatment with an inhibitor of ISR kinase, PERK, decreases the percentage of SG-positive OLs, specifically implicating PERK as responsible for ISR activation in ART treated OLs as well as SG formation. These findings suggest that formation of SGs in OLs may contribute to persistent white matter pathology in PWH with HAND. These studies are among the first to study stress granules in OLs, and have the potential to contribute to the improvement of patient outcomes for PWH on ART.

# Abstract: P02.24 – Astrocytes Dysregulate GABAergic Inhibition to Impair Auditory Processing in Fragile X Syndrome

**Published:** 2025-09-05

**Authors:** Victoria Wagner, Alexandra Varallo, Anna Anna O. Kulinich, Samantha Sutley-Koury, Maham Rais, Walker Woodard, Xinghao Shuai, Timo Piepponen, Maija Castren, Khaleel Razak, Iryna Ethell

**Affiliations:** a University of California, Riverside, Riverside, CA, USA; b University of Helsinki, Helsinki, Finland

## Abstract

Fragile X syndrome (FXS) is a leading genetic cause of autism-like symptoms, including sensory hypersensitivity and cortical hyperexcitability, resulting from epigenetic silencing of the Fragile X messenger ribonucleoprotein (Fmr1) gene. Recent observations in humans and Fmr1 knockout (KO) animal models of FXS suggest symptoms are mediated by abnormal GABAergic signaling. As most studies have focused on neuronal mechanisms, the role of astrocytes in mediating defective inhibition in FXS is largely unknown. We found that human FXS astrocytes derived from patient-specific induced pluripotent stem cells (iPSCs) show ∼7-fold increase in GABA levels compared to their control counterparts using high-performance liquid chromatography (HPLC). Similar to FXS human astrocytes Fmr1 KO mouse astrocytes showed increased levels of GABA, potentially due to an up-regulation of GABA-synthesizing enzymes GAD65/67 assessed with western blotting and immunostaining. Finally, we found that astrocyte-specific Fmr1 deletion during P14-P28 period reduces inhibitory connections in the cortex, leading to long-term changes in cortical responses with impaired sound-evoked synchronization to gamma frequencies, but enhanced background gamma power and reduced habituation to sound using EEG recordings. Our findings suggest astrocytic FMRP has a key role in the development and function of inhibitory circuits and astrocytes are a valuable target for therapies to relieve FXS-associated phenotypes.

# Abstract: P02.25 – Investigating the Role of Copper in Oligodendrocyte Myelination

**Published:** 2025-09-05

**Authors:** Johnathan Wong, Judith Grinspan, Kelly Jordan-Sciutto

**Affiliations:** a University of Pennsylvania, Philadelphia, PA, USA; b Children’s Hospital of Philadelphia, Philadelphia, PA, USA

## Abstract

Oligodendrocytes are glial cells responsible for synthesizing myelin, a lipid-enriched membrane crucial for facilitating the rapid transmission of action potentials and providing trophic support to axons. Consequently, the loss of myelin can contribute to motor and neurocognitive dysfunction in demyelinating diseases such as multiple sclerosis (MS). Diseases involving an excess or deficiency in cytoplasmic copper (Wilson’s and Menkes disease, respectively) have also been characterized by impaired myelination of axons in the CNS. Therefore, it is assumed that oligodendrocytes could require copper in order to form myelin. In fact, one of the gold standard models used to experimentally induce demyelination involves cuprizone, a copper chelator. Described as early as the 1950s, the cuprizone model induces demyelination in animals, primarily in the corpus callosum. Though its copper chelating actions suggest a copper-based role, the exact mechanism by which cuprizone induces demyelination remains unclear. In this study, we aim to investigate the effect of cuprizone on oligodendrocytes and myelination in vitro and elucidate if a different ion’s chelation, such as iron, would produce similar effects. Specifically, we explore the impact of these chelators on myelin formation and maintenance, as well as the differentiation and survival of oligodendrocytes. I hypothesize that copper chelation specifically disrupts oligodendrocyte function, leading to demyelination. The results of this study will provide valuable insights into the effects of copper homeostasis on oligodendrocytes.

# Abstract: P02.26 – PTEN-Induced Kinase 1 Modulates Neuronal Development and Plasticity Through Calcium/Calmodulin-Dependent Protein Kinase II and IV

**Published:** 2025-09-05

**Authors:** Micah Woodruff, Smijin Soman, Anirudh Praveen, Ruben Dagda

**Affiliations:** a Reno School of Medicine, University of Nevada, Reno, NV, USA

## Abstract

Parkinson’s disease (PD) is a progressive neurodegenerative disease that results in motor control dysfunction. Certain forms of PD exhibit non-motor symptoms such as cognitive decline, due in part to an overt atrophy of the hippocampus and cortex. Mutations of PTEN-induced Kinase 1 (PINK1), a serine/threonine (ser/thr) kinase, are associated with early-onset, familial forms of PD. In healthy neurons, PINK1 plays an important role in governing neuronal development by regulating mitochondrial recycling and trafficking, dendrite development, and brain-derived neurotrophic factor (BDNF) regulation. Full-length PINK1 (fl-PINK1) is primarily anchored to the outer mitochondrial membrane but gets imported to the mitochondrion, cleaved to a lower molecular weight form, and shuttled to the cytoplasm to exert critical functions. Our research group has shown that the cleaved form of PINK1 (c-PINK1) can exert important neuronal functions via downstream activation of Protein Kinase A (PKA) including synaptic plasticity and neuronal development. Although extensive progress has been made in eliciting the functions of fl-PINK1 in healthy neurons, there is still limited knowledge on the molecular mechanisms by which c-PINK1 promotes synaptic plasticity and neuronal development. Emerging evidence from our research group suggests that pharmacological activation of PINK1, via treatment of primary neurons with kinetin triphosphate (KTP), a pharmacological activator of PINK1, is associated with increased phosphorylation of calcium/calmodulin-dependent protein kinase II and IV (CaMKII/IV). CaMKII/IV are kinases that respond to changes in cytosolic calcium levels and regulate critical cellular processes, including gene transcription, cell survival, cytoskeletal reorganization, and learning and memory. Based on our preliminary data, we hypothesized that c-PINK1 phosphorylates CaMKII and CaMIV to stimulate synaptic plasticity and neuronal development by promoting dendrite outgrowth. By employing Western blotting and immunohistochemistry approaches, we observed that endogenous PINK1 activation, via KTP treatment of primary cortical and hippocampal neurons, increased the phosphorylation of PINK1, a proxy of PINK1 activity, in addition to increasing the phosphorylation of CAMKII and CAMKIV. The increase in phosphorylation is concomitant with activation of PKA in addition to increases in the levels of mature BDNF, translocation of CAMKIV to the nucleus, and significantly increased formation of synaptic spines in hippocampal neurons. Conversely, pharmacological inhibition of CaMKII/IV, via co-treatment of primary cortical and hippocampal neurons with KN93, mitigated the effects of PINK1 on CAMKII/IV phosphorylation, formation of dendritic spines, and levels of BDNF. Furthermore, immunoprecipitation/Western blot data suggest that endogenous PINK1 associates with CaMKII and IV revealing them to be possible substrates of PINK1. In aggregate, our data show that PINK1 regulates the activation status of CAMKII/IV, highlighting a novel mechanism through which PINK1 may regulate synaptic plasticity, neuronal development, and cognition. Future work will focus on further establishing CaMKII-dependent PINK1-mediated dendrite growth and development and the colocalization of endogenous c-PINK1 and CaMKII/IV. These results provide supporting evidence for the important regulatory role PINK1 plays within healthy neurons to promote neuronal survival and development. This research was funded by the National Institute of Health under award number 2R01NS105783-05 and the Department of Undergraduate Research at the University of Nevada, Reno through the Nevada Undergraduate Research Award.

# Abstract: P02.27 – Investigating Ischemic Vascular Dysfunction in Alzheimer’s Disease

**Published:** 2025-09-05

**Authors:** Simone Woodruff, Ozama Ismail, Marty Pike, Wenri Zhang, Randy Woltjer, Laura Villasana, Bradley Marxmiller, Anusha Mishra

**Affiliations:** a Oregon Health and Science University, Portland, OR, USA; b Legacy Research Institute, Portland, OR, USA

## Abstract

Vascular dysfunction is one of the earliest pathological signs in the progression of Alzheimer’s disease (AD). Ischemic injuries cause long-lasting vascular dysfunction in affected individuals and increase a patient’s risk of developing dementia. Importantly, almost half of AD patients show indications of prior ischemia, emphasizing the importance of studying these conditions concurrently to better understand AD and related dementias (ADRD). However, the mechanisms and factors that initiate and perpetuate vascular dysfunction in ADRD are still unclear. We hypothesize that ischemic injury triggers widespread cerebrovascular dysfunction and accelerates the progression of AD. We are investigating this hypothesis using an innovative mixed model of dementia, in which 6-month-old Tg2576 AD-like model mice receive transient mild subcortical ischemia (tMSCI) via a 30-min middle cerebral artery occlusion. This is a particularly relevant model as subcortical strokes are the most common strokes in AD patients that correlate with dementia. Our data demonstrate that even a mild ischemic event in adult AD and wildtype (WT) mice leads to chronic bilateral vascular dysfunction that persists with age. In addition, results indicate persistent reactive astrocytes after ischemic injury – astrogliosis is pronounced 8 months after tMSCI in both WT and Tg2576 mice. Finally, ischemia increased parenchymal amyloid beta plaque pathology, but not cerebral amyloid angiopathy, in Tg2576 mice. Our results reveal that mild ischemia leads to bilateral vascular dysfunction and persistent glial pathology at a chronic timepoint post-ischemia. These pathologies are exacerbated and coincide with increased amyloid pathology in an AD mouse model. We are following this up with an ongoing study using a cross-sectional design with assessments of disease progression, including cognition, vascular function, and histopathology, at multiple timepoints after ischemia. Our goals are to understand how this cerebrovascular dysfunction initiates and progresses over lifespan and to correlate AD-associated cognitive decline with pathophysiology. This project could reveal insights into the mechanism of ischemia-induced chronic neurovascular impairments and how they contribute to the development of AD-like dementia.

# Abstract: P02.28 – Delineating the Mechanisms Leading to Aberrant Lipid Storage and Trafficking Defects in Astroglia Models of Cholesterol Synthesis Disorders

**Published:** 2025-09-05

**Authors:** Jazmine Yaeger, Keiji Kawatani, Sonali Sengupta, Megan Raszler, Amelia Lawver, Takahisa Kanekiyo, Kevin Francis

**Affiliations:** a Cellular Therapies and Stem Cell Biology Group, Sanford Research, Sioux Falls, SD, USA; b Department of Neuroscience, Mayo Clinic, Jacksonville, FL, USA; c Sanford Program for Undergraduate Research (SPUR) Scholar from Dordt University, Sioux Falls, SD, USA; d Sanford Program for Undergraduate Research (SPUR) Scholar from Bethel University, Arden Hills, USA; e Department of Pediatrics, Sanford School of Medicine, University of South Dakota, Sioux Falls, SD, USA

## Abstract

Cholesterol is critical to the CNS, providing structural integrity to membranes, anchoring proteins and carbohydrates to membranes, functioning as a precursor in steroidogenesis, and regulating distinct signaling pathways. Transport of dietary cholesterol across the blood-brain-barrier is limited, making CNS sterol biosynthesis imperative to brain health. Numerous neurodevelopmental and neurodegenerative diseases exhibit disequilibrium in cholesterol homeostasis. For example, Smith-Lemli-Opitz syndrome (SLOS), a rare genetic disorder where cholesterol biosynthesis is perturbed due to mutation of 7-dehydrocholesterol reductase (DHCR7), is characterized by intellectual disability, motor dysfunction, and autism. Despite the known roles for cholesterol within the CNS, the cellular impairments that result from cholesterol disequilibrium remain elusive. Since the trafficking and transport of synthesized cholesterol is important for maintaining a homeostatic lipid environment, we predicted disruption of sterol synthesis within glia would impair cellular transport and induce storage-associated stress. We first assessed the biochemical consequences of impaired cholesterol biosynthesis in genetic and pharmacological glial models, demonstrating a reduction in cholesterol production and an increase in the sterol precursor 7-dehydrocholesterol (7-DHC) relative to controls. Next, we analyzed the formation of lipid droplets (LD), storage organelles for neutralized fatty acids and cholesterol. We found astrocytes and microglia from Dhcr7 mutant mice accumulate LDs relative to controls. A similar effect was observed in astrocytes derived from SLOS patient induced-pluripotent stem cells (iPSCs). Perilipin-positive LDs physically associated with KDEL + endoplasmic reticulum (ER), suggesting ER storage. Accumulation of LDs was promoted in all cell types through challenge with oleic acid (OA) or lipopolysaccharide (LPS). In Dhcr7 astrocytes, large ER dilations were observed that were similarly observed after OA challenge in controls. These ER structural malformations suggested a link between cholesterol biosynthesis dysfunction and ER stress. We next analyzed lipid transport through endosomal sorting, lysosomal fusion, and secretion. Both genetic and pharmacological inhibition of cholesterol biosynthesis led to an accumulation of Rab5+ early endosomes and Rab7+ late endosomes. Inhibiting cholesterol synthesis also increased the production and release of sterol enriched extracellular vesicles (EVs) following cholesterol disruption. While endosomal accumulation and increased EV release coincide with enhanced autophagosome formation observed by transmission electron microscopy (TEM) and microtubule-associated proteins 1 A/1B light chain 3B (LC3B) accumulation, autophagolysosome formation was impaired. Additionally, SLOS iPSC-derived astrocytes exhibited impaired apolipoprotein E3 (ApoE3) secretion, limiting neuronal response to environmental stress. We believe inhibited glial cholesterol synthesis prevents effective membrane fusion required for autophagic processes, causing ER stress and roadblocks in endosomal sorting, ultimately leading to glial dysfunction and neurological phenotypes.

# Abstract: P02.29 – Role of Autophagy and Fatty Acid Binding Protein 5 During Docosahexaenoic Acid Inhibition of Palmitic Acid-Induced Lipotoxicity

**Published:** 2025-09-05

**Authors:** Francis Zamora, Jo-wen Liu, Frankis Almaguel, Marino De Leon

**Affiliations:** a Loma Linda University, Loma Linda, CA, USA

## Abstract

High levels of saturated fatty acids elicit lipotoxicity (LTx) in nerve cells, with palmitic acid (PA) as the predominant type in Western diets, whose lipotoxic effects may contribute to the onset of chronic neuropathic pain (NP). We have previously reported that in Schwann cells, PA-LTx causes decreased calcium in the endoplasmic reticulum (ER), followed by ER stress, mitochondrial membrane depolarization, and apoptotic cell death. However, PA-LTx is inhibited when Schwann cells are co-treated with calcium chelator BAPTA-AM, antioxidant MC-186, or polyunsaturated fatty acid docosahexaenoic acid (DHA). In vivo, we have shown that an omega-3 fatty acid-enriched diet promotes an anti-inflammatory metabolomic profile and significantly reduces NP. The present study examines the potential role of autophagy during PA-LTx in immortalized SCs (iSCs). Here, LTx was induced by exposing iSC cultures to 300 µM PA: 150 µM bovine serum albumin (2:1 ratio) for 24 and 48 h. Cells were also co-treated with 50 µM DHA to inhibit LTx or Chloroquine (CQ) to inhibit autophagic flux. Protein levels of autophagy marker LC3-II, autophagic flux marker p62/SQSTM1, and fatty acid binding protein 5 (FABP5) were assessed using Western blot. Real-time qPCR measured the expression of autophagy-related gene 12 (ATG 12) and FABP5, and cell viability was assessed using a WST-1 assay. Consistent with previous findings, PA treatment decreased cell viability, while co-treatment with DHA fully protected against PA-LTx. CQ alone did not affect cell viability; however, CQ + PA exacerbated PA-LTx-induced cell death. DHA protection from PA-LTx was not affected by CQ during the first 24 h of exposure. However, by 48HRs, CQ significantly decreased DHA’s protection against PA-LTx, suggesting the importance of autophagic flux in DHA’s mechanisms. Assessment of autophagy activity revealed that PA increased LC3-II and ATG 12 expression, while DHA normalized these levels. p62/SQSTM1 levels were significantly increased by PA at 24 and 48 h, suggesting an impairment of autophagic flux during PA-LTx. In contrast, DHA co-treatment reduced p62 accumulation while protecting against PA-LTx. Lastly, iSCs exposed to PA had significantly increased FABP5 mRNA and protein levels, but this up-regulation was not observed in the presence of DHA co-treatment. CQ treatment significantly increased FABP5 expression, supporting a link between FABP5 and autophagic flux. Our data suggests that in iSCs, PA-LTx induces an initial increase in autophagy as part of a cell-survival response and its inhibition may exacerbate apoptotic cell death. Further, DHA protection from PA-LTx may include promoting normal autophagic flux.

# Abstract: P02.30 – The Effect and Mechanism of GDNF Released From Reactive Astrocytes on Neuronal and Brain Protection After Ischemic Stroke

**Published:** 2025-09-05

**Authors:** Zhe Zhang, Nannan Zhang, Shinghua Ding

**Affiliations:** a Department of Chemical and Biomedical Engineering, Columbia, MO, USA; b Dalton Cardiovascular Research Center, Columbia, MO, USA

## Abstract

Focal ischemic stroke (FIS) is a serious neurological disease. Astrocytes are the predominant glia cell types in the central nervous system (CNS) and undergo transformation to a reactive state after FIS. This process is termed reactive astrogliosis and the activated cells are called reactive astrocytes. Reactive astrogliosis is a hallmark of FIS and contributes to tissue remodeling and functional recovery following FIS. Glial cell-derived neurotrophic factor (GDNF) was originally isolated from a rat glioma cell-line supernatant and has been discovered to be a potent survival neurotrophic factor for dopaminergic, noradrenergic and motor neurons. In our previous study, we found that reactive astrocytes upregulate GDNF expression and secretion after ischemia, and deletion of GDNF in astrocyte leads to increased neuronal death and brain damage after photothrombosis (PT)-induced FIS. Moreover, GDNF deletion inhibits reactive astrogliosis and reduces proliferation of reactive astrocytes, suggesting astrocyte derived GDNF can promote neural survival. Here, we tested whether overexpression of GDNF in astrocytes had neuronal and brain protective effect after ischemia. First, we constructed AAV vectors with astrocyte-promotor to overexpress GDNF in astrocytes after injection. We observed that astrocyte specific GDNF overexpression decreases brain infarction and promotes motor function after PT. We also found that overexpression of GDNF increased reactive astrogliosis and reduced oxidative stress in the peri-infarct region (PIR) after PT. Second, to test whether it is extracellular GDNF released from astrocytes exert its neuronal and brain protective effect after ischemia, we transfected cultured astrocytes with DNA plasmid encoding GDNF. Using ELISA, we found GDNF concentration was significantly elevated in culture medium after oxygen-glucose deprivation (OGD), and GDNF concentration was further elevated in culture medium after DNA transfection. Moreover, astrocyte condition medium (ACM), collected from OGD subjected astrocyte culture, significantly increased neuronal survival and viability after OGD, but GDNF neutralizing antibody suppressed this beneficial effect. Lastly, we found that astrocyte derived GDNF triggered the activation of the Ret receptor in cultured neurons and suppresses caspase-dependent cell apoptosis after OGD. Collectively, our results indicate that reactive astrocytes can release GDNF after ischemia and protect neurons and brain through non-cell autonomous mechanism. Our study underscores the importance of reactive astrocytes in neuronal and brain protection in ischemia and suggests that promoting endogenous neurotrophic factor release from reactive astrocytes might be a potential approach in stroke therapy.

# Abstract: P02.31 – The Sphingosine-1-Phosphate Receptor 1 Antagonist Ponesimod Reduces Neuroinflammation via Microglial Aβ Clearance

**Published:** 2025-09-05

**Authors:** Zhihui Zhu, Xiaojia Ren, Liping Zhang, Priyanka Tripathi, Zainuddin Quadri, Stefka D. Spassieva, Erhard Bieberich

**Affiliations:** a Department of Physiology, College of Medicine, University of Kentucky, Lexington, KY, USA; b Veterans Affairs Medical Center, Lexington, KY, USA

## Abstract

**Background:**

Previously, we showed that the sphingosine-1-phosphate (S1P) transporter spinster 2 (Spns2) mediates activation of microglia in response to amyloid peptide Aβ. Here, we investigated if Ponesimod, a functional antagonist specific for the S1P receptor 1 (S1PR1), could prevent Aβ-induced activation of microglia and Alzheimer’s disease (AD) pathology.

**Methods:**

We used primary cultures of mixed glia and pure microglia as well as the 5XFAD mouse model to determine the effect of Aβ and Ponesimod on glial activation, Aβ phagocytosis, cytokine levels and activation of proinflammatory cell signaling pathways, and AD pathology and cognitive performance.

**Results:**

In primary cell cultures of astrocytes and microglia, oligomeric Aβ42 increased the levels of TLR4 and S1PR1 and induced the formation of a complex between the two receptors as shown by proximity ligation assays (PLAs) and co-immunoprecipitation experiments. Ponesimod prevented the Aβ-induced increase of TLR4 and S1PR1 as well as reduced the number of PLA signals and the amount of (co-) immunoprecipitated TLR4 and S1PR1. Aβ42 activated the pro-inflammatory signaling pathways Stat1 and p38 MAPK, which was prevented by Ponesimod, while Stat6 was activated by Ponesimod. Consistent with Stat6 activation, Ponesimod increased phagocytosis of Aβ42 in microglia in vitro. In comparison, FTY720, a functional antagonist of several S1P receptors, did not enhance phagocytosis of Aβ42. These results were confirmed by RNA-seq analysis showing that in contrast to FTY 720, Ponesimod upregulates genes encoding the phagosome and phagocytic proteins in microglia. In 5XFAD mice, Ponesimod decreased TNF-α and CXCL10, two pro-inflammatory cytokines activating TLR4 and Stat1, while it increased the level of IL-33, an anti-inflammatory cytokine that activates Stat6 and induces Aβ phagocytosis in microglia. Consistent with reduced neuroinflammation and increased phagocytosis, Ponesimod decreased the number of IBA-1 (+) microglia and GFAP (+) astrocytes, and the size and number of amyloid plaques, while it improved spatial memory measured by a Y-maze test.

**Conclusion:**

Targeting S1PR1 with Ponesimod is a promising therapeutic approach to reprogram microglia, reduce neuroinflammation, and increase Aβ clearance in AD

# Abstract: P02.32 – Effects of Centella Asiatica Water Extract on Cerebrovascular Function in Mice

**Published:** 2025-09-05

**Authors:** Benjamin Zimmerman, Amala Soumyanath, Nora Gray, Martin Pike, Anusha Mishra

**Affiliations:** a National University of Natural Medicine, Portland, OR, USA; b Oregon Health and Science University, Portland, OR, USA

## Abstract

Scientific evidence has supported the reputed ability of the botanical Centella asiatica to promote healthy cognitive function in aging. However, the biological mechanisms mediating these beneficial effects are not well understood. Elucidating these mechanisms is critical to optimizing products derived from traditional botanicals and assessing appropriate biomarkers of target engagement and biological outcome measures in clinical trials. One potential mechanism underlying the benefit of C. asiatica is through acute or longer-term effects on the tone of cerebral small vessels, which could support cognition by increasing cerebral blood flow. We tested this mechanism by investigating the effects of C. asiatica on cerebrovascular function in mice. We treated 2-month old or 17-month old C57BL/6 mice with an aqueous extract of C. asiatica (CAW; 10 mg/mL in drinking water) for five weeks and then assessed cerebrovascular function in vivo using arterial spin labeling MRI to measure resting brain perfusion and cerebrovascular reactivity to a hypercapnic challenge. Control mice received drinking water with no additives. We found that CAW treatment did not change resting perfusion but resulted in a significant increase in hypercapnia-evoked cerebrovascular reactivity compared to control animals. In the same mice, we then assessed the vasomotor response of capillaries to an oxidative stress challenge ex vivo using brain slice preparations. We applied 1 mM H_2_O_2_ and assessed the resultant change in diameter of capillaries. We found both age and treatment effects in the capillary responses to H_2_O_2_, with older mice showing larger constrictions than young mice, and a stronger loss of late-stage constriction in controls compared to CAW treated mice. In separate experiments, we also examined whether CAW exhibits acute vasodilatory effects ex vivo. However, capillaries pre-constricted with U46619 showed no significant dilation in response to CAW at 50 µg/ml or 100 µg/ml in brain slices. Overall, CAW appears to exert some beneficial effects on cerebrovascular function, which may be mediated through resilience to oxidative stress rather than acute vasodilatory effects on the cerebral microvasculature. However, CAW compounds may require activation by biotransformation in vivo, which would not be detected in ex vivo experiments.

# Abstract: P02.33 – Developing Tools to Study Cholesterol Metabolism in Microglia During Demyelination

**Published:** 2025-09-05

**Authors:** Matthew Zupan, Meredith Hartley

**Affiliations:** a University of Kansas, Lawrence, KS, USA

## Abstract

Microglia are innate immune system macrophages that play a crucial role in central nervous system remyelination, in part by clearing myelin debris following demyelination. Myelin debris clearance is an essential process that allows for the recruitment of oligodendrocyte progenitor cells to the lesion site to initiate myelin repair. Thyroid hormone is known to induce phagocytosis in microglia. Thyroid hormone agonists can promote remyelination in mouse models of myelin damage by promoting differentiation of oligodendrocyte precursor cells, but these drugs may also be acting on microglia to help promote remyelination. Our lab is interested in further exploring the role of thyroid hormone action in microglia and elucidating a novel mechanism for how thyroid hormone agonists promote remyelination. We have preliminary data showing that Sob-AM2, a CNS-penetrating thyroid hormone agonist, increases the phagocytic activity of primary microglia. In addition, we have data from animal models showing that Sob-AM2 treatment can reduce the accumulation of cholesterol esters in microglia during demyelination. To follow up on these results, we are currently developing a mass spectrometry assay that will allow us to study cholesterol metabolism in microglia. We will introduce isotopically labeled cholesterol and cholesterol ester to primary microglia and use mass spectrometry to analyze the resulting metabolites produced by the cells under different experimental conditions. We expect that microglia treated with thyroid hormone agonists in the presence of myelin should activate phagocytic pathways in microglia, therefore stimulating cholesterol metabolism and decreasing the accumulation of cholesterol esters. We are also studying Sob-AM2 action in microglia in a mouse model of demyelination in which the oligodendrocyte progenitor cells are not able to remyelinate. This will allow us to isolate the effect of Sob-AM2 on microglia from the effects of Sob-AM2 on oligodendrocyte progenitor cell differentiation. We expect that Sob-AM2 treatment will trigger microglia towards an anti-inflammatory, phagocytic, restorative phenotype and improve the neurological clinical signs of these mice, even in the absence of remyelination. Overall, we hope to better understand the role of thyroid hormone action on microglia to enable the development of better treatments for demyelinating diseases.

# Abstract: P02.34 – Kindling Epileptogenesis Induces Broad Alterations in the Hippocampal Transcriptome of Mice Lacking TIA1 Cytotoxic Granule Associated RNA Binding Protein

**Published:** 2025-09-05

**Authors:** Antoaneta Andonova, Yifan Gong, Yasir Ahmed-Braimah, Sandra Hewett, James Hewett

**Affiliations:** a Syracuse University, Syracuse, NY, USA; b LinkedIn, Sunnyvale, CA, USA

## Abstract

TIA-1 is an RNA binding protein that is best known as a regulator of alternative RNA slicing and as a posttranscriptional modulator of gene expression. In the latter case, it silences translation of target mRNAs primarily by binding to adenine/uridine-rich sequences in the 3’ untranslated region. Estimates suggest that it targets several thousand transcripts. TIA-1 is a major component of stress granules and as such, suppresses expression of proinflammatory genes, including tumor necrosis factor alpha and cyclooxygenase-2. This may contribute to its ability to control inflammatory arthritis and neuroinflammatory processes associated with chronic neurological conditions, like tauopathies. In addition to its role in inflammation, recent results suggest that TIA-1 may suppress brain hyperexcitability associated with the process of epileptogenesis. In this regard, mice lacking TIA-1 (KO) exhibited 2 to 3-fold higher incidence of acquired epilepsy in the kindling model of epileptogenesis compared to wildtype (WT) littermate controls. How TIA-1 modulates kindling is not clear, particularly since inflammation is not a major aspect of kindling progression. To begin to answer this question, this study analyzed transcriptional changes in the hippocampus during the kindling treatment paradigm. KO or WT mice were treated daily with the convulsant agent, pentylenetetrazole (PTZ), at a dose that did not elicit convulsive seizures initially. Cohorts of mice from each genotype were treated in parallel with saline. Hippocampal tissue samples were harvested from PTZ- and saline-treated mice prior to the occurrence of convulsive seizures in the former and transcriptional changes were assessed by next generation RNAseq analysis. Expression of 1465 genes were modified in KO mice by PTZ treatment (FDR <0.05). By comparison, only 206 genes were altered in PTZ-treated wildtype mice. Moreover, expression of 1469 genes were changed in KO mice treated with saline (FDR <0.05), suggesting that TIA-1 indirectly or directly plays an important role in basal gene expression in the hippocampal formation. Interestingly, of the 305 genes with background changes in PTZ-treated KO mice (FDR <0.02), 235 returned to wildtype levels. This suggests that the kindling paradigm caused a majority of these genes to return to the wildtype homeostatic set point. Gene Ontology enrichment analysis identified several pathways that were modified in PTZ-treated KO mice. These include regulation of dendritic morphogenesis, axonogenesis, regulation of synaptic plasticity, regulation of neurogenesis, regulation of chemical synaptic transmission, potassium ion transport, cellular response to calcium ion, protein folding, and negative regulation of neuron apoptotic process. Expression of a large number of transcriptional regulators were altered as well. These changes only partially overlapped with WT mice treated with PTZ. Notably lacking in these mice were pathways related dendrite morphogenesis, potassium ion transport, cellular response to calcium ion, protein folding, and negative regulation of neuron apoptosis. This suggests that changes in these pathways may contribute to the enhanced kindling acquisition in the KO mice. This information could be useful for the identification of endogenous pathways that serve to suppress epileptogenesis in humans.

# Abstract: P02.35 – Metabolic Derangement Resulting From Diet-Induced Obesity Is Dependent on Estrogen in a Mouse Model of Menopause

**Published:** 2025-09-05

**Authors:** Steven Barger, Nicole Schartz, Jin Hee Sung

**Affiliations:** a Central Arkansas Veterans Healthcare System, University of Arkansas for Medical Sciences, Little Rock, AR, USA; b Little Rock, AR, USA

## Abstract

Type-2 diabetes (T2D) is often accompanied by cardiovascular pathologies such as hypertension, atherosclerosis, and thrombosis, all of which promote vascular dementia (VaD), the second-leading cause of severe cognitive impairment in older Americans. Speculation has arisen that T2D may also elevate the risk for Alzheimer’s disease (AD), but stringent epidemiological analysis suggests that the dementia risk from T2D is restricted to VaD. Nevertheless, both disorders have a special relationship to estrogen, as women experience both VaD and AD at higher rates after menopause. Estrogen has been documented to provide protection against metabolic syndrome, especially in response to diet-induced obesity. Feeding mice a high-fat, high-sucrose (“western”) diet produces a metabolic syndrome, including memory deficits which draw relevance to VaD; in C57BL/6, these effects are limited to males. The protection of female mice from such pathology likely reflects the absence of menopause in this species. We have determined that ovariectomy renders female mice vulnerable to metabolic and cognitive disturbances in a model of AD, prevented by replenishing 17ꞵ-estradiol. We have also instituted a diet-induced obesity protocol after similar depletion of ovarian hormones. As in the AD model, 17ꞵ-estradiol was added back to one cohort of the affected mice. The hormone-depleted mice exhibited glucose intolerance similar to that of males, an effect prevented by 17ꞵ-estradiol. Cognitive performance appears to correlate with glucose tolerance. This paradigm thus strengthens the links between estrogen, diet, glucose regulation, and cognition in a model that approximates dementia associated with metabolic disorders as distinguished from AD, per se.

# Abstract: P02.36 – A Novel Mechanism for Brain Prostanoid Regulation Through Oxygen Availability Under Ischemia

**Published:** 2025-09-05

**Authors:** Derek Besch, Drew Seeger, Brennon Schofield, Svetlana Golovko, Peddanna Kotha, Meredith Parmer, Mikhail Golovko

**Affiliations:** a University of North Dakota, Grand Forks, ND, USA

## Abstract

We and others have previously demonstrated a dramatic and rapid prostanoid (PG) increase upon brain ischemia. This increase in PG is traditionally explained through the activation of phospholipases, releasing esterified arachidonic acid (20:4n6) as a substrate for cyclooxygenases (COX). However, the availability of oxygen as a necessary substrate for this reaction was not considered as a regulatory factor under these conditions. To address this mechanism of PG regulation via oxygen availability, we measured mouse brain oxygen concentrations after global ischemic onset and used head-focused microwave irradiation (MW) to inactivate enzymes in situ at different time points after ischemic onset for PG and free 20:4n6 analysis. O_2_ concentrations were measured in the mouse cortex using a microsensor probe. For PG and free 20:4n6 analysis, mice were anesthetized with isoflurane and euthanized by cervical dislocation preceding craniotomy (nonMW group), or exposure to MW before craniotomy (MW group) at 0.5, 2, and 10 min postmortem. Frozen brain tissue powder underwent homogenization and liquid/liquid extraction for PG analysis, and PG were quantified using UPLC-MS/MS against stable isotope-labeled internal standards. For the ischemic brain, oxygen half-life was 5.32 ± 0.45 s and dropped below detectable levels within 12 s of ischemia onset, while there were no significant free 20:4n6 or PG changes at 30 s ischemia in MW-fixed tissue. Furthermore, PG were not significantly increased at 2 and 10 min after ischemia onset compared to basal levels, while free 20:4n6 was increased ∼50 and ∼100 fold, respectively. However, there was a ∼30 fold increase in PG when ischemia was followed by craniotomy of non-MW tissue, exposing metabolically active tissues to atmospheric oxygen. Moreover, craniotomy of non-MW tissue performed in anoxic conditions did not result in PG induction, while there was significant induction of PG when these brains were further exposed to atmospheric oxygen. These data indicate, for the first time, that the availability of oxygen is a crucial regulatory factor for PG induction during ischemia. Further studies are required to investigate the physiological role of eicosanoid regulation through tissue oxygen concentration.

# Abstract: P02.37 – Fractalkine Overexpression via rAAVs Differentially Regulate Microglial Activation and Vascular Damage in the Diabetic Retina

**Published:** 2025-09-05

**Authors:** Derek Rodriguez, Kaira Church, Difernando Vanegas, Chelsea Smith, Sandra Cardona, Isabel Muzzio, Kevin Nash, Astrid Cardona

**Affiliations:** a Department of Molecular Microbiology and Immunology, The University of Texas at San Antonio, San Antonio, TX, USA; b Department of Psychological and Brain Sciences, University of Iowa, Iowa, IA, USA; c Department of Molecular Pharmacology and Physiology, University of South Florida, Tampa, FL, USA

## Abstract

Diabetic retinopathy (DR) is the leading cause of blindness worldwide due to neuronal loss, microgliosis, and vascular damage. Inflammation caused by microglia exacerbates retinal damage, resulting in glial and neuronal cell dysfunction. Microglial-neuronal crosstalk mediated by CX3CR1/FKN signaling regulates microglial responses. CX3CR1 polymorphic variants present in 25% of the population decrease FKN binding affinity to CX3CR1, and their role in microglia function in the retina during diabetes is unknown. FKN exists as a membrane-bound (mFKN) adhesion molecule. When cleaved by proteases, the soluble (sFKN) form exerts chemoattractant properties. Previous studies show that disruption of CX3CR1/FKN signaling worsens retinal pathology and can be rescued by administration of recombinant FKN. Mechanisms by which mFKN and sFKN regulate retinal function are still unknown. We hypothesize that during diabetes, the over-expression of sFKN using recombinant adeno-associated viruses (rAAVs) will prevent vascular and neuronal damage, and improve visual function. Using neuron-specific recombinant adeno-associated viruses (rAAVs), we overexpressed soluble (sFKN) or membrane-bound (mFKN) FKN using intra-vitreal delivery before and at the onset of diabetes. This study highlights the neuroprotective role of rAAV-sFKN, reducing microglial activation, vascular tortuosity, fibrin(ogen) deposition, and astrogliosis and supporting the maintenance of the GJ connexin-43 (Cx43) and TJ zonula occludens-1 (ZO-1) molecules. The results also show that microglia–vascular interactions influence the vascular width upon administration of rAAV-sFKN and rAAV-mFKN. Administration of rAAV-sFKN improved visual function without affecting peripheral immune responses. These findings suggest that overexpression of rAAV-sFKN can mitigate vascular abnormalities by promoting glia–neural signaling. sFKN gene therapy is a potentially promising and safe translational approach to reverse tissue damage in DR.

# Abstract: P02.38 – TMEM106B Core Deposition Associates With TDP-43 Pathology and Is Increased in Risk SNP Carriers for Frontotemporal Dementia

**Published:** 2025-09-05

**Authors:** Casey Cook

**Affiliations:** a Mayo Clinic, Jacksonville, FL, USA

## Abstract

Genetic variation at the transmembrane protein 106B gene (TMEM106B) has been linked to risk of frontotemporal lobar degeneration with TDP-43 inclusions (FTLD-TDP) through an unknown mechanism. We found that presence of the TMEM106B rs3173615 protective genotype was associated with longer survival after symptom onset in a postmortem FTLD-TDP cohort, suggesting a slower disease course. The seminal discovery that filaments derived from TMEM106B is a common feature in aging and, across a range of neurodegenerative disorders, suggests that genetic variants in TMEM106B could modulate disease risk and progression through modulating TMEM106B aggregation. To explore this possibility and assess the pathological relevance of TMEM106B accumulation, we generated a new antibody targeting the TMEM106B filament core sequence. Analysis of postmortem samples revealed that the TMEM106B rs3173615 risk allele was associated with higher TMEM106B core accumulation in patients with FTLD-TDP. In contrast, minimal TMEM106B core deposition was detected in carriers of the protective allele. Although the abundance of monomeric full-length TMEM106B was unchanged, carriers of the protective genotype exhibited an increase in dimeric full-length TMEM106B. Increased TMEM106B core deposition was also associated with enhanced TDP-43 dysfunction, and interactome data suggested a role for TMEM106B core filaments in impaired RNA transport, local translation, and endolysosomal function in FTLD-TDP. Overall, these findings suggest that prevention of TMEM106B core accumulation is central to the mechanism by which the TMEM106B protective haplotype reduces disease risk and slows progression.

# Abstract: P02.39 – Inferring Axon Diameters in the Mouse Corpus Callosum Using Diffusion MRI: Comparison of ROI-Based and Voxel-Based Analysis

**Published:** 2025-09-05

**Authors:** Emma Friesen, Madison Chisholm, Morgan Mercredi, Sheryl Herrera, Melanie Martin

**Affiliations:** a Department of Chemistry, University of Winnipeg, Winnipeg, Canada; b Department of Biology, University of Winnipeg, Winnipeg, Canada; c Department of Physics, University of Winnipeg, Winnipeg, Canada

## Abstract

Alterations in myelinated axon diameters are thought to predate clinical presentations of various neurodegenerative disorders. In vivo measurements of axon diameters could lead to an early biomarker. Magnetic Resonance Imaging (MRI) Temporal Diffusion Spectroscopy (TDS) has been previously used to infer larger axon diameters. Smaller axon diameters like the majority of axons constituting cortical connections, can be targeted using the higher frequency Oscillating Gradient Spin Echo (OGSE) pulse sequences. The current project aims to compare two common methods of analysis: Region-of-Interest (ROI)-based and Voxel-Based Analysis (VBA) to infer axon diameters in mice using TDS with OGSE to determine if the additional spatial information from VBA adds more information about axon diameter distributions above the added uncertainties. A 12-week-old male Clostridioides difficile infected (CDI) mouse was perfused with heparin in 0.1 M PBS and 4% PFA. The brain (in skull) was soaked in 4% PFA in a 15 mL sterile centrifuge tube for 24 h and then transferred to 0.1 M PBS solution for 24 h to remove any remaining fixative. All experiments were approved by the Universities’ Animal Care Committee. The brain (in skull) was placed inside a Teflon sample holder containing FOMBLIN and imaged using a 7 Tesla 21 cm bore Bruker BioSpin NMR System with ParaVision 5.0 with a 35 mm RF coil within a Bruker BG6 gradient set. Two 20 ms apodised cosine gradient pulses each had *n* = 1 to 6 cycles and were separated by 24.25 ms. Gradient strengths of 15% and 30% of the maximum (1.01 T/m) for *n* = 1 were used, increasing with n to maintain constant b-values until maximum gradient power was obtained, at which point 95% of the maximum gradient was used instead. Imaging parameters include TR =1250 ms, TE =50 ms, 2 cm^2^ FOV, matrix 128 × 128, 256 µm in plane resolution, acquisition time of 32 mins (scans =168, 3.73 days), 3 mm image slice positioned perpendicular to the corpus callosum. Prior to fitting, collected images were manually registered to the b = 0 diffusion image using custom-built MATLAB^®^ code. ROIs were drawn around the corpus callosum and within the noise. Signals were extracted and fitted to the ActiveAx model, either using ROI-based analysis for a single AxD for the entire ROI or VBA for a single AxD for each voxel within the ROI, along with 95% confidence bounds. ROI-based analysis mean inferred axon diameter was found to be 5.2 ± 0.8 µm. VBA inferred axon diameters ranging between 2.1 ± 0.3 µm to 10.8 ± 1.1 µm, with mode of 2.4 µm, median of 4.7 µm, and mean of 4.8 ± 1.4 µm. The current project was successful at measuring axon diameters using both ROI-based and VBA methods. VBA has a significantly larger computational load compared to the faster ROI-based analysis; and increased confidence intervals, however, this method provides information regarding variation in the sample which is overlooked in ROI-based analysis. Further research is required to determine the required confidence interval for distinguishing healthy and diseased tissue.

# Abstract: P02.40 – Neuroactive Effects of Ashwagandha: From Ayurvedic Medicine to Cellular Bioactivity and Mass Spectrometry

**Published:** 2025-09-05

**Authors:** Miss. Emily Georges, Luke Marney, Jaewoo Choi, Liping Yang, Md Alam Nure, Arpa Ebrahimi, Armando Alcazar Magana, Amala Soumyanath, Carmen Wong, Bailey Keefe, Jan F. Stevens, Claudia S. Maier

**Affiliations:** a Department of Chemistry, Oregon State University, Corvallis, OR, USA; b Center for Healthy Aging Research, Oregon State University, Corvallis, OR, USA; c BENFRA Botanical Dietary Supplements Research Center, Oregon Health and Science University, Portland, OR, USA; d Linus Pauling Institute, Oregon State University, Corvallis, OR, USA; e Department of Neurology, Oregon Health and Science University, Portland, OR, USA; f College of Health, Oregon State University, Corvallis, OR, USA; g Department of Pharmaceutical Sciences, Oregon State University, Corvallis, OR, USA

## Abstract

**Introduction and objective:**

Ashwagandha (Withania somnifera, WS) is an herb that has been used traditionally in Ayurvedic medicine. Traditional Ayurvedic use of ashwagandha is seen in Rasayana treatment for mental support where it is often prepared in multi-herb mixtures in honey and ghee. Understanding the cultural heritage of Ayurveda is important for the ethical cohesion of Rasayana with current scientific research. Rasayana focuses on enhancing life mentally, physically, and spiritually. Rasayana and the use of ashwagandha restores imbalance in the mind that is believed to be the cause of mental degeneration associated with aging. How can Ayurvedic medicine be examined through a cultural lens to better understand the treatment principles of balance and wellness, and are rasayana principles of balancing of the doshas used to describe and treat neurodegeneration related with metabolites found in Withania somnifera? There are many bioactive molecular families of interest in ashwagandha, including, but not limited to, tropane alkaloids and steroidal lactones. Here we combine standardization of the metabolites found in ashwagandha extract using Liquid Chromatography Time-of-Flight Mass spectrometry (LC-TOF-MS) with in vitro bioassays.

**Methods:**

Using an ethanol-water extraction method, ashwagandha root was extracted by equal volumes of ethanol and water, vortexed and sonicated, and then centrifuged. Quantification of targeted phytochemicals in the ethanolic-water extract of ashwagandha was achieved by LC-TOF-MS on a Waters Synapt G2 mass spectrometer. Eight commercially available standards were used for quantification. Additional chemical constituents are explored using ProgenesisQI and GNPS (Global Natural Product Social Networking). NRF2/ARE induction was examined using a HepG2 cell line with a luciferase reporter. Cell viability was measured using CellTiter Glo Ⓡ.

**Results:**

Quantification of known withanolides showed the largest abundance in the extract seen for withaferin A, withanolide B, and 12-deoxywithastramonolide. ProgenesisQI processing and filtering resulted in 500 deisotoped and adduct combined molecular features and GNPS resulted in a network graph of mass spectral similarity grouping common features together. At concentrations of 0.5–500 µg/mL in cell media, the ashwagandha extract demonstrated dose dependent NRF2/ARE induction in the HepG2 cell line. In contrast to other WS extracts and pure withanolides, there was no negative effect on cellular viability.

**Conclusions:**

Preliminary results suggest that there is some activity of compounds present in ashwagandha root ethanol-water to stimulate an antioxidant response in a hepatic cellular assay. Further research needs to corroborate whether this antioxidant pathway is key to ashwagandha’s use in neurodegeneration. Antioxidant action, by possibly reducing oxidative stress on body cells, reflects one of Rasayana’s purposes as described by Charaka, “achieving finest quality of body tissue.”

# Abstract: P02.41 – A Novel Nanoparticle-Mediated Delivery of Estrogen to Protect Neurons and Improve Functional Recovery in Spinal Cord Injury

**Published:** 2025-09-05

**Authors:** Azizul Haque, Vandana Zaman, Denise Matzelle, Alexey Vertegel, Abhay Varma, Donald Shields, Naren Banik

**Affiliations:** a Medical University of South Carolina, Charleston, SC, USA; b Ralph Johnson VA Medical Center, Charleston, SC, USA; c Clemson University, Clemson, SC, USA

## Abstract

Spinal cord injury (SCI) is a complex debilitating condition leading to permanent life-long neurological deficits. In addition, individuals with SCI experience neurogenic muscle loss due to immobility. Amelioration of neurological deficits and prevention of skeletal muscle loss are intricately related to recovery of function following SCI. While some progress has been made toward understanding the mechanisms that underlie muscle loss, molecular mechanisms resulting neuronal impairment and skeletal muscle loss resulting from SCI, remain incompletely understood. Our laboratory is among the first to show steroid hormone estrogen (E2) driven neuroprotection in experimental SCI in rats, suggesting E2 warrants clinical evaluation in neurotrauma patients. The beneficial effect in SCI was found at the low dose of 10 μg/kg E2, but the dose remains non-physiologic and thereby poses a safety concern for clinical use. The emergence of smart drug delivery techniques, such as E2-embedded nanoparticles at the site of injury, may allow for increased drug safety and improved efficacy. Studies here suggest that a single administration of a combined fast-release formulation (FNP-E2) and slow-release formulation (SNP-E2) to the contused spinal cord attenuates inflammatory cytokines/chemokines, gliosis, glial scarring, and neurogenic muscle loss. The focal delivery of E2 promotes microglial and astroglial differentiation where subpopulations of anti-inflammatory microglia and astrocytes could inhibit inflammation, axonal damage, and neuronal loss. NP-mediated delivery of E2 gel patch therapy also reduced inflammation- or insult-induced muscle RING finger 1 (MuRF1) and muscle atrophy F-box (MAFbx) proteins in vitro in myoblast cells and in vivo in rats following mild to moderate SCI (40 g/cm injury) in rats. Thus, using a novel combined FNP-E2 and SNP-E2, may allow for acute inflammation to be halted by FNP-E2 and modulated by SNP-E2 thereafter.

# Abstract: P02.42 – PMP22 Null Mutant Mice Exhibit Morphological and Functional Deficits in the Respiratory System

**Published:** 2025-09-05

**Authors:** Evan Jones, Victoria Defilippi, Thomas Gould, Valerie Handlos, Nicole Procacci, Lucia Notterpek

**Affiliations:** a Reno – School of Medicine, University of Nevada, Reno, NV, USA

## Abstract

Haploinsufficiency of peripheral myelin protein 22 (PMP22) is associated with Hereditary Neuropathy with Pressure Palsy (HNPP), an under-diagnosed, compression-induced neuropathy. Neuropathic patients with PMP22 null mutations are rare and present with an early onset, complex clinical phenotype, suggesting a critical role for PMP22 in neural development beyond myelination. As PMP22-deficient mice die around 1-mo of age, possibly due to respiratory dysfunction, in the current study we investigated the role of PMP22 in postnatal neural development by focusing on the respiratory system, including the diaphragm, the phrenic nerve, and the cervical 3–6 region of the spinal cord. Genotyped littermates of wild type (Wt) and PMP22-knock out (KO) mice were collected at 3-week of age, and the diaphragm, the phrenic nerve, and the cervical 3–6 region of the spinal cord were processed for immunohistochemical and biochemical studies. Cryosections of the diaphragm tissue revealed a deficit in the number of innervated nicotinic acetylcholine receptor (AChR) clusters in the motor endplate band. Additionally, the morphology of AChR clusters in the PMP22-KO diaphragms were atypical and exhibited less perforations than their Wt littermates. Immunolabeling of diaphragm muscle tissue with myofiber-specific markers revealed significant atrophy of myofiber cross-sectional areas (CSA), and this atypical phenotype was consistent across all myofiber types (type 1, type 2a, type 2b/x). We also identified an increase in the number of type 1 myofibers and a concurrent decrease in both 2a and 2b/x myofibers in the diaphragms of PMP22-KO mice. Western blots of diaphragm lysates from Wt and PMP22-KO mice showed an increase in protein markers of atrophy, consistent with the fiber typing data. When semithin plastic sections of the phrenic nerves were processed for morphometrics, pronounced decrease in nerve CSAs occupied by axon fibers was observed, along with reduction in the percentage of myelinated fibers and fiber diameter. The spinal cord of affected mice displayed astrogliosis in the grey matter near the ventral horns, where phrenic motor neurons are located. Given the pronounced morphological alterations at the neuromuscular junction (NMJ) in PMP22-KO mice, we investigated the response of Wt and PMP22-KO diaphragms to 30-second trains of high-frequency stimulation (40 Hz) of the phrenic nerve, in the presence of the muscle-specific myosin blocker BHC. PMP22-KOs exhibited significantly fewer successfully transmitted action potentials. Together, these studies support a role for PMP22 in early neural postnatal development, including peripheral innervation of the diaphragm muscle, which may contribute to the impairment of neuromuscular transmission.

# Abstract: P02.43 – The Natural Compounds Oridonin and Shikonin Exert Beneficial Effects on Reactive Microglia, Independent of Their Inflammasome Inhibitory Activity

**Published:** 2025-09-05

**Authors:** Andis Klegeris, Ms Bridget Greuel, Dylan Da Silva, Ms Victoria Robert-Gostlin

**Affiliations:** a University of British Columbia Okanagan, Kelowna, Canada

## Abstract

Alzheimer’s disease (AD) is the sixth leading cause of death worldwide and is characterized by chronic neuroinflammation. Microglia, the brain immunocytes that normally support and protect neurons, can become adversely activated, contributing to the onset and progression of neuroinflammation – a driving mechanism in AD and a wide spectrum of other neurodegenerative diseases. In their reactive form, microglia secrete a mixture of potentially neurotoxic molecules and exhibit defective phagocytic activity. Correcting these abnormal functions of microglia is one potential therapeutic strategy to consider in the search for effective treatments of neurodegenerative diseases. Nucleotide-binding domain leucine-rich repeat and pyrin domain-containing receptor (NLRP) 3 inflammasomes are intracellular protein complexes implicated in adverse microglial activation. Their inhibitors, such as the natural compounds oridonin and shikonin, have been shown to reduce the secretion of inflammatory mediators by microglia. We hypothesized that some of the beneficial effects of oridonin and shikonin on microglia are independent of their suppression of inflammasome activity. We tested this hypothesis using an in vitro cell culture model system, where BV-2 murine microglia were stimulated with bacterial lipopolysaccharide (LPS) only, which did not induce the assembly of the NLRP3 inflammasome and the resulting production of interleukin 1β. Under these experimental conditions, both oridonin and shikonin reduced NO secretion by LPS-stimulated BV-2 microglia and diminished their overall cytotoxicity towards HT-22 murine neuronal cells. Additionally, both oridonin and shikonin upregulated the phagocytosis of latex beads by BV-2 murine microglia. Notably, only oridonin inhibited the secretion of tumor necrosis factor by stimulated BV-2 microglia, while shikonin only significantly suppressed the respiratory burst response of human microglia-like cells. The latter assay utilized dimethyl sulfoxide-differentiated HL-60 human monocytic cells to model the release of excess reactive oxygen species by immune-stimulated microglia. These cells were first primed by LPS to induce their reactive state and then stimulated with N-formylmethionine-leucyl-phenylalanine to trigger the respiratory burst response characterized by the production of high levels of reactive oxygen species in a nicotinamide adenine dinucleotide phosphate (NADPH) oxidase-dependent manner. Our data confirm that oridonin and shikonin display inflammasome-independent beneficial effects on reactive microglia by reducing their release of potentially harmful molecules and their overall neurotoxicity while upregulating microglial phagocytic activity. The observed discrepancy in the suppressive actions of oridonin and shikonin on microglia may indicate that these natural compounds have different molecular targets in this cell type, but this hypothesis will require additional investigation. Overall, our results suggest that oridonin and shikonin should be further investigated as pharmacological agents capable of correcting dysfunctional microglia, supporting their potential use as therapeutic agents for targeting neuroinflammation in AD and other neurodegenerative disorders.

# Abstract: P02.44 – N6-Cyclohexyladenosine Is Better Than Meperidine and Buspirone at Suppressing Metabolism During TTM32 but Does Not Improve Outcome Post Cardiac Arrest

**Published:** 2025-09-05

**Authors:** Bernard Laughlin, Hoshi Sugiura, Mackenzie Jenkins, Kelly Drew

**Affiliations:** a University of Alaska Fairbanks, Fairbanks, AK, USA

## Abstract

**Background:**

N6-Clyclohexyladenosine (CHA) is an adenosine A1 receptor agonist (A1AR) that inhibits thermogenesis to induce a hypometabolic state. However, the side effect profile of hypotension and bradycardia may limit use of CHA as a therapeutic. Others have shown that this cardiovascular side effect profile can be prevented by administering 8-p-(sulphophenyl)theophylline (8-SPT), a nonspecific antagonist that does not cross the blood-brain barrier. We show that 8-SPT alone is not sufficient to prevent hypotension during entrance into the hypometabolic state. However, CHA-induced hypotension and bradycardia can be prevented by combined pretreatment with atropine and 8-SPT. We tested the hypothesis that if blood pressure was managed during the transition into a hypometabolic state that prolonged inhibition of metabolism post-cardiac arrest would improve outcome better than standard-of-care anti-shivering medication, meperidine and buspirone.

**Methods:**

Drug combinations were administered to Sprague-Dawley rats in a cross-over design to determine a formulation that best minimized A1AR agonist-mediated bradycardia and hypotension (*n* = 3–5). This formulation was then tested against meperidine and buspirone after cardiac arrest. Rats were randomly assigned to treatment groups and underwent 7 min of asphyxia to induce cardiac arrest followed by up to 3 min of CPR to achieve return of spontaneous circulation (ROSC) (*n* = 6). Treatment commenced 1 or 2 h after ROSC. Cage surface temperature was adjusted to maintain core body temperature at 32 °C for 24 h.

**Results:**

Pretreatment with 8-SPT, 25 mg/kg and atropine 1 mg/kg 15 min before CHA 1 mg/kg preserves MAP and HR baseline values after CHA administration (*p* < 0.05, two-way repeated measure ANOVA). Pretreatment with 8-SPT or atropine alone did not prevent the fall in MAP and HR. Core body temperature and oxygen consumption differed significantly between treatment groups after cardiac arrest (*p* < 0.05, linear mixed effect model). There was no difference in survival rates between groups (*p* > 0.05, log-rank). Rewarming with CHA was identified as a vulnerable period in the CHA-treated group.

**Conclusion:**

CHA-mediated hypotension can be mitigated by pretreatment with atropine and 8-SPT. This combination when administered after cardiac arrest to facilitate cooling followed by continuous infusion of CHA was superior to meperidine and buspirone at lowering metabolic rate but did not improve survival.

# Abstract: P02.45 – The Role of Connexin43 Mediated Coupling in Neuronal Activity

**Published:** 2025-09-05

**Authors:** Owen Leitzel, Carmen Muñoz-Ballester, Samantha Golf, Stefanie Robel

**Affiliations:** a Graduate Biomedical Sciences, University of Alabama at Birmingham, Birmingham, AL, USA; b Cell, Developmental and Integrative Biology, University of Alabama at Birmingham, Birmingham, AL, USA; c Physical Medicine and Rehabilitation, University of Alabama at Birmingham, Birmingham, AL, USA

## Abstract

Astrocytes couple into networks of cells via gap junctions (GJs) composed of connexins. In acquired epilepsy, many studies have shown dysfunction of GJs and dysregulation of Connexin43 (Cx43), one of the connexins that form astrocyte gap junctions. There is conflict in the literature as to how astrocyte coupling affects acquired epilepsy. A lack of astrocyte coupling can promote seizures. Yet, the opposite has also been shown that inhibiting coupling reduces abnormal neuronal activity and seizures. Thus, whether changes in astrocyte coupling in the context of injury promotes or counteracts neuronal hyperexcitability is not resolved. Here, we are investigating the relationship between astrocyte coupling and neuronal activity in the context of traumatic brain injury (TBI). After mild/concussive TBI, Cx43 is heterogeneously regulated with subsets of astrocytes lacking Cx43 showing reduced coupling with neighboring astrocytes. Other astrocyte populations express increased or unchanged Cx43 protein levels. However, because Cx43 also has non-junctional roles and Cx43 location matters, it is unclear whether astrocytes with more Cx43 are adequately coupled after mild TBI and if and how local neuronal activity is affected by changes in astrocyte coupling. This project tests the hypothesis that TBI-induced impaired gap junctional coupling drives neuronal hyperexcitability. We assess gap junction function and neuronal activity after mild TBI by simultaneous intravital 2-photon imaging (stimulated Ca + signals traveling between astrocytes as a readout for coupling) in conjunction with silicon probe electrophysiological recordings. To determine how impaired gap junction function influences neuronal activity, gap junction function is manipulated using AAV-delivered dominant negative Cx43 constructs that modulate only gap junction opening but not non-junctional Cx43 functions.

# Abstract: P02.46 – Distinct Odor and Reward-Evoked GABAergic Neuronal Activity in the Basal Forebrain Influences Odor Perception and Decision-Making

**Published:** 2025-09-05

**Authors:** Elizabeth Moss, Evelyne Tantry, Elaine Le, Katie Brandel-Ankrapp, Benjamin Arenkiel

**Affiliations:** a Oregon Health and Science University, Portland, OR, USA; b Baylor College of Medicine, Houston, TX, USA

## Abstract

Sensory perception relies on the flexible detection and interpretation of stimuli across variable contexts, conditions, and behavioral states. The basal forebrain is a hub for behavioral state regulation, supplying dense cholinergic and GABAergic projections to various brain regions involved in sensory processing. Of GABAergic neurons in the basal forebrain, parvalbumin (PV) and somatostatin (SST) subtypes serve opposing roles regulating behavioral states. To elucidate the role of basal forebrain circuits in sensory-guided behavior, we investigated GABAergic signaling dynamics during odor-guided decision-making. We used fiber photometry to record cell type-specific basal forebrain activity during an odor discrimination task and correlate temporal patterns of PV and SST neuronal activity with olfactory task performance. We found that while both PV-expressing and SST-expressing GABAergic neurons are activated by odors, PV neurons are selectively suppressed by reward whereas SST neurons are activated. Notably, chemogenetic inhibition of BF SST neurons modestly alters decision bias to favor reward-seeking while optogenetic inhibition of BF PV neurons during odor presentations improves discrimination accuracy. These results suggest that the bidirectional activity of GABAergic basal forebrain neuron subtypes distinctly influences perception and decision-making during olfactory guided behavior.

# Abstract: P02.47 – NMNAT2: A Guardian of Axonal Integrity and Cortical Energy Balance in the Brain

**Published:** 2025-09-05

**Authors:** Zhen-Xian Niou, Sen Yang, Jui-Yen Huang, Hui-Chen Lu

**Affiliations:** a Indiana University Bloomington, Bloomington, IN, USA

## Abstract

Nicotinamide mononucleotide adenylyl transferase 2 (NMNAT2) is a neuroprotective enzyme in several preclinical neurodegeneration models. Belonging to the NMNAT family, it serves as an essential NAD synthesizing enzyme and molecular chaperone. NMNAT2, primarily expressed in the brain, is highly prevalent in post-mitotic cortical neurons. While its role in maintaining axonal health has been established, whether it plays a pivotal role in supporting cortical energy homeostasis remains unclear. In this study, we employed a targeted approach to conditionally knockout (cKO) NMNAT2 in cortical glutamatergic neurons, enabling a comprehensive exploration of NMNAT2’s functions in the brain. Our findings unveil that NMNAT2 depletion results in early-onset axonal degeneration and subsequent degenerative behavioral phenotypes. Moreover, the absence of NMNAT2 disrupts the cortical NAD(P)/NAD(P)H ratio and disturbs glycolysis. This dysregulation manifests as a significant increase in the oxidized/reduced glutathione ratio, indicating elevated oxidative stress in absence of neuronal NMNAT2. Furthermore, astrocytes and microglia were activated, suggesting heightened inflammatory responses in the absence of NMNAT2. Notably, ablating Sarm1 function in NMNAT2 cKO mice eliminates the impact of NMNAT2 loss on axonal integrity and brain homeostasis. In summary, our study offers compelling in vivo evidence supporting critical roles for NMNAT2 in the formation and maintenance of CNS neurons, as well as an essential role in maintaining cortical energy homeostasis.

# Abstract: P02.48 – Differentiation State Impacts the Interaction of mTOR and TFEB in Oligodendrocytes

**Published:** 2025-09-05

**Authors:** Davin Packer, Wendy Macklin

**Affiliations:** a University of Colorado Anschutz Medical Campus, Denver, CO, USA

## Abstract

Transcription Factor EB (TFEB) regulates endolysosomal, autophagic, and apoptotic gene expression in many cells. It, in turn, is regulated by the Mammalian target of rapamycin (mTOR) signaling complex 1 (mTORC1), which senses nutrient availability and regulates numerous metabolically demanding cellular tasks including endolysosomal activities; it also regulates the nuclear localization of TFEB, i.e., its transcriptional activity. In oligodendrocytes, the myelinating cells of the central nervous system (CNS), TFEB regulates pro-apoptotic gene expression, and it may impact endosome recycling and lysosomal activities, which are important in myelination. However, despite their clear connection in other cells, mTOR, TFEB, and endolysosomes have not yet been studied as a single system in oligodendroglia. Objectives: (1) to elucidate interactions between mTOR, TFEB, and endolysosomes in oligodendrocytes of the brain and spinal cord and (2) to determine how the mTOR-endolysosomal-TFEB system affects oligodendrocyte survival, differentiation, and myelination. Our studies establish differential activities of mTORC1 in oligodendrocytes in brain versus those in spinal cord. We are investigating whether these differences extend to its regulation of TFEB localization as well. Methods: Primary rat oligodendrocytes from either brain or spinal cord were differentiated and then deprived of amino acids with or without the mTOR inhibitor, Torin. Oligodendrocytes were stained for lysosomes or for TFEB, imaged by confocal microscopy, and quantified using blinded semi-automated processes. Results: As noted by others, TFEB protein expression increased as oligodendrocytes progressed from the progenitor state to an intermediate differentiation state, then decreased in more differentiated cells. Amino acid deprivation alone was insufficient to cause TFEB nuclear translocation. However, in differentiated oligodendrocytes from both the brain and spinal cord, Torin treatment resulted in TFEB nuclear translocation. In immature oligodendrocytes from the brain, Torin treatment did not induce TFEB nuclear translocation. Ongoing experiments will determine the effects of Torin on TFEB localization in immature oligodendrocytes from the spinal cord. Neither amino acid deprivation, nor Torin treatment yielded detectable changes in lysosomal quantity, intensity, or localization. Conclusion: the current results support a role for mTOR in regulating TFEB activity in oligodendrocytes, which increases as cells differentiate. Given that TFEB regulates transcription of pro-apoptotic genes, this may mean that oligodendrocytes are increasingly vulnerable to mTOR perturbations with increasing differentiation. Current experiments will determine the effects of mTOR disruption on TFEB localization in a myelinating co-culture system, and the effects of endolysosomal disruption on oligodendrocyte survival, differentiation and myelination.

# Abstract: P02.49 – Serpina3n/SERPINA3 in LPS-Induced Neonatal Sepsis: Liver-Brain Axis

**Published:** 2025-09-05

**Authors:** Joohyun Park, Meina Zhu, Yan Wang, Fuzheng Guo

**Affiliations:** a Department of Neurology, School of Medicine, UC Davis, Sacramento, NC, USA; b Institute for Pediatric Regenerative Medicine (IPRM), Shriners Hospitals for Children – North California, Sacramento, NC, USA

## Abstract

Neonate sepsis is one of the critical causes of mortality, which ranked third in neonatal death, and morbidity in newborns. Neonatal sepsis causes brain damage by directly infecting the central nervous system and/or indirectly causing inflammation in the encephalitis. Sepsis often ultimately leads to brain injury and cerebral palsy. Liver-Brain axis in neuroinflammation; there is a growing understanding that inflammatory liver disorders are linked to changes in brain development and behavior Conventionally, liver to brain communication mechanisms have been emphasized on the three cytokines TNFα, IL-1β, and IL-6 between the periphery and the brain during inflammation. In this project, we discover serine protease inhibitor clade a member 3n (Serpina3n) as a novel and crucial mediator for liver-to-brain communications. Oligodendrocytes are myelin-forming cells in the central nervous system (CNS). The involvement of oligodendrocytes in neuroinflammation has only recently come under the spotlight. although the role of microglia and astrocytes is well established. Under pathological states, it has become apparent that oligodendrocytes can transition to disease-specific cell states characterized by the release of pro/anti-inflammatory chemokines, cytokines previously thought limited to immune cells, overturning their image as victims with no immunological properties. However, the molecular mechanism under which oligodendrocytes regulates neuroinflammation remains underdefined. Murine Serpina3n and its human orthologue SERPINA3 is an acute phase inflammatory glycoprotein secreted primarily by hepatocytes into the bloodstream and modulating systemic inflammation. SERPINA3 is dysregulated in brain neural cells, cerebrospinal fluid (CSF), and plasma in response to neurological diseases and injuries. Recent data including my preliminary data suggest that oligodendrocytes may be the primary CNS cell population expressing Serpina3n under neurological conditions, and its role in different pathologies remains controversial. My pilot study showed that: a) Serpina3n level is remarkably elevated in the liver and the brain in the acute phase of lipopolysaccharide (LPS)-induced sepsis, b) in the brain, Serpina3n is predominantly expressed in oligodendrocytes, and c) oligodendrocyte RNA-seq data demonstrated that immune-related pathways and lipid metabolism functions are significantly upregulated in LPS-induced septic brain versus control brain. Based on these exciting data, I hypothesize that Serpina3n is a key novel molecular target by which liver and brain communicate during neonatal sepsis and oligodendrocytes regulate neuroinflammation in the brain. I plan to employ cell-specific gene knockout approaches to answer two specific questions: a) if the liver modulates brain neuroinflammation through Serpina3n upon LPS-induced sepsis since LPS per se cannot cross over the blood brain barrier and b) if oligodendrocyte-derived Serpina3n in the brain modulate neuroinflammation and myelination in neonatal sepsis. This project holds fundamental significance for the field as it challenges our current mechanistic understanding of sepsis within the context of the liver-brain axis. It aims to identify Serpina3n/SERPINA3 as a new target in immunomodulatory oligodendroglial cells, exploring both biology and pathology. I believe that exploring my hypothesis is valuable due to its potential translational significance. By examining downstream targets in LPS-induced sepsis using neonatal animal models, it lays the groundwork for future therapeutic strategies.

# Abstract: P02.50 – Diiodothyropropionic Acid Facilitates Oligodendrocyte Differentiation and Myelination to Enhance Neuroprotection and Neurorepair in the Central Nervous System

**Published:** 2025-09-05

**Authors:** Rahimeh Emamnejad, Maurice Pagnin, Kaylene Young, Steven Petratos

**Affiliations:** a Monash University, Melbourne, Australia; b University of Tasmania, Hobart, Australia

## Abstract

In multiple sclerosis (MS), oligodendrocyte (OL) degeneration and subsequent demyelination in the central nervous system (CNS) causes neurological impairment. Monocarboxylate transporter 8 (MCT8) may promote OL maturation and myelination by actively transporting thyroid hormone (TH) into the CNS and thereby facilitating key transcription and metabolomic pathways for myelin biogenesis. Diiodothyropropionic acid (DITPA), a synthetic TH-analog, can be transported into the CNS, independent of MCT8 and may promote OL maturation and myelination. In this study, we investigated the potential of DITPA to limit OL dystrophy and promote remyelination in the context of neuroinflammation, to demonstrate potential therapeutic efficacy for neuro-protection/repair during MS. Using archival human tissue, white matter demyelinated lesions from individuals with secondary progressive MS (SPMS) displayed significantly reduced MCT8 expression levels along with intracellular deiodinase 2 and 3, illustrating a brain hypothyroid state which may drive progressive disease. Furthermore, the metabolomic analysis indicated dysregulated TH signaling due to MCT8-deficiency and abrogated downstream AKT-mTOR-PANK2 signalling pathways. Utilising OLs derived from the NKX2.1-GFP human embryonic stem cell (hESC) reporter line, in-vitro treatment with DITPA outperformed active Triiodothyronine (T3), and MCT8-independent TH-analog, triiodothyroacetic acid (TRIAC), by enhancing OL differentiation and myelination of cocultured retinal ganglion cells during MCT8-knockdown. Preclinical studies in the experimental autoimmune encephalomyelitis (EAE) mouse model of MS demonstrated that DITPA promoted the AKT-mTOR-PAK2 signaling pathway and enhanced neuroprotection and remyelination, when benchmarked against other synthetic TH analogs, TRIAC and LT3. Collectively, these data emphasise that DITPA may signal neuroprotection in the context of neuroinflammation and has potential as a therapeutic intervention for MS.

# Abstract: P02.51 – Delayed Administration of an Angiotensin(II) Type(2) Receptor Agonist for the Treatment of Ischemic Stroke: A Preclinical Trial in Hypertensive Rats

**Published:** 2025-09-05

**Authors:** Mohammed Sayed, Mahmoud Mahmoud Abdelbary, Bindu Pillai, Abdulkarim Alshammari, Asmat Jahan, Susan Fagan, Adviye Ergul

**Affiliations:** a Lincoln Memorial University, Knoxville, TN, USA; b Oregon Health and Science University, Portland, OR, USA; c University of Georgia, Augusta, GA, USA; d University of South Carolina, Charleston, SC, USA

## Abstract

The aim of this translational, randomized, controlled, blinded preclinical trial was to determine the effect of delayed administration of compound 21 (C21) in ischemic stroke in hypertensive rats. Spontaneously hypertensive rats (SHR) were subjected to 60-min middle cerebral artery occlusion (MCAO) or sham surgery. They received C21 (0.12 mg/kg/d) or water (orally) for 8 weeks, with the first dose given at 3 days post-MCAO. Adhesive removal task (ART), Novel Object Recognition (NOR), Passive Avoidance Test (PAT) and Sucrose Preference Test (SPT) were utilized to test sensorimotor and cognitive function. After performing the behavior tests, brains were collected for analyses. Markers of inflammation, cell-death and DNA damage were quantified in the brain lysates. Stroked animals suffered significant sensorimotor deficits that improved over time and persistent cognitive deficits compared to sham animals. Exploration time was reduced due to stroke and there was evidence of depressive-like symptoms at 8 weeks after stroke. Delayed treatment with C21 was not effective in improving the rate of sensorimotor recovery or preventing cognitive deficits.

# Abstract: P02.52 – Sex and Viral Infection-Induced Inflammation as Contributing Factors to Alterations in Cognitive Performance and Microglial Response in Aged C57BL6/J Mice

**Published:** 2025-09-05

**Authors:** Eva Simoncicova, Antonia Landwehr, Makenna Gargus, Katherine Picard, Mohammadparsa Khakpour, Keelin Henderson Pekarik, Maryam Zafarullah, Mariem Mahmood, Eva Tunderman, Sophia Loewen, Haley A. Vecchiarelli, Marie-Ève Tremblay

**Affiliations:** a Division of Medical Sciences, University of Victoria, Victoria, Canada; b Neuroscience Graduate Program, University of Victoria, Victoria, Canada; c Center for Translational Immunology, University Medical Center Utrecht, Utrecht, Netherlands; d Institute of Lifelong Health and Aging, University of Victoria, Victoria, Canada; e Centre for Advanced Materials and Related Technology (CAMTEC), University of Victoria, Victoria, Canada; f Department of Biochemistry and Molecular Biology, University of British Columbia, Vancouver, Canada; g Neurology and Neurosurgery Department, McGill University, Montréal, Canada

## Abstract

Aging presents an increased demand on brain maintenance by glial cells. Associated with a sustained pro-inflammatory status, aging may lead to a loss of functional flexibility of microglia, the resident immune cells of the central nervous system (CNS). Microglia are important for core processes of learning and memory, such as homeostatic maintenance and synaptic plasticity, which are negatively impacted by aging. We hypothesize that viral infection exacerbates aging-associated brain homeostatic impairment and inflammation, causing a cognitive impairment, notably via dysregulation of microglial functions as well as emergence of microglia sustaining a CNS pro-inflammatory balance. To test this hypothesis, we exposed 20-month-old male and female C57BL/6J mice to a single 5 mg/kg intraperitoneal dose of polyinosinic:polycytidylic acid (poly(I:C)), a viral mimic, which induced a severe acute sickness response within a 24-h period. A battery of behavioral paradigms including the open field test, novel and spatial object recognition test, as well as the Barnes maze, were employed to assess adaptability, anxiety-like behavior, learning, short- and long-term memory in the post-sickness phase at two timepoints. Brain samples from ventral hippocampus, a seat of learning and memory, were used to investigate microglial density, morphology and ultrastructure using epifluorescence, confocal and scanning electron microscopy. Our results show both sex- and/or treatment-dependent changes in locomotion, exploration, and short-term object memory within a 3- or 10-week period post challenge. Particularly, poly(I:C)-exposed female mice displayed an increased novel object discrimination ability compared to both saline-exposed control female or poly(I:C)-exposed male mice at 3 weeks. In the Barnes maze, on the other hand, poly(I:C)-exposed male mice showed a tendency toward an increased number of learning errors across the training period and a lower preference toward a hippocampus-associated search strategy in the probe test, suggesting learning impairment at 3 weeks after exposure. Preliminary analysis did not show changes in IBA1-positive(+)/TMEM119+ microglia density or distribution in the hippocampal CA1 stratum radiatum or stratum lacunosum-moleculare between poly(I:C)-challenged and control mice. Regardless of challenge, aged male mice displayed increased microglial density and neighbour distance compared to aged female mice. Qualitative analysis of microglial ultrastructure points to an elevated deposition of lipid vacuoles and lipofuscin granules in microglia of poly(I:C)-challenged mice. With the globally increasing older adult population, a better understanding of the processes underlying the cognitive aging and associated pathological conditions, such as dementia and Alzheimer’s disease, as well as of the outcomes of preventive and intervention strategies, holds utmost clinical implication for the future of brain health and cognitive aging.

# Abstract: P02.53 – The Beneficial Effects of NLY01 on Astrocyte Coverage and Functional Recovery in a Mouse Model of Stroke

**Published:** 2025-09-05

**Authors:** Julia Staton, Kimberly Prescott, Faith Bullock, John Holsten, Bella Readling, Elan Doyle, Shane Liddelow, Todd Peterson

**Affiliations:** a University of North Carolina Wilmington, Wilmington, NC, USA

## Abstract

Ischemic stroke was responsible for over 6.5 million deaths worldwide in 2022, and the current treatment options are very limited. During and after an insult, one of the main pathological cascades is neuroinflammation which can be beneficial or detrimental if it is not resolved appropriately. Understanding how the neuroinflammatory response mediates detrimental outcomes may lead to potential therapeutic options. Following an ischemic event, immune cells like microglia become activated to respond to the damaged tissue and release pro-inflammatory cytokines, some of which include TNF, IL-1α, and C1q (TIC). TIC released from microglia shifts astrocytes into a neurotoxic reactive phenotype (nRA), which has been shown to kill neurons and other cells, driving infarct expansion. We recently demonstrated the beneficial effects of a global knockout of TIC, which limited nRA induction after stroke. However, the glycogen-like peptide-1 receptor (GLP1R) agonist NLY01 is capable of inhibiting microglia from releasing TIC and is more clinically relevant. Evidence suggests that NLY01 reduces nRAs and improves outcomes in mouse models of Parkinson’s Disease and other neurodegenerative disorders. Therefore, we hypothesized that NLY01 treatment would reduce nRA formation, thereby improving functional recovery following stroke. To examine this, we induced photothrombotic stroke over the motor cortex in 22 male (C57BL/6j) mice and 56 female mice. At both 4- (r.o.) and 72 (i.p.) hr following stroke, we administered NLY01 (0, 1, 3, or 15 mg/kg), measured functional recovery of motor behavior at multiple time points, and then perfused animals 28 d post-stroke. We assessed motor behavior prior to stroke induction and 1-, 3-, 7-, 14- and 28 days post-stroke. We also measured astrocyte coverage (GFAP) following NLY01 treatment. In male mice, we did not find any significant differences in astrocyte (GFAP) coverage between the treatments (*p* = 0.7514) in the peri-infarct area. However, we did find improvements in functional recovery in NLY01 treated (3 mg/kg) mice with a reduced number of errors on the tapered beam (*p* = 0.0461) and increased distance traveled on the rotating beam (*p* = 0.0106). In female mice, the 3 mg/kg NLY01 group had significantly less astrocyte (GFAP) coverage in the peri-infarct area when compared to the 0 mg/kg group (*p* = 0.0104). We also found significant improvements in functional recovery within mice treated with NLY01. For tapered beam, the 3 mg/kg NLY01 group made significantly less foot faults (*p* = 0.01). As for rotating beam, the 3 mg/kg NLY01 group traveled further than the 0 mg/kg (*p* = 0.015) as well. Understanding the mechanistic underpinnings of neuroinflammation following stroke will lead to increased therapeutic options. NLY01 inhibits NRA formation, comparable to genetic deletion of TIC, but is more clinically relevant. Following stroke, it reduces astrocyte coverage and improves functional recovery. NLY01 improves the detrimental pathology that manifests following insult and after more preclinical testing may indicate a new effective treatment for stroke.

# Abstract: P02.54 – Transcriptome Analysis in Lumbosacral Dorsal Root Ganglia Revealed Sex-Specific Mechanisms Underlying Visceral Hypersensitivity

**Published:** 2025-09-05

**Authors:** Sathish Kumar Yesupatham, Alison Xiaoqiao Xie

**Affiliations:** a University of Colorado, Aurora, CO, USA

**Keywords:** Urological chronic pelvic pain syndrome (UCPPS), Sensory Nerves, Vascular Endothelial Growth Factor dorsal root ganglia, nociception, T regulatory cell, glial activation

## Abstract

**Introduction:**

The lumbosacral dorsal root ganglia (DRG)’s molecular processes underlying the symptoms of pelvic pain and overactive bladder are still unknown. In order to map the gene and signaling pathway alterations linked to the pathogenesis of pelvic pain, visceral hypersensitivity was induced in mice using a vascular endothelial growth factor (VEGFA)-induced mouse model of urological chronic pelvic pain syndrome (UCPPS). Bulk RNA sequencing transcriptome analysis was carried out on lumbosacral (L1-L2, L6-S2) DRGs. Coupled with ingenuity pathway analysis (IPA), our data unraveled some unique sex-differences in the pain perception during UCPPS.

**Methods:**

In this study, C57BL6/J mice, both male and female, were used. UCPPS symptoms including visceral mechanical hypersensitivity were induced by intravesical instillations of recombinant VEGFA, an animal model of UCPPS we previously established. The mice were then euthanized and the L1, L2, L6, S1, and S2 DRGs were dissected out for bulk RNA sequencing. Samples were sequenced at a depth of 40 million reads per sample. Quality control, Alignment to reference genome, differential gene expression was performed on Partek genomic platform. IPA (Qiagen) was performed to understand the changes in the canonical pathways associated with UCPPS symptoms.

**Results:**

In VEGFA-injected male and female mice (exhibiting visceral hypersensitivity), there was a differential expression of 110 genes and 176 genes, respectively, as compared to saline-instilled littermate control animals. surprisingly, there were only two genes that showed differential expression in both sexes (Sfrp4 and Angptl4). The pathway changes in the male mice instilled with VEGFA, when compared to saline-instilled control males, suggested glial cell activation, while the data from their female counterparts showed increased T-cells regulatory responses. Differences were also observed in the expression of distinct sets of nociceptive genes between sexes.

**Conclusions:**

Our data provides the first characterization of signaling pathway alterations in lumbosacral DRGs that occur during the development of UCPPS symptoms using animal models. For the first time, sex differences in lumbosacral DRG transcriptome at the baseline is reported. Additionally, our data revealed sex-specific changes in signaling pathways that contribute to nociceptive responses. In females, visceral hypersensitivity is associated with strong T-reg cells responses, while in males, visceral hypersensitivity is correlated to glial activation mechanisms. Lastly, our research identified potential targets in the PPAR signaling pathway which are up-regulated in both sexes correlated to UCPPS symptoms.

# Abstract: P02.55 – The Role of Oligodendrocyte Specific Serpina3n in Physiological and Pathological Condition

**Published:** 2025-09-05

**Authors:** Meina Zhu, Yan Wang, Joohyun Park, Fuzheng Guo

**Affiliations:** a Department of Neurology, School of Medicine, UC Davis, Sacramento, NC, USA; b Institute for Pediatric Regenerative medicine (IPRM), Shriners Hospitals for Children – Northern California, Sacramento, NC, USA

## Abstract

Serine protease inhibitor (Serpin) clade A member 3n (Serpina3n) and its human orthologue SERPINA3, also known as α1-antichymotrypsin (ACT), are acute-phase inflammatory glycoproteins. It has been widely accepted by the neuroscience community that Serpina3n/SERPINA3 is a biomarker of reactive astrocytes. Yet, recent data suggest that oligodendrocytes, the myelin-forming cells in the CNS, may be the primary cellular source of Serpina3n/SERPINA3 under neurological conditions. To address the specific in vivo genetic requirements for Serpina3n during oligodendrocyte development and demyelination, we generated an oligodendrocyte-specific Serpina3n knockout mouse line, Olig2 Cre; Serpina3n fl/fl. Examining the expression of Serpina3n in the developing central nervous system (CNS) revealed a unique pattern in the oligodendrocyte lineage in white matter, with particularly enriched expression in differentiated oligodendrocytes. However, the conditional knockout (cKO) of Serpina3n from Olig2-expressing cells did not affect motor and cognitive functions in mice. In investigating the role of Serpina3n in demyelination, we utilized an animal model of primary demyelination induced by oligodendrocyte injury (Cuprizone), mimicking Pattern III/IV multiple sclerosis lesions in the corpus callosum. In this model, Serpina3n was dysregulated primarily in oligodendrocytes, both intracellularly and secreted extracellularly in Serpina3n cKO. Notably, oligodendrocytes and myelin were preserved in the Serpina3n-deficient group compared to the control, suggesting that depleting oligodendrocyte-derived Serpina3n is oligodendroglial protective. These findings indicate that oligodendrocyte-specific Serpina3n is dispensable for regulation in brain development and myelination. Moreover, oligodendrocytes emerge as the major cellular source of CNS Serpina3n during demyelinating injury, justifying the use of an oligodendrocyte-specific Cre line for Serpina3n cKO. This study provides the first conceptual landscape of the functional significance of Serpina3n in both physiological and pathological conditions.

# Abstract: Index

**Published:** 2025-09-05

**Table:** 

Abalde-Atristain	49	P01.001
Adeniyi	37	OR01.01
Adkins-Travis	50	P01.002
Ahmad	51	P01.003
Alam	52	P01.004
Alemani	53	P01.005
Anderson	54	P01.006
Andonova	55	P01.007
Andonova	201	P02.34
Angeliu	56	P01.008
Avetisyan	48	OR02.06
Baderinwa	57	P01.009
Baker	58	P01.010
Barger	202	P02.35
Beiter	59	P01.011
Besch	203	P02.36
Bhupana	60	P01.012
Bieberich	61	P01.013
Bojovic	62	P01.014
Brashaw	63	P01.015
Buck	64	P01.016
Butler	65	P01.017
Cadet	66	P01.018
Cai	41	OR01.05
Cardona	204	P02.37
Chandler	67	P01.019
Chandrakantan	68	P01.020
Chandrasekaran	69	P01.021
Charest	168	P02.01
Cook	205	P02.38
Copenhaver	70	P01.022
Corral	71	P01.023
Corty	72	P01.024
Cotter	73	P01.025
Cruz Berrios	74	P01.026
DeLucia	44	OR02.02
Dhari	75	P01.027
Doan	76	P01.028
Domowicz	77	P01.029
Drew	78	P01.030
Duan	79	P01.031
Duran Laforet	80	P01.032
Ebrahimi	81	P01.033
Elbaz	82	P01.034
Emberley	83	P01.035
Estes	84	P01.036
Ethell	85	P01.037
Falk	86	P01.038
Feri	87	P01.039
Fernandez Fernandez	88	P01.040
Festa	89	P01.041
Firdauzy	90	P01.042
Flury	91	P01.043
Frazier	47	OR02.05
Friedman	92	P01.044
Friesen	206	P02.39
Gadelkarim	93	P01.045
Gallagher	94	P01.046
Gallo	95	P01.047
Garbarini	97	P01.048
Georges	207	P02.40
Gimenez Gomez	43	OR02.01
Gray	98	P01.049
Gujar	99	P01.050
Haak	100	P01.051
Haque	208	P02.41
Harmon	101	P01.052
Harmon	102	P01.053
Hashimoto	103	P01.054
Haspula	104	P01.055


Heo	105	P01.056
Holsten	106	P01.057
Houmam	107	P01.058
Hu	108	P01.059
Hu	109	P01.060
Huang	110	P01.061
Imani Farahani	111	P01.062
Jaramillo	112	P01.063
Jay	113	P01.064
Jeffries	114	P01.065
Jeong	115	P01.066
Jones	209	P02.42
Kang	116	P01.067
Kantak	117	P01.068
Kayed	118	P01.069
Khatibi	119	P01.070
Kim	120	P01.071
Kim	121	P01.072
Klegeris	210	P02.43
Knies	122	P01.073
Lapato	123	P01.074
Laughlin	211	P02.44
Leitzel	212	P02.45
Li	38	OR01.02
Lietzke	124	P01.075
Lin	125	P01.076
Long	39	OR01.03
Louie	126	P01.077
Louie	127	P01.078
Lundt	128	P01.079
Manavi	129	P01.080
Manzo	130	P01.081
Martin	131	P01.082
Martinez-Lozada	40	OR01.04
Mather	132	P01.083
McAtee	133	P01.084
McCallum	134	P01.085
McDonald	135	P01.086
McFarland	136	P01.087
Mcgrath	137	P01.088
Meares	138	P01.089
Mendoza Silva	139	P01.090
Mittra	140	P01.091
Morganti	141	P01.092
Moss	213	P02.46
Munoz-Ballester	142	P01.093
Nazir	143	P01.094
Nevin	144	P01.095
Nguyen	145	P01.096
Nguyen	146	P01.097
Niemeyer	147	P01.098
Niou	214	P02.47
Nobuta	148	P01.099
Oldham	149	P01.100
Osborne	150	P01.101
Oseas	151	P01.102
Packer	215	P02.48
Park	216	P02.49
Pennypacker	152	P01.103
Peters	153	P01.104
Petratos	217	P02.50
Piller	154	P01.105
Puleo	155	P01.106
Quadri	156	P01.107
Athira	157	P01.108
Ravichandar	158	P01.109
Ren	159	P01.110
Sanchez	160	P01.111
Sandau	46	OR02.04
Sapkota	161	P01.112


Saugstad	162	P01.113
Sauter	163	P01.114
Sayed	218	P02.51
Scavuzzo	164	P01.115
Schofield	169	P02.02
Scholz	170	P02.03
Seeger	171	P02.04
Senatore	172	P02.05
Shalih Maraicar	173	P02.06
Sheehan	174	P02.07
Shen	175	P02.08
Simoncicova	219	P02.52
Sims	176	P02.09
Singh	165	P01.116
Sompol	177	P02.10
Spillman	178	P02.11
Sriram	179	P02.12
Srivastava	180	P02.13
Stackhouse	181	P02.14
Staton	220	P02.53
Stunault	182	P02.15
Subair	183	P02.16


Sullivan	184	P02.17
Tabaie	185	P02.18
Tandon	186	P02.19
Tavares	45	OR02.03
Traetta	187	P02.20
Tripathi	188	P02.21
Van Eldik	189	P02.22
von Krusenstiern	190	P02.23
Wagner	191	P02.24
Wang	166	P01.117
Weigel	167	P01.118
Wong	42	OR01.06
Wong	192	P02.25
Woodruff	193	P02.26
Woodruff	194	P02.27
Yaeger	195	P02.28
Yesupatham	221	P02.54
Zamora	196	P02.29
Zhang	197	P02.30
Zhu	198	P02.31
Zhu	222	P02.55
Zimmerman	199	P02.32
Zupan	200	P02.33
